# Design and Synthesis of Novel Morpholine-Derived Nitrogen-Rich Scaffolds as Multifunctional Anticancer and Antibacterial Agents: Biological Evaluation and Computational Studies

**DOI:** 10.3390/pharmaceutics18080982

**Published:** 2026-08-09

**Authors:** Hagar S. El-Hema, Esraa Adel, Wagdy I. El-Dougdoug, Ashraf A. F. Wasfy, Ahmed F. El-Sayed, Eman S. Nossier, Modather F. Hussein, Reem Binsuwaidan, Asmaa Saleh, Adel A. -H. Abdel-Rahmanh

**Affiliations:** 1 Basic Science Department (Chemistry), Thebes Higher Institute for Engineering, Thebes Academy, Maadi 11434, Egypt; 2 Chemistry Department, Faculty of Science, Benha University, Benha 13518, Egypt; israa20974@fsc.bu.edu.eg (E.A.); wagdi.ali@fsc.bu.edu.eg (W.I.E.-D.); ashraf.farouk@fsc.bu.edu.eg (A.A.F.W.); 3 Microbial Genetics Department, Biotechnology Research Institute, National Research Centre, Giza 12622, Egypt; ahmedfikry.nrc@gmail.com; 4 Egypt Center for Research and Regenerative Medicine (ECRRM), Cairo 11381, Egypt; 5 Pharmaceutical Medicinal Chemistry and Drug Design Department, Faculty of Pharmacy (Girls), Al-Azhar University, Cairo 11754, Egypt; dr.emannossier@gmail.com; 6 Chemistry Department, College of Science, Jouf University, P.O. Box 2014, Sakaka 72341, Aljouf, Saudi Arabia; mfhussin@ju.edu.sa; 7 Department of Pharmaceutical Sciences, College of Pharmacy, Princess Nourah Bint Abdulrahman University, P.O. Box 84428, Riyadh 11671, Riyadh Province, Saudi Arabia; rabinsuwaidan@pnu.edu.sa (R.B.); asali@pnu.edu.sa (A.S.); 8 Chemistry Department, Faculty of Science, Menoufia University, Shebin El-Kom 32511, Egypt

**Keywords:** morpholine derivatives, nitrogen-rich heterocycles, EGFR/PI3K/mTOR inhibition, anticancer activity, antibacterial activity, antibiofilm activity, antioxidant activity, molecular dynamics simulation

## Abstract

**Background/Objectives:** The development of multifunctional small molecules capable of simultaneously addressing cancer progression and antimicrobial resistance represents an important challenge in medicinal chemistry. This study aimed to design, synthesize, and biologically evaluate a series of novel morpholine-based nitrogen-rich heterocyclic hybrids as potential anticancer and antibacterial agents, supported by computational investigations. **Methods:** Twelve morpholine-derived nitrogen-enriched heterocyclic hybrids incorporating pyran, triazine, pyrimidinone, and sulfur-containing scaffolds were synthesized and fully characterized using IR, ^1^H NMR, ^13^C NMR, mass spectrometry, and elemental analysis. Their antiproliferative activities were evaluated against MCF-7 and HCT-116 cancer cell lines. The most active compounds were further investigated through kinase inhibition assays, cell cycle analysis, apoptosis, mitochondrial membrane potential, intracellular ROS determination, and apoptosis-related gene expression. Antibacterial, antibiofilm, antioxidant, and computational studies, including molecular docking, molecular dynamics simulations, MM-GBSA/MM-PBSA binding free-energy calculations, DFT calculations, and ADMET prediction, were also performed. **Results:** Compounds **3**, **10**, and **12** exhibited the highest antiproliferative activity, with compound **10** emerging as the lead candidate. It potently inhibited EGFR, PI3K, and mTOR, with IC_50_ values of 0.086 ± 0.003, 0.107 ± 0.005, and 0.223 ± 0.008 μM, respectively. Mechanistic investigations revealed G_2_/M arrest in MCF-7 cells and G_0_/G_1_ arrest in HCT-116 cells, accompanied by apoptosis rates of 32.66% and 37.12%; mitochondrial membrane depolarization; a 3.55-fold increase in intracellular ROS; upregulation of caspase-3, caspase-9, and Bax; and downregulation of Bcl-2, supporting activation of the intrinsic apoptotic pathway. Compound **10** also displayed the broadest antibacterial spectrum, surpassed ciprofloxacin against several tested isolates, exhibited MIC values of 5–20 μg/mL, achieved 42.80% inhibition of *Pseudomonas aeruginosa* biofilm formation, and showed the strongest antioxidant activity in DPPH and ABTS assays. Computational analyses supported the experimental findings by predicting stable interactions with EGFR and *Staphylococcus aureus* DNA gyrase, together with favorable MM-GBSA/MM-PBSA binding free energies of −23.44 and −24.99 ± 2.71 kcal/mol, respectively. **Conclusions:** The present findings identify compound **10** as a promising multifunctional lead with potent anticancer, antibacterial, antibiofilm, antioxidant, and multitarget kinase inhibitory activities. The combined biochemical, cellular, and computational findings support the proposed involvement of the EGFR/PI3K/mTOR signaling pathway in its antiproliferative activity and identify DNA gyrase as a potential antibacterial target. Nevertheless, the present study is limited to in vitro biological evaluation and computational investigations. Therefore, further in vivo efficacy studies, pharmacokinetic profiling, toxicity assessment, and experimental validation of the proposed molecular targets are warranted before considering preclinical development.

## 1. Introduction

**Figure 1:**
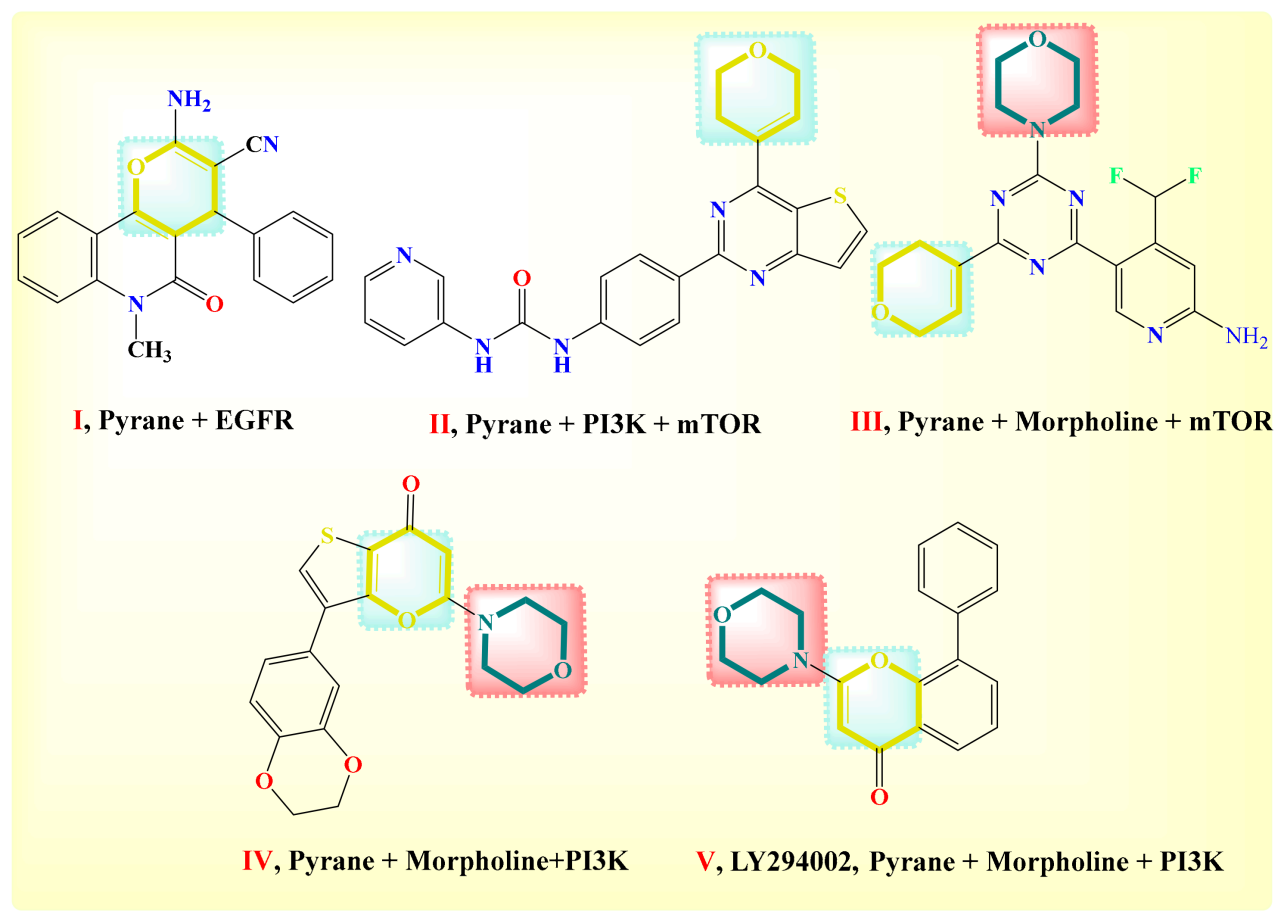
Representative pyran-containing and pyran–morpholine hybrid derivatives exhibiting potent inhibitory activities against EGFR, PI3Kα, and mTOR signaling pathways.

Despite substantial progress in early diagnosis and therapeutic interventions, cancer remains one of the leading causes of death worldwide [1,2,3,4,5,6]. Among the most frequently diagnosed and aggressive malignancies are breast [7,8] and colorectal cancers [9,10], which are characterized by high incidence, metastatic potential, and increasing resistance to conventional chemotherapy. The clinical effectiveness of current chemotherapeutic agents is further compromised by treatment-associated toxicity, poor selectivity toward malignant cells, acquired drug resistance, and frequent tumor recurrence. Consequently, considerable efforts have been devoted to developing novel anticancer agents with enhanced efficacy and improved safety profiles [11,12,13,14]. In this context, targeting dysregulated intracellular signaling pathways that regulate cancer cell proliferation and survival has emerged as a promising strategy in contemporary anticancer drug discovery [15]. Among these pathways, epidermal growth factor receptor (EGFR) [16,17,18], phosphatidylinositol-3-kinase (PI3K) [19,20], and mammalian target of rapamycin (mTOR) [21,22,23] play crucial roles in tumor growth, angiogenesis, metastasis, and resistance to apoptosis. Therefore, inhibition of these molecular targets has attracted considerable attention for the development of potent anticancer agents [24]. Owing to their remarkable structural diversity and wide range of biological activities, heterocyclic scaffolds have become indispensable building blocks in medicinal chemistry and drug discovery [25,26,27,28,29,30,31,32,33,34]. Particularly, morpholine-containing derivatives have gained significant interest owing to their remarkable biological properties and favorable pharmacokinetic characteristics [35]. The morpholine moiety is a common structural feature in several clinically approved kinase inhibitors, where it contributes to improving aqueous solubility, membrane permeability, and target binding affinity [36]. Nitrogen-containing heterocyclic scaffolds such as pyran, triazine, and pyrimidinone derivatives have attracted considerable attention as promising anticancer pharmacophores owing to their remarkable inhibitory activities against several oncogenic targets, including EGFR, PI3K, and mTOR. In particular, both pyran-based derivatives and pyran–morpholine hybrids have demonstrated significant kinase inhibitory activities and promising anticancer potential, highlighting the value of combining morpholine with nitrogen-rich heterocyclic frameworks in the development of novel anticancer agents. Pyran-containing scaffolds have attracted considerable interest as anticancer pharmacophores because of their ability to inhibit several oncogenic kinases. Representative pyranoquinoline derivatives have shown potent EGFR inhibitory activity [37], whereas pyran-based hybrids have also demonstrated remarkable dual PI3Kα/mTOR inhibition [38]. Likewise, tetrahydro-2H-pyran-substituted triazine–morpholine derivatives exhibited potent mTOR inhibitory activity [39]. Furthermore, incorporation of the morpholine moiety into pyran/chromene frameworks significantly enhanced PI3K inhibition, as demonstrated by morpholine-containing pyran derivatives [40] and the well-known PI3K inhibitor LY294002 [41] (Figure 1).

**Figure 2:**
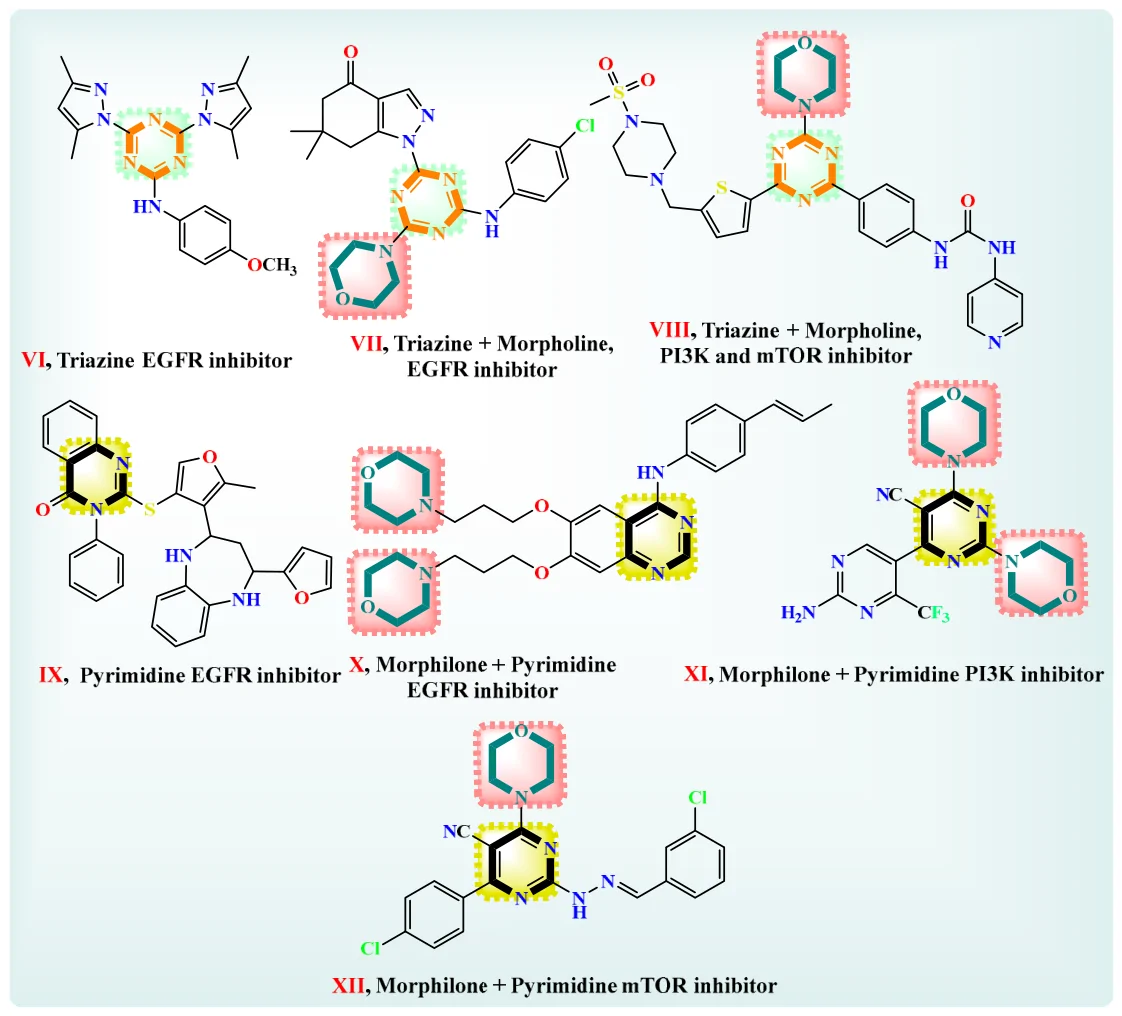
Representative triazine- and pyrimidinone-containing heterocyclic derivatives exhibiting potent inhibitory activities against EGFR, PI3K, and mTOR signaling pathways.

Triazine- and pyrimidinone-containing heterocyclic derivatives have attracted considerable attention as promising kinase-targeted anticancer agents owing to their potent inhibitory activities against EGFR, PI3K, and mTOR signaling pathways. These nitrogen-rich heterocyclic scaffolds are considered important bioisosteric pharmacophores in medicinal chemistry because of their structural similarity and remarkable ability to interact with kinase active sites. Representative triazine derivatives have demonstrated potent EGFR inhibitory activity [42], while morpholine-substituted triazine analogues effectively inhibited EGFR and suppressed the PI3K/AKT/mTOR signaling pathway [43]. Furthermore, morpholine–triazine hybrids exhibited promising dual PI3Kα/mTOR inhibitory activity, highlighting their potential as multitarget kinase inhibitors [44]. Likewise, pyrimidinone derivatives have shown significant anticancer activity through EGFR inhibition [45], whereas morpholine-containing pyrimidine derivatives displayed potent inhibitory activities against EGFR, PI3K, and mTOR, further emphasizing the value of integrating morpholine with nitrogen-rich heterocyclic pharmacophores for the development of targeted anticancer agents [46,47,48] (Figure 2).

**Figure 3:**
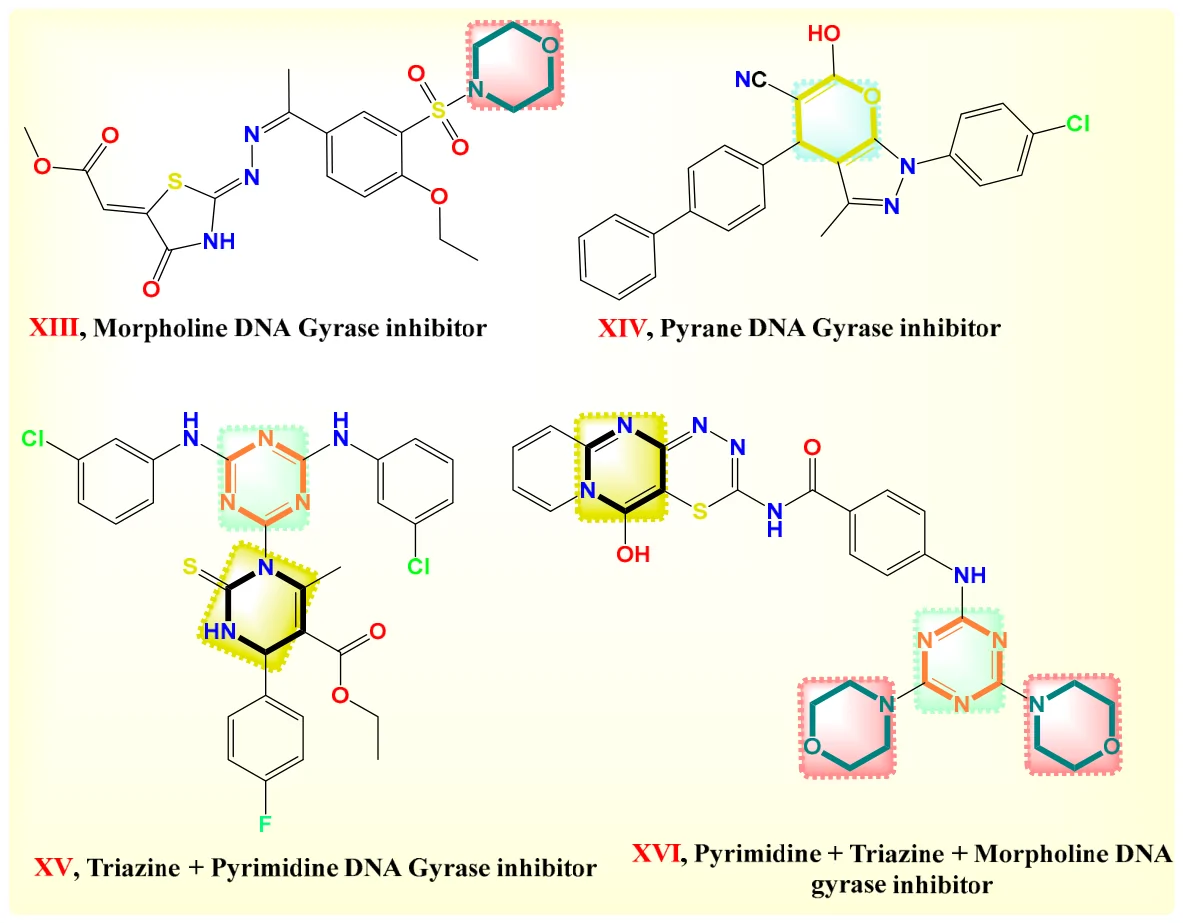
Representative heterocyclic derivatives containing morpholine, pyran, triazine, and pyrimidine pharmacophores exhibiting potent antimicrobial activity through DNA gyrase inhibition.

Besides cancer progression, microbial infections remain a serious complication in immunocompromised cancer patients, particularly during chemotherapy. The increasing prevalence of multidrug-resistant microbial strains has intensified the need for the development of novel antimicrobial agents with improved therapeutic efficacy. Among the validated antibacterial targets, DNA gyrase is considered one of the most attractive enzymes owing to its essential role in bacterial DNA replication and transcription. Accordingly, several heterocyclic scaffolds incorporating morpholine, pyran, triazine, and pyrimidine pharmacophores have demonstrated promising antimicrobial activity through DNA gyrase inhibition. Representative morpholine-containing derivatives [49], pyranopyrazole analogues [50], triazine-based compounds [51], and morpholine-substituted pyrimidine–triazine hybrids [52] have all shown significant DNA gyrase inhibitory activity, highlighting the antimicrobial potential of these privileged heterocyclic scaffolds (Figure 3).

Although morpholine-, pyran-, triazine-, pyrimidinone-, and sulfur-containing heterocyclic scaffolds have individually demonstrated remarkable anticancer and antimicrobial activities through modulation of clinically relevant molecular targets, including EGFR, PI3K/mTOR, and DNA gyrase [37,38,39,40,41,42,43,44,45,46,47,48,49,50,51,52], their integration into multifunctional molecular hybrids remains relatively unexplored. Considering that molecular hybridization of complementary pharmacophores may simultaneously improve target affinity, physicochemical properties, and multitarget therapeutic efficacy, the development of novel nitrogen-rich heterocyclic hybrids incorporating these privileged structural motifs represents a promising strategy for the discovery of new anticancer and antimicrobial agents. To the best of our knowledge, no previous study has reported the design of morpholine-based multifunctional hybrids simultaneously integrating pyran, triazine, pyrimidinone, and sulfur-containing pharmacophores within a single molecular framework for the concurrent targeting of EGFR, PI3K/mTOR, and DNA gyrase.

### Rationale for the Study

Inspired by the reported biological activities of **Gefitinib** [53], **Copanlisib** [54], and **Zoliflodacin** [55] as clinically established inhibitors of EGFR, PI3K/mTOR, and DNA gyrase, respectively, and considering the unexplored potential of integrating multiple privileged pharmacophores into a single molecular framework, a molecular hybridization strategy was adopted for the design of the target compounds. Structural modification of the selected pharmacophores was achieved through incorporation of the morpholine moiety owing to its recognized role in improving aqueous solubility, membrane permeability, and kinase-binding affinity, while the incorporation of pyran, triazine, pyrimidinone, and sulfur-containing heterocyclic frameworks was anticipated to provide complementary interactions with multiple biological targets.

**Figure 4:**
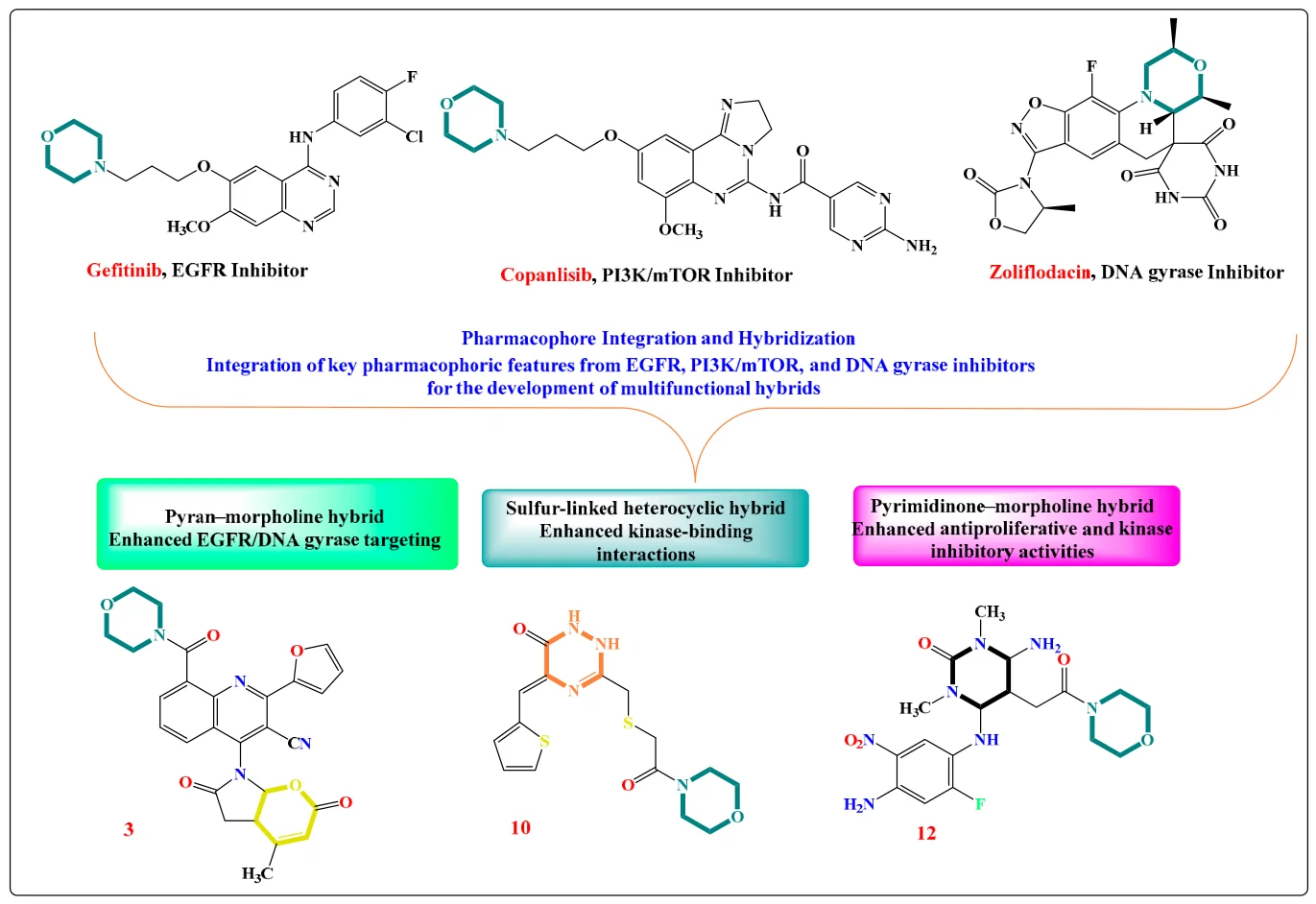
Schematic overview of the molecular design approach employed to generate multifunctional morpholine-containing heterocyclic hybrids by integrating key pharmacophoric features of reported EGFR, PI3K/mTOR, and DNA gyrase inhibitors.

Accordingly, compound **3** was designed by hybridizing the morpholine scaffold with a fused pyran-containing heterocyclic system and a cyano functionality to enhance EGFR inhibitory activity while potentially improving interactions with bacterial DNA gyrase. Compound **10** incorporated a sulfur-linked nitrogen-rich heterocyclic framework together with the morpholine moiety to increase structural flexibility and strengthen interactions with PI3K/mTOR-associated binding sites. In compound **12**, the introduction of pyrimidinone, amino, and nitro functionalities was expected to enhance antiproliferative activity by increasing hydrogen-bonding capability and optimizing electronic interactions within the active sites of the targeted enzymes. Collectively, these structural modifications were intended to generate multifunctional heterocyclic hybrids capable of simultaneously targeting EGFR, PI3K/mTOR, and DNA gyrase, thereby improving both anticancer and antimicrobial activities (Figure 4).

Based on the above rationale and the identified knowledge gap, and motivated by our continuing interest in the development of biologically active heterocyclic frameworks, we designed and synthesized a new library of morpholine-containing derivatives incorporating diverse nitrogen-rich heterocyclic pharmacophores. The synthesized compounds were evaluated for their antiproliferative activity against human breast (MCF-7) and colorectal (HCT-116) cancer cell lines, together with their antibacterial activity against selected microbial strains. To elucidate their underlying mechanisms of action, the most promising compounds were further investigated through enzyme inhibition assays targeting EGFR, PI3K, mTOR, and DNA gyrase. These experimental investigations were complemented by molecular docking, molecular dynamics simulations, MM-GBSA/MM-PBSA binding free-energy calculations, and additional mechanistic biological studies to provide a comprehensive evaluation of their therapeutic potential.

## 2. Materials and Methods

### 2.1. Chemistry

***(2-Aminophenyl)(morpholino)methanone (A)***

Anthranilic acid (11 mmol, 1.51 g) was dispersed in anhydrous dichloromethane (30 mL), after which thionyl chloride (15 mmol, 1.10 mL) and triethylamine (0.5 mL) were introduced. The reaction mixture was heated under reflux at 40 °C for 4 h to generate the corresponding acid chloride in situ. Without isolation of this intermediate, morpholine (10 mmol, 0.86 mL) was added dropwise, and heating was continued at 40 °C for an additional 6 h. Reaction progress was followed by TLC using DCM/MeOH (9:1) as the eluent (Rf = 0.45). Upon completion, the mixture was allowed to cool, poured onto crushed ice, neutralized with saturated aqueous NaHCO_3_, and the resulting solid was collected by filtration. The crude product was washed thoroughly with water and recrystallized from ethanol to furnish compound **A** as an off-white solid (85% yield, m.p. 165–167 °C). IR (KBr, ν max, cm^−1^): 3427, 3388 (NH_2_), 3058 (Ar–CH), 2925, 2855 (aliphatic CH), 1676 (C=O), 1607 (C=C). ^1^H NMR (400 MHz, DMSO-*d*_6_) δ (ppm): 2.79 (t, 4H, 2CH_2_–N), 3.67 (t, 4H, 2CH_2_–O), 5.05 (br s, 2H, NH_2_, exchangeable), 6.59–6.73 (m, 4H, Ar–H). ^13^C NMR (100 MHz, DMSO-*d*_6_) δ (ppm): 45.09 (2CH_2_–N), 65.56 (2CH_2_–O), 122.48–133.98 (Ar–C), 148.81 (Ar–C–NH_2_), 168.49 (C=O). Anal. Calcd for C_11_H_14_N_2_O_2_ (206.24): C, 64.06; H, 6.84; N, 13.58%. Found: C, 64.05; H, 6.82; N, 13.58%.

***2-Chloro-1-morpholinoethan-1-one (B)***

Morpholine (10 mmol, 0.86 mL) was dissolved in anhydrous acetone (25 mL), followed by the addition of potassium carbonate (15 mmol, 2.07 g). The reaction mixture was cooled to 0–5 °C, and chloroacetyl chloride (11 mmol, 0.88 mL) was added slowly under constant stirring. After complete addition, the reaction was allowed to warm to room temperature and stirred for 8 h. The reaction was monitored by TLC using hexane/ethyl acetate (7:3) as the developing solvent (Rf = 0.55). The inorganic salts were separated by filtration, and the filtrate was concentrated under reduced pressure. The resulting residue was poured onto crushed ice, neutralized with saturated aqueous NaHCO_3_, and the precipitated solid was isolated by filtration. After washing thoroughly with water, the product was recrystallized from ethanol to afford compound **B** as a white solid (75% yield, m.p. 96–98 °C). IR (KBr, νcm^−1^): 2975, 2917, 2853 (aliphatic CH), 1670 (amide C=O). ^1^H NMR (400 MHz, DMSO-*d*_6_) δ (ppm): 2.89 (t, 4H, 2CH_2_–N), 3.59 (t, 4H, 2CH_2_–O), 4.53 (s, 2H, CH_2_–Cl). ^13^C NMR (100 MHz, DMSO-*d*_6_) δ (ppm): 41.87 (CH_2_–Cl), 45.58 (2CH_2_–N), 65.83 (2CH_2_–O), 163.67 (C=O). Anal. Calcd for C_6_H_10_ClNO_2_ (163.60): C, 44.05; H, 6.16; N, 8.56%. Found: C, 44.04; H, 6.16; N, 8.58%.

***4-Amino-2-(furan-2-yl)-8-(morpholine-4-carbonyl)quinoline-3-carbonitrile (1)***

Intermediate **A** (N-(anthraniloyl)morpholine) (10 mmol, 2.06 g) was dissolved in ethanol (30 mL), followed by the addition of the corresponding arylidene malononitrile derivative (11 mmol, 1.44 g) and triethylamine (0.5 mL). The reaction mixture was heated under reflux at 78 °C for 6–8 h, while its progress was periodically checked by TLC using DCM/MeOH (9:1) as the mobile phase (Rf = 0.40). After completion, the reaction was allowed to cool naturally to room temperature before being poured onto crushed ice. The precipitated solid was collected by filtration, washed thoroughly with water, and purified by recrystallization from ethanol to furnish compound **1** as a yellow solid (80% yield, m.p. 180–182 °C). The obtained product corresponds to a fused quinoline (pyridine) derivative incorporating a cyano substituent. IR (KBr, ν max, cm^−1^): 3482, 3423 (NH_2_), 3075, 3055 (Ar-CH), 2926, 2856 (aliphatic CH), 2191 (CN), 1679 (C=O), 1617 (C=N), 1515, 1449 (C=C). ^1^H NMR (400 MHz, DMSO-*d*_6_) δ (ppm): 2.57 (t, 4H, 2CH_2_–N), 3.69 (t, 4H, 2CH_2_–O), 6.02 (br s, 2H, NH_2_, exchangeable), 6.59–7.88 (m, 6H, Ar–H). ^13^C NMR (100 MHz, DMSO-*d*_6_) δ (ppm): 45.12 (2CH_2_–N), 64.94 (2CH_2_–O), 87.17 (C–CN, pyridine), 113.95 (CN), 119.46–153.03 (Ar–C), 156.35 (C–NH_2_), 163.00 (C=N), 166.08 (C=O). Anal. Calcd for C_19_H_16_N_4_O_3_ (348.36): C, 65.51; H, 4.63; N, 16.08%. Found: C, 65.50; H, 4.63; N, 16.08%.

***4-(2,5-Dioxopyrrolidin-1-yl)-2-(furan-2-yl)-8-(morpholine-4-carbonyl)quinoline-3-carbonitrile (2)***

Compound **1** (10 mmol, 3.48 g) was dissolved in methanol (30 mL), after which succinic anhydride (11 mmol, 1.10 g) and potassium hydroxide (10 mmol, 0.56 g) were added sequentially. The resulting mixture was heated under reflux at 65 °C for 6 h. Reaction progress was followed by TLC using DCM/MeOH (9:1) as the developing solvent (Rf = 0.35). Upon completion, the reaction solution was allowed to reach room temperature and then poured onto crushed ice. The separated solid was collected by filtration, washed thoroughly with water, and purified by recrystallization from ethanol to furnish compound **2** as an off-white solid (75% yield, m.p. 210–212 °C). The isolated product was identified as a fused heterocyclic derivative incorporating a succinimide moiety. IR (KBr, ν max, cm^−1^): 3071, 3051 (Ar-CH), 2988, 2935, 2853 (aliphatic CH), 2192 (CN), 1682 (C=O, imide), 1658 (C=O, amide), 1607 (C=N), 1511, 1465 (C=C). ^1^H NMR (400 MHz, DMSO-d_6_) δ (ppm): 2.46 (t, 4H, 2CH_2_, succinimide), 2.79 (t, 4H, 2CH_2_–N), 3.80 (t, 4H, 2CH_2_–O), 6.61–7.89 (m, 6H, Ar–H). ^13^C NMR (100 MHz, DMSO-d_6_) δ (ppm): 30.03 (2CH_2_, succinimide), 45.33 (2CH_2_–N), 64.56 (2CH_2_–O), 87.50 (C–CN, pyridine), 114.01 (CN), 119.00–150.55 (Ar–C), 158.28 (C–N), 162.88 (C=N), 166.15 (C=O, amide), 176.03 (2C=O, imide). Anal. Calcd for C_23_H_18_N_4_O_5_ (430.41): C, 64.18; H, 4.22; N, 13.02%. Found: C, 64.20; H, 4.22; N, 13.01%.

***2-(Furan-2-yl)-4-(4-methyl-2,6-dioxo-4a,5,6,7a-tetrahydropyrano [2,3-b]pyrrol-7(2H)-yl)-8-(morpholine-4-carbonyl)quinoline-3-carbonitrile* *(3)***

Compound **2** (19 mmol, 8.17 g) was placed in a 25 mL round-bottom flask and dissolved in concentrated sulfuric acid (5–6 mL). Ethyl acetoacetate (20 mmol, 2.60 mL) was then introduced dropwise with continuous magnetic stirring. The reaction mixture was maintained at 50 °C for 1 h. The course of the reaction was monitored by TLC using DCM/MeOH (9:1) as the mobile phase (Rf = 0.30). After completion, the reaction mixture was allowed to cool to ambient temperature before being carefully poured into 200 mL of cold water. The precipitated product was isolated by filtration, washed thoroughly with water, and purified by recrystallization from ethyl acetate to give compound **3** as a light-yellow crystalline solid (69% yield, m.p. 230–232 °C). The isolated material was assigned as a fused pyranopyrroledione derivative. IR (KBr, ν max, cm^−1^): 3087, 3050 (Ar-CH), 2988, 2942, 2848 (aliphatic CH), 2211 (CN), 1685 (C=O, imide), 1655 (C=O, amide), 1633 (C=O, pyranone), 1583 (C=N), 1522, 1495, 1445 (C=C). ^1^H NMR (400 MHz, DMSO-*d*_6_) δ (ppm): 1.81 (s, 3H, CH_3_), 2.50 (d, 2H, CH_2_, succinimide), 2.89 (t, 4H, 2CH_2_–N), 3.15 (q, 1H, CH, succinimide), 3.78 (t, 4H, 2CH_2_–O), 5.39 (s, 1H, CH, pyranone), 6.62 (d, 1H, CH, succinimide), 6.94–7.47 (m, 6H, Ar–H).^13^C NMR (100 MHz, DMSO-*d*_6_) δ (ppm): 26.45 (CH_3_), 30.79 (CH), 35.62 (CH_2_), 45.51 (2CH_2_–N), 66.00 (2CH_2_–O), 87.50 (C–CN), 88.77 (C–N), 114.31 (CN), 122.52–141.91 (Ar–C), 142.23, 153.35 (Ar–C–O), 159.15 (C–N), 162.33 (C=N), 166.06 (C=O), 167.67 (C=O, pyranone), 170.00 (C–CH_3_), 176.13 (C=O, imide). MS (EI): m/z = 498 (M^+^), 263 (base peak). Anal. Calcd for C_27_H_22_N_4_O_6_ (498.50): C, 65.06; H, 4.45; N, 11.24%. Found: C, 65.06; H, 4.47; N, 11.24%.

***(Z)-2-(Furan-2-yl)-N’-hydroxy-4-(4-methyl-2,6-dioxo-4a,5,6,7a-tetrahydropyrano [2,3-b]pyrrol-7(2H)-yl)-8-(morpholine-4-carbonyl)quinoline-3-carboximidamide (4)***

Compound **3** (10 mmol, 4.98 g) was dissolved in ethanol (30 mL), after which hydroxylamine hydrochloride (12 mmol, 0.83 g) and anhydrous potassium carbonate (15 mmol, 2.07 g) were added. The resulting reaction mixture was heated under reflux at 78 °C for 6 h. Progress of the transformation was followed by TLC using DCM/MeOH (9:1) as the developing solvent (Rf = 0.25). Upon completion, the mixture was allowed to cool to ambient temperature and filtered to separate the inorganic salts. The filtrate was concentrated under reduced pressure, and the remaining residue was poured onto crushed ice. The precipitated product was collected by filtration, washed thoroughly with water, and purified by recrystallization from ethanol to give compound **4** as a pale-yellow solid (82% yield, m.p. 243–245 °C). The isolated compound was identified as an amidoxime-functionalized fused heterocyclic derivative. IR (KBr, ν max, cm^−1^): 3412 (OH), 3392, 3313 (NH_2_), 3085, 3053, 3016 (Ar-CH), 2985, 2942, 2842 (aliphatic CH), 1694 (C=O, imide), 1650 (C=O, amide), 1619 (C=O, pyranone), 1560 (C=N), 1500, 1443 (C=C). ^1^H NMR (400 MHz, DMSO-*d*_6_) δ (ppm): 1.81 (s, 3H, CH_3_), 2.38 (d, 2H, CH_2_, succinimide), 2.91 (t, 4H, 2CH_2_–N), 3.59 (q, 1H, CH, succinimide), 3.89 (t, 4H, 2CH_2_–O), 5.42 (s, 1H, CH, pyranone), 6.59 (d, 1H, CH, succinimide), 6.86 (br s, 2H, NH_2_, exchangeable), 7.16–7.52 (m, 6H, Ar–H), 9.44 (br s, 1H, OH). ^13^C NMR (100 MHz, DMSO-d_6_) δ (ppm): 25.99 (CH_3_), 31.19 (CH), 35.15 (CH_2_), 46.04 (2CH_2_–N), 66.08 (2CH_2_–O), 87.98 (C–N), 116.17–139.78 (Ar–C), 142.20, 151.17 (Ar–C–O), 156.31 (C–N), 160.10 (C=N–NH_2_), 163.29 (C=N), 166.14 (C=O), 167.31 (C=O, pyranone), 172.21 (C–CH_3_), 176.50 (C=O, imide). Anal. Calcd for C_27_H_25_N_5_O_7_ (531.53): C, 61.01; H, 4.74; N, 13.18%. Found: C, 61.03; H, 4.74; N, 13.20%.

**2-((2-Morpholino-2-oxoethyl)thio)acetic acid (5)**

Intermediate **B** (10 mmol, 1.63 g) was dissolved in an ethanol/water mixture (1:1, 30 mL). Thioglycolic acid (11 mmol, 0.84 mL) and potassium carbonate (15 mmol, 2.07 g) were subsequently added, and the reaction mixture was stirred at 50 °C for 6 h. The progress of the reaction was periodically checked by TLC using DCM/MeOH (9:1) as the eluent (Rf = 0.35). After completion, the reaction was allowed to cool to room temperature before being poured onto crushed ice. The resulting solid was isolated by filtration, washed thoroughly with water, and purified by recrystallization from ethanol to furnish compound **5** as an off-white solid (90% yield, m.p. 150–152 °C). The product was assigned as a thioether-functionalized morpholine derivative containing a carboxylic acid moiety. IR (KBr, ν max, cm^−1^): 3412 (OH), 2974, 2928, 2850 (aliphatic CH), 1750 (C=O, COOH), 1675 (C=O, amide). ^1^H NMR (400 MHz, DMSO-*d*_6_) δ (ppm): 2.92 (t, 4H, 2CH_2_–N), 3.22 (s, 2H, CH_2_–COOH), 3.50 (s, 2H, CH_2_–CO), 3.59 (t, 4H, 2CH_2_–O), 12.22 (br s, 1H, OH). ^13^C NMR (100 MHz, DMSO-*d*_6_) δ (ppm): 39.05 (CH_2_), 42.22 (CH_2_–COOH), 45.33 (2CH_2_–N), 65.09 (2CH_2_–O), 166.40 (C=O), 174.49 (COOH). Anal. Calcd for C_8_H_13_NO_4_S (219.26): C, 43.82; H, 5.98; N, 6.39%. Found: C, 43.80; H, 5.98; N, 6.39%.

**2-(2-Morpholino-2-oxoethyl)malononitrile (6)**

Compound **B** (10 mmol, 1.63 g) was dissolved in ethanol (30 mL), followed by the sequential addition of malononitrile (12 mmol, 0.79 g) and potassium carbonate (15 mmol, 2.07 g). The reaction mixture was refluxed at 78 °C for 6 h, and its progress was monitored by TLC using hexane/ethyl acetate (6:4) (Rf = 0.50). After completion, the mixture was cooled to ambient temperature and poured onto crushed ice. The resulting solid was collected by filtration, washed with water, and recrystallized from ethanol to give compound **6** as a pale-yellow solid (78% yield, m.p. 140–142 °C). The product was assigned as a dicyanomethyl-functionalized morpholine derivative. IR (KBr, ν max, cm^−1^): 2979, 2944, 2868 (aliphatic CH), 2190, 2164 (2CN), 1629 (C=O, amide). ^1^H NMR (400 MHz, DMSO-*d*_6_) δ (ppm): 2.81 (s, 2H, CH_2_), 2.90 (t, 4H, 2CH_2_–N), 3.59 (t, 4H, 2CH_2_–O), 4.10 (t, 1H, CH). ^13^C NMR (100 MHz, DMSO-*d*_6_) δ (ppm): 15.05 (CH), 28.28 (CH_2_), 45.09 (2CH_2_–N), 65.56 (2CH_2_–O), 112.44 (2CN), 171.49 (C=O). MS (EI): m/z = 193 (M^+^), 164 (base peak). Anal. Calcd for C_9_H_11_N_3_O_2_ (193.21): C, 55.95; H, 5.74; N, 21.75%. Found: C, 55.93; H, 5.76; N, 21.75%.

***2-((2-Morpholino-2-oxoethyl)thio)acetyl chloride* *(7)***

Compound **5** (10 mmol, 2.19 g) was suspended in thionyl chloride (20 mL) in a round-bottom flask and heated under reflux in an oil bath maintained at 80 °C for 6 h. The reaction progress was periodically followed by TLC using DCM/MeOH (9:1) as the eluent (Rf = 0.50). After completion of the reaction, the excess thionyl chloride was removed under reduced pressure to generate the corresponding acid chloride intermediate, which was used in the subsequent step without additional purification. The remaining residue was allowed to cool and then carefully poured onto crushed ice with continuous stirring. The resulting solid was isolated by filtration, washed with cold water, air-dried, and finally recrystallized from ethanol to furnish compound **7** as an off-white solid (85% yield, m.p. 120–122 °C). The isolated product was assigned as the acid chloride analogue of compound 5. IR (KBr, ν max, cm^−1^): 2964, 2926, 2852 (aliphatic CH), 1718 (C=O–Cl), 1653 (C=O, amide). ^1^H NMR (400 MHz, DMSO-*d*_6_) δ (ppm): 2.91 (t, 4H, 2CH_2_–N), 3.50 (s, 2H, CH_2_–CO), 3.59 (t, 4H, 2CH_2_–O), 3.99 (s, 2H, CH_2_–CCl). ^13^C NMR (100 MHz, DMSO-*d*_6_) δ (ppm): 39.05 (CH_2_), 45.35 (2CH_2_–N), 47.00 (CH_2_–CCl), 65.28 (2CH_2_–O), 166.12 (C=O), 173.00 (CO–Cl). MS (EI): m/z = 237 (M^+^), 164 (base peak). Anal. Calcd for C_8_H_12_ClNO_3_S (237.70): C, 40.42; H, 5.09; N, 5.89%. Found: C, 40.41; H, 5.09; N, 5.89%.

***(2-((2-Morpholino-2-oxoethyl)thio)acetyl)glycine (8)***

Compound **7** (10 mmol, 2.37 g) was dissolved in chloroform (30 mL). Glycine (11 mmol, 0.83 g) together with triethylamine (0.5 mL) was subsequently added, and the reaction mixture was maintained at **50 °C** with continuous stirring for 6 h. The reaction was monitored by TLC using DCM/MeOH (9:1) (Rf = 0.30). After cooling to room temperature, the mixture was poured onto crushed ice, and the resulting solid was isolated by filtration, washed with water, and recrystallized from ethanol to afford compound **8** as an off-white solid (71% yield, m.p. 173–175 °C). The product was identified as a glycine-conjugated amide bearing a terminal carboxyl group. IR (KBr, ν max, cm^−1^): 3403 (OH), 3186 (NH), 2976, 2937, 2857, 2739 (aliphatic CH), 1720 (C=O, COOH), 1703, 1655 (2C=O). ^1^H NMR (400 MHz, DMSO-*d*_6_) δ (ppm): 2.92 (t, 4H, 2CH_2_–N), 3.59 (t, 4H, 2CH_2_–O), 3.70 (s, 4H, 2CH_2_–CO), 4.30 (s, 2H, CH_2_–NH), 9.60 (br s, 1H, NH, exchangeable), 12.25 (br s, 1H, OH). ^13^C NMR (100 MHz, DMSO-*d*_6_) δ (ppm): 39.03 (CH_2_), 40.99 (CH_2_–CONCH_2_), 42.22 (CH_2_–COOH), 47.11 (2CH_2_–N), 66.22 (2CH_2_–O), 165.35 (C=O, morpholine), 170.03 (CO–NH), 175.05 (COOH). Anal. Calcd for C_10_H_16_N_2_O_5_S (276.31): C, 43.47; H, 5.84; N, 10.14%. Found: C, 43.49; H, 5.84; N, 10.12%.

***(Z)-2-(((2-Morpholino-2-oxoethyl)thio)methyl)-4-(thiophen-2-ylmethylene)oxazol-5(4H)-one (9)***

Compound **8** (11 mmol, 3.04 g) was dissolved in acetic anhydride (3 mL), after which thiophene-2-carboxaldehyde (10 mmol, 0.95 mL) and sodium acetate (12 mmol, 0.98 g) were introduced. The resulting mixture was refluxed at 120 °C for 2 h, while the reaction progress was periodically checked by TLC using DCM/MeOH (9:1) (Rf = 0.35). Upon completion, the mixture was allowed to cool to ambient temperature before ethanol (5 mL) was added. Standing at room temperature for 20 h resulted in gradual formation of a precipitate, which was isolated by filtration, washed with cold ethanol, and dried to give compound **9** as a yellow solid (78% yield, m.p. 200–202 °C). The product corresponds to a fused heterocyclic system incorporating a thiophene ring. IR (KBr, ν max, cm^−1^): 3020 (CH–thiophene), 2970, 2922, 2868 (aliphatic CH), 1696, 1660 (2C=O), 1582 (C=N), 1514 (C=C). ^1^H NMR (400 MHz, DMSO-*d*_6_) δ (ppm): 2.90 (t, 4H, 2CH_2_–N), 3.03 (s, 2H, CH_2_–oxazolone), 3.20 (s, 4H, 2CH_2_–CO), 3.67 (t, 4H, 2CH_2_–O), 7.37–7.45 (m, 4H, Ar–H). ^13^C NMR (100 MHz, DMSO-*d*_6_) δ (ppm): 35.03 (CH_2_–oxazolone), 39.00 (CH_2_–CO), 47.50 (2CH_2_–N), 66.22 (2CH_2_–O), 108.20 (CH, alkene–thiophene), 118.25–139.75 (Ar–C), 143.33 (C, alkene–oxazolone), 162.39 (C=O, oxazolone), 165.35 (C=O, morpholine), 168.03 (C=N). MS (EI): m/z = 352 (M^+^), 234 (base peak). Anal. Calcd for C_15_H_16_N_2_O_4_S_2_ (352.06): C, 51.12; H, 4.58; N, 7.95%. Found: C, 51.10; H, 4.58; N, 7.92%.

***(Z)-3-(((2-Morpholino-2-oxoethyl)thio)methyl)-5-(thiophen-2-ylmethylene)-2,5-dihydro-1,2,4-triazin-6(1H)-one (10)***

Compound **9** (10 mmol, 3.52 g) was dissolved in ethanol (30 mL), followed by sequential addition of hydrazine hydrate (12 mmol, 0.60 mL), sodium acetate (12 mmol, 0.98 g), and glacial acetic acid (2 mL). The reaction was maintained under reflux at 78 °C for 6 h with magnetic stirring, and its progress was periodically examined by TLC using DCM/MeOH (9:1) (Rf = 0.25). Once the reaction had reached completion, the mixture was cooled to room temperature and carefully poured onto crushed ice. The resulting solid was isolated by filtration, washed with water, and recrystallized from ethanol to give compound **10** as a pale-yellow solid (60% yield, m.p. 223–225 °C). The product represents a fused thiophene-containing triazine derivative. IR (KBr, ν max, cm^−1^): 3323, 3239 (2NH), 3006 (CH–thiophene), 2970, 2929, 2863 (aliphatic CH), 1713, 1662 (2C=O), 1582 (C=N), 1486 (C=C). ^1^H NMR (400 MHz, DMSO-*d*_6_) δ (ppm): 2.88 (t, 4H, 2CH_2_–N), 3.11 (s, 4H, 2CH_2_), 3.79 (t, 4H, 2CH_2_–O), 7.07–7.74 (m, 3H, Ar–H), 8.70 (d, 1H, Ar–H–S), 9.90, 11.77 (br s, 2H, 2NH, triazinone, exchangeable). ^13^C NMR (100 MHz, DMSO-*d*_6_) δ (ppm): 35.83 (CH_2_–triazinone), 39.12 (CH_2_–CO), 47.13 (2CH_2_–N), 66.03 (2CH_2_–O), 103.68 (CH, alkene–thiophene), 117.93–139.74 (Ar–C), 140.03 (C, triazinone), 152.60 (C=N), 162.29 (C=O, triazinone), 165.39 (C=O, morpholine). MS (EI): m/z = 366 (M^+^), 368 (M+2), 118 (base peak). Anal. Calcd for C_15_H_18_N_4_O_3_S_2_ (366.45): C, 49.16; H, 4.95; N, 15.29%. Found: C, 49.15; H, 4.98; N, 15.29%.

***4,6-Diamino-1,3-dimethyl-5-(2-morpholino-2-oxoethyl)tetrahydropyrimidin-2(1H)-one (11)***

Compound **6** (10 mmol, 1.93 g) was dissolved in ethanol (30 mL). N, N-Dimethylurea (11 mmol, 0.96 g) and potassium carbonate (15 mmol, 2.07 g) were subsequently introduced, and the mixture was refluxed at 78 °C for 6 h. The progress of the reaction was followed by TLC using DCM/MeOH (9:1) (Rf = 0.30). After cooling to room temperature, the reaction mixture was poured onto crushed ice, and the resulting solid was collected by filtration. The crude product was washed with water and recrystallized from ethanol to give compound **11** as an off-white solid (74% yield, m.p. 188–190 °C). IR (KBr, ν max, cm^−1^): 3397, 3378 (2NH_2_), 2976, 2920, 2886 (aliphatic CH), 1677 (C=O, pyrimidine), 1628 (C=O, amide). ^1^H NMR (400 MHz, DMSO-*d*_6_) δ (ppm): 2.20 (d, 2H, CH_2_), 2.88 (t, 4H, 2CH_2_–N), 3.10 (s, 6H, 2CH_3_), 3.30 (t, 1H, CH, pyrimidine), 3.58 (t, 4H, 2CH_2_–O), 5.70 (d, 2H, CH–NH_2_), 9.29 (br s, 4H, 2NH_2_, exchangeable). ^13^C NMR (100 MHz, DMSO-*d*_6_) δ (ppm): 17.99 (CH_2_–pyrimidine), 31.36 (2CH_3_), 42.72 (CH, pyrimidine), 45.75 (2CH_2_–N), 65.50 (2CH_2_–O), 75.05 (2CH–NH_2_), 156.42 (C=O, pyrimidine), 171.18 (C=O). Anal. Calcd for C_12_H_23_N_5_O_3_ (285.35): C, 50.51; H, 8.12; N, 24.54%. Found: C, 50.50; H, 8.15; N, 24.54%.

***4-Amino-6-((4-amino-2-fluoro-5-nitrophenyl)amino)-1,3-dimethyl-5-(2-morpholino-2-oxoethyl)tetrahydropyrimidin-2(1H)-one (12)***

Compound **11** (10 mmol, 2.85 g) was dissolved in chloroform (30 mL), after which 2-amino-4,5-difluoronitrobenzene (10 mmol, 1.76 g) and potassium carbonate (15 mmol, 2.07 g) were introduced. The resulting mixture was stirred at 50 °C for 6 h, and reaction progress was periodically checked by TLC using DCM/MeOH (9:1) as the eluent (Rf = 0.40). Once the reaction was complete, the mixture was cooled to ambient temperature and poured onto crushed ice. The resulting precipitate was isolated by filtration, washed with water, and recrystallized from ethanol to give compound **12** as a yellow solid (83% yield, m.p. 213–215 °C). IR (KBr, ν max, cm^−1^): 3495, 3479, 3365, 3314 (2NH_2_), 3255 (NH), 3034 (Ar–CH), 2980, 2930, 2830 (aliphatic CH), 1655 (C=O, pyrimidine), 1624 (C=O, amide), 1580 (C=C), 1534, 1350 (NO_2_). ^1^H NMR (400 MHz, DMSO-*d*_6_) δ (ppm): 2.20 (d, 2H, CH_2_), 2.90 (t, 4H, 2CH_2_–N), 3.15 (s, 6H, 2CH_3_), 3.64 (t, 1H, CH, pyrimidine), 3.66 (t, 4H, 2CH_2_–O), 5.66 (d, 2H, CH–NH_2_), 5.96 (br s, 1H, NH_2_, exchangeable), 7.06–7.07 (s, 2H, Ar–H), 7.12, 9.25 (br s, 4H, 2NH_2_, exchangeable). ^13^C NMR (100 MHz, DMSO-*d*_6_) δ (ppm): 18.12 (CH_2_–pyrimidine), 30.94 (2CH_3_), 41.94 (CH–CH_2_), 45.12 (2CH_2_–N), 66.20 (2CH_2_–O), 75.17 (CH–NH_2_), 85.12 (CH–NH), 113.95–131.00 (Ar–C), 156.35 (C=O, pyrimidine), 164.69 (Ar–C–F), 171.08 (C=O). MS (EI): m/z = 435 (M–4), 147 (base peak). Anal. Calcd for C_18_H_26_FN_7_O_5_ (439.45): C, 49.20; H, 5.96; N, 22.31%. Found: C, 49.23; H, 5.96; N, 22.33%.

### 2.2. Biological Evaluation

Only a concise description of each experimental procedure is provided in the main manuscript, while the complete experimental details are available in the Supplementary Materials.

#### 2.2.1. In Vitro Cytotoxicity and Selectivity Assessment

The cytotoxic potential of the synthesized derivatives toward MCF-7 [56] and HCT-116 [57] cells was determined using the MTT colorimetric assay [58]. Human WI-38 fibroblasts [59] were included as the normal cell model to evaluate cellular selectivity.

#### 2.2.2. Multitarget Egfr/pi3k/mtor Kinase Inhibition

The inhibitory activities of compounds **3**, **10**, and **12** toward EGFR [60], PI3Kα [61], and mTOR [62] were determined using commercially available enzyme assay kits following the manufacturers’ recommendations. Kinase inhibition was measured by luminescence- or TR-FRET-based detection methods. Erlotinib [63], pictilisib [64], and rapamycin [65] served as the reference inhibitors for EGFR, PI3Kα, and mTOR, respectively.

#### 2.2.3. Cell Cycle Modulation and Apoptosis Induced by Compound **10**

The influence of compound **10** on cell-cycle progression [66] and apoptotic cell death [67,68] was examined in MCF-7 and HCT-116 cells by flow cytometry. Cell-cycle distribution was analyzed using propidium iodide (PI) staining, whereas apoptosis was assessed using the Annexin V-FITC/PI dual-staining method.

#### 2.2.4. Effect of Compound 10 on Mitochondrial Membrane Potential (Δψm) in Hct-116 Cells

Changes in the mitochondrial membrane potential (ΔΨm) of HCT-116 cells following treatment with compound **10** were determined using the MitoProbe™ DiOC2(3) flow cytometric assay in accordance with the manufacturer’s instructions [69,70,71].

#### 2.2.5. Effect of Compound **10** on Intracellular Reactive Oxygen Species (Ros) Generation in Hct-116 Cells

Intracellular ROS levels were quantified in HCT-116 cells exposed to compound **10** using the DCFDA Cellular ROS Assay Kit (Abcam, Cambridge, UK; Cat. No. ab113851) followed by flow cytometric detection. Fluorescence signals were recorded in the FITC channel and expressed relative to untreated control cells [72,73,74].

#### 2.2.6. Modulation of Apoptosis-Related Gene Expression by Compound **10**

The transcriptional response of the apoptosis-associated genes caspase-3, caspase-9, Bax, and Bcl-2 was investigated in HCT-116 cells treated with compound **10** by quantitative RT-PCR using the iScript™ One-Step RT-PCR Kit with SYBR^®^ Green (Bio-Rad, Hercules, CA, USA). Relative gene expression was normalized against GAPDH and calculated using the 2^−ΔΔCt^ method [75,76,77].

#### 2.2.7. In Vitro Antimicrobial Activity

The antibacterial properties of the selected morpholine derivatives (**12**, **5**, **3**, **10**, **8**, and **6**) were examined using the agar-well diffusion technique against *Enterococcus faecalis* ATCC 29212, *Staphylococcus aureus* ATCC 25923, *Escherichia coli* ATCC 25915, and *Pseudomonas aeruginosa* ATCC 10145, following the previously reported procedure [78]. Ciprofloxacin (20 μg/mL) was included as the positive reference drug.

#### 2.2.8. Determination of Minimum Inhibitory Concentrations (Mics)

The minimum inhibitory concentrations (MICs) of the most active derivatives (**12**, **3**, and **10**) were established by the broth microdilution method in accordance with the Clinical and Laboratory Standards Institute (CLSI) recommendations [79]. Serial concentrations ranging from 5 to 160 μg/mL were prepared in nutrient broth, inoculated with the corresponding bacterial strains, and incubated at 37 °C for 24 h. The MIC value was recorded as the lowest concentration at which no visible bacterial growth was observed.

#### 2.2.9. Evaluation of Antibiofilm Activity

The antibiofilm potential of the most active derivatives (**12**, **3**, and **10**) was investigated using the crystal violet microplate assay according to the previously published protocol [79]. Bacterial suspensions were exposed to the tested compounds at their respective MIC values and incubated at 37 °C for 24 h. Biofilm biomass was subsequently quantified by crystal violet staining, and absorbance was recorded at 590 nm.

#### 2.2.10. DPPH and ABTS Radical Scavenging Activity

The antioxidant capacity of the synthesized derivatives was determined using DPPH and ABTS free-radical scavenging assays as previously reported [80]. Radical scavenging was quantified spectrophotometrically by measuring absorbance at 517 nm for DPPH and 734 nm for ABTS. The antioxidant activity of each compound was expressed as both percentage inhibition and IC_50_ values, with BHT employed as the reference antioxidant.

### 2.3. Computational Studies

#### 2.3.1. Computational Admet and Drug-Likeness Assessment

In silico evaluation of the pharmacokinetic profile, physicochemical characteristics, and drug-likeness of compounds **3**, **10**, and **12** was carried out using the SwissADME and admetSAR prediction platforms [81,82,83,84,85].

#### 2.3.2. Molecular Docking Simulation

The interaction of the most active morpholine derivatives (**3**, **10**, and **12**) with EGFR (1M17), PI3K (3L54), mTOR (4JT6), and *S. aureus* DNA gyrase (2XCT) was explored through molecular docking simulations. Protein coordinates were downloaded from the Protein Data Bank [86,87,88,89], whereas ligand structures were generated in ChemDraw 24.0, converted to optimized three-dimensional geometries, and minimized using the MMFF94x force field. Docking calculations were carried out with MOE 2024.0601 (Chemical Computing Group, Montreal, Canada) [90,91,92]. Prior to docking, protein structures were prepared using the Protonate 3D protocol. Ligand placement was performed using the Triangle Matcher algorithm, followed by ranking with the London dG scoring function. The docking procedure was validated by re-docking the native co-crystallized ligands into their original binding sites before analyzing the synthesized derivatives.

#### 2.3.3. Molecular Dynamics Simulations

To assess the dynamic stability of the selected protein–ligand complexes, 50 ns molecular dynamics simulations were performed using GROMACS 2018 with the CHARMM36 force field. Ligand parameters were assigned using the CGenFF server, and all systems were equilibrated under NVT followed by NPT conditions prior to the production phase. The resulting trajectories were analyzed by monitoring RMSD, RMSF, and radius of gyration (Rg) as indicators of conformational stability and structural flexibility [93].

#### 2.3.4. Quantum Chemical Calculations

Quantum chemical calculations were carried out within the framework of density functional theory (DFT) using ORCA 6 with the B3LYP exchange-correlation functional and the 6-311G++(d,p) basis set. The optimized electronic structures were used to obtain frontier molecular orbital (FMO) energies and the related global reactivity descriptors. Additional electronic structure analyses, including density of states (DOS), electrostatic potential (ESP), and noncovalent interaction (NCI) mapping, were performed to provide further insight into the electronic characteristics of the synthesized derivatives [94,95,96,97,98].

## 3. Results and Discussion

### 3.1. Chemistry

**Scheme 1:**
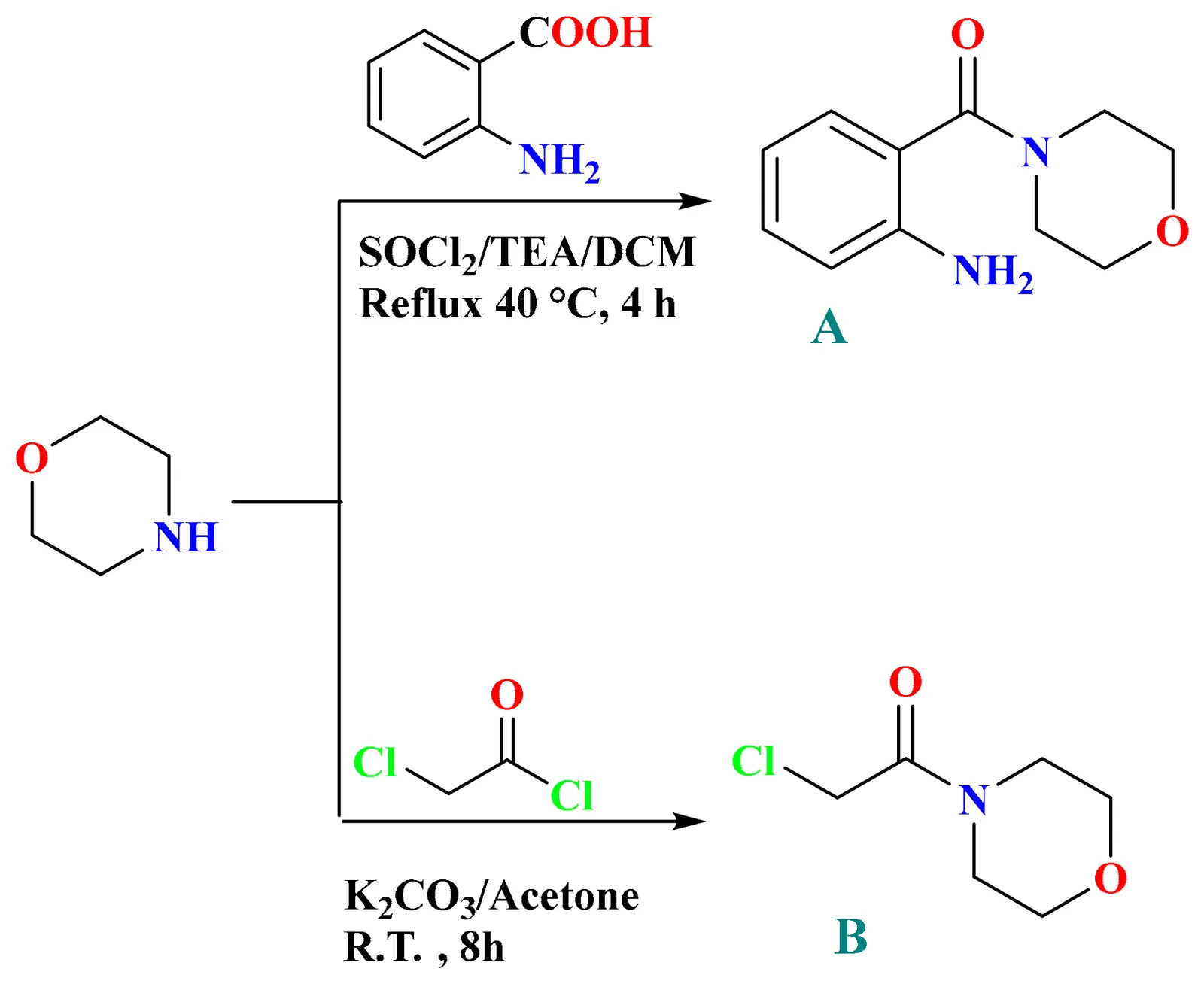
Divergent synthesis of intermediates **A** and **B** via N-acylation of morpholine.

Compounds **A** and **B** were synthesized by N-acylation of morpholine via nucleophilic acyl substitution using two complementary synthetic approaches (Scheme 1). In the synthesis of compound **A**, anthranilic acid was first activated in situ with thionyl chloride to generate the corresponding acyl chloride, thereby increasing the electrophilicity of the carbonyl carbon. The secondary amino group of morpholine subsequently attacked the activated carbonyl center, followed by elimination of chloride to afford the desired amide while preserving the ortho-amino substituent. The preferential N-acylation of morpholine over the aromatic amino group can be attributed to the higher nucleophilicity of the cyclic secondary amine together with the lower reactivity of the aniline amino group due to resonance delocalization into the aromatic ring. Similarly, compound **B** was obtained through direct acylation of morpholine with 2-chloroacetyl chloride in the presence of K_2_CO_3_, which acts both as an acid scavenger and as a base to neutralize the generated hydrochloric acid, thereby facilitating amide bond formation. Importantly, the reaction selectively occurred at the acyl chloride carbonyl rather than the chloromethylene carbon because the carbonyl carbon is considerably more electrophilic. Consequently, the reactive –CH_2_Cl functionality remained intact, providing a versatile synthetic handle for subsequent nucleophilic substitution reactions. The mild reaction conditions, straightforward work-up, and preservation of the functional groups demonstrate the efficiency of the developed synthetic strategy and furnish two complementary intermediates suitable for further structural diversification. Thus, both compounds constitute key intermediates for subsequent derivatization. The structures of compounds **A** and **B** were confirmed by IR and NMR spectroscopy. Compound **A** exhibited characteristic NH_2_ absorption bands at 3427 and 3388 cm^−1^, which were absent in compound **B**, while both compounds showed the expected amide C=O bands. In the ^1^H NMR spectra, both compounds displayed the characteristic morpholine resonances, whereas compound **A** showed an NH_2_ singlet at δ 5.05 ppm and aromatic proton signals, while compound **B** exhibited the diagnostic CH_2_Cl singlet at δ 4.53 ppm. The ^13^C NMR spectra further confirmed the proposed structures through the expected carbonyl and CH_2_Cl carbon resonances.

**Scheme 2:**
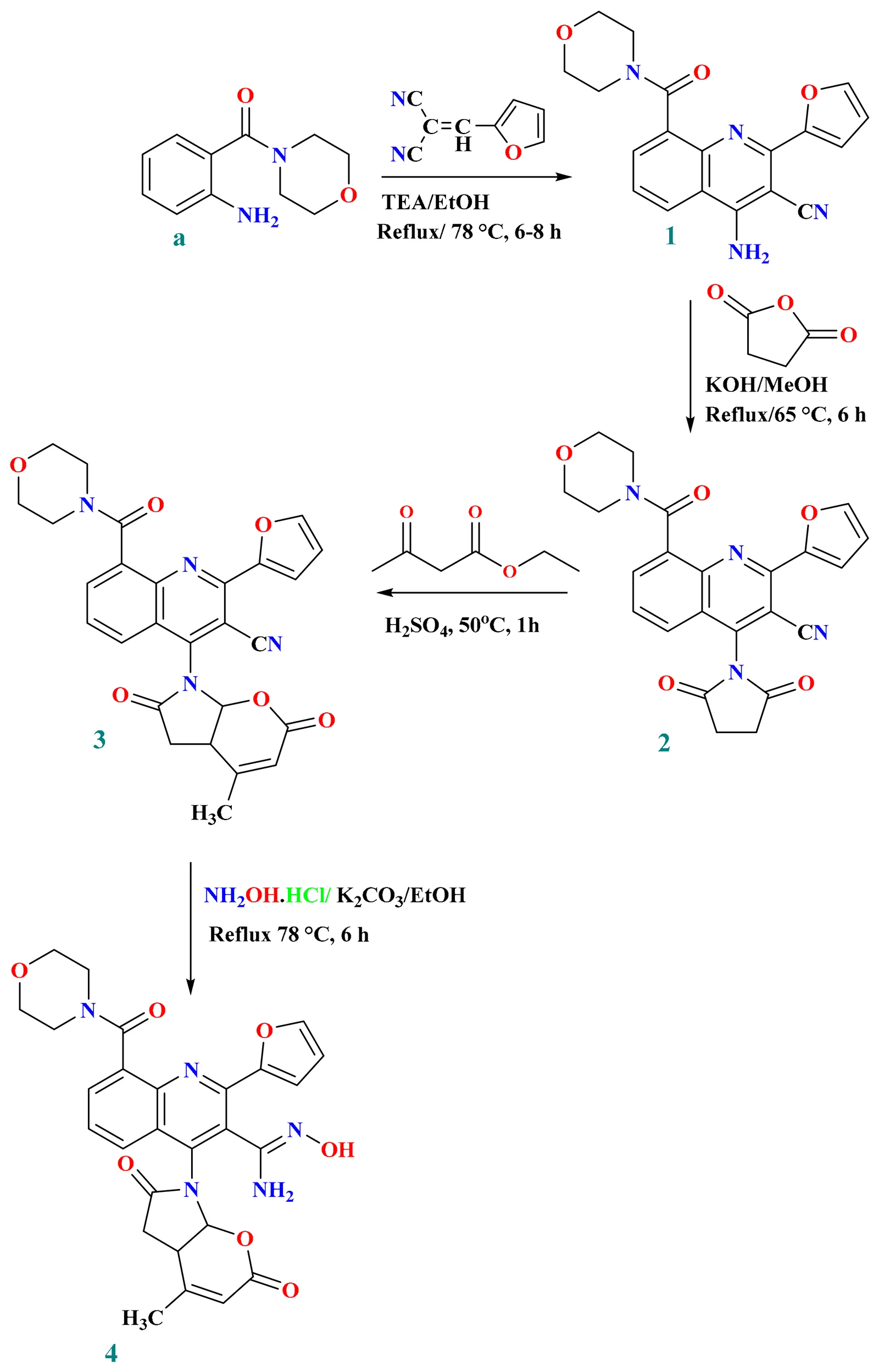
Synthesis of fused heterocyclic derivatives **1**–**4** via pyridine formation, succinimide cyclization, pyranone construction, and amidoxime functionalization.

The synthetic pathway commenced from compound **A**, which reacted with the arylidene malononitrile derivative under basic conditions to afford compound 1 through an initial Michael addition of the exocyclic amino group to the activated α,β-unsaturated system, followed by intramolecular cyclization to generate the fused pyridine ring (Scheme 2). The reaction preferentially proceeded through the exocyclic amino group because of its higher nucleophilicity, while the electron-withdrawing cyano groups activated the double bond toward nucleophilic attack and facilitated efficient ring closure. Compound **1** was subsequently acylated with succinic anhydride via nucleophilic acyl substitution to furnish the succinimide derivative **2**, with the anhydride carbonyl serving as the preferred electrophilic center. Under acidic conditions, compound **2** underwent condensation with ethyl acetoacetate followed by cyclization to afford the fused pyranone derivative **3**. Finally, treatment of **3** with hydroxylamine hydrochloride selectively converted the cyano group into the corresponding amidoxime derivative **4** without further cyclization, reflecting the good chemoselectivity and functional group tolerance of the developed synthetic strategy. The formation of compound **1** was confirmed by the characteristic IR absorptions at 2191 cm^−1^ (CN) and 1617 cm^−1^ (C=N), together with the downfield NH_2_ resonance at δ 6.02 ppm and the expected carbon resonances for the nitrile and imine carbons. Cyclization to compound **2** was evidenced by disappearance of the NH_2_ signal and the appearance of two imide carbonyl absorptions at 1682 and 1658 cm^−1^, in addition to a new CH_2_ resonance at δ 2.46 ppm and a carbonyl carbon signal at δ 176.03 ppm, while the CN and C=N functionalities remained unchanged. The successful formation of compound **3** was confirmed by the appearance of the pyranone carbonyl absorption at 1633 cm^−1^, new CH_3_ and methine resonances at δ 1.81, 5.39, and 3.15 ppm, respectively, together with the expected carbonyl carbon signals and the molecular ion peak (M^+^ = 498). Subsequent conversion of **3** into **4** was readily verified by disappearance of the CN absorption and the emergence of broad OH and NH_2_ bands, accompanied by new NH_2_ and OH resonances at δ 6.86 and 9.44 ppm. The disappearance of the nitrile carbon signal and appearance of the characteristic C=N–NH_2_ carbon resonance further confirmed the proposed amidoxime structure.

**Scheme 3:**
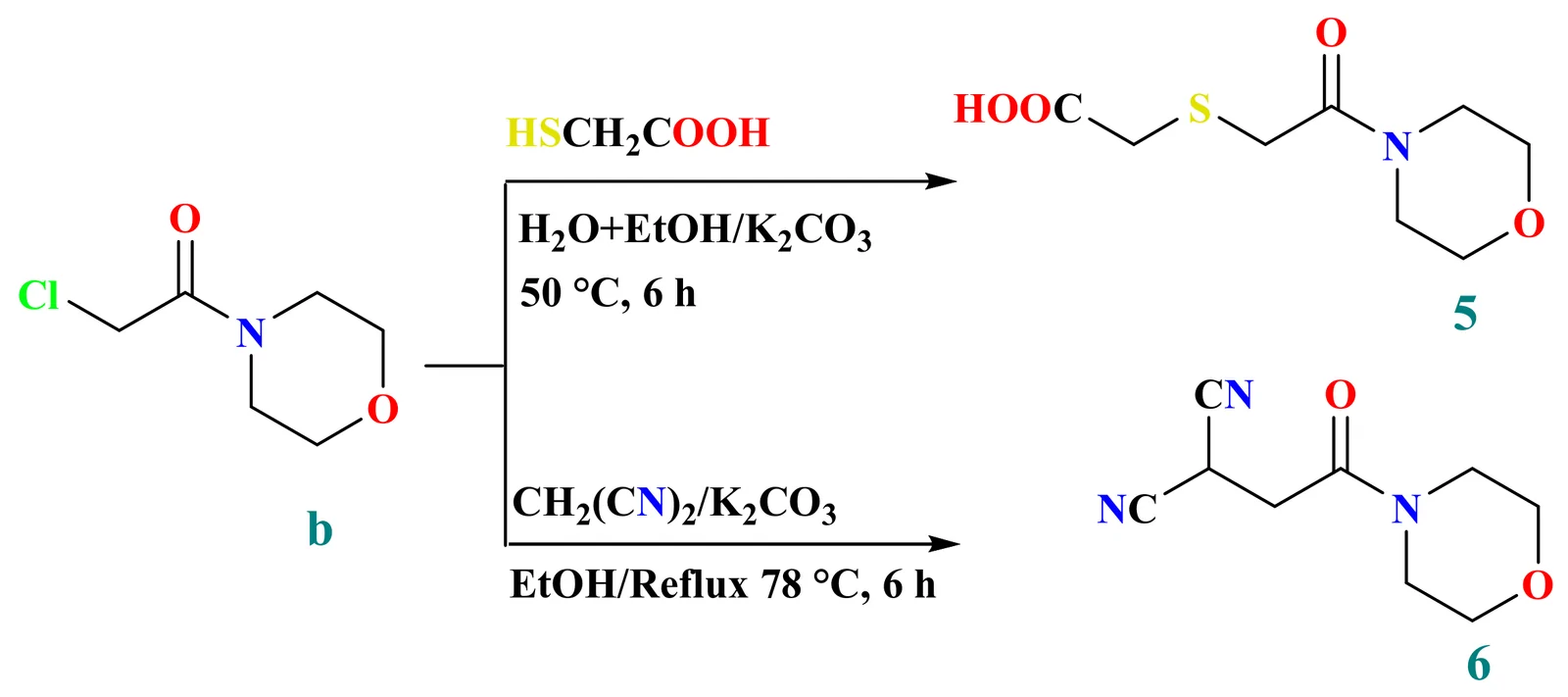
Synthesis of compounds **5** and **6** from compound **B** via divergent nucleophilic substitution.

The synthetic versatility of compound **B** was explored through nucleophilic substitution at the activated α-chloro center (Scheme 3). Treatment with thioglycolic acid in the presence of K_2_CO_3_ furnished compound **5** via an S_N2 substitution mechanism, in which the thiolate anion attacked the electrophilic methylene carbon, displacing the chloride ion and affording the corresponding thioether-linked carboxylic acid. The reaction proceeded with high chemoselectivity without affecting the amide functionality or promoting intramolecular cyclization under the applied conditions. Similarly, reaction of **B** with malononitrile generated compound **6** through S_N2 displacement of chloride by the resonance-stabilized malononitrile carbanion, introducing a highly activated dicyanomethyl functionality. The incorporation of this electron-deficient fragment provides a valuable intermediate for subsequent cyclization reactions, demonstrating the versatility of compound **B** as a convenient synthetic precursor for further structural elaboration. The structure of **5** was confirmed by the appearance of characteristic absorptions at 1750 cm^−1^ (C=O) and 3412 cm^−1^ (OH), together with an OH resonance at δ 12.22 ppm. Additional signals at δ 3.22 ppm assigned to the CH_2_–COOH group, along with carbon resonances at δ 42.22 ppm (CH_2_) and 174.49 ppm (COOH), verified the successful incorporation of the carboxylic acid functionality. Successful formation of **6** was demonstrated by the presence of two nitrile stretching bands at 2190 and 2164 cm^−1^, confirming introduction of the dicyanomethyl group [–CH(CN)_2_]. Compared with compound B, where the CH_2_Cl group resonated at δ 4.53 ppm, the product exhibited new resonances at δ 2.81 ppm (CH_2_) and 4.10 ppm (CH), consistent with the formation of the CH_2_–CH(CN)_2_ fragment. This assignment was further reinforced by carbon signals at δ 15.05 ppm for the methine carbon and δ 112.44 ppm for the two nitrile carbons, while the molecular ion peak at *m/z* = 193 (M^+^) provided additional evidence supporting the proposed structure.

**Scheme 4:**
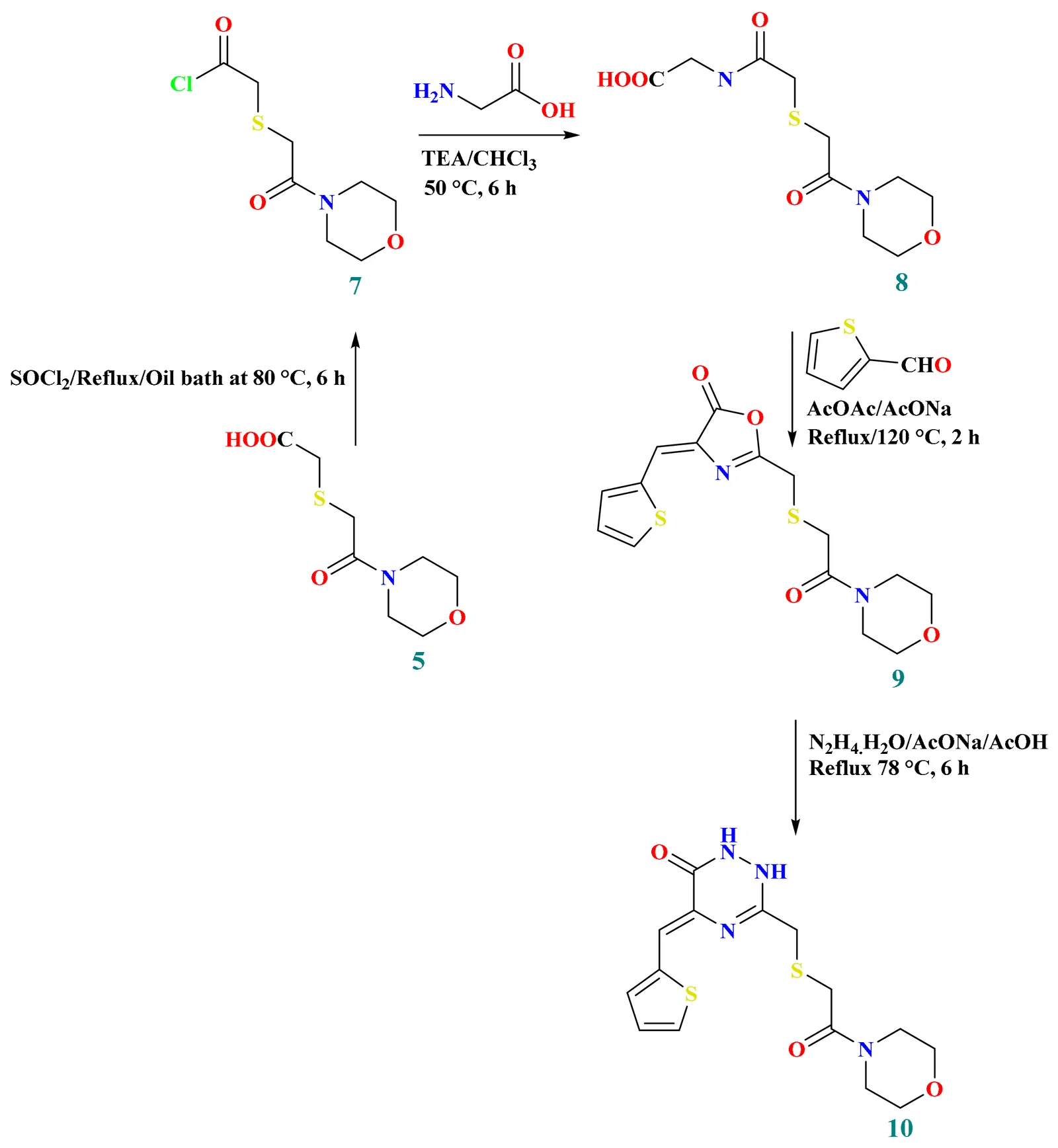
Synthesis of compounds **7**–**10** via thioether formation, acyl chloride activation, amide coupling, oxazolone cyclization, and triazinone formation.

Compounds **7**–**10** were synthesized from compound **5** through sequential activation, substitution, condensation, and cyclization reactions (Scheme 4). Initially, treatment of compound **5** with SOCl_2_ converted the carboxylic acid into the corresponding acid chloride **7**, thereby increasing the electrophilicity of the carbonyl carbon and facilitating subsequent amidation. Reaction of **7** with glycine afforded the amide derivative 8 through nucleophilic acyl substitution, while preserving the terminal carboxylic acid functionality for further derivatization. Condensation of compound **8** with thiophene-2-carboxaldehyde under AcONa/AcOH conditions furnished the conjugated oxazolone derivative **9** through cyclodehydration, with the extended conjugated system favoring ring formation and product stabilization. Finally, treatment of compound **9** with hydrazine hydrate resulted in nucleophilic ring transformation to afford the triazinone derivative **10**. The overall sequence proceeded with good chemoselectivity, allowing selective functional group transformations while preserving the integrity of the morpholine and thioether moieties, thus providing an efficient synthetic route toward the targeted triazinone scaffold. Conversion of **5** to **7** was confirmed by replacement of the carboxylic acid with an acid chloride, as evidenced by the shift in the carbonyl absorption peak from 1750 to 1718 cm^−1^ and disappearance of the OH band. The corresponding changes in the ^1^H and ^13^C NMR spectra, including the downfield shifts in the adjacent methylene and carbonyl signals, further supported the proposed structure. Formation of compound **8** was verified by the disappearance of the acid chloride absorption and the appearance of characteristic carboxylic acid, amide, NH, and OH bands, together with new NH and OH resonances in the ^1^H NMR spectrum and the expected carbonyl carbon signals in the ^13^C NMR spectrum. The conversion of **8** into **9** was confirmed by the loss of the NH and OH absorptions, the appearance of new carbonyl and C=N bands, characteristic thiophene proton signals, and the molecular ion peak at *m*/*z* = 352 (M^+^). Finally, successful cyclization to compound **10** was verified by the characteristic carbonyl and NH absorptions, the appearance of NH resonances in the ^1^H NMR spectrum, the expected triazinone carbon signals in the ^13^C NMR spectrum, and molecular ion peaks at m/z 366 and 368, confirming formation of the target triazinone derivative.

**Scheme 5:**
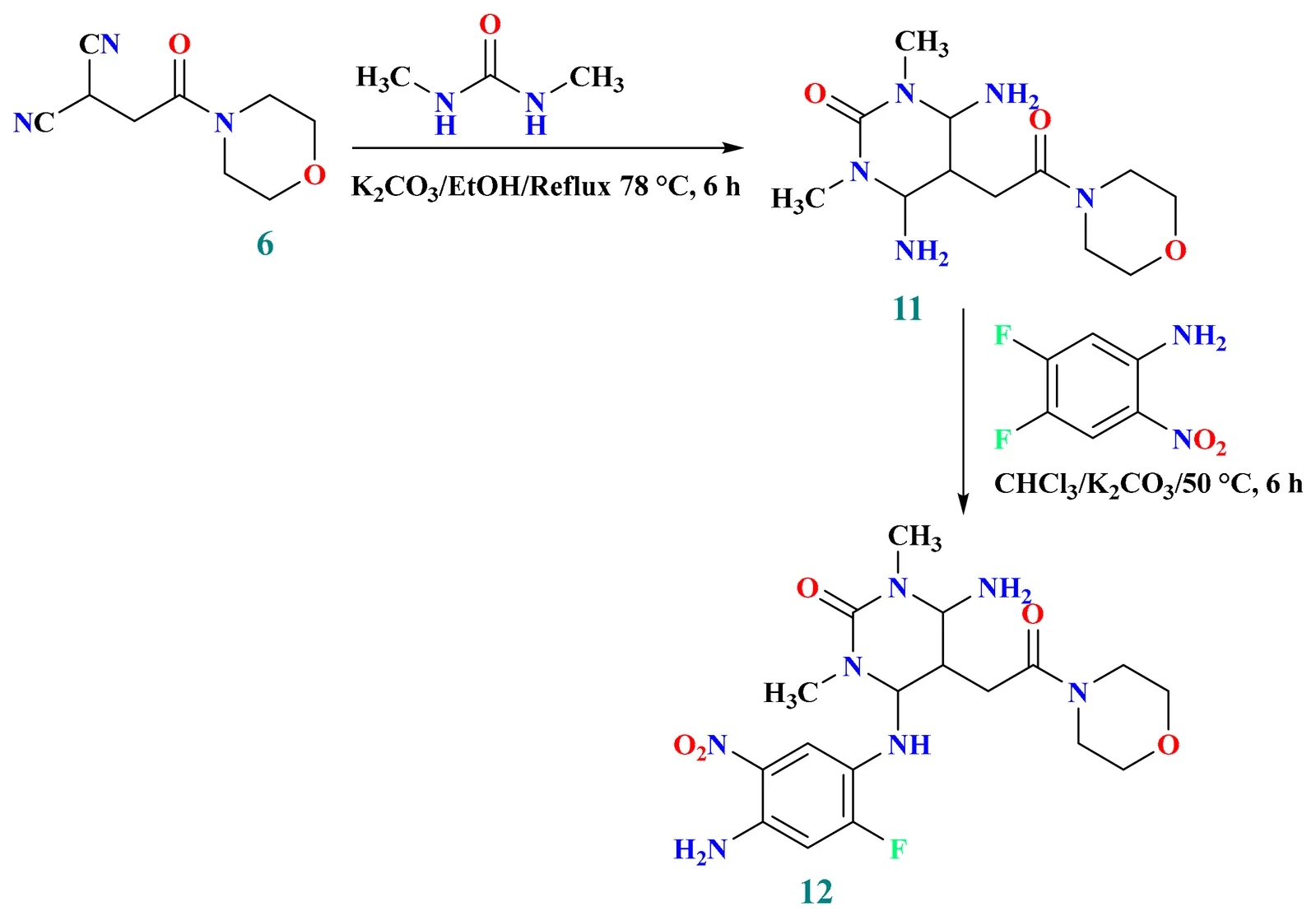
Conversion of compound **6** into pyrimidinone derivative **11** and its subsequent arylation to give compound **12**.

Compound **11** was synthesized from compound **6** by reaction with dimethylurea under basic reflux conditions through an initial nucleophilic addition to one of the cyano groups, followed by intramolecular cyclization to construct the pyrimidinone ring (Scheme 5). The presence of the highly electrophilic dicyanomethyl functionality in compound **6** facilitated nucleophilic attack and promoted efficient ring closure, while the second cyano group remained unaffected under the reaction conditions, demonstrating good chemoselectivity. Subsequently, compound **11** underwent nucleophilic substitution with the appropriate substituted nitroaniline in the presence of K_2_CO_3_ to afford compound **12** through selective C–N bond formation. The reaction proceeded without further cyclization or disruption of the pyrimidinone nucleus, preserving the integrity of the heterocyclic framework and providing an efficient route for introducing the substituted aniline pharmacophore. The formation of compound **11** was confirmed by the disappearance of the nitrile absorptions and the appearance of characteristic carbonyl and NH_2_ bands, indicating successful pyrimidinone ring formation. Consistent with this transformation, the ^1^H NMR spectrum showed the disappearance of the CH_2_–CH(CN)_2_ signals together with the appearance of new resonances assigned to the pyrimidine CH, CH–NH_2_, NH_2_, and dimethylurea methyl groups. The ^13^C NMR spectrum further supported cyclization through the loss of the nitrile carbon signal and the emergence of the characteristic pyrimidinone carbonyl and ring carbon resonances. Conversion of **11** into **12** was verified by incorporation of the nitrofluoroaryl moiety, as evidenced by the appearance of characteristic NH_2_, NH, aromatic C–H, and NO_2_ absorptions in the IR spectrum. These changes were further confirmed by the expected exchangeable proton and aromatic signals in the ^1^H NMR spectrum together with the corresponding aromatic and pyrimidinone carbon resonances in the ^13^C NMR spectrum. Finally, the molecular ion peak at *m/z* = 435 (M–4), instead of the calculated *m/z* = 439, was attributed to hydrogen loss during ionization, providing additional support for the proposed structure.

Overall, the developed synthetic strategy provided efficient access to structurally diverse morpholine-based nitrogen-rich heterocyclic derivatives through straightforward reaction sequences, good chemoselectivity, and tolerance toward multiple functional groups. Nevertheless, some transformations required prolonged reflux conditions or multistep sequences, which may represent limitations for large-scale synthesis and warrant further optimization in future studies.

### 3.2. Biological Evaluation

#### 3.2.1. In Vitro Cytotoxicity and Selectivity Assessment

To investigate the antiproliferative potential of the synthesized morpholine derivatives (**1**–**12**), their cytotoxic activities were evaluated against human colorectal (HCT-116) and breast (MCF-7) cancer cell lines, while cytotoxicity toward normal WI-38 fibroblasts was assessed in parallel to determine cellular selectivity. Erlotinib was used as the reference drug (Table 1). The results clearly demonstrated that both the nature of the incorporated heterocyclic scaffold and the attached pharmacophoric fragments markedly influenced the antiproliferative activity and cellular selectivity of the synthesized compounds. Among the investigated series, compounds **10**, **12**, and **3** emerged as the most promising derivatives. Compound **10** exhibited potent cytotoxic activity against HCT-116 and MCF-7 cells (IC_50_ = 5.12 ± 0.5 and 6.26 ± 0.3 μM, respectively), with activity comparable to that of erlotinib, indicating that the sulfur-linked triazinone framework efficiently enhances antiproliferative activity. Interestingly, compound **12** displayed comparable cytotoxic potency (IC_50_ = 5.06 ± 0.3 and 6.14 ± 0.2 μM, respectively), while exhibiting the highest selectivity indices toward both cancer cell lines, suggesting that incorporation of the pyrimidinone scaffold together with the substituted aniline moiety contributes more significantly to selective cancer cell inhibition than to increasing intrinsic cytotoxic potency. Likewise, compound **3** exhibited an excellent balance between potency and safety, supporting the beneficial contribution of the fused pyran scaffold to the antiproliferative profile. In contrast, compounds lacking these optimized structural features generally displayed lower cytotoxic activity and/or reduced selectivity, highlighting the importance of appropriate pharmacophore integration for achieving enhanced biological performance. Although the observed selectivity toward WI-38 cells is encouraging, the present evaluation was limited to a single normal cell line. Therefore, additional studies using tissue-relevant non-malignant breast and colorectal cell models will be valuable to further validate the therapeutic selectivity and safety profile of the most active compounds.

##### Anticancer Structure-Activity Relationship (SAR)

The antiproliferative data summarized in Table 1 clearly demonstrate that both the heterocyclic framework and the nature of the incorporated substituents govern the cytotoxic potency and cellular selectivity of the synthesized derivatives. Within the pyran-containing series, construction of the fused pyranopyridine scaffold significantly enhanced biological activity. While compound **1** exhibited only moderate antiproliferative activity, introduction of the cyclic imide ring in compound **2** proved unfavorable, most likely because of increased steric constraints and reduced molecular flexibility. In contrast, annulation to generate the rigid fused pyranopyrrole system in compound **3** markedly improved cytotoxic potency, suggesting that the polycyclic architecture together with the additional carbonyl functionalities provides a more favorable structural arrangement for interaction with the biological target. However, further conversion into the more polar amidoxime derivative **4** reduced activity, indicating that excessive polarity may compromise cellular permeability. Within the sulfur-linked series, the presence of the thioacetic acid side chain in compound **5** contributed to moderate antiproliferative activity, whereas introduction of the dicyanomethyl moiety in compound **6** did not further improve potency. Transformation into the α-chloro intermediate **7** was detrimental to activity, while incorporation of the glycine spacer in compound **8** partially restored biological activity, particularly against MCF-7 cells, emphasizing the favorable contribution of the amide linker. Interestingly, cyclization of compound **8** into the oxazolone derivative **9** failed to improve activity, suggesting that rigidification alone is insufficient for enhanced cytotoxicity. Conversely, construction of the triazinone nucleus in compound **10** resulted in a remarkable increase in antiproliferative activity, highlighting the beneficial role of nitrogen-rich heterocyclic systems in optimizing molecular recognition through complementary hydrogen-bonding and electronic interactions. A similar trend was observed in the pyrimidinone series. Although compound **11** displayed limited activity, further introduction of the substituted fluoro-nitroaniline fragment in compound **12** dramatically enhanced both potency and cellular selectivity. This improvement may arise from a synergistic combination of the pyrimidinone scaffold with appropriately balanced electronic and lipophilic substituents, thereby promoting more favorable interactions with the biological target while maintaining reduced toxicity toward normal cells.

**Figure 5:**
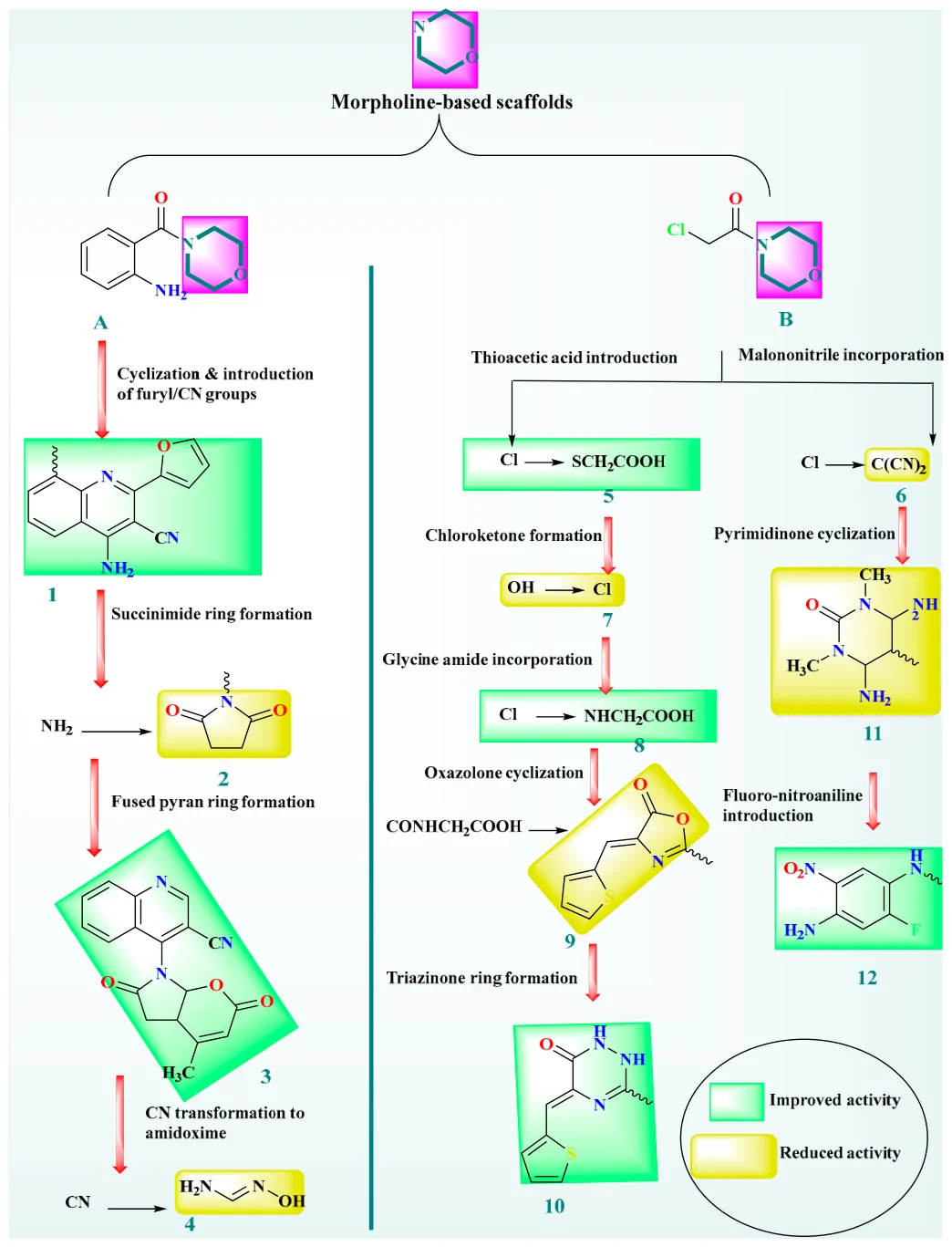
Schematic representation of the structure–activity relationship (SAR) observed for the synthesized morpholine-derived heterocyclic compounds, correlating structural modifications with their antiproliferative activities against HCT-116 and MCF-7 cancer cell lines.

Collectively, these findings identify compounds **3**, **10**, and **12** as the lead derivatives of the present series and demonstrate that the integration of rigid nitrogen-rich heterocyclic frameworks with carefully optimized peripheral substituents is essential for achieving potent and selective antiproliferative activity (Figure 5).

#### 3.2.2. Multitarget Egfr/pi3k/mtor Kinase Inhibition

To gain further insight into the molecular basis of the observed antiproliferative activity, the three most active derivatives (**3**, **10**, and **12**) were selected for enzymatic evaluation against EGFR, PI3K, and mTOR (Table 2). All three compounds inhibited the investigated kinases, although with different levels of potency. Among them, compound 10 exhibited the strongest multitarget inhibitory activity, with IC_50_ values of 0.086 ± 0.003, 0.107 ± 0.005, and 0.223 ± 0.008 μM against EGFR, PI3K, and mTOR, respectively, approaching the potency of the corresponding reference inhibitors. This superior activity can be attributed to the cooperative contribution of the morpholine nucleus and the sulfur-containing triazinone scaffold, which provide complementary hydrogen-bonding and hydrophobic interactions within the kinase active sites. Compound **3** also displayed pronounced kinase inhibition, confirming that the fused pyran-containing framework represents another favorable structural motif for kinase targeting. Interestingly, although compound **12** exhibited comparatively weaker enzymatic inhibition, it retained excellent antiproliferative activity and the highest cellular selectivity. This apparent discrepancy suggests that its biological performance cannot be explained solely by direct EGFR/PI3K/mTOR inhibition and may additionally reflect more favorable cellular uptake, intracellular accumulation, reduced susceptibility to efflux mechanisms, or interactions with complementary molecular targets. Collectively, the enzymatic and cellular findings strongly support the involvement of the EGFR/PI3K/mTOR signaling pathway in the antiproliferative activity of compound **10**. However, direct validation of downstream pathway modulation at the cellular level, such as the phosphorylation status of EGFR, AKT, and mTOR, was beyond the scope of the present study. Therefore, further protein-level investigations are warranted to provide additional confirmation of the proposed cellular mechanism of action.

#### 3.2.3. Cell Cycle Modulation and Apoptosis Induced by Compound **10**

**Figure 6:**
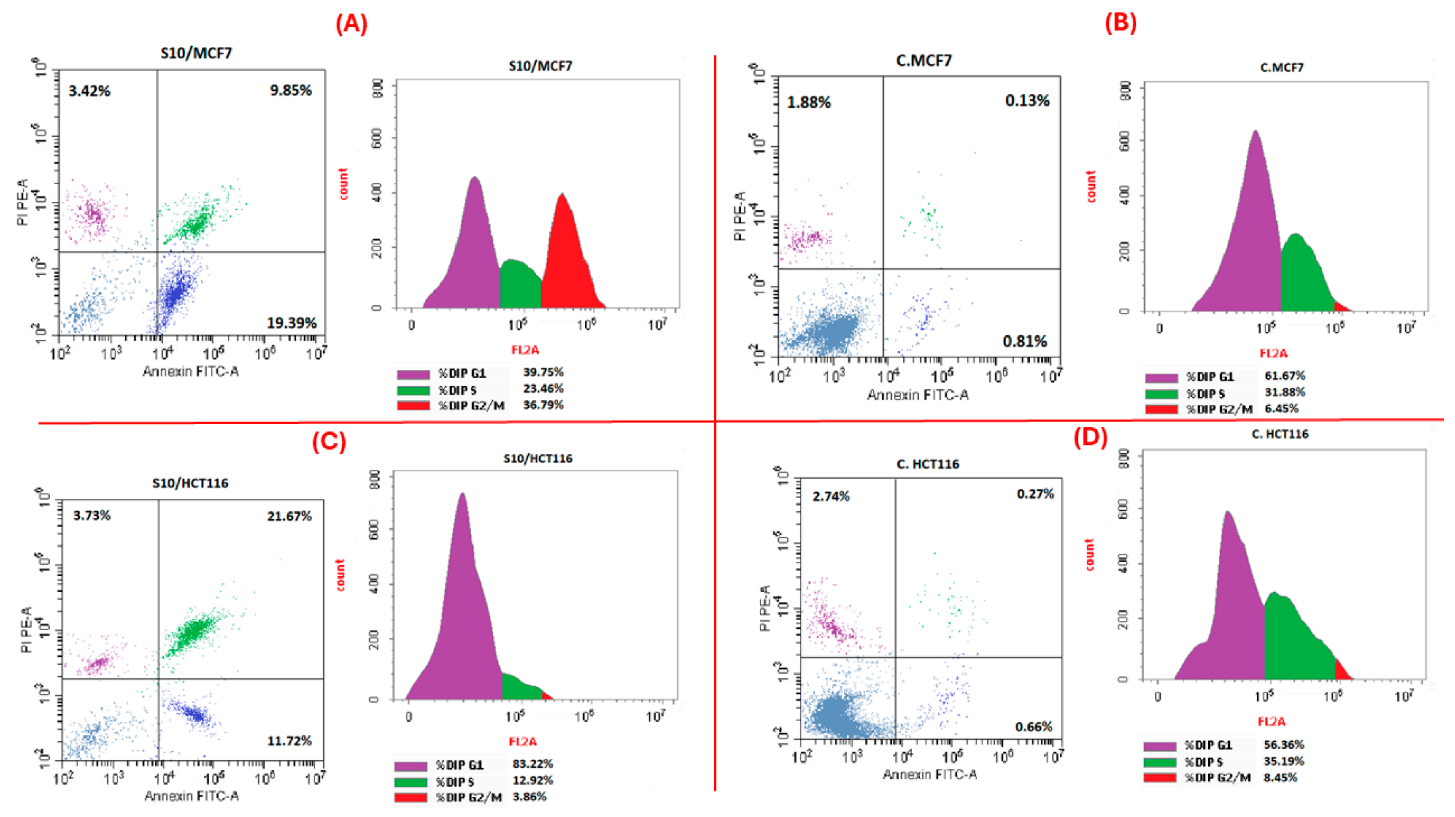
Flow cytometric analysis of cell cycle distribution and apoptosis in MCF-7 and HCT-116 cells following treatment with compound **10**. (**A**) Compound **10**-treated MCF-7 cells; (**B**) untreated MCF-7 control cells; (**C**) compound **10**-treated HCT-116 cells; and (**D**) untreated HCT-116 control cells. Treatment with compound **10** markedly altered cell cycle distribution and increased apoptotic cell populations relative to the corresponding untreated controls.

To investigate the mechanism responsible for the antiproliferative activity of compound **10**, its influence on cell cycle progression and apoptosis was examined in MCF-7 and HCT-116 cells (Figure 6 and Tables S5 and S6). Exposure to compound **10** markedly disturbed cell cycle progression in both cell lines; however, the pattern of arrest differed according to the cellular background. In MCF-7 cells, the proportions of cells in the G_0_/G_1_ and S phases declined from 61.67% and 31.88% to 39.75% and 23.46%, respectively, whereas the G_2_/M population increased markedly from 6.45% to 36.79%, indicating preferential arrest at the G_2_/M checkpoint. Conversely, HCT-116 cells accumulated predominantly in the G_0_/G_1_ phase, where the cell population increased from 56.36% to 83.22%, accompanied by reductions in the S phase (35.19% to 12.92%) and G_2_/M phase (8.45% to 3.86%). These findings demonstrate that compound **10** induces cell line-dependent cell-cycle arrest, suggesting that its antiproliferative activity is associated with disruption of key regulatory checkpoints governing cell-cycle progression. The mechanism of growth inhibition was further explored by Annexin V-FITC/PI staining, which revealed that loss of cell viability resulted primarily from apoptotic cell death rather than necrosis. In MCF-7 cells, the percentage of total apoptotic cells increased from 2.82% to 32.66%, with early apoptosis rising from 0.81% to 19.39% and late apoptosis from 0.13% to 9.85%. An even greater apoptotic response was detected in HCT-116 cells, where total apoptosis increased from 3.67% to 37.12%, while early and late apoptotic populations increased from 0.66% to 11.72% and from 0.27% to 21.67%, respectively. In contrast, necrotic cells remained at relatively low levels, increasing only from 1.88% to 3.42% in MCF-7 cells and from 2.74% to 3.73% in HCT-116 cells. The predominance of apoptosis over necrosis indicates that compound **10** suppresses cancer cell growth through activation of programmed cell death rather than nonspecific cytotoxicity. Importantly, these biological findings are in good agreement with the potent inhibition of EGFR, PI3K, and mTOR observed in the biochemical enzyme assays. Since the EGFR/PI3K/mTOR signaling pathway plays a central role in regulating cell-cycle progression and apoptosis, the combined biochemical and cellular findings support the proposed involvement of this pathway in the antiproliferative activity of compound **10**. Nevertheless, direct validation of downstream pathway modulation at the protein level was beyond the scope of the present study, and therefore the precise cellular signaling events require further investigation. The subsequent molecular docking and molecular dynamics studies provide additional computational support for the proposed mechanism.

#### 3.2.4. Effect of Compound **10** on Mitochondrial Membrane Potential (Δψm) in Hct-116 Cells

**Figure 7:**
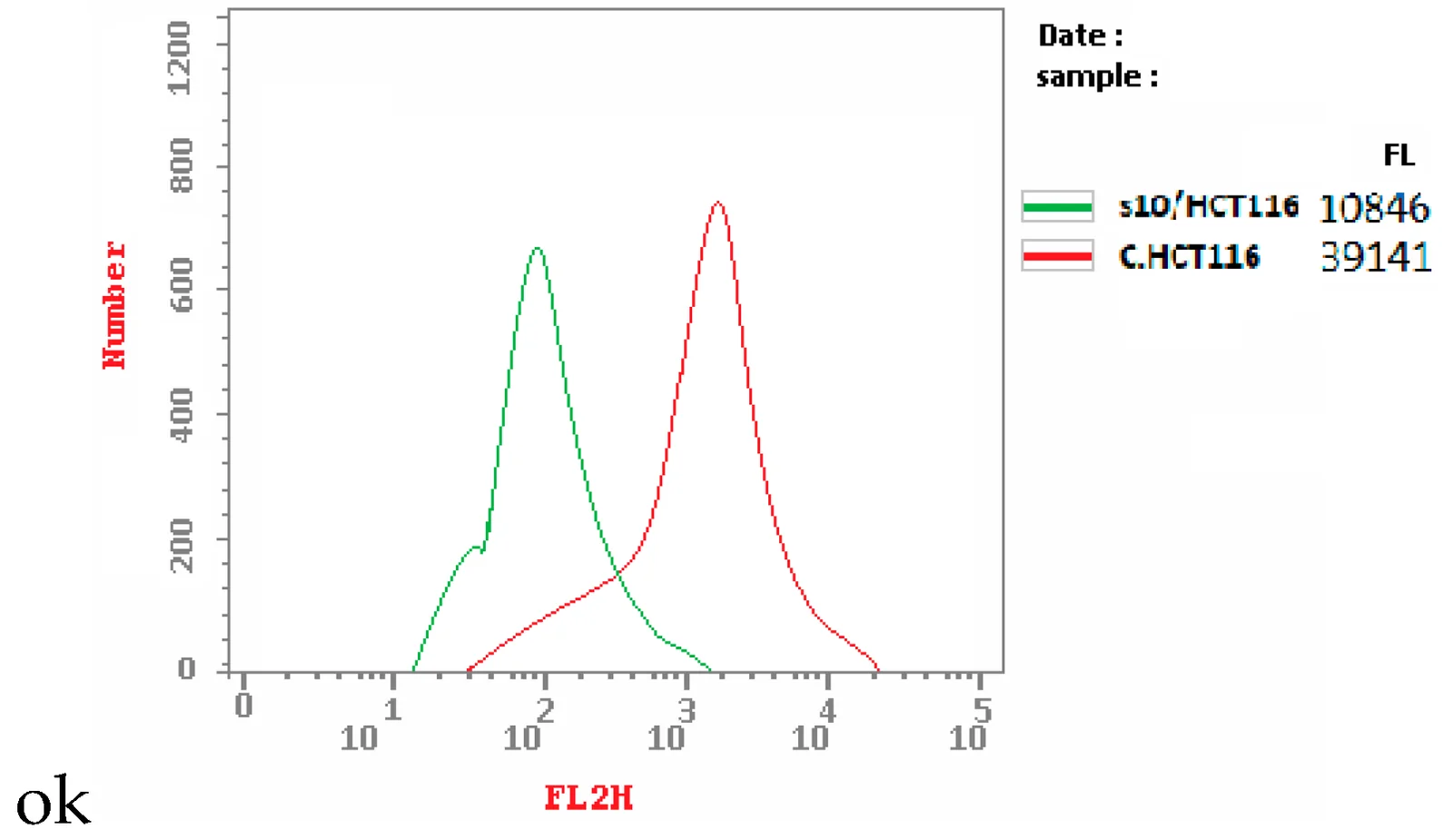
Flow cytometric analysis of mitochondrial membrane potential (ΔΨm) in HCT-116 cells after treatment with compound **10**. A marked reduction in fluorescence intensity was observed in treated cells, indicating mitochondrial membrane depolarization compared with the untreated control.

To determine the contribution of mitochondrial dysfunction to the apoptotic response induced by compound **10**, the mitochondrial membrane potential (ΔΨm) was monitored in HCT-116 cells. The quantitative findings are presented in Tables S7 and S8, and the representative fluorescence histogram is shown in Figure 7. A substantial loss of mitochondrial membrane potential was observed following treatment, with fluorescence intensity decreasing from 39,141 in untreated cells to 10,846 in treated cells, corresponding to 27.71% of the control level. The pronounced leftward shift in the treated cell population toward lower fluorescence intensity confirmed mitochondrial depolarization, indicating a marked reduction in ΔΨm. Since disruption of mitochondrial membrane potential represents an early hallmark of the intrinsic apoptotic pathway, these findings provide strong evidence that compound **10** induces mitochondria-mediated apoptosis. Moreover, the observed mitochondrial dysfunction is fully consistent with the Annexin V apoptosis assay and further supports that the potent antiproliferative activity of compound **10** is mediated through activation of the intrinsic apoptotic pathway, most likely as a downstream consequence of EGFR/PI3K/mTOR pathway inhibition.

#### 3.2.5. Effect of Compound **10** on Intracellular Reactive Oxygen Species (Ros) Generation in Hct-116 Cells

To further characterize the mechanism responsible for compound **10**-induced apoptosis, intracellular reactive oxygen species (ROS) production was quantified in HCT-116 cells. As summarized in Table 3, treatment with compound **10** markedly elevated ROS levels from 51.56 ± 2.4 to 185.94 ± 7.7 pg/mL, corresponding to an approximately 3.55-fold increase relative to untreated cells. This substantial ROS accumulation indicates pronounced oxidative stress capable of disrupting intracellular redox homeostasis, damaging essential cellular macromolecules, and impairing mitochondrial function. The observed ROS overproduction is fully consistent with the previously detected collapse of the mitochondrial membrane potential (ΔΨm), supporting the involvement of oxidative stress in mitochondrial depolarization and subsequent activation of the intrinsic apoptotic pathway. Furthermore, these findings complement the Annexin V apoptosis and kinase inhibition results, indicating that ROS-mediated mitochondrial dysfunction represents an important downstream event contributing to the potent antiproliferative activity of compound **10**.

#### 3.2.6. Modulation of Apoptosis-Related Gene Expression by Compound **10**

To obtain further molecular evidence for the apoptotic mechanism of compound **10**, the expression of key apoptosis-associated genes was quantified in HCT-116 cells using RT-PCR (Table 4). Treatment with compound **10** produced a pronounced increase in the expression of the pro-apoptotic markers caspase-3 (9.71-fold), caspase-9 (5.39-fold), and Bax (6.82-fold), whereas the expression of the anti-apoptotic Bcl-2 gene was markedly reduced to 0.22-fold relative to untreated cells. The simultaneous upregulation of caspase-9, caspase-3, and Bax, together with suppression of Bcl-2, reflects activation of the mitochondrial apoptotic signaling cascade. These transcriptional changes closely parallel the previously observed elevation in intracellular ROS, loss of mitochondrial membrane potential (ΔΨm), and increased apoptotic cell populations, providing additional molecular evidence that compound **10** induces apoptosis through ROS-mediated mitochondrial dysfunction followed by activation of the caspase-dependent pathway. Collectively, these molecular findings are in excellent agreement with the enzymatic inhibition, cell-cycle, and apoptosis studies, supporting a coherent mechanistic pathway in which inhibition of the EGFR/PI3K/mTOR signaling axis triggers mitochondrial dysfunction and culminates in caspase-dependent apoptotic cell death.

#### 3.2.7. In Vitro Antimicrobial Activity

**Figure 8:**
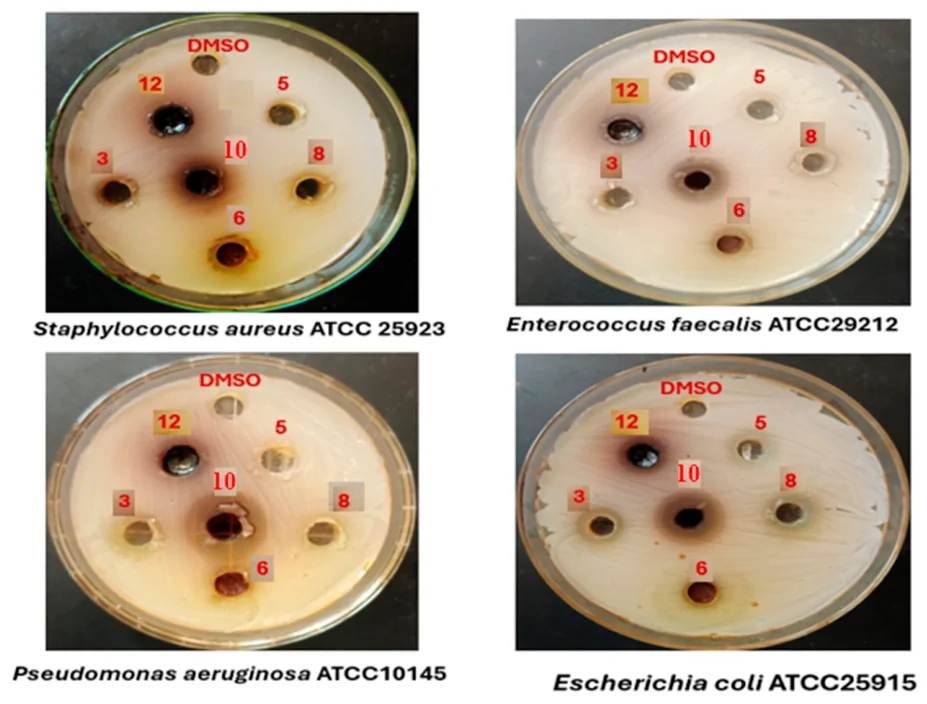
Representative agar-well diffusion assay demonstrating the growth inhibition produced by compounds **12**, **5**, **3**, **10**, **8**, and **6** against the selected bacterial strains. Wells containing DMSO served as the negative control.

Based on the preliminary cytotoxicity screening, the most active derivatives (**12**, **5**, **3**, **10**, **8**, and **6**) were selected for antimicrobial evaluation. Given that many bioactive heterocycles exhibit both anticancer and antimicrobial activities, these compounds were screened by the agar-well diffusion method to assess their potential dual biological activity (Table 5, Figure 8). The tested compounds displayed variable antibacterial activities, with compound **10** showing the broadest and most potent activity against all investigated strains. It produced inhibition zones of 16.50 ± 0.09 mm against *Pseudomonas aeruginosa* ATCC10145, 14.55 ± 0.05 mm against *Escherichia coli* ATCC25915, 11.00 ± 0.08 mm against *Staphylococcus aureus* ATCC25923, and 10.50 ± 0.20 mm against Enterococcus faecalis ATCC29212. Notably, its activity exceeded that of ciprofloxacin against all four tested bacteria, highlighting its remarkable broad-spectrum antibacterial potential. In contrast, the remaining compounds exhibited weak or strain-dependent activity. Compound **12** inhibited *E. faecalis*, *E. coli*, and *P. aeruginosa* (3.00–4.00 mm) but was inactive against *S. aureus*. Compound **3** showed moderate inhibition of *E. coli* (5.00 ± 0.08 mm), *P. aeruginosa* (4.50 ± 0.10 mm), and *S. aureus* (4.50 ± 0.00 mm) without activity against *E. faecalis*. Compounds **5** and **8** inhibited only *E. coli*, whereas compound **6** was active exclusively against *S. aureus*. Owing to its superior broad-spectrum activity, compound **10** was selected for MIC, antibiofilm, molecular docking, and molecular dynamics studies to further investigate its antibacterial mechanism.

#### 3.2.8. Determination of Minimum Inhibitory Concentrations (Mics)

The antibacterial potency of the most active derivatives (**12**, **3**, and **10**) was further characterized by determining their minimum inhibitory concentrations (MICs) against the tested bacterial strains (Table 6). Among the evaluated compounds, compound **10** displayed the greatest antibacterial efficacy, producing MIC values of 5.00 μg/mL against *Pseudomonas aeruginosa*, 10.00 μg/mL against both *Escherichia coli* and *Staphylococcus aureus*, and 20.00 μg/mL against *Enterococcus faecalis*. Notably, these values were superior to those of ciprofloxacin against *P. aeruginosa*, *E. coli*, and *S. aureus*, indicating that lower concentrations of compound **10** were sufficient to suppress bacterial growth. Compound **12** demonstrated intermediate antibacterial activity, with MIC values of 20.00 μg/mL against *E. faecalis*, *E. coli*, and *P. aeruginosa*, while a higher concentration (40.00 μg/mL) was required to inhibit *S. aureus*. In comparison, compound **3** exhibited the weakest antibacterial performance, showing an MIC of 80.00 μg/mL against *E. faecalis* and 20.00 μg/mL against the remaining bacterial strains. The MIC data closely paralleled the inhibition zone results obtained from the agar-well diffusion assay and further established compound **10** as the most promising antibacterial candidate for subsequent antibiofilm evaluation and computational investigations, including molecular docking and molecular dynamics simulations.

All experiments were performed as biological triplicate and assigned as Mean ± SE. Ciprofloxacin were used as standard antibacterial agents.

#### 3.2.9. Evaluation of Antibiofilm Activity

The antibiofilm activities of compounds **12**, **3**, and **10** were evaluated against the tested bacterial strains (Table 7). Consistent with the antimicrobial and MIC results, compound **10** exhibited the strongest antibiofilm activity, with inhibition percentages of 42.80 ± 0.10% against *Pseudomonas* aeruginosa, 35.55 ± 0.07% against Escherichia coli, 27.50 ± 0.08% against *Enterococcus faecalis*, and 25.00 ± 0.08% against *Staphylococcus aureus*. Its activity exceeded that of ciprofloxacin against *P. aeruginosa* and *E. coli* and was comparable, although slightly lower, against *E. faecalis* and *S.* aureus. Compound **3** showed moderate antibiofilm activity against *E.* coli (21.50 ± 0.08%), *P. aeruginosa* (18.50 ± 0.09%), and *S. aureus* (15.20 ± 0.10%), with no detectable effect on *E. faecalis*. In contrast, compound **12** displayed weak activity, inhibiting only the Gram-negative strains, with biofilm inhibition values of 16.30 ± 0.10% (*E. coli*) and 15.30 ± 0.08% (*P. aeruginosa*). These findings further identify compound **10** as the lead antibacterial derivative because of its superior inhibition of both bacterial growth and biofilm formation.

#### 3.2.10. DPPH and ABTS Radical Scavenging Activity

The antioxidant potential of the selected derivatives was investigated using the DPPH and ABTS radical scavenging assays (Table 8). Among the evaluated compounds, compound **10** displayed the strongest antioxidant performance, achieving radical scavenging values of 27.11 ± 0.08% in the DPPH assay and 16.30 ± 0.09% in the ABTS assay. This superior activity was accompanied by the lowest IC_50_ values; namely, 112.50 ± 0.12 μg/mL and 153.37 ± 0.08 μg/mL, respectively. Compounds **3** and **5** also exhibited noticeable DPPH radical scavenging activity, with IC_50_ values of 113.12 ± 0.09 and 115.42 ± 0.11 μg/mL, whereas their activities in the ABTS assay were comparatively weaker. Compound **12** showed moderate antioxidant capacity, while compounds **8** and **6** were the least effective, as reflected by lower radical scavenging percentages and higher IC_50_ values in both assays. Although none of the synthesized derivatives matched the potency of the reference antioxidant BHT (IC_50_ = 33.24 μg/mL for DPPH and 54.94 μg/mL for ABTS), compound **10** exhibited the most balanced biological profile by combining potent anticancer, antibacterial, antibiofilm, and antioxidant activities.

**Figure 9:**
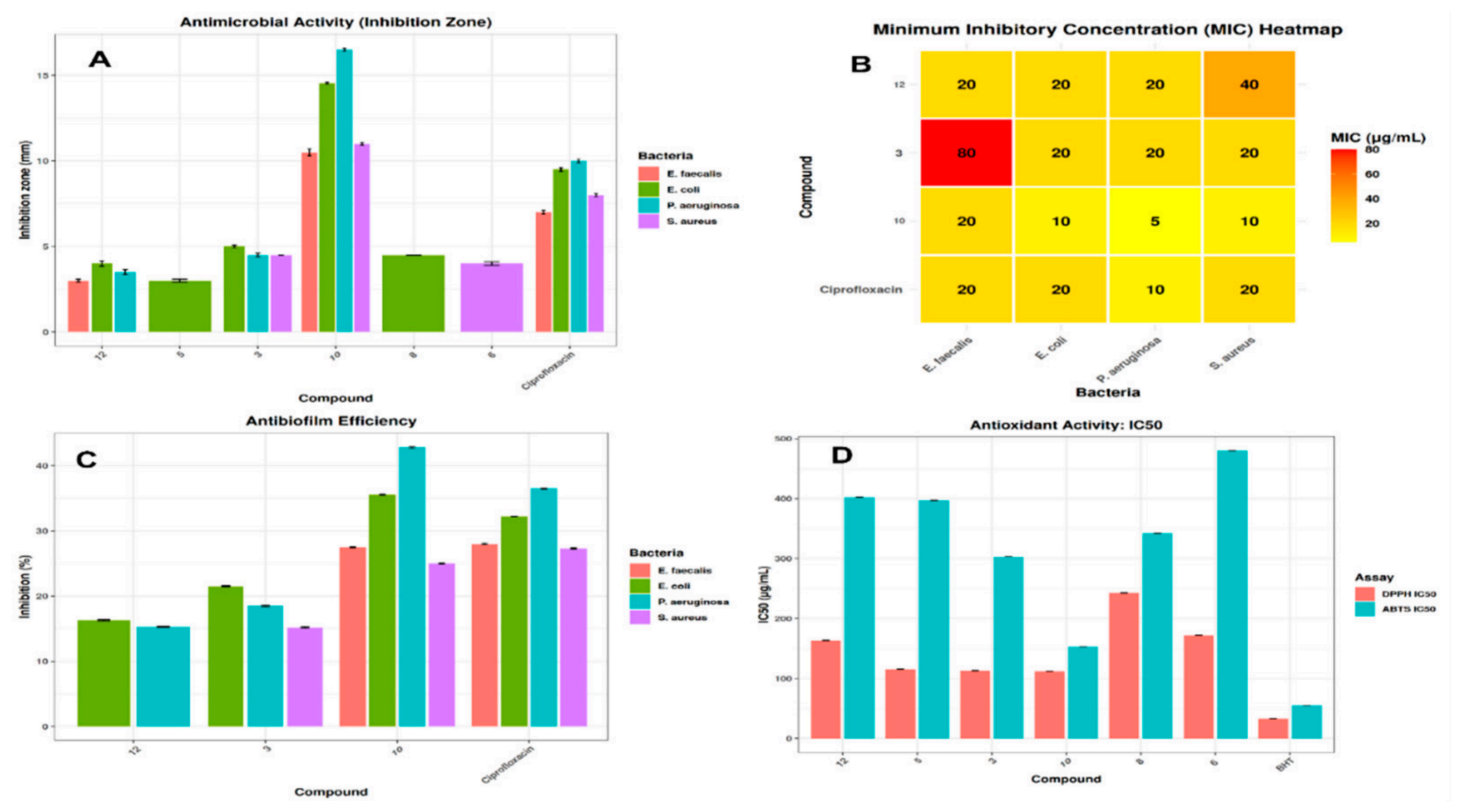
Summary of the biological activities of the synthesized morpholine derivatives. (**A**) Agar-well diffusion assay showing antibacterial activity of compounds **12**, **5**, **3**, **10**, **8**, and **6**. (**B**) Heat map of the minimum inhibitory concentrations (MICs) for the most active antibacterial derivatives (**12**, **3**, and **10**). (**C**) Antibiofilm activity of compounds **12**, **3**, and **10**. (**D**) Antioxidant activity of compounds **12**, **5**, **3**, **10**, **8**, and **6** determined by the DPPH and ABTS radical scavenging assays.

At first glance, the observed antioxidant activity in the DPPH and ABTS assays may appear inconsistent with the pronounced intracellular ROS generation induced by compound **10** in HCT-116 cells. However, these findings reflect fundamentally different biological contexts rather than contradictory mechanisms. The DPPH and ABTS assays are cell-free chemical tests that evaluate the intrinsic radical scavenging capacity of a compound, whereas intracellular ROS production depends on complex cellular processes, including mitochondrial dysfunction, redox homeostasis, and metabolic regulation. Therefore, a compound may exhibit antioxidant activity in chemical assays while simultaneously promoting ROS accumulation in cancer cells through disruption of mitochondrial function and oxidative homeostasis. This interpretation is fully consistent with the observed mitochondrial membrane depolarization and apoptosis induced by compound **10**, suggesting that intracellular ROS generation represents a cell-dependent pro-apoptotic mechanism rather than a contradiction to its intrinsic antioxidant properties (Figure 9).

Results are expressed as the mean ± SE of three independent biological replicates. BHT (50 μg/mL) was included as the positive antioxidant control.

#### 3.2.11. Structure–Activity Relationship (SAR) for Antibacterial Activity

**Figure 10:**
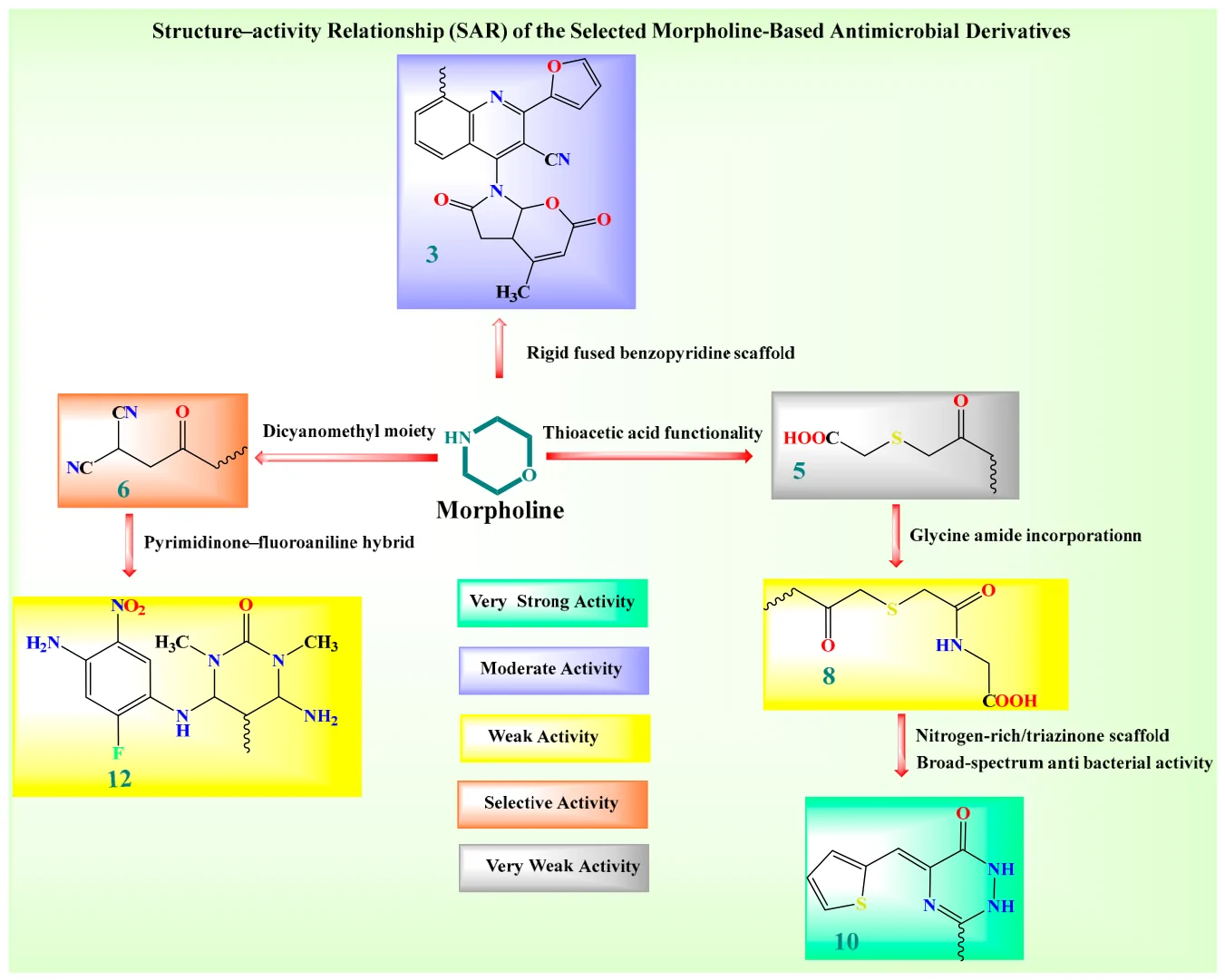
SARs of the selected morpholine-based antimicrobial derivatives. The figure highlights the effect of key structural modifications on antibacterial activity, demonstrating that incorporation of a nitrogen-rich triazinone scaffold markedly enhanced antibacterial potency, whereas thioacetic acid, dicyanomethyl, glycine amide, and pyrimidinone-based modifications resulted in limited, selective, or weak activities.

As summarized in Table 5, Table 6, Table 7 and Table 8, structural modification of the morpholine scaffold markedly influenced the antibacterial, antibiofilm, and antioxidant properties of the synthesized derivatives. Based on their antiproliferative activity, compounds **12**, **5**, **3**, **10**, **8**, and **6** were selected for antimicrobial evaluation to explore their multifunctional potential. Within this series, the fused pyrano-pyrrole-2,6-dione derivative **3** exhibited moderate broad-spectrum antibacterial activity, likely owing to its rigid polycyclic framework and multiple carbonyl groups that favor hydrogen-bonding interactions with bacterial targets. In contrast, the fluoronitroaniline-substituted pyrimidinone **12** showed only weak antibacterial activity despite its excellent anticancer potency, indicating that structural features promoting kinase inhibition do not necessarily enhance antimicrobial activity. Sulfur-containing intermediates **5**, **6**, and **8** displayed weak or strain-selective activity, suggesting that the thioacetic acid, dicyanomethyl, or glycine-derived amide functionalities alone are insufficient for broad-spectrum antibacterial effects. A marked improvement was achieved after cyclization to the nitrogen-rich triazinone scaffold of compound **10**, which exhibited the broadest antibacterial spectrum, the lowest MIC values, and the highest antibiofilm and antioxidant activities. This superior performance is likely attributable to the synergistic combination of the triazinone core, sulfur linker, and morpholine pharmacophore, providing favorable molecular rigidity, electronic distribution, and hydrogen-bonding capability for stronger interactions with bacterial targets. Overall, the SAR indicates that combining nitrogen-rich fused heterocycles with appropriate electronic properties and molecular rigidity is essential for maximizing antibacterial activity. Accordingly, compound **10** emerged as the lead multifunctional derivative and was selected for subsequent molecular docking and molecular dynamics studies against *Staphylococcus aureus* DNA gyrase (Figure 10).

### 3.3. Computational Investigations

#### 3.3.1. Physicochemical Properties and Admet Prediction

**Figure 11:**
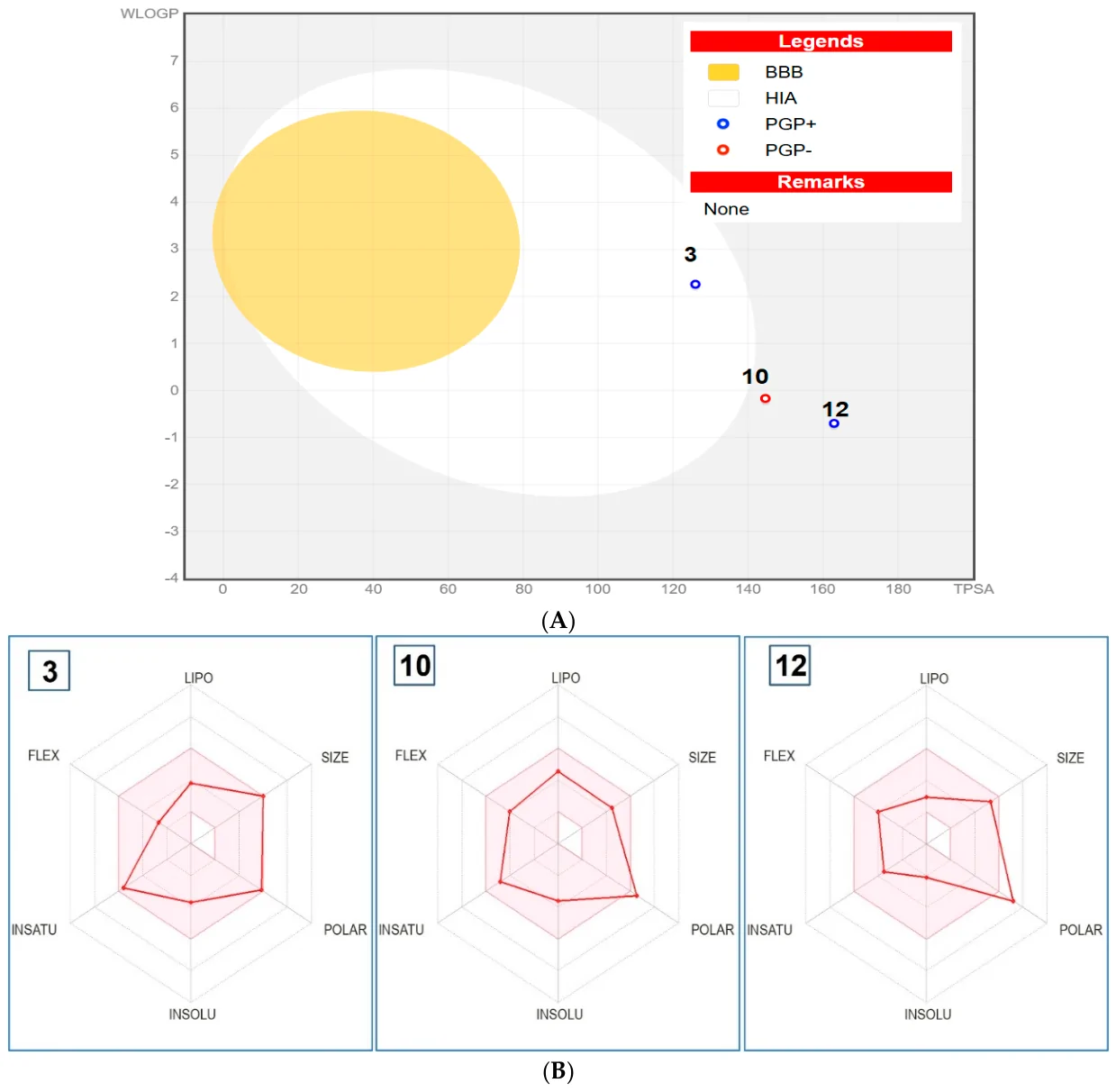
(**A**) BOILED-Egg prediction of gastrointestinal absorption (HIA) and blood–brain barrier (BBB) penetration for compounds **3**, **10**, and **12**. PGP− and PGP+ indicate predicted non-substrates and substrates of P-glycoprotein, respectively. (**B**) SwissADME bioavailability radar illustrating the physicochemical characteristics governing oral bioavailability. The red profile corresponds to the calculated properties of each compound, whereas the shaded pink region defines the optimal physicochemical space for orally active molecules.

To evaluate the pharmacokinetic behavior and drug-likeness of the most active derivatives (**3**, **10**, and **12**), SwissADME and pkCSM analyses were performed, and the calculated descriptors are summarized in Table 9 and Table 10. Compound **3** fully satisfied both Lipinski’s Rule of Five and Veber’s criteria, exhibiting favorable physicochemical properties (MW = 498.49 Da, HBA = 8, HBD = 0, TPSA = 125.97 Å^2^, MLogP = 0.35), consistent with good drug-likeness. In contrast, compounds **10** and **12** showed minor deviations, mainly related to their higher polarity (TPSA = 144.62 and 162.98 Å^2^, respectively), which may adversely affect oral bioavailability. These observations were further supported by the SwissADME bioavailability radar and BOILED-Egg model (Figure 11), where compound **3** displayed the most favorable oral absorption profile, whereas compounds **10** and **12** were predicted to exhibit lower gastrointestinal absorption and poor blood–brain barrier permeability. Furthermore, compounds **3** and **12** were predicted to be P-glycoprotein substrates, suggesting possible susceptibility to active efflux, while compound **10** was identified as a non-substrate, indicating a lower likelihood of P-glycoprotein-mediated efflux and potentially improved intracellular retention. Overall, the ADMET predictions suggest that compound **3** possesses the most favorable pharmacokinetic profile, whereas compound **10** combines excellent biological activity with acceptable predicted pharmacokinetic characteristics despite its relatively higher polarity.

Regarding metabolic stability and safety, the compounds displayed distinct interactions with cytochrome P450 enzymes. Compounds **3** and **10** were predicted to inhibit CYP3A4, whereas compound **12** showed no inhibitory activity. Toxicity predictions indicated that all three derivatives may exhibit hepatotoxicity. In addition, compound **12** was predicted to be AMES-positive, while compounds **3** and **12** showed potential hERG II inhibition. Nevertheless, none of the investigated compounds was predicted to cause skin sensitization. Overall, these ADMET predictions highlight potential pharmacokinetic and safety liabilities that should be considered during future structural optimization and experimental validation.

#### 3.3.2. Molecular Docking Simulation

**Figure 12:**
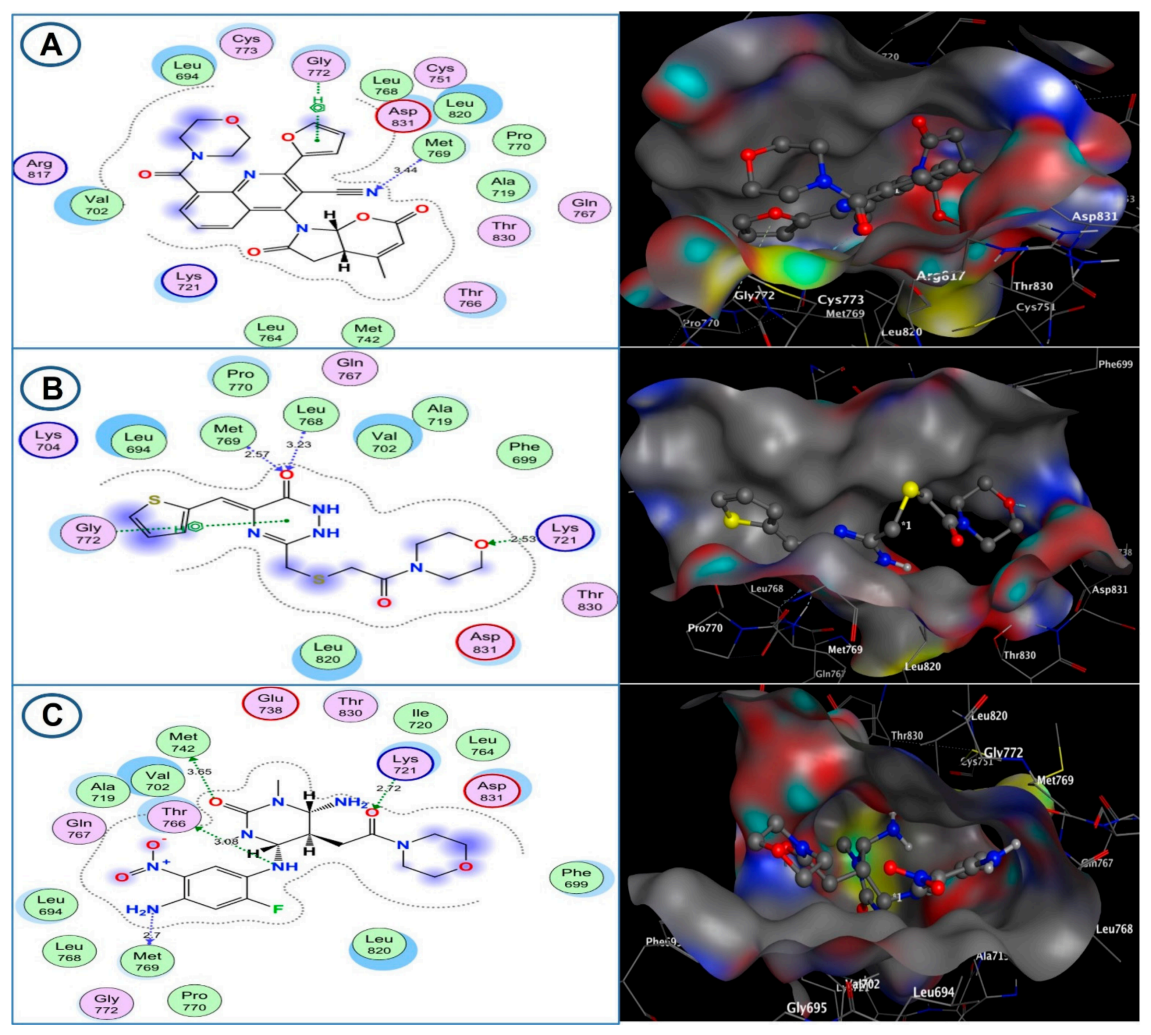
Predicted binding modes of the active morpholino-2-oxoethyl derivatives within the EGFR kinase domain (PDB ID: 1M17). (**A**) Compound **3**, (**B**) compound **10**, and (**C**) compound **12**, shown as two-dimensional interaction maps together with their corresponding three-dimensional binding poses.

To explore the molecular basis underlying the in vitro antiproliferative activity, the binding modes of the most active morpholino-2-oxoethyl derivatives (**3**, **10**, and **12**) were investigated by molecular docking against EGFR, PI3K, and mTOR (PDB IDs: 1M17, 3L54, and 4JT6, respectively) using MOE-Dock v2024.0601. The docking protocol was validated by redocking the co-crystallized ligands (erlotinib, LXX, and X6K), yielding RMSD values of 0.88, 1.25, and 0.72 Å with docking scores of −10.26, −9.82, and −10.14 kcal/mol, respectively, confirming the reliability of the adopted protocol (Figure S50). The predicted binding modes within the EGFR active site are shown in Figure 12. Compound **10** exhibited the most favorable docking score (−10.33 kcal/mol), followed by compounds **3** (−9.86 kcal/mol) and **12** (−9.42 kcal/mol). Compound **3** established two key interactions with **Gly772** and **Met769**, whereas compound **10** formed multiple hydrogen-bond interactions with **Lys721**, **Leu768**, and **Met769**, together with an additional arene–H interaction with **Gly772**, accounting for its superior predicted binding. Compound **12** also formed multiple hydrogen bonds with **Lys721**, **Met742**, **Thr766**, and **Met769**, supporting stable accommodation within the ATP-binding pocket. Overall, all three derivatives adopted favorable binding orientations within the EGFR active site, with compound **10** displaying the strongest predicted docking score, consistent with its superior enzymatic inhibitory activity.

**Figure 13:**
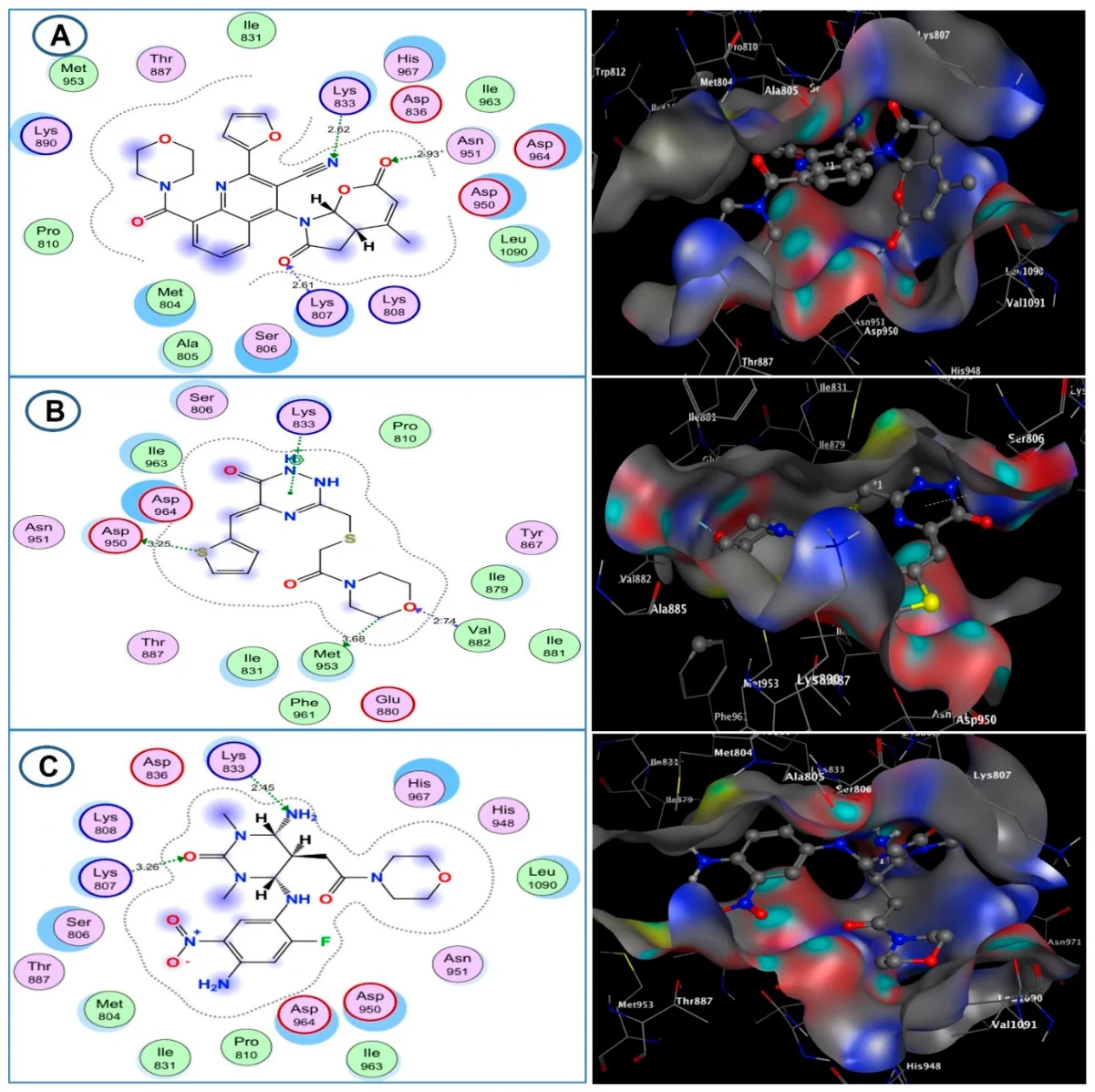
Molecular docking interaction patterns of compounds **3**, **10**, and **12** within the PI3K active site (PDB ID: 3L54). Panels (**A**–**C**) depict the two-dimensional interaction diagrams and their corresponding three-dimensional binding poses for compounds **3**, **10**, and **12**, respectively.

Interactions within the PI3K active site, as shown in Figure 13, demonstrate the structural stabilization of the main scaffolds of **3**, **10**, and **12** with energy scores of −8.30, −9.16, and −8.22 kcal/mol, respectively. Compound **3** (Figure 13A) is anchored by three hydrogen bond acceptors with **Lys807**, **Lys833**, and **Asn951** (distances = 2.61, 2.62, and 2.93 Å, respectively). Compound **10** (Figure 13B) shows stabilization via hydrogen bonding of morpholine with **Val882** and **Met953** (distances = 2.74 and 3.68 Å, respectively), in addition to arene-cation of triazinone scaffold with **Lys833**. Furthermore, thiophene sulfur presents an H-bond donor with **Asp950** sidechain (3.25 Å). Compound **12** (Figure 13C) forms two hydrogen bonds, notably with **Lys807** (3.26 Å) and **Lys833** (2.45 Å).

**Figure 14:**
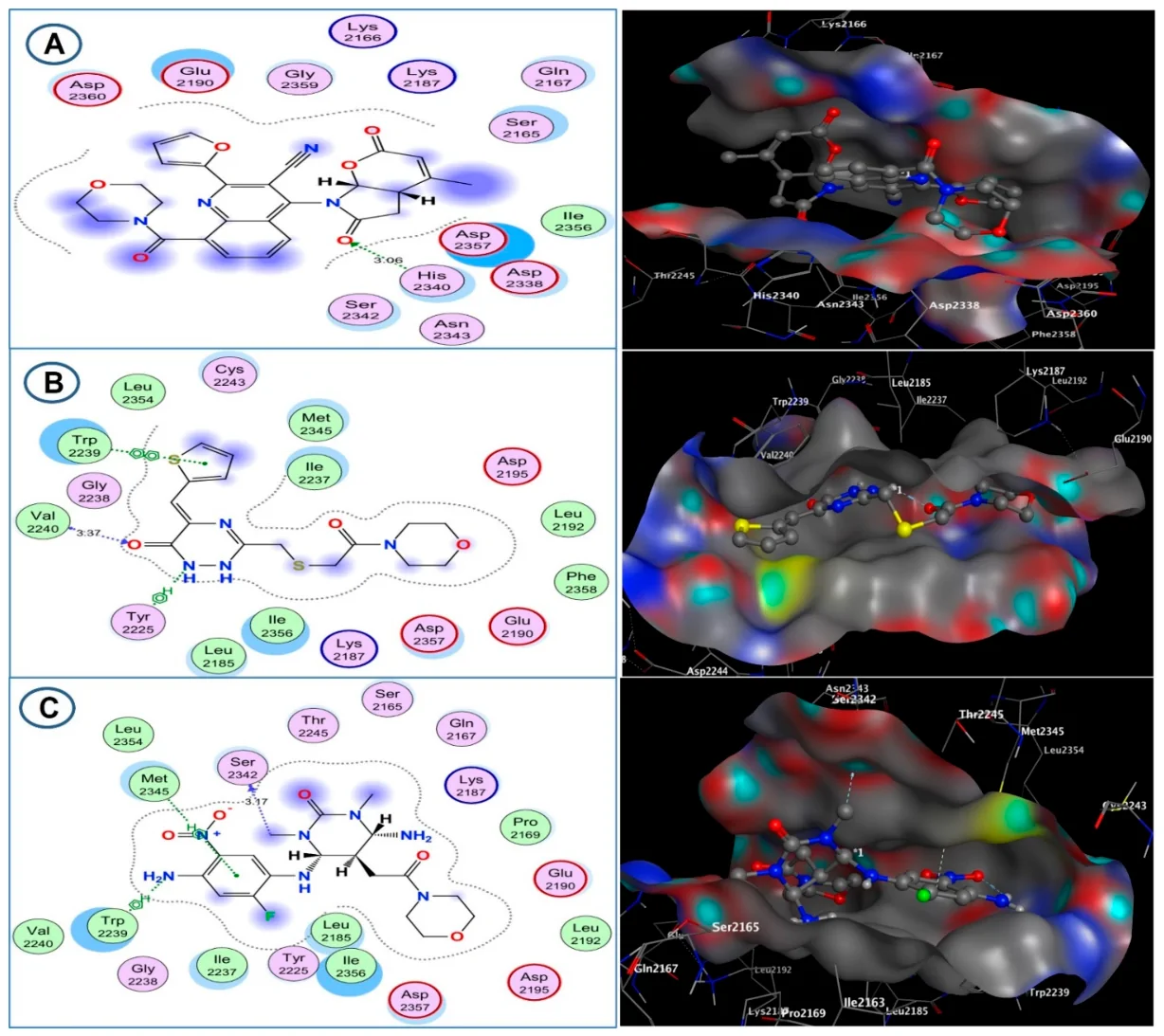
Molecular docking interaction profiles of compounds **3**, **10**, and **12** within the catalytic binding pocket of mTOR (PDB ID: 4JT6). Panels (**A**–**C**) illustrate the corresponding two-dimensional interaction diagrams and three-dimensional binding conformations for compounds **3**, **10**, and **12**, respectively.

Docking poses within the mTOR kinase domain, displayed in Figure 14, indicate specific stabilization modes of **3, 10,** and **12** with energy scores of –9.10, –10.20, and –9.34 kcal/mol, respectively. Compound **3** (Figure 14A) is stabilized by a hydrogen bond with **His2340** (3.06 Å). Compound **10** (Figure 14B) interacts via hydrogen bonding with **Val2240** (3.37 Å), in addition to pi-stacking with **Tyr2225** and **Trp2239**. Compound **12** (Figure 14C) demonstrates hydrogen bonding with **Ser2342** (3.17 Å), complemented by hydrophobic contacts involving **Trp2239** and **Met2345**.

**Figure 15:**
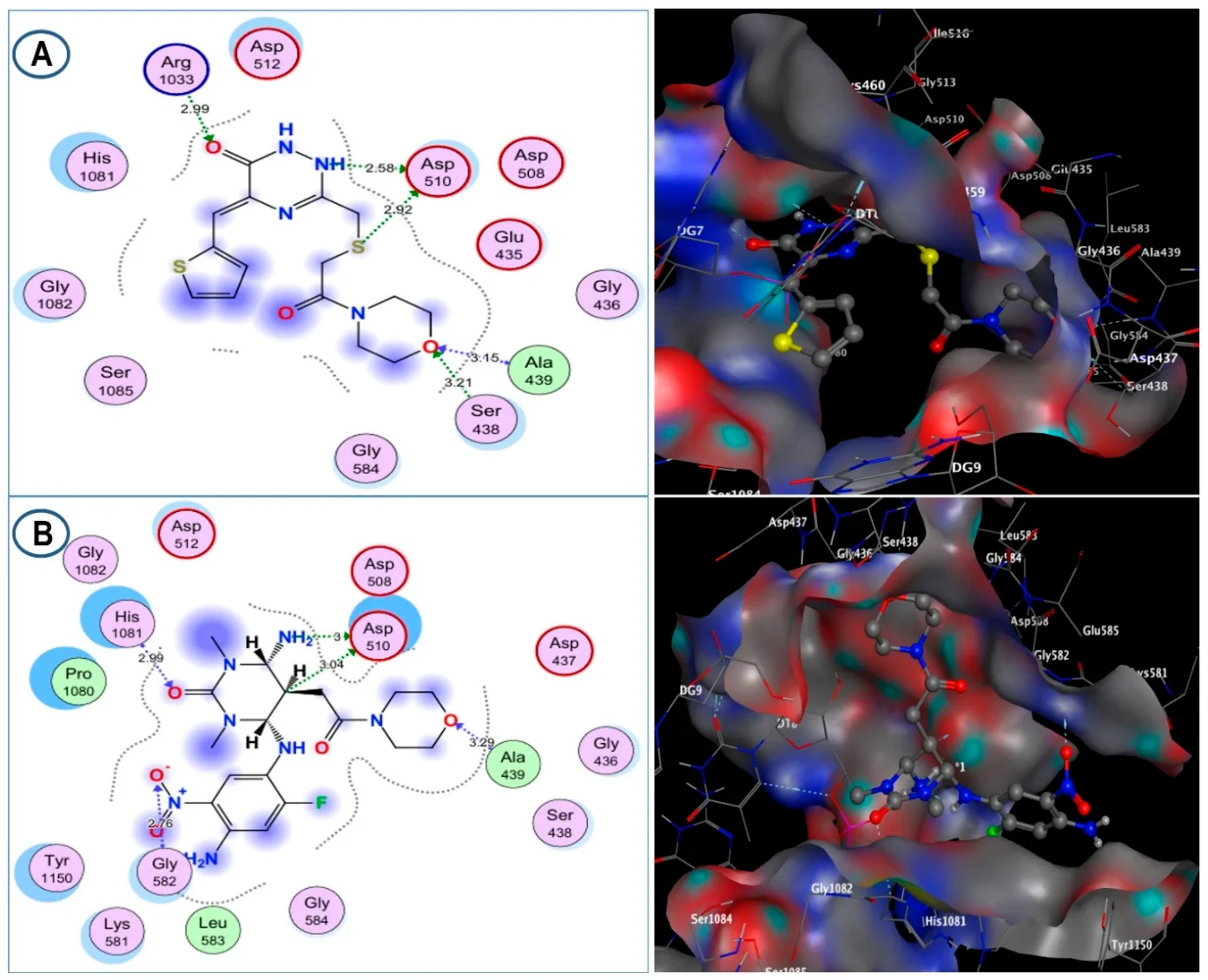
Predicted binding modes of the most active antibacterial derivatives within the *Staphylococcus aureus* DNA gyrase active site (PDB ID: 2XCT). (**A**) Compound **10** and (**B**) compound **12**, presented as two-dimensional interaction maps together with the corresponding three-dimensional binding poses.

To gain molecular insight into the antibacterial activity of the most active derivatives, compounds **10** and **12** were docked into the catalytic pocket of *Staphylococcus aureus* DNA gyrase (PDB ID: 2XCT). The docking protocol was validated by redocking the co-crystallized ligand ciprofloxacin, yielding an RMSD of 0.82 Å and a docking score of −9.75 kcal/mol, confirming the reliability of the adopted protocol (Figure S51). The predicted docking scores of compounds **10** and **12** were −9.86 and −9.33 kcal/mol, respectively (Figure 15). As illustrated in Figure 15A, compound **10** established multiple hydrogen-bond interactions with Arg1033 (2.99 Å), Asp510 (2.58 and 2.92 Å), Ser438, and Ala439 (3.21 and 3.15 Å, respectively), indicating extensive stabilization within the binding pocket. In comparison, compound **12** (Figure 15B) formed hydrogen-bond interactions with His1081 (2.99 Å), Asp510 (3.00 and 3.04 Å), and Ala439 (3.29 Å). Overall, both derivatives exhibited favorable binding orientations within the DNA gyrase active site, with compound **10** displaying the stronger predicted binding affinity, which is consistent with its superior antibacterial activity. These computational findings suggest that DNA gyrase may represent a potential molecular target for the investigated compounds. However, direct experimental validation of DNA gyrase inhibition was beyond the scope of the present study and will be required to confirm the proposed antibacterial mechanism.

Overall, compound **10** exhibited the most favorable binding characteristics across the investigated targets, adopting well-oriented binding conformations and establishing multiple interactions with catalytically important amino acid residues, including **Met769** in EGFR, **Val882 and Met953** in PI3K, **Val2240**, **Tyr2225**, and **Trp2239** in mTOR, as well as **Arg1033**, **Asp510**, **Ser438**, and **Ala439** in *Staphylococcus aureus* DNA gyrase. The predicted interaction patterns are consistent with the experimental enzyme inhibition, antiproliferative, and antibacterial findings, highlighting compound **10** as the most promising member of this series. Collectively, these computational findings support the proposed binding of compound **10** to the investigated molecular targets and provide additional evidence consistent with the experimental biological results. While the docking analyses suggest that DNA gyrase may represent a potential antibacterial target, direct experimental validation of DNA gyrase inhibition is required to confirm this proposed mechanism. Overall, the morpholino-2-oxoethyl scaffold represents a promising platform for the future development and optimization of multitarget anticancer and antibacterial agents.

#### 3.3.3. Molecular Dynamics Simulations

##### Dynamic Behavior and Binding Free Energy of the EGFR–Compound 10 Complex

**Figure 16:**
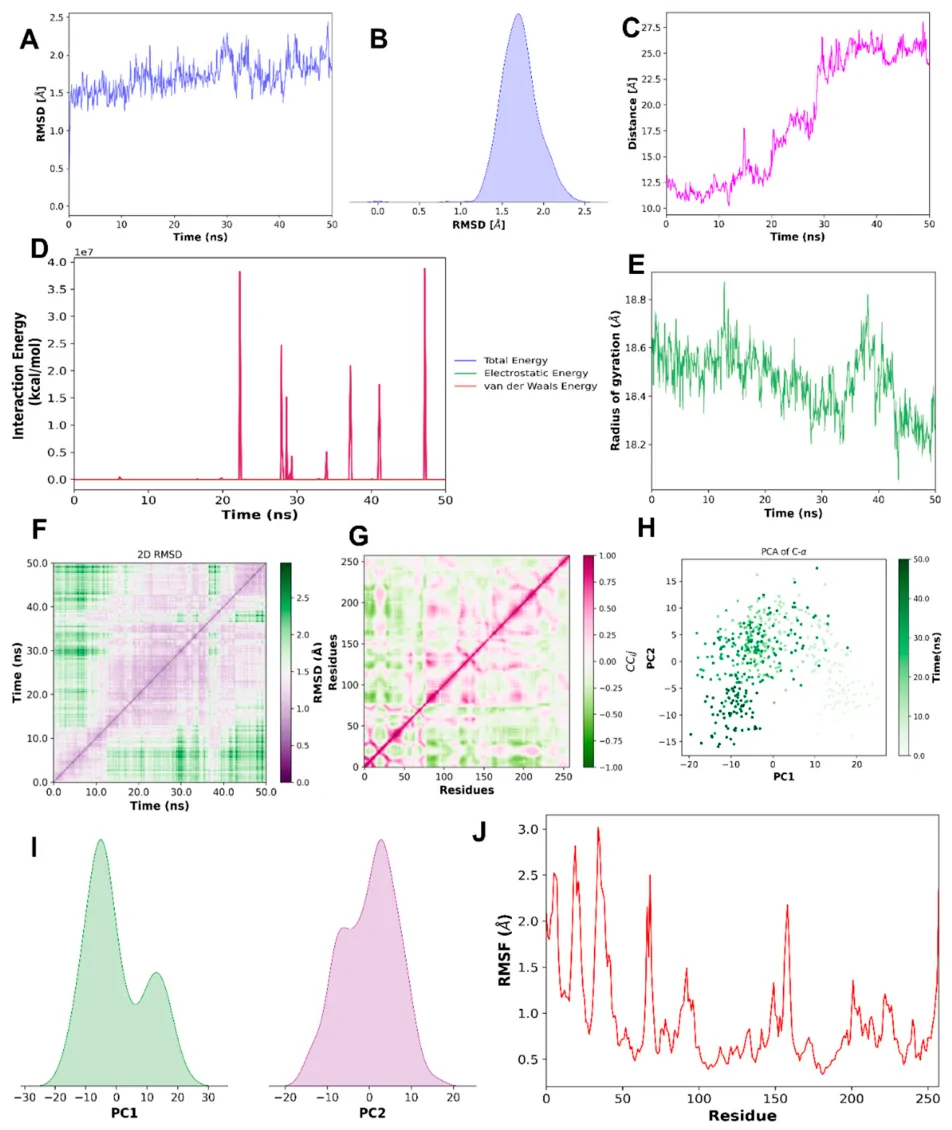
Molecular dynamics characterization of the EGFR–compound **10** complex over a 50 ns simulation. (**A**) Backbone RMSD. (**B**) Distribution of RMSD values. (**C**) Protein–ligand distance profile. (**D**) Interaction energy decomposition (total, electrostatic, and van der Waals contributions). (**E**) Radius of gyration (Rg). (**F**) Pairwise RMSD heat map. (**G**) Dynamic cross-correlation matrix (DCCM). (**H**) Principal component analysis (PCA) conformational projection. (**I**) Density distributions of PC1 and PC2. (**J**) Residue-wise RMSF profile.

The dynamic behavior of the EGFR–compound **10** complex was further investigated using a 50 ns molecular dynamics (MD) simulation. Structural stability, conformational flexibility, compactness, intermolecular interactions, and collective motions were analyzed using RMSD, RMSF, radius of gyration (Rg), interaction energy, principal component analysis (PCA), dynamic cross-correlation matrix (DCCM), and related trajectory descriptors (Figure 16A–J). The backbone RMSD remained relatively stable throughout the simulation, with fluctuations mainly between 1.4 and 2.1 Å, while the corresponding probability density profile indicated a dominant conformational state. The Rg values (18.2–18.7 Å) and RMSF analysis further demonstrated preservation of the overall protein compactness, with increased flexibility restricted mainly to the N-terminal region and a few loop segments. Similarly, the interaction energy profile remained predominantly stable, suggesting persistent ligand binding despite minor transient energetic fluctuations. PCA, DCCM, and the two-dimensional RMSD matrix collectively indicated limited conformational rearrangements and localized cooperative residue motions without evidence of major structural reorganization. Overall, these MD analyses demonstrate that the EGFR–compound **10** complex maintains a stable binding mode throughout the 50 ns simulation, supporting the docking results and the experimentally observed kinase inhibitory activity.

**Figure 17:**
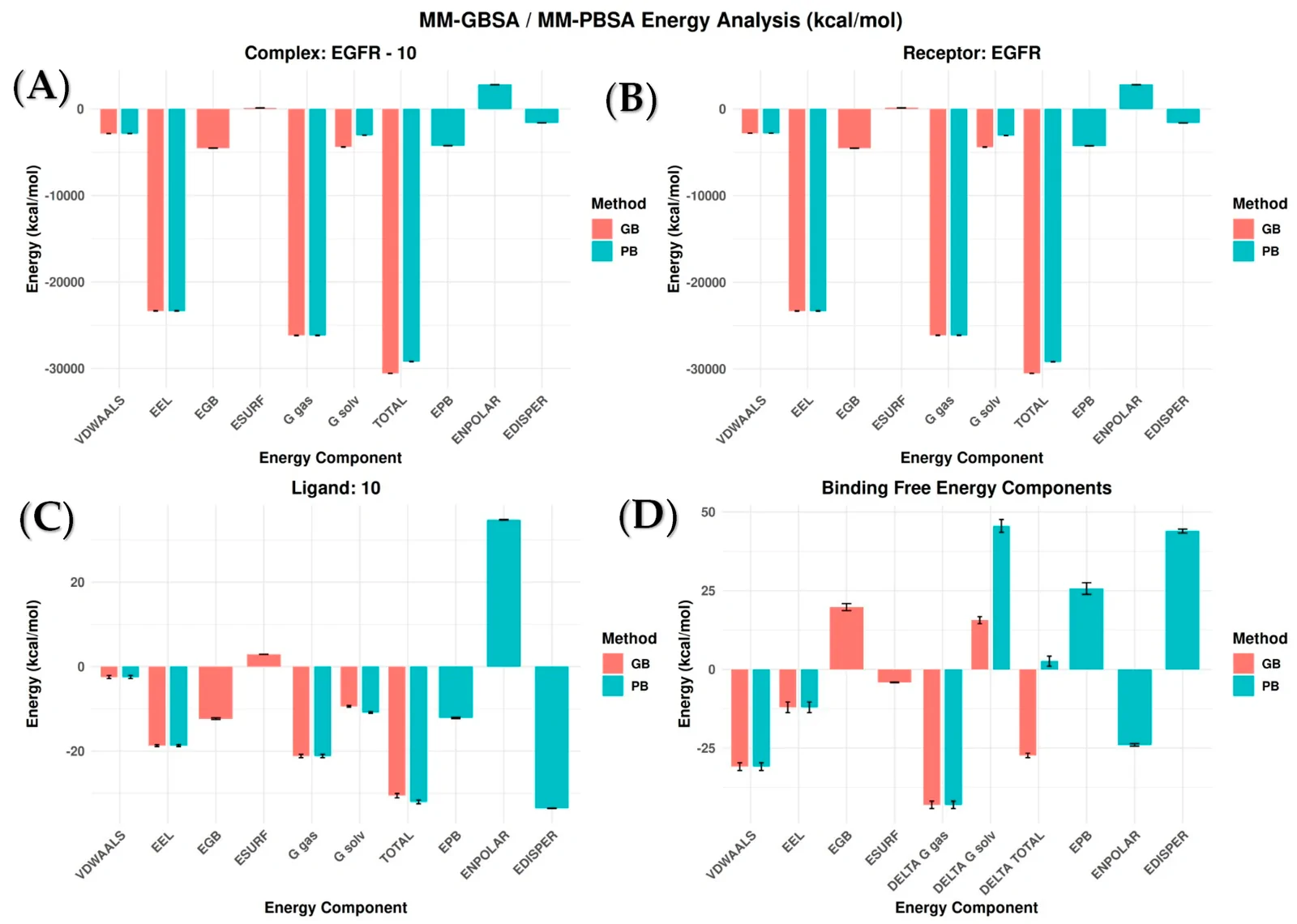
MM-GBSA/MM-PBSA energy component analysis of the EGFR–compound **10** complex. Panels show the calculated energetic contributions for the protein–ligand complex (**A**), EGFR (**B**), compound **10** (**C**), and the corresponding binding free-energy terms (**D**), where ΔG = Complex − Receptor − Ligand. Calculations were performed using the Generalized Born (GB) and Poisson–Boltzmann (PB) implicit solvent approaches. Energies are expressed in kcal/mol. Abbreviations: VDWAALS, van der Waals energy; EEL, electrostatic energy; EGB, Generalized Born polar solvation energy; EPB, Poisson–Boltzmann polar solvation energy; ESURF, nonpolar solvation energy (GB); ENPOLAR, nonpolar solvation energy (PB); EDISPER, dispersion energy; Ggas, gas-phase energy; Gsolv, solvation free energy; TOTAL, total free energy.

To complement the molecular dynamics analysis, the binding energetics of the EGFR–compound **10** complex were evaluated using MM-GBSA/MM-PBSA calculations based on representative snapshots extracted from the equilibrated region of the MD trajectory (Figure 17A–D). The MM-GBSA binding free energy (ΔG_bind) was calculated to be −23.44 kcal/mol, indicating energetically favorable complex formation. Both the Generalized Born (GB) and Poisson–Boltzmann (PB) models generated comparable energetic profiles, with van der Waals and electrostatic interactions representing the principal favorable contributors to binding, whereas the polar solvation terms opposed complex formation because of the energetic cost of desolvation. Despite minor quantitative differences between the GB and PB models, both approaches consistently predicted favorable ligand binding and demonstrated that the attractive nonpolar interactions outweighed the unfavorable polar solvation contributions. Overall, the MM-GBSA/MM-PBSA analyses, together with the molecular dynamics results, support the formation of a dynamically stable and energetically favorable EGFR–compound **10** complex, consistent with its potent experimental EGFR inhibitory activity.

##### Molecular Dynamics Simulation and MM-GBSA/MM-PBSA Binding Free Energy Analysis of the Ligand–*Staphylococcus Aureus* DNA Gyrase Complex

**Figure 18:**
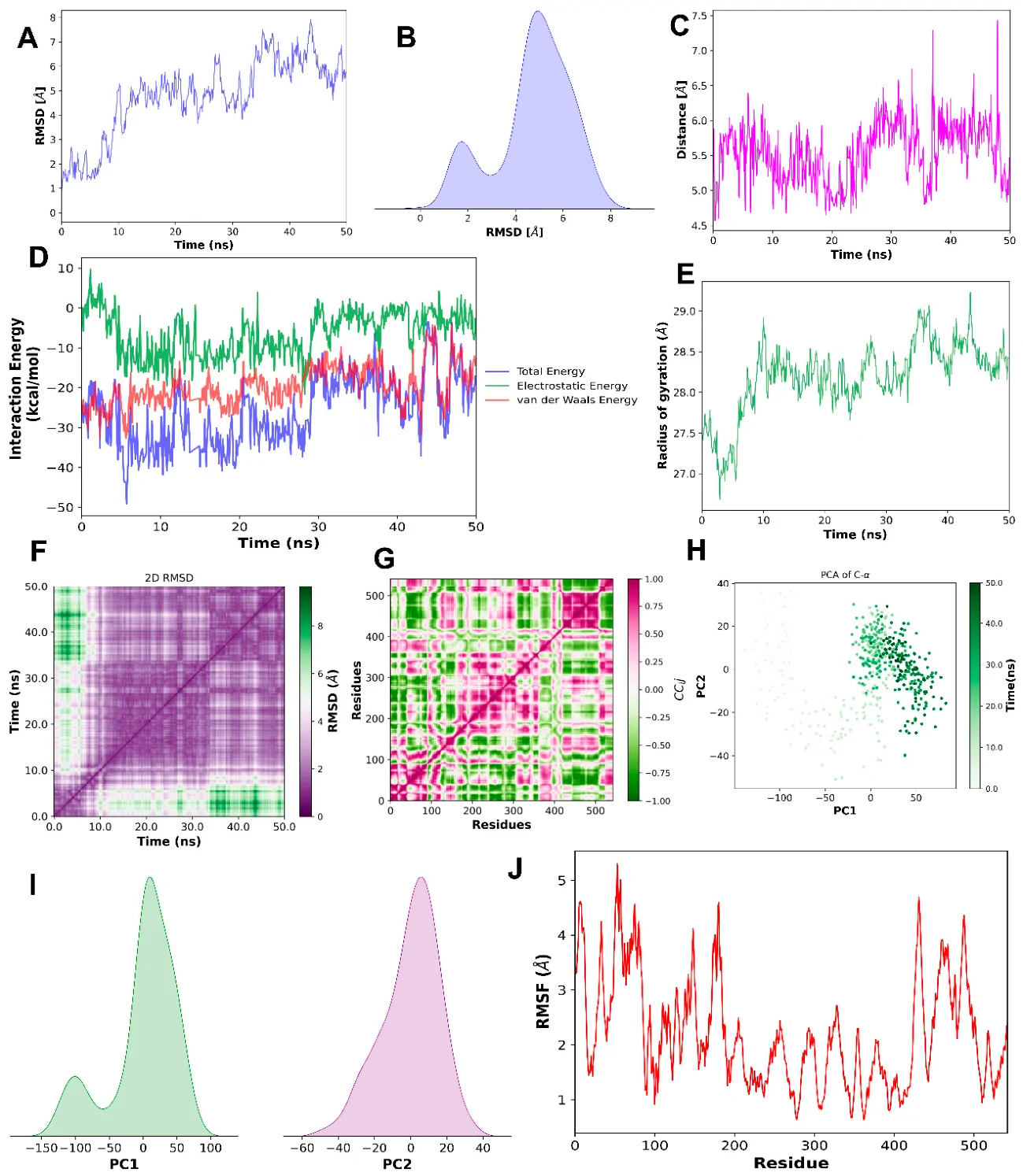
Dynamic and structural analyses of the *Staphylococcus aureus* DNA gyrase–compound **10** complex obtained from the 50 ns molecular dynamics simulation. (**A**) Backbone RMSD trajectory. (**B**) Distribution of RMSD values. (**C**) Protein–ligand distance profile. (**D**) Interaction energy components (total, electrostatic, and van der Waals). (**E**) Radius of gyration (Rg). (**F**) Pairwise 2D RMSD similarity matrix. (**G**) Dynamic cross-correlation matrix (DCCM) of residue motions. (**H**) Principal component analysis (PCA) conformational projection onto PC1 and PC2. (**I**) Probability density distributions of PC1 and PC2. (**J**) Residue-wise RMSF profile.

Following the docking study, the stability of the *Staphylococcus aureus* DNA gyrase–compound **10** complex was further examined by a 50 ns molecular dynamics (MD) simulation. Multiple trajectory descriptors, including RMSD, RMSF, protein compactness, interaction energies, principal component analysis (PCA), and dynamic cross-correlation analysis (DCCM), were evaluated to characterize the dynamic behavior of the complex (Figure 18A–J). The backbone Cα RMSD (Figure 18A) increased from ~1.2 Å at the beginning of the simulation to 4.0–8.0 Å after approximately 20–40 ns, reached a transient peak of ~8.0 Å around 40–45 ns, and finally stabilized at approximately 5.5–6.5 Å, indicating substantial conformational rearrangement without complete equilibration. Consistently, the RMSD probability density plot (Figure 18B) exhibited a bimodal distribution with populations centered at approximately 1–2 Å and 5 Å, confirming sampling of two major conformational states. The protein–ligand distance profile (Figure 18C) fluctuated between approximately 4.5 and 7.5 Å throughout the simulation, with transient excursions near 35 and 48 ns, indicating dynamic ligand repositioning within the binding pocket. The interaction energy profile (Figure 18D) remained predominantly favorable, with van der Waals interactions consistently ranging from approximately −10 to −30 kcal/mol, whereas the electrostatic contribution fluctuated between approximately −20 and +10 kcal/mol, indicating that ligand stabilization was primarily driven by van der Waals interactions. The radius of gyration (Figure 18E) remained within a narrow range of 27.2–28.8 Å, demonstrating preservation of the overall protein compactness despite the observed conformational flexibility. The 2D RMSD matrix (Figure 18F) revealed progressive structural transitions rather than rapid convergence to a single conformation. Similarly, the DCCM analysis (Figure 18G) demonstrated pronounced correlated and anti-correlated residue motions, indicating coordinated inter-domain dynamics. PCA (Figure 18H) showed conformational sampling over PC1 values of approximately −150 to +100 and PC2 values of approximately −40 to +40, while the bimodal PC1 density distribution (Figure 18I) further confirmed the existence of two dominant conformational substates. RMSF analysis (Figure 18J) revealed baseline residue fluctuations of approximately 2.0–4.0 Å, with maxima approaching 5.0 Å in the N-terminal region (residues ~1–60) and residues 420–460, whereas residues 200–350 exhibited comparatively lower fluctuations, indicating a more structurally constrained protein.

Collectively, these results indicate that compound **10** remained associated with the predicted DNA gyrase binding pocket throughout the simulation, primarily stabilized by van der Waals interactions. Although the complex underwent pronounced conformational transitions characterized by elevated RMSD, bimodal conformational sampling, extensive inter-domain correlated motions, and broad PCA distribution, the MD simulation supports the feasibility of the proposed binding mode. However, these findings represent computational evidence and should not be considered direct experimental confirmation of DNA gyrase inhibition. Therefore, experimental validation will be required to confirm the proposed antibacterial mechanism.

**Figure 19:**
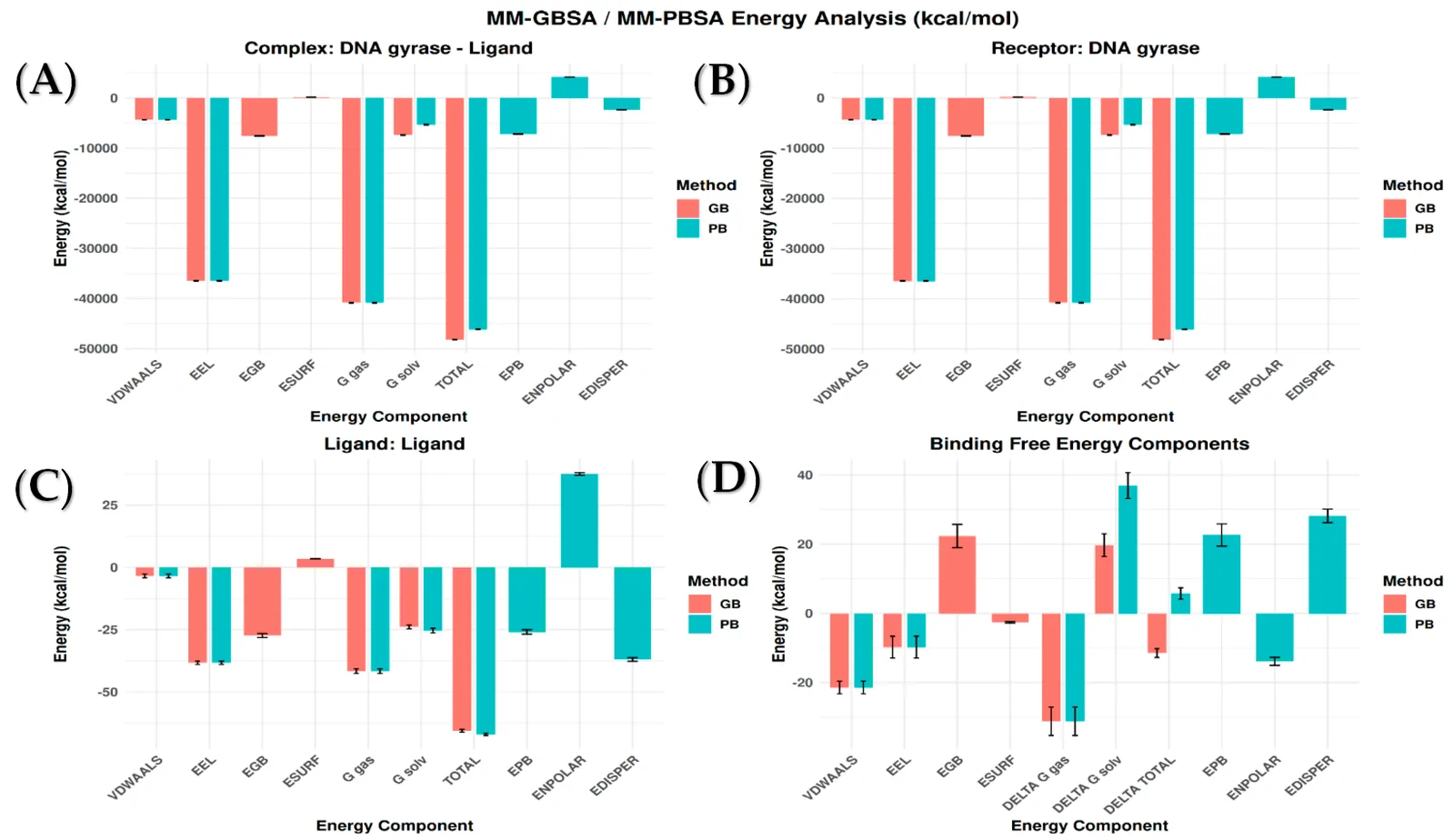
MM-GBSA/MM-PBSA free-energy decomposition for the *Staphylococcus aureus* DNA gyrase–compound **10** complex (PDB ID: 2XCT). The energy profiles of the protein–ligand complex (**A**), isolated receptor (**B**), free ligand (**C**), and the calculated binding free-energy components ((**D**); ΔG = Complex − Receptor − Ligand) are shown using the Generalized Born (GB) and Poisson–Boltzmann (PB) implicit solvent approaches. All energies are expressed in kcal/mol. Abbreviations: VDWAALS, van der Waals energy; EEL, electrostatic energy; EGB, Generalized Born polar solvation energy; EPB, Poisson–Boltzmann polar solvation energy; ESURF, nonpolar solvation energy (GB); ENPOLAR, nonpolar solvation energy (PB); EDISPER, dispersion energy; Ggas, gas-phase energy; Gsolv, solvation free energy; TOTAL, total free energy.

Figure 19 presents the MM-GBSA/MM-PBSA energy decomposition analysis for the protein–ligand complex, free receptor, free ligand, and the corresponding binding free energies. In both GB and PB models, the total interaction energy was dominated by favorable van der Waals and electrostatic contributions, whereas the polar solvation terms opposed complex formation. Nevertheless, the nonpolar contributions compensated for these unfavorable effects, resulting in an overall favorable binding free energy. The MM-GBSA binding free energy calculation yielded a favorable binding energy of −24.99 ± 2.71 kcal/mol, providing thermodynamic evidence for strong ligand binding. Collectively, the MD simulation and free-energy analyses support the formation of a stable and energetically favorable predicted complex between compound **10** and *Staphylococcus aureus* DNA gyrase. These computational findings further support DNA gyrase as a potential molecular target; however, direct experimental validation is required to confirm its involvement in the observed antibacterial activity.

#### 3.3.4. Quantum Chemical Calculations

**Figure 20:**
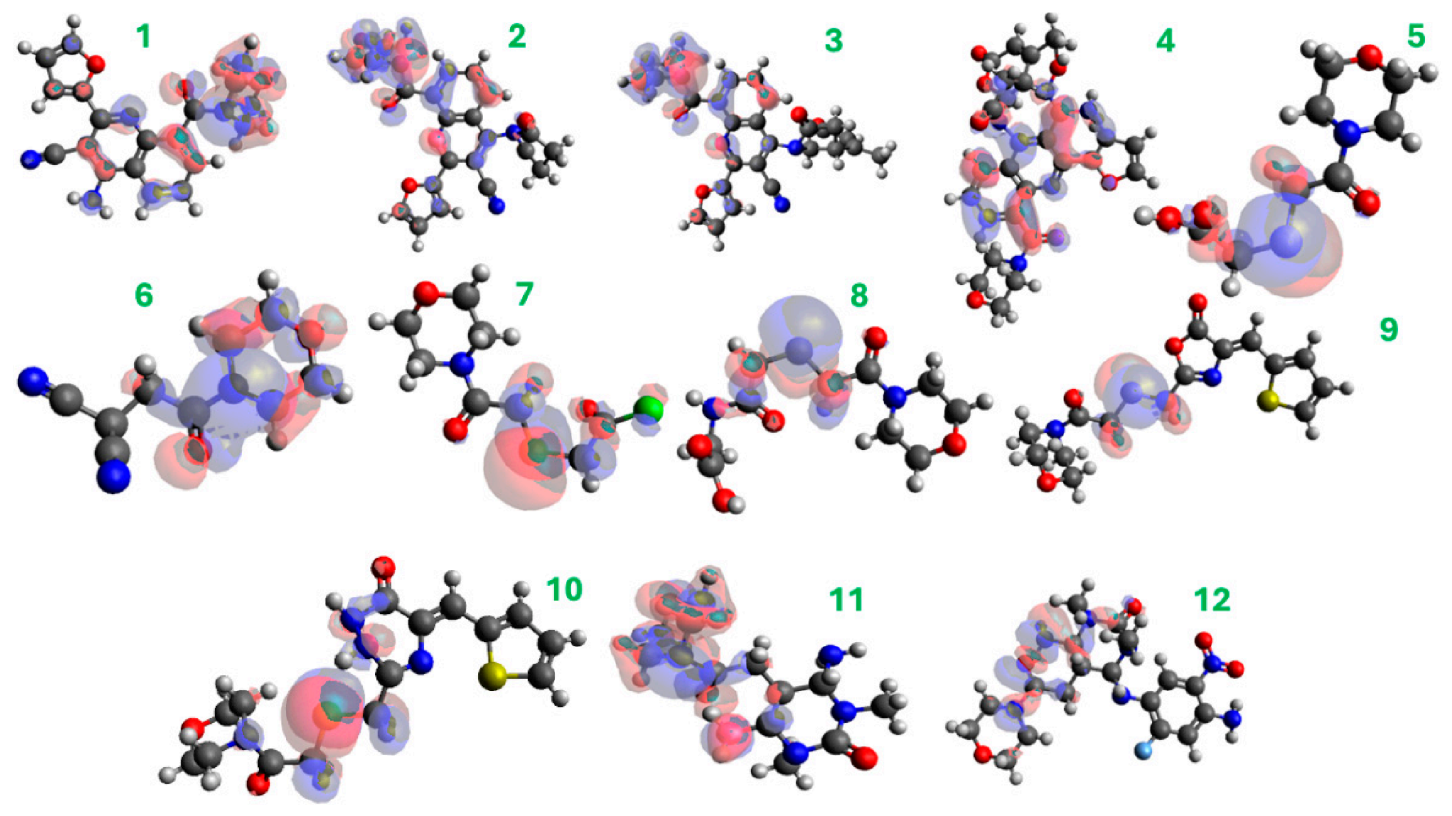
DFT-derived HOMO orbital surfaces of the synthesized morpholine-derived nitrogen-rich heterocyclic derivatives (**1**–**12**), highlighting the spatial distribution of the highest occupied molecular orbital and electron-rich regions associated with each molecular structure.

**Figure 21:**
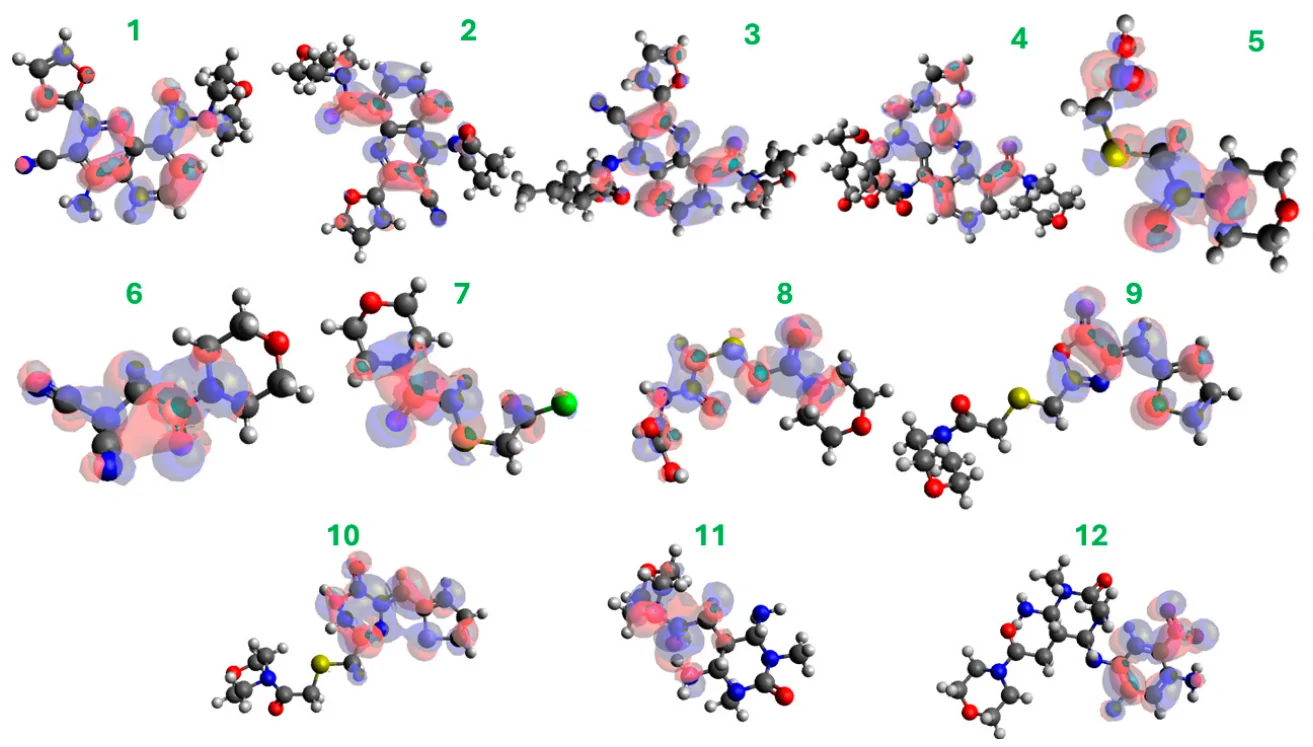
DFT-derived LUMO orbital surfaces of the synthesized morpholine-derived nitrogen-rich heterocyclic derivatives (**1**–**12**), showing the spatial localization of the lowest unoccupied molecular orbital and the preferred electron-accepting regions within each derivative.

To gain deeper insight into the electronic characteristics of the synthesized morpholine-based derivatives (**1**–**12**), density functional theory (DFT) calculations were carried out. Frontier molecular orbital (FMO) energies, global reactivity descriptors (Table 11), HOMO/LUMO orbital distributions (Figure 20 and Figure 21), optimized geometries, and molecular electrostatic potential (ESP) maps (Figure S52) were analyzed to establish correlations between the electronic features of the molecules and their chemical reactivity as well as their biological performance. The calculated HOMO–LUMO energy gaps (ΔE) varied considerably among the synthesized derivatives. Compound **4** exhibited the smallest energy gap (0.735 eV), together with the lowest hardness (η = 0.3675), highest softness (S = 2.721), electrophilicity (ω = 103.77), and electronic charge acceptance (ΔNmax = 8.40), indicating the highest electronic reactivity and charge transfer capability. Compounds **9** and **10** also showed favorable electronic characteristics with ΔE values of 1.477 and 1.661 eV, respectively, whereas compound **12** displayed intermediate behavior (ΔE = 1.995 eV). In contrast, compounds **5**–**8** and **11** were less reactive, while compound **6** exhibited the largest energy gap (4.953 eV), highest hardness (η = 2.4765), and lowest softness (S = 0.404), reflecting high kinetic stability and limited charge transfer ability. Accordingly, the electronic reactivity followed the order: **4** > **9** > **10** > **12** > **3** > **2** > **1** > **8** > **7** > **5** > **11** > **6**. The FMO distributions supported these results. Compounds **1**–**4**, particularly compound **4**, showed extensive HOMO and LUMO delocalization over the fused aromatic and heterocyclic framework with substantial orbital overlap, accounting for their enhanced charge transfer capability and reduced energy gaps. In contrast, compounds **5**–**11** exhibited more localized frontier orbitals, whereas compound **12** displayed intermediate orbital delocalization consistent with its moderate electronic behavior. The optimized geometries further demonstrated that compounds **1**–**4** possess rigid fused-ring π-conjugated structures favoring efficient electron delocalization, whereas compounds **5**–**10** adopt more flexible conformations due to the presence of aliphatic and sulfur-containing linkers. Compounds **11** and **12** exhibited relatively compact heteroatom-rich conformations that may facilitate intramolecular electronic communication. Consistent with these observations, the ESP maps revealed electron-rich regions localized around oxygen- and nitrogen-containing groups (carbonyl, nitrile, nitro, carboxyl, and morpholine moieties), while positive electrostatic potential was concentrated around amino, amide, and carboxylic acid hydrogens, identifying the principal hydrogen-bond acceptor and donor sites, respectively. Overall, the global reactivity descriptors, frontier orbital distributions, optimized geometries, and ESP maps provide valuable insight into the electronic behavior of the synthesized derivatives and complement the molecular docking and molecular dynamics studies. Although compound **4** exhibited the highest calculated electronic reactivity, the biological activity of the investigated compounds was not governed solely by their intrinsic electronic properties. Instead, the superior biological performance of compounds **3**, **10**, and **12** is likely the result of the combined influence of favorable electronic characteristics together with appropriate molecular geometry, pharmacophore arrangement, target binding interactions, and physicochemical properties governing ligand–target recognition. Therefore, the DFT calculations should be considered complementary to the experimental and computational findings, providing an electronic rationale that supports, rather than independently predicts, the observed biological activities.

##### Discussion of Advanced Quantum Chemical Analyses for Compound **3**

**Figure 22:**
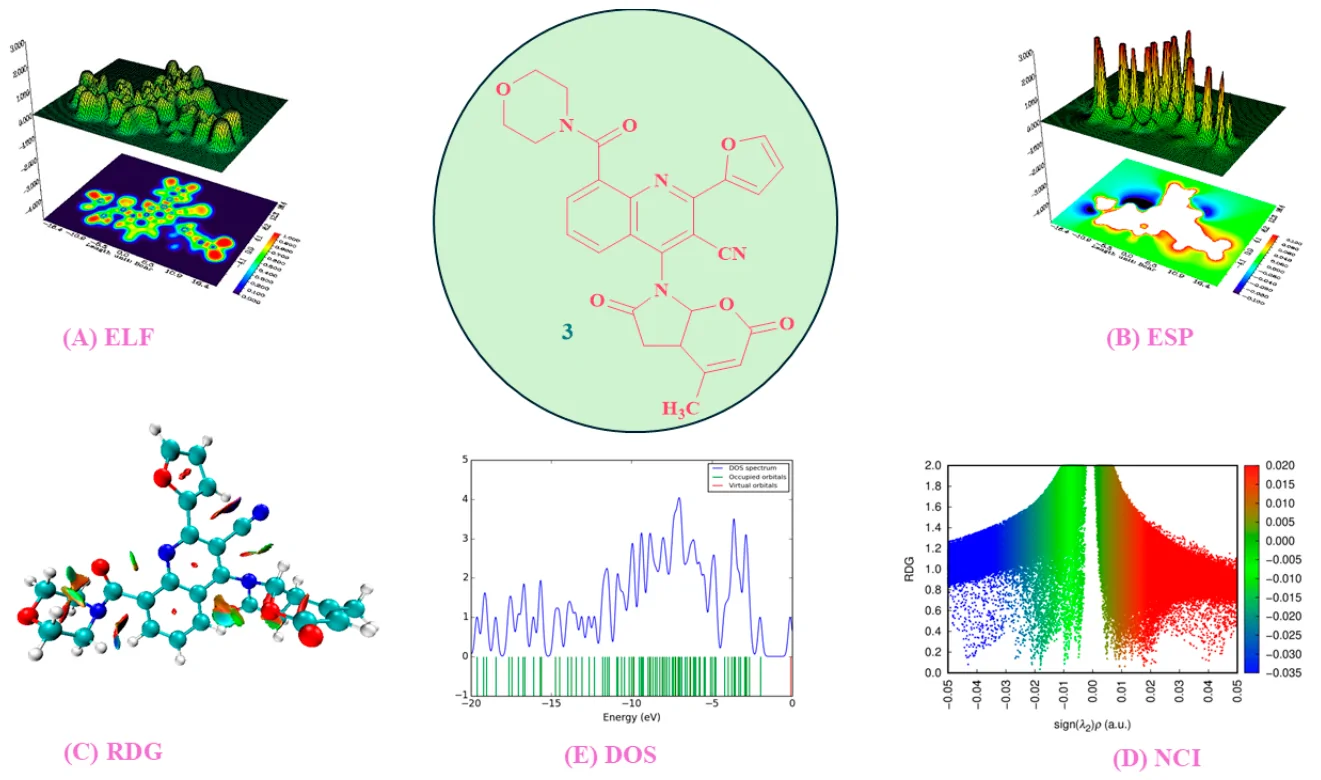
Advanced electronic structure analyses of compound **3** showing (**A**) ELF, (**B**) ESP, (**C**) RDG, (**D**) DOS, and (**E**) NCI results, highlighting the role of the quinoline–pyrrolone–pyranone framework in charge delocalization, electronic communication, and non-covalent interaction stabilization.

Advanced quantum chemical analyses of compound **3** revealed an extended π-conjugated system spanning the quinoline nucleus and the fused pyrrolone–pyranone framework (Figure 22). The ELF and ESP maps consistently identified the carbonyl oxygen atoms of the pyrrolone–pyranone moiety, the morpholine oxygen, the nitrile nitrogen, and the furan oxygen as the main electron-rich regions, whereas positive electrostatic potential was localized around hydrogen-bearing atoms. The fused pyrrolone–pyranone scaffold serves as an efficient electronic bridge between the quinoline core and the peripheral substituents, promoting charge delocalization across the molecule. RDG and NCI analyses indicated that weak attractive interactions and van der Waals contacts predominated with minimal steric repulsion, supporting the stability of the optimized structure. Moreover, the DOS spectrum exhibited a high density of electronic states near the frontier orbitals, consistent with efficient electronic communication within the conjugated framework. Collectively, these quantum chemical descriptors provide an electronic rationale for the favorable molecular recognition properties of compound **3**. The identified electron-rich regions, extensive charge delocalization, and predominance of stabilizing non-covalent interactions may facilitate productive interactions with biological targets, thereby complementing the molecular docking, molecular dynamics, and experimental biological findings. Accordingly, these analyses should be regarded as supportive computational evidence rather than independent predictors of biological activity.

##### Discussion of Advanced Quantum Chemical Analyses for Compound **10**

**Figure 23:**
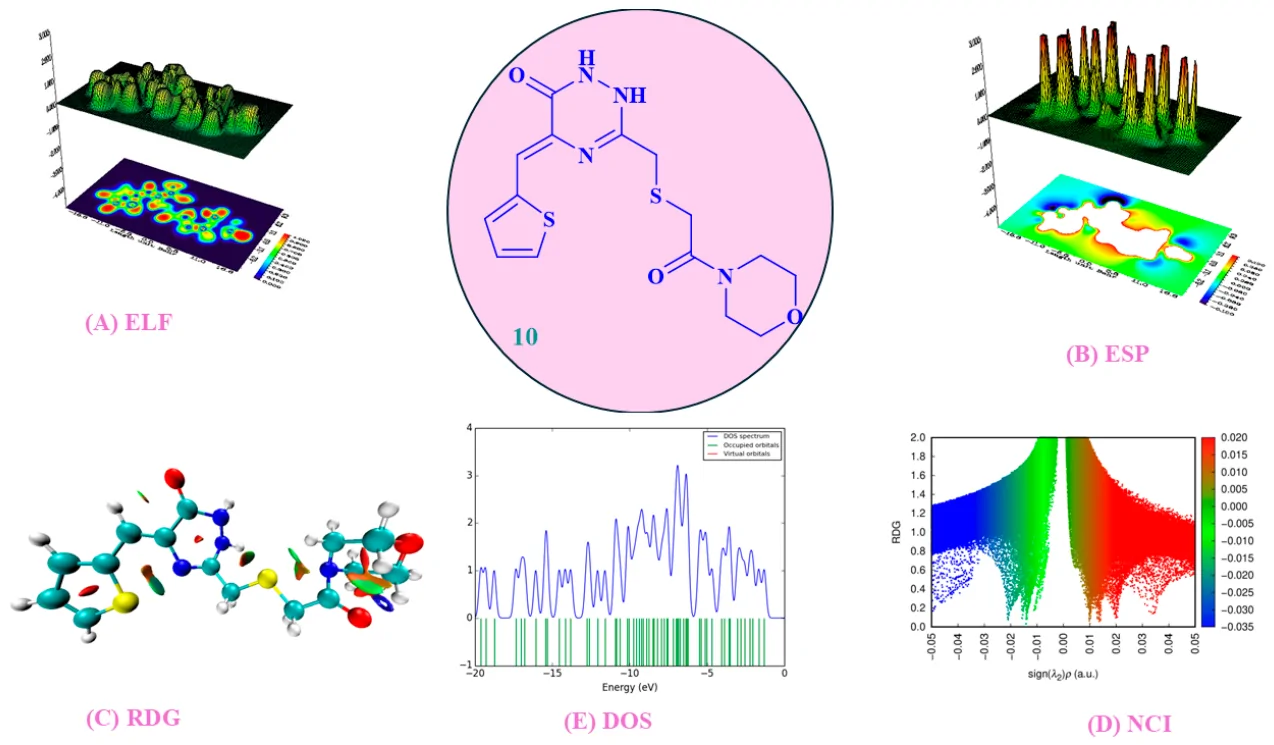
Advanced electronic structure analyses of compound **10** including (**A**) ELF, (**B**) ESP, (**C**) RDG, (**D**) DOS, and (**E**) NCI analyses, demonstrating the role of the thiophene–thioether–triazine framework in charge delocalization, electronic communication, and stabilization through non-covalent interactions.

Advanced quantum chemical analyses of compound **10** highlighted the key electronic role of the thiophene–thioether–triazinone framework (Figure 23). The ELF and ESP maps consistently identified the carbonyl oxygen, triazinone nitrogen atoms, morpholine oxygen, and sulfur-containing centers as the principal electron-rich regions, whereas positive electrostatic potential was mainly localized around the NH groups, establishing a clear donor–acceptor distribution. The sulfur linker provides an efficient electronic bridge between the electron-rich thiophene ring and the electron-deficient triazinone nucleus, facilitating charge delocalization throughout the molecule. RDG and NCI analyses revealed predominantly weak attractive interactions and van der Waals contacts with minimal steric repulsion, supporting the stability of the optimized structure. Likewise, the DOS spectrum showed a high density of electronic states near the frontier orbitals, indicating efficient charge transfer within the conjugated framework. Collectively, these quantum chemical descriptors provide an electronic rationale for the favorable molecular recognition properties of compound **10**. The identified electron-rich regions, efficient charge delocalization, and predominance of stabilizing non-covalent interactions may facilitate productive interactions with biological targets, thereby complementing the molecular docking, molecular dynamics, and experimental biological findings. Accordingly, these analyses should be regarded as supportive computational evidence that helps rationalize the superior biological performance of compound **10** rather than as independent predictors of biological activity.

##### Discussion of Advanced Quantum Chemical Analyses for Compound **12**

**Figure 24:**
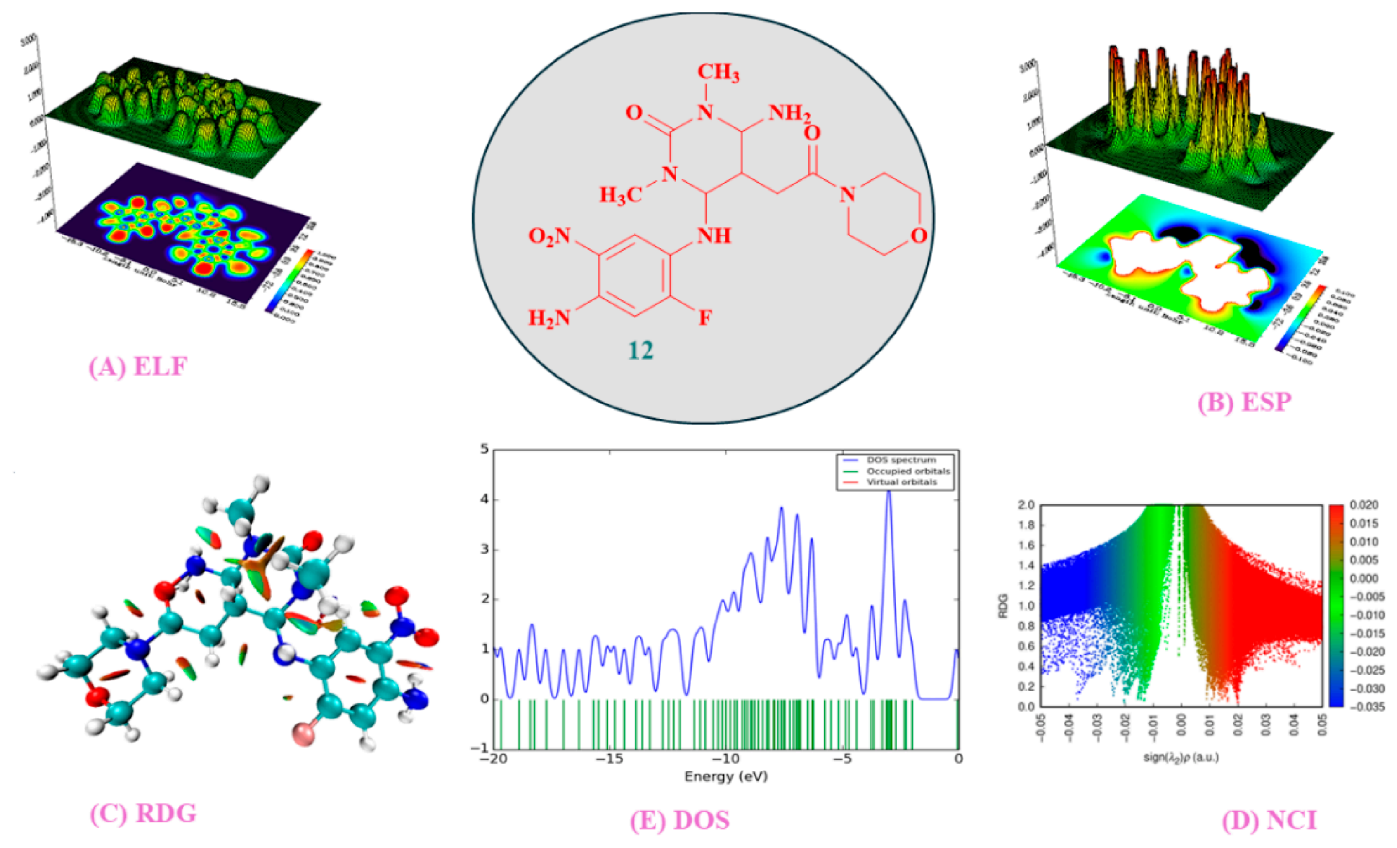
Advanced quantum chemical analyses of compound **12**, including (**A**) electron localization function (ELF), (**B**) molecular electrostatic potential (ESP), (**C**) reduced density gradient (RDG), (**D**) non-covalent interaction (NCI) analysis, and (**E**) density of states (DOS), illustrating electron localization, charge distribution, and intermolecular interaction characteristics.

Advanced quantum chemical analyses of compound **12** provided further insight into its electronic properties (Figure 24). The ELF and ESP maps consistently identified the carbonyl oxygen atoms of the pyrimidinedione ring, the nitro group, and the morpholine oxygen as the principal electron-rich regions, whereas positive electrostatic potential was mainly localized around the NH and NH_2_ groups, establishing a distinct donor–acceptor distribution. The pyrimidinedione nucleus functions as an efficient electronic bridge between the electron-donating amino groups and the electron-withdrawing nitro and carbonyl functionalities, promoting charge polarization across the molecule. RDG and NCI analyses revealed predominantly weak attractive interactions and van der Waals contacts with minimal steric repulsion, supporting the stability of the optimized structure. Likewise, the DOS spectrum showed a high density of electronic states near the frontier orbitals, indicating efficient electronic communication and charge transfer. Collectively, these quantum chemical descriptors provide an electronic rationale for the favorable molecular recognition properties of compound **12**. The distinct donor–acceptor distribution, efficient charge polarization, and predominance of stabilizing non-covalent interactions may facilitate productive interactions with biological targets, thereby complementing the molecular docking, molecular dynamics, and experimental biological findings. These electronic characteristics may also contribute to the enhanced cellular selectivity exhibited by compound **12** despite its comparatively weaker kinase inhibition, although additional experimental studies are required to confirm this hypothesis. Accordingly, these analyses should be regarded as supportive computational evidence that helps rationalize the observed biological behavior of compound **12** rather than as independent predictors of biological activity.

## 4. Conclusions

This work describes the successful development of a new collection of morpholine-based nitrogen-rich heterocyclic derivatives (**1**–**12**) containing pyran, triazinone, pyrimidinone, and sulfur-containing frameworks. Comprehensive spectroscopic characterization confirmed the proposed molecular structures, while biological screening highlighted compounds **3**, **10**, and **12** as the most active members against MCF-7 and HCT-116 cancer cells. Among them, compound **10** exhibited the best overall biological profile, combining potent inhibition of EGFR, PI3K, and mTOR with pronounced induction of cell-cycle arrest, ROS generation, mitochondrial membrane depolarization, and mitochondria-mediated apoptosis through upregulation of Bax, caspase-3, and caspase-9, together with suppression of Bcl-2 expression. These biological findings, together with the biochemical enzyme inhibition results, support the proposed involvement of the EGFR/PI3K/mTOR signaling pathway in its antiproliferative activity. Beyond its anticancer activity, compound **10** also demonstrated superior antibacterial, antibiofilm, and antioxidant properties, including greater antibacterial potency than ciprofloxacin against selected bacterial strains. Computational investigations consistently supported the experimental observations. Molecular docking, molecular dynamics simulations, and MM-GBSA/MM-PBSA analyses predicted stable binding of compound **10** with both EGFR and *Staphylococcus aureus* DNA gyrase, yielding favorable binding free energies of −23.44 and −24.99 ± 2.71 kcal/mol, respectively. In addition, DFT calculations together with FMO, ESP, ELF, RDG, NCI, and DOS analyses revealed electronic characteristics that were consistent with the enhanced biological performance of the most active derivatives, particularly compounds **10** and **12**. Overall, the present findings identify compound **10** as a promising multifunctional lead scaffold with multitarget anticancer and antibacterial potential. Nevertheless, the present study is limited to in vitro biological evaluation, antibacterial assays, and computational investigations. Furthermore, the selectivity profile was assessed using only a single normal cell line (WI-38), and therefore additional studies employing tissue-relevant non-malignant cell models are required to further validate the therapeutic selectivity and safety profile of the most active compounds. Moreover, direct validation of downstream EGFR/PI3K/AKT/mTOR signaling proteins and experimental confirmation of DNA gyrase inhibition were beyond the scope of the present study. Therefore, further investigations involving in vivo efficacy studies, pharmacokinetic profiling, comprehensive toxicity assessment, and experimental validation of the proposed molecular targets are warranted before considering preclinical development. The combined experimental and computational findings provide a solid foundation for the continued optimization and future development of this class of morpholine-derived nitrogen-rich heterocycles.

## Figures and Tables

**Table 1:** Cytotoxic activity (IC_50_, μM) and selectivity indices (SI) of the synthesized morpholine derivatives (**1**–**12**) against HCT-116 and MCF-7 cancer cells, with normal WI-38 fibroblasts included for selectivity assessment. Erlotinib served as the reference drug.

Comp.		In Vitro Cytotoxicity IC_50_ (µM)			
	WI-38	HCT-116	SI (HCT-116)	MCF-7	SI (MCF-7)
**Erlotinib**	10.65 ± 0.8	5.47 ± 0.3	1.95	7.26 ± 0.3	1.47
**1**	64.16 ± 3.5	42.81 ± 2.4	1.50	38.47 ± 2.3	1.67
**2**	>100	68.74 ± 3.6	˂1.45	74.35 ± 3.7	˂1.34
**3**	87.61 ± 3.4	6.15 ± 1.3	14.25	7.48 ± 1.4	11.71
**4**	83.57 ± 4.2	51.76 ± 3.0	1.61	45.69 ± 2.6	1.83
**5**	76.01 ± 3.7	13.51 ± 1.0	5.63	18.15 ± 1.3	4.19
**6**	60.33 ± 3.4	25.12 ± 1.6	2.40	31.76 ± 1.9	1.90
**7**	36.21 ± 2.2	>100	0.36	69.44 ± 3.5	0.52
**8**	55.24 ± 3.2	34.78 ± 2.2	1.59	11.57 ± 0.8	4.77
**9**	>100	89.42 ± 4.5	˃1.12	62.74 ± 3.4	˃1.59
**10**	47.42 ± 2.6	5.12 ± 0.5	9.26	6.26 ± 0.3	7.58
**11**	28.64 ± 1.9	66.25 ± 3.4	0.43	52.63 ± 3.0	0.54
**12**	82.48 ± 1.4	5.06 ± 0.3	16.30	6.14 ± 4.2	16.30

Values are presented as the mean ± SD from three independent experiments. IC_50_ denotes the concentration required to reduce cell viability by 50%. Erlotinib was included as the positive reference compound.

**Table 2:** Inhibitory potency (IC_50_, μM) of the most active morpholine derivatives (**3**, **10**, and **12**) toward EGFR, PI3K, and mTOR relative to the corresponding reference inhibitors.

Compound	EGFR IC_50_ (μM)	PI3K IC_50_ (μM)	mTOR IC_50_ (μM)
**3**	0.214 ± 0.008	0.326 ± 0.014	0.580 ± 0.019
**10**	0.086 ± 0.003	0.107 ± 0.005	0.223 ± 0.008
**12**	0.274 ± 0.013	0.420 ± 0.018	1.201 ± 0.057
**Reference Inhibitor**	**Erlotinib** (0.056 ± 0.002)	**Pictilisib** (0.071 ± 0.006)	**Rapamycin** (0.208 ± 0.009)

**Table 3:** Quantification of intracellular reactive oxygen species (ROS) in HCT-116 cells after exposure to compound **10**. Values are expressed as ROS concentration (pg/mL) and fold change relative to the untreated control.

Sample	ROS (pg/mL)	Fold Change
**Control HCT-116**	51.56 ± 2.4	1.00
**Compound 10-treated HCT-116**	185.94 ± 7.7	3.55

**Table 4:** Relative expression of apoptosis-associated genes in HCT-116 cells after exposure to compound **10**, determined by RT-PCR. Data are expressed as fold change compared with untreated control cells.

Sample	RT-PCR (Fold Change)			
	Casp-3	Casp-9	Bax	Bcl-2
**S10/HCT116**	9.7136	5.3889	6.8211	0.2238
**C/HCT116**	1	1	1	1

**Table 5:** Antibacterial activity of selected compounds (**12**, **5**, **3**, **10**, **8**, and **6**) evaluated by the agar-well diffusion assay.

Compound	Inhibition Zone (mm)			
	*Enterococcus faecalis* ATCC 29212	*Escherichia coli* ATCC 25915	*Pseudomonas aeruginosa* ATCC 10145	*Staphylococcus aureus* ATCC 25923
**12**	3.00 ± 0.09	4.00 ± 0.15	3.50 ± 0.15	ND
**5**	ND	3.00 ± 0.09	ND	ND
**3**	ND	5.00 ± 0.08	4.50 ± 0.10	4.50 ± 0.00
**10**	10.50 ± 0.20	14.55 ± 0.05	16.50 ± 0.09	11.00 ± 0.08
**8**	ND	4.50 ± 0.00	ND	ND
**6**	ND	ND	ND	4.00 ± 0.10
**Ciprofloxacin**	7.00 ± 0.11	9.50 ± 0.10	10.00 ± 0.10	8.00 ± 0.08

ND: No inhibition detected. Ciprofloxacin was used as the standard antibacterial agent at 20 μg/mL All experiments were performed in biological triplicate, and the results are presented as mean ± SE.

**Table 6:** MIC values (μg/mL) of compounds **12**, **3**, and **10** determined against the investigated bacterial strains.

Compound	Minimum inhibitory concentration (MIC, μg/mL)			
	*Enterococcus faecalis* ATCC 29212	*Escherichia coli* ATCC 25915	*Pseudomonas aeruginosa* ATCC 10145	*Staphylococcus aureus* ATCC 25923
**12**	20.00	20.00	20.00	40.00
**3**	80.00	20.00	20.00	20.00
**10**	20.00	10.00	5.00	10.00
**Ciprofloxacin**	20.00	20.00	10.00	20.00

**Table 7:** Antibiofilm inhibitory activity of the selected compounds (**12**, **3**, and **10**).

Compound	Antibiofilm Efficiency			
	*Enterococcus faecalis* ATCC 29212	*Escherichia coli* ATCC 25915	*Pseudomonas aeruginosa* ATCC 10145	*Staphylococcus aureus* ATCC 25923
**12**	ND	16.30 ± 0.10	15.30 ± 0.08	ND
**3**	ND	21.50 ± 0.08	18.50 ± 0.09	15.20 ± 0.10
**10**	27.50 ± 0.08	35.55 ± 0.07	42.80 ± 0.10	25.00 ± 0.08
***Ciprofloxacin***	28.00 ± 0.11	32.20 ± 0.00	36.50 ± 0.08	27.30 ± 0.10

ND: No inhibition detected. all experiments were performed as biological triplicate and assigned as Mean ± SE. Ciprofloxacin were used as standard antibacterial agents at 20 μg/mL.

**Table 8:** Antioxidant properties of compounds **12**, **5**, **3**, **10**, **8**, and **6** determined by DPPH and ABTS radical scavenging assays.

Compound	Antioxidant Activity (%)			
	DPPH Scavenging Activity %	IC_50_ (μg/mL)	ABTS Scavenging Activity %	IC_50_ (μg/mL)
**12**	15.30 ± 0.18	163.40 ± 0.12	6.21 ± 0.08	402.57 ± 0.10
**5**	21.65 ± 0.15	115.42 ± 0.11	6.30 ± 0.21	396.82 ± 0.00
**3**	22.12 ± 0.03	113.12 ± 0.09	8.25 ± 0.10	303.30 ± 0.10
**10**	27.11 ± 0.08	112.50 ± 0.12	16.30 ± 0.09	153.37 ± 0.08
**8**	10.30 ± 0.18	242.71 ± 0.09	7.30 ± 0.01	342.46 ± 0.15
**6**	14.50 ± 0.18	172.41 ± 0.10	5.21 ± 0.09	479.84 ± 0.10
**BHT (control)**	75.20 ± 0.10	33.24 ± 0.10	45.50 ± 0.32	54.94 ± 0.10

**Table 9:** Calculated physicochemical characteristics of the promising morpholino-2-oxoethyl-based derivatives **3**, **10**, and **12**.

Compd.	Violations ^a^	MW ^b^	nHBD ^c^	nHBA ^d^	nRB ^e^	TPSA (Å^2^) ^f^	MLogP ^g^
**Rule**	−	≤500	≤5	≤10	≤10	≤140	≤4.15
**3**	Lipinski & Veber (0 violations)	498.49	0	8	4	125.97	0.35
**10**	Lipinski (Yes, 0 violations) Veber (No, 1 violation, TPSA > 140)	366.46	2	4	6	144.62	0.34
**12**	Lipinski (Yes, 1 violation, NorO > 10) Veber (No, 1 violation, TPSA > 140)	439.44	3	7	6	162.98	−0.51

**a**, Lipinski’s Rule of Five and Veber’s rule violations; **b**, molecular weight (MW); **c**, number of hydrogen bond donors (HBD); **d**, number of hydrogen bond acceptors(HBA); **e**, number of rotatable bonds (RB); **f**, calculated octanol/water partition coefficient (MLogP); and **g**, topological polar surface area (TPSA).

**Table 10:** Anticipated ADMET profile of morpholino-2-oxoethyl-based derivatives **3**, **10**, and **12** using *pkCSM*.

Properties (Predicted Value)	Compound		
	3	10	12
**Absorption**			
BBB	NO	NO	NO
HIA	High	Low	Low
P-glycoprotein Substrate	YES	NO	YES
**Distribution**			
VDss (human)	−0.083	−0.442	−0.407
Fraction unbound (human)	0.102	0.334	0.405
BBB permeability	−1.194	−0.957	−2.143
**Metabolism**			
CYP2D6 substrate	No	Yes	No
CYP3A4 substrate	Yes	No	No
CYP1A2 inhibitor	No	No	No
CYP2C19 inhibitor	No	No	No
CYP2C9 inhibitor	Yes	No	No
CYP2D6 inhibitor	No	No	No
CYP3A4 inhibitor	Yes	Yes	No
**Excretion and Toxicity**			
Total Clearance	0.539	0.507	0.185
Renal OCT2 substrate	No	No	No
AMES toxicity	No	No	Yes
Max. tolerated dose (human)	−0.686	−0.287	0.283
hERG I inhibitor	No	No	No
hERG II inhibitor	Yes	No	Yes
Oral Rat Acute Toxicity (LD_50_)	2.86	2.983	2.58
Oral Rat Chronic Toxicity (LOAEL)	1.676	1.53	1.839
Hepatotoxicity	Yes	Yes	Yes
Skin Sensitization	No	No	No
*T. Pyriformis* toxicity	0.298	0.56	0.285
Minnow toxicity	2.05	2.088	4.928

olubility is classified by log S values: very soluble (>0), extremely soluble (−2 to 0), soluble (−4 to −2), moderately soluble (−6 to −4), weakly soluble (−10 to −6), and insoluble (<−10). High Caco-2 permeability is indicated by log Papp > 0.9; low skin permeability by log Kp > −2.5. Volume of distribution (VDss) is low if log VDss < −0.15 and high if log VDss > 0.45. Compounds with log BB > 0.3 readily cross the blood–brain barrier, while those with log BB < −1 are poorly distributed to the brain. Central nervous system (CNS) penetration is expected if log PS > −2, but unlikely if log PS < −3.

**Table 11:** Frontier molecular orbital (FMO) energies and global reactivity descriptors obtained from density functional theory (DFT) calculations for the synthesized morpholine-derived nitrogen-rich heterocyclic derivatives (**1**–**12**). The calculated descriptors include HOMO and LUMO energies, energy gap (ΔE), electronegativity (χ), chemical hardness (η), softness (S), electrophilicity index (ω), and maximum electronic charge transfer (ΔN_max).

No.	LUMO	HOMO	ΔE	A	I	X	η	S or σ	ω	ΔN max	ΔN
**1**	−6.064	−8.588	2.524	6.064	8.588	7.326	1.262	0.792393027	42.52795246	2.902535658	−0.129160063
**2**	−6.213	−8.673	2.46	6.213	8.673	7.443	1.23	0.81300813	45.03922683	3.025609756	−0.180081301
**3**	−6.156	−8.588	2.432	6.156	8.588	7.372	1.216	0.822368421	44.69275	3.03125	−0.152960526
**4**	−5.808	−6.543	0.735	5.808	6.543	6.1755	0.3675	2.721088435	103.7736061	8.402040816	1.121768707
**5**	−4.118	−7.215	3.097	4.118	7.215	5.6665	1.5485	0.645786245	20.73569406	1.829673878	0.430577979
**6**	−4.2	−9.153	4.953	4.2	9.153	6.6765	2.4765	0.403795679	17.99945578	1.347970927	0.065313951
**7**	−4.114	−7.2	3.086	4.114	7.2	5.657	1.543	0.64808814	20.73988918	1.833117304	0.435191186
**8**	−4.125	−7.215	3.09	4.125	7.215	5.67	1.545	0.647249191	20.80834951	1.834951456	0.430420712
**9**	−5.695	−7.172	1.477	5.695	7.172	6.4335	0.7385	1.354096141	56.04593399	4.355788761	0.383547732
**10**	−5.576	−7.237	1.661	5.576	7.237	6.4065	0.8305	1.204093919	49.41991842	3.857013847	0.357314871
**11**	−4.482	−8.813	4.331	4.482	8.813	6.6475	2.1655	0.461787116	20.40602921	1.534864927	0.081389979
**12**	−5.168	−7.163	1.995	5.168	7.163	6.1655	0.9975	1.002506266	38.1086619	3.09047619	0.418295739

Lower HOMO–LUMO energy gaps (ΔE) and hardness values (η), together with higher softness (S), electrophilicity index (ω), and electron transfer capacity (ΔN_max), are generally associated with increased molecular reactivity, enhanced electronic communication, and improved probability of productive interaction within biological active sites.

## Data Availability

All data supporting the findings of this study are available within the article and its Supplementary Information (SI).

## Supplementary Materials

The following supporting information can be downloaded at: https://www.mdpi.com/article/10.3390/pharmaceutics18080982/s1, The SI contains IR, 1H NMR, 13C NMR, and mass spectral data together with the structures of the synthesized compounds (Figures S1–S48), dose–responsedose-response viability curves for the tested compounds (Figure S49), two-dimensional interaction diagrams for the re-docked co-crystallized ligands used for docking protocol validation (Figures S50 and S51), optimized geometries and electrostatic potential surface (OPT/ESP) maps of the synthesized compounds (Figure S52), detailed experimental procedures, and supporting tables (Tables S1–S10).

## References

[1] GBD 2023 Cancer Collaborators (2025). The Global, Regional, and National Burden of Cancer, 1990–2023, with Forecasts to 2050: A Systematic Analysis for the Global Burden of Disease Study 2023. Lancet.

[2] Bray F., Laversanne M., Sung H., Ferlay J., Siegel R.L., Soerjomataram I., Jemal A. (2024). Global Cancer Statistics 2022: GLOBOCAN Estimates of Incidence and Mortality Worldwide for 36 Cancers in 185 Countries. CA Cancer J. Clin..

[3] Al-Karmalawy A.A., Eissa M.E., Ashour N.A., Yousef T.A., Al Khatib A.O., Hawas S.S. (2025). Medicinal Chemistry Perspectives on Anticancer Drug Design Based on Clinical Applications (2015–2025). RSC Adv..

[4] Zafar A., Khatoon S., Khan M.J., Abu J., Naeem A. (2025). Advancements and Limitations in Traditional Anti-Cancer Therapies: A Comprehensive Review of Surgery, Chemotherapy, Radiation Therapy, and Hormonal Therapy. Discov. Oncol..

[5] Luo Q., Smith D. (2025). Global Cancer Burden: Progress, Projections, and Challenges. Lancet.

[6] Hu Z., Bousbaa H. (2025). Recent Advances in Anticancer Strategies. Cancers.

[7] Freihat O., Sipos D., Kovacs A. (2025). Global Burden and Projections of Breast Cancer Incidence and Mortality to 2050: A Comprehensive Analysis of GLOBOCAN Data. Front. Public Health.

[8] Saha S., Mahapatra S., Khanra S., Mishra B., Swain B., Malhotra D., Saha S., Panda V.K., Kumari K., Jena S. (2025). Decoding Breast Cancer Treatment Resistance Through Genetic, Epigenetic, and Immune-Regulatory Mechanisms: From Molecular Insights to Translational Perspectives. Cancer Drug Resist..

[9] Zhang T., Guo Y., Qiu B., Dai X., Wang Y., Cao X. (2025). Global, Regional, and National Trends in Colorectal Cancer Burden from 1990 to 2021 and Projections to 2040. Front. Oncol..

[10] Al-Jaber H., Biswas K.H., Al-Mansoori L. (2025). Advancing Targeted Therapy for Colorectal Cancer: Harnessing Ligand-Directed Enzyme Prodrug Therapy for Highly Specific Interventions. Front. Oncol..

[11] Li J., Hu J., Yang Y., Zhang H., Liu Y., Fang Y., Qu L., Lin A., Luo P., Jiang A. (2025). Drug Resistance in Cancer: Molecular Mechanisms and Emerging Treatment Strategies. Mol. Biomed..

[12] Mengistu B.A., Tsegaw T., Demessie Y., Getnet K., Belete Bitew A., Kinde M.Z., Beirhun A.M., Mebratu A.S., Mekasha Y.T., Feleke M.G. (2024). Comprehensive Review of Drug Resistance in Mammalian Cancer Stem Cells: Implications for Cancer Therapy. Cancer Cell Int..

[13] Juthani R., Punatar S., Mittra I. (2024). New Light on Chemotherapy Toxicity and Its Prevention. BJC Rep..

[14] Fotie J. (2024). Editorial for the Special Issue “Advances in the Development of Anticancer Drugs”. Int. J. Mol. Sci..

[15] Yip H.Y.K., Papa A. (2021). Signaling Pathways in Cancer: Therapeutic Targets, Combinatorial Treatments, and New Developments. Cells.

[16] Halder S., Basu S., Lall S.P., Ganti A.K., Batra S.K., Seshacharyulu P. (2023). Targeting the EGFR Signaling Pathway in Cancer Therapy: What’s New in 2023?. Expert Opin. Ther. Targets.

[17] Seshacharyulu P., Ponnusamy M.P., Haridas D., Jain M., Ganti A.K., Batra S.K. (2012). Targeting the EGFR Signaling Pathway in Cancer Therapy. Expert Opin. Ther. Targets.

[18] Du Y., Karatekin F., Wang W.K., Hong W., Boopathy G.T. (2025). Cracking the EGFR Code: Cancer Biology, Resistance Mechanisms, and Future Therapeutic Frontiers. Pharmacol. Rev..

[19] Akinleye A., Avvaru P., Furqan M., Song Y., Liu D. (2013). Phosphatidylinositol 3-Kinase (PI3K) Inhibitors as Cancer Therapeutics. J. Hematol. Oncol..

[20] Hossain M.T., Hossain M.A. (2025). Targeting PI3K in Cancer Treatment: A Comprehensive Review with Insights from Clinical Outcomes. Eur. J. Pharmacol..

[21] Panwar V., Singh A., Bhatt M., Tonk R.K., Azizov S., Raza A.S., Sengupta S., Kumar D., Garg M. (2023). Multifaceted Role of mTOR (Mammalian Target of Rapamycin) Signaling Pathway in Human Health and Disease. Signal Transduct. Target. Ther..

[22] Xie J., Wang X., Proud C.G. (2016). mTOR Inhibitors in Cancer Therapy. F1000Research.

[23] Dufour M., Dormond-Meuwly A., Demartines N., Dormond O. (2011). Targeting the Mammalian Target of Rapamycin (mTOR) in Cancer Therapy: Lessons from Past and Future Perspectives. Cancers.

[24] Liao J., Yang Z., Azarbarzin S., Cullen K.J., Dan H. (2024). Differential Modulation of PI3K/Akt/mTOR Activity by EGFR Inhibitors: A Rationale for Co-Targeting EGFR and PI3K in Cisplatin-Resistant HNSCC. Head Neck.

[25] El-Hema H.S., Mady M.F., Abdel-Rahman A.A.-H., Nossier E.S., El-Sayed A.F., Sabry A., El-Fekey M., Hawata M.A. (2026). Novel Pyrimidopyridothiazine-Based Derivatives as Potential Anticancer and Antibacterial Agents: A Step towards Design and Synthesis of Multi-Targeted Therapeutics. J. Mol. Struct..

[26] El-Hema H.S., Shehata H.E., Hawata M.A., Nossier E.S., El-Sayed A.F., Altwaijry N.A., Saleh A., Hussein M.F., Sabry A., Abdel-Rahman A.A.-H. (2025). Innovative Amino-Functionalization of Pyrido[2,3-*d*]Pyrimidine Scaffolds for Broad Therapeutic Applications Supported by Computational Analyses. Pharmaceuticals.

[27] El-Hema H.S., Soliman S.M., El-Dougdoug W., Ahmed M.H.M., Abdelmajeid A., Nossier E.S., Hussein M.F., Alrayes A., Hassan M., Ahmed N.A. (2025). Design, Characterization, Antimicrobial Activity, and In Silico Studies of Theinothiazoloquinazoline Derivatives Bearing Thiazinone, Tetrazole, and Triazole Moieties. ACS Omega.

[28] El-Hema H.S., El-Shazly H.A., Hawata M.A., Yousif N.M., Hussein M.F., Said M.A., Aidy E.A., Shehata H.E., Abdel-Rahman A.A.-H. (2026). Expanding the Chemical and Therapeutic Landscape of 5H-Indeno[1,2-*b*]Pyridin-5-One Derivatives: Novel Anticancer Activity, EGFR Inhibition, and Modulation of HIF–VEGF and PI3K/AKT/mTOR Pathways Supported by Computational Insights. Bioorganic Chem..

[29] El-Hema H.S., El Fekey H.M., Hawata M.A., Hussein M.F., Elhendawy A.T., Abdel-Rahman A.A.H. (2026). From Benzo[*g*]quinoline Multitargeted Anticancer Scaffold to a Potent Benzo[*g*]Pyrimido[4,5-*b*]quinoline Lead Targeting Wild-Type and Mutant EGFR: Insights from Integrated Computational Studies, Molecular Signaling Modulation, and Gene Expression Networks. J. Mol. Struct..

[30] Hussein M.F., Said M.A., Abuessawy A., Alsahli S.A., Al-Sirhani A.M., Manni E., El-Hema H.S. (2026). Thienyl-Based Pyrimidine Hybrids as Multi-Target Anticancer Agents: Design, Synthesis, and Integrated Experimental-Computational Insights into CDK4 Inhibition and Apoptosis. J. Mol. Struct..

[31] El-Hema H.S., El-Fekey H.M., Abdel-Rahman A.A.-H., Morsy A.R.I., Radwan A.A., Nossier E.S., Alshabani L.A., Saleh A., Hussein M.F., Hawata M.A. (2026). Design and Multi-Level Biological Evaluation of Naphthyridine-Based Derivatives as Topoisomerase I/II-Targeted Anticancer Agents with Anti-Fowlpox Virus Activity Supported by In Silico Analysis. Int. J. Mol. Sci..

[32] El-Hema H.S., Abed M.A., Hawata M.A., Nossier E.S., Altwaijry N.A., Saleh A., Hassan M., Hashem R.A., Hussein M.F., Abdel-Rahman A.A.-H. (2026). A Multi-Target Nitrogen-Fused Azole Drug Platform Derived from a Pyrazoline-Thiadiazole Moiety: In Vivo Antimicrobial Validation and Comprehensive Anticancer Investigation Supported by Computational Studies. Pharmaceutics.

[33] El-Hema H.S., Ahmed A., Waheed A., Hawata M.A., Hussein M.F., Khalifa M.M., Elhendawy A.T., El-Sayed H.A., Abdel-Rahman A.A.-H. (2026). Hybridization-Driven Design of Triazole-Imidazol-4(5H)-One Conjugates as Multi-Target Cholinesterase and Inflammatory Enzyme Inhibitors: Synthesis, Biological Evaluation, and Comprehensive Computational Studies. J. Mol. Struct..

[34] El-Hema H.S., El-Ghorab R.A.-S., Hawata M.A., Nossier E.S., Hussein M.F., Elhendawy A.T., Hamza M.F., Abdel-Rahman A.A.-H. (2026). Rationally Designed Quinazolinone Derivatives Incorporating Acetyl Pyridine and Thiazole Scaffolds as Dual EGFR/HER-2 Anticancer Agents: Integrated Biological Evaluation, Molecular Profiling, and Advanced Computational Studies. Bioorganic Chem..

[35] Kourounakis A.P., Xanthopoulos D., Tzara A. (2020). Morpholine as a Privileged Structure: A Review on the Medicinal Chemistry and Pharmacological Activity of Morpholine-Containing Bioactive Molecules. Med. Res. Rev..

[36] Manni E., Hussein M.F., Elkady S., Abdel-Rahman A.A.-H., Hawata M.A., El-Sayed W.A., El-Sayed A.F., El-Hema H.S. (2026). From Synthesis to Mechanism: Biological Evaluation of *p*-Toluidine-Based Thiazolidinone-Quinoline VEGFR-2 Candidate Supported by CADD. Int. J. Mol. Sci..

[37] Al-Wahaibi L.H., Mohassab A.M., Rabea S.M., Youssif B.G.M., Bräse S., El-Sheref E.M. (2025). Design, Synthesis, and Antiproliferative Activity of New 2-Amino-Pyrano[3,2-*c*]Quinoline-3-Carbonitriles as Potential EGFR, BRAFV600E, and HER-2 Inhibitors. RSC Adv..

[38] Kaplan J., Verheijen J.C., Brooijmans N., Toral-Barza L., Hollander I., Yu K., Zask A. (2010). Discovery of 3,6-Dihydro-2H-Pyran as a Morpholine Replacement in 6-Aryl-1H-Pyrazolo[3,4-*d*]Pyrimidines and 2-Arylthieno[3,2-*d*]Pyrimidines: ATP-Competitive Inhibitors of the Mammalian Target of Rapamycin (mTOR). Bioorganic Med. Chem. Lett..

[39] De Pascale M., Bissegger L., Tarantelli C., Beaufils F., Prescimone A., Hedad H.M.S., Kayali O., Orbegoso C., Raguž L., Schaefer T. (2023). Investigation of Morpholine Isosters for the Development of a Potent, Selective, and Metabolically Stable mTOR Kinase Inhibitor. Eur. J. Med. Chem..

[40] Vlahos C.J., Matter W.F., Hui K.Y., Brown R.F. (1994). A Specific Inhibitor of Phosphatidylinositol 3-Kinase, 2-(4-Morpholinyl)-8-Phenyl-4H-1-Benzopyran-4-One (LY294002). J. Biol. Chem..

[41] Morales G.A., Garlich J.R., Su J., Peng X., Newblom J., Weber K., Durden D.L. (2013). Synthesis and Cancer Stem Cell-Based Activity of Substituted 5-Morpholino-7H-Thieno[3,2-*b*]Pyran-7-Ones Designed as Next-Generation PI3K Inhibitors. J. Med. Chem..

[42] Shawish I., Barakat A., Aldalbahi A., Malebari A.M., Nafie M.S., Bekhit A.A., Albohy A., Khan A., Ul-Haq Z., Haukka M. (2022). Synthesis and Antiproliferative Activity of a New Series of Mono- and Bis(Dimethylpyrazolyl)-*s*-Triazine Derivatives Targeting EGFR/PI3K/AKT/mTOR Signaling Cascades. ACS Omega.

[43] Shawish I., Barakat A., Aldalbahi A., Alshaer W., Daoud F., Alqudah D.A., Al Zoubi M., Hatmal M.M., Nafie M.S., Haukka M. (2022). Acetic Acid Mediated One-Pot Synthesis of Novel Pyrazolyl s-Triazine Derivatives for the Targeted Therapy of Triple-Negative Breast Tumor Cells (MDA-MB-231) *via* EGFR/PI3K/AKT/mTOR Signaling Cascades. Pharmaceutics.

[44] Ali M.I., Naseer M.M. (2023). Recent Biological Applications of Heterocyclic Hybrids Containing *s*-Triazine Scaffold. RSC Adv..

[45] Soliman S.M., Abdel-Rahman A.A.-H., Nossier E.S., Hussein M.F., Sabry A., El-Hema H.S. (2025). Design, Antiproliferative Potency, and In Silico Studies of Novel ((5-Methylfuran-3-yl)thio)-3-Phenylquinazolin-4(3H)-One-Based Derivatives as Potential EGFR Inhibitors. Sci. Rep..

[46] Șandor A., Ionuț I., Marc G., Oniga I., Eniu D., Oniga O. (2023). Structure–Activity Relationship Studies Based on Quinazoline Derivatives as EGFR Kinase Inhibitors (2017–Present). Pharmaceuticals.

[47] Huang D., Yang J., Zhang Q., Zhou X., Wang Y., Shang Z., Li J., Zhang B. (2024). Design, Synthesis, and Biological Evaluation of 2,4-Dimorpholinopyrimidine-5-Carbonitrile Derivatives as Orally Bioavailable PI3K Inhibitors. Front. Pharmacol..

[48] Rady G.S., El Deeb M.A., Sarg M.T., Taher A.T., Helwa A.A. (2024). Design, Synthesis, and Biological Evaluation of Novel Morpholinopyrimidine-5-Carbonitrile Derivatives as Dual PI3K/mTOR Inhibitors. RSC Med. Chem..

[49] Abdou M.M., Eliwa E.M., Abdel Reheim M.A.M., Abu-Rayyan A., Abd El-Gilil S.M., Abu-Elghait M., Sharaf M.H., Kalaba M.H., Halawa A.H., Elgammal W.E. (2024). Tailoring of Novel Morpholine-Sulphonamide-Linked Thiazole Moieties as Dual-Targeting DHFR/DNA Gyrase Inhibitors: Synthesis, Antimicrobial and Antibiofilm Activities, and DFT with Molecular Modelling Studies. New J. Chem..

[50] Almaghrabi M., Musa A., Aljohani A.K.B., Ahmed H.E.A., Alsulaimany M., Miski S.F. (2024). Introducing a Novel Class of Pyrano[2,3-*c*]Pyrazole-5-Carbonitrile Analogs with Potent Antimicrobial Activity, DNA Gyrase Inhibition, and Prominent Pharmacokinetic and CNS Toxicity Profiles Supported by Molecular Dynamic Simulation. J. Biomol. Struct. Dyn..

[51] Masih A., Shrivastava J.K., Bhat H.R., Singh U.P. (2020). Potent Antibacterial Activity of Dihydropyrimidine-1,3,5-Triazines *via* Inhibition of DNA Gyrase and Antifungal Activity with Favourable Metabolic Profile. Chem. Biol. Drug Des..

[52] Khalil H.H., Khattab S.N., Toughan M.M., El-Saghier A.M., El-Wakil M.H. (2020). Identification of a Novel DNA Gyrase Inhibitor *via* Design and Synthesis of New Antibacterial Pyrido[1′,2′:1,2]Pyrimido [4,5-*e*][1,3,4]Thiadiazin-5-ol Derivatives. ChemistrySelect.

[53] Kazandjian D., Blumenthal G.M., Yuan W., He K., Keegan P., Pazdur R. (2016). FDA Approval of Gefitinib for the Treatment of Patients with Metastatic EGFR Mutation-Positive Non-Small Cell Lung Cancer. Clin. Cancer Res..

[54] Mensah F.A., Blaize J.P., Bryan L.J. (2018). Spotlight on Copanlisib and Its Potential in the Treatment of Relapsed/Refractory Follicular Lymphoma: Evidence to Date. Onco Targets Ther..

[55] Morgan H., Lipka-Lloyd M., Warren A.J., Hughes N., Holmes J., Burton N.P., Mahenthiralingam E., Bax B.D. (2023). A 2.8 Å Structure of Zoliflodacin in a DNA Cleavage Complex with *Staphylococcus aureus* DNA Gyrase. Int. J. Mol. Sci..

[56] Soule H.D., Vazquez J., Long A., Albert S., Brennan M.J. (1973). A Human Cell Line from a Pleural Effusion Derived from a Breast Carcinoma. J. Natl. Cancer Inst..

[57] Brattain M.G., Fine W.D., Khaled F.M., Thompson J., Brattain D.E. (1981). Heterogeneity of Malignant Cells from a Human Colonic Carcinoma. Cancer Res..

[58] Mosmann T. (1983). Rapid Colorimetric Assay for Cellular Growth and Survival: Application to Proliferation and Cytotoxicity Assays. J. Immunol. Methods.

[59] Hayflick L., Moorhead P.S. (1961). The Serial Cultivation of Human Diploid Cell Strains. Exp. Cell Res..

[60] Ushiro H., Cohen S. (1980). Identification of Phosphotyrosine as a Product of Epidermal Growth Factor-Activated Protein Kinase in A-431 Cell Membranes. J. Biol. Chem..

[61] Whitman M., Downes C.P., Keeler M., Keller T., Cantley L. (1988). Type I Phosphatidylinositol Kinase Makes a Novel Inositol Phospholipid, Phosphatidylinositol-3-Phosphate. Nature.

[62] Sabatini D.M., Erdjument-Bromage H., Lui M., Tempst P., Snyder S.H. (1994). RAFT1: A Mammalian Protein That Binds to FKBP12 in a Rapamycin-Dependent Fashion and Is Homologous to Yeast TORs. Cell.

[63] Moyer J.D., Barbacci E.G., Iwata K.K., Arnold L., Boman B., Cunningham A., DiOrio C., Doty J., Morin M.J., Moyer P. (1997). Induction of Apoptosis and Cell Cycle Arrest by CP-358,774, an Inhibitor of Epidermal Growth Factor Receptor Tyrosine Kinase. Cancer Res..

[64] Folkes A.J., Ahmadi K., Alderton W.K., Alix S., Baker S.J., Box G., Chuckowree I.S., Clarke P.A., Depledge P., Eccles S.A. (2008). The Identification of 2-(1H-Indazol-4-yl)-6-(4-Methanesulfonyl-Piperazin-1-ylmethyl)-4-Morpholin-4-yl-Thieno[3,2-*d*]Pyrimidine (GDC-0941) as a Potent, Selective, Orally Bioavailable Inhibitor of Class I PI3 Kinase for the Treatment of Cancer. J. Med. Chem..

[65] Vézina C., Kudelski A., Sehgal S.N. (1975). Rapamycin (AY-22,989), a New Antifungal Antibiotic. I. Taxonomy of the Producing Streptomycete and Isolation of the Active Principle. J. Antibiot..

[66] Krishan A. (1975). Rapid Flow Cytofluorometric Analysis of Mammalian Cell Cycle by Propidium Iodide Staining. J. Cell Biol..

[67] Koopman G., Reutelingsperger C.P.M., Kuijten G.A.M., Keehnen R.M.J., Pals S.T., van Oers M.H.J. (1994). Annexin V for Flow Cytometric Detection of Phosphatidylserine Expression on B Cells Undergoing Apoptosis. Blood.

[68] Vermes I., Haanen C., Steffens-Nakken H., Reutelingsperger C. (1995). A Novel Assay for Apoptosis: Flow Cytometric Detection of Phosphatidylserine Expression on Early Apoptotic Cells Using Fluorescein-Labelled Annexin V. J. Immunol. Methods.

[69] Maglothin J.A., Wilson I.B. (1974). Equilibrium Constants for the Phosphorylation of Acetylcholinesterase by Some Diethyl Phosphorothiolates and Phosphates. Biochemistry.

[70] Novo D., Perlmutter N.G., Hunt R.H., Shapiro H.M. (1999). Accurate Flow Cytometric Membrane Potential Measurement in Bacteria Using Diethyloxacarbocyanine and a Ratiometric Technique. Cytometry.

[71] Novo D.J., Perlmutter N.G., Hunt R.H., Shapiro H.M. (2000). Multiparameter Flow Cytometric Analysis of Antibiotic Effects on Membrane Potential, Membrane Permeability, and Bacterial Counts of *Staphylococcus aureus* and *Micrococcus luteus*. Antimicrob. Agents Chemother..

[72] Bass D.A., Parce J.W., DeChatelet L.R., Szejda P., Seeds M.C., Thomas M. (1983). Flow Cytometric Studies of Oxidative Product Formation by Neutrophils: A Graded Response to Membrane Stimulation. J. Immunol..

[73] LeBel C.P., Ischiropoulos H., Bondy S.C. (1992). Evaluation of the Probe 2′,7′-Dichlorofluorescin as an Indicator of Reactive Oxygen Species Formation and Oxidative Stress. Chem. Res. Toxicol..

[74] Eruslanov E., Kusmartsev S. (2009). Identification of ROS Using Oxidized DCFDA and Flow Cytometry. Advanced Protocols in Oxidative Stress II.

[75] Livak K.J., Schmittgen T.D. (2001). Analysis of Relative Gene Expression Data Using Real-Time Quantitative PCR and the 2^−ΔΔCT^ Method. Methods.

[76] Schmittgen T.D., Livak K.J. (2008). Analyzing Real-Time PCR Data by the Comparative CT Method. Nat. Protoc..

[77] Higuchi R., Fockler C., Dollinger G., Watson R. (1993). Kinetic PCR Analysis: Real-Time Monitoring of DNA Amplification Reactions. Nat. Biotechnol..

[78] Sroor F.M., El-Sayed A.F., Abdelraof M. (2024). Design, Synthesis, Structure Elucidation, Antimicrobial, Molecular Docking, and SAR Studies of Novel Urea Derivatives Bearing Tricyclic Aromatic Hydrocarbon Rings. Arch. Pharm..

[79] Sroor F.M., Younis A., Abdelraof M., Abdelhamid I.A. (2025). Synthesis, Molecular Docking, and Anti-Biofilm Activity of Novel Benzo[4,5]Imidazo[2,1-*a*]Quinazoline, 4H-Chromene, and Acridine Derivatives as Potent Anti-*Candida* Agents. J. Mol. Struct..

[80] Khidre R.E., Sabry E., El-Sayed A.F., Sediek A.A. (2023). Design, One-Pot Synthesis, In Silico ADMET Prediction and Molecular Docking of Novel Triazolyl Thiadiazine and Thiazole Derivatives with Evaluation of Antimicrobial, Antioxidant, and Antibiofilm Inhibition Activity. J. Iran. Chem. Soc..

[81] Daina A., Michielin O., Zoete V. (2017). SwissADME: A Free Web Tool to Evaluate Pharmacokinetics, Drug-Likeness and Medicinal Chemistry Friendliness of Small Molecules. Sci. Rep..

[82] Cheng F., Li W., Zhou Y., Shen J., Wu Z., Liu G., Tang Y. (2012). admetSAR: A Comprehensive Source and Free Tool for Assessment of Chemical ADMET Properties. J. Chem. Inf. Model..

[83] Alamshany Z.M., Tashkandi N.Y., Othman I.M.M., Anwar M.M., Nossier E.S. (2026). Development of Benzothiazole-Grafted Heterocyclic Ring Systems as New Antiproliferative Hybrids Inducing Cell Cycle Arrest and Apoptosis in Triple-Negative Breast Cancer. J. Mol. Struct..

[84] Alsfouk A.A., Othman I.M., Anwar M.M., Saleh A., Tashkandi N.Y., Nossier E.S. (2025). New Quinazolinone-Based Heterocyclic Compounds as Promising Antimicrobial Agents: Development, DNA Gyrase B/Topoisomerase IV Inhibition Activity, and In Silico Computational Studies. J. Mol. Struct..

[85] Altwaijry N.A., Othman I.M., Anwar M.M., Nosier S.S., Saleh A., Tashkandi N.Y., Nossier E.S. (2026). Development, Anti-Proliferative Activity, Multi-Target Kinase Inhibition Against CHK1, PIM1, and CDK-2, and Computational Insights of New Thiazole-Based Hybrids. RSC Adv..

[86] Alamshany Z.M., Tashkandi N.Y., Othman I.M., Anwar M.M., Nossier E.S. (2022). New Thiophene, Thienopyridine and Thiazoline-Based Derivatives: Design, Synthesis and Biological Evaluation as Antiproliferative Agents and Multitargeting Kinase Inhibitors. Bioorganic Chem..

[87] Batran R.Z., Ahmed E.Y., Nossier E.S., Awad H.M., Latif N.A.A. (2024). Anticancer Activity of New Triazolopyrimidine-Linked Coumarin and Quinolone Hybrids: Synthesis, Molecular Modeling, TrkA, PI3K/AKT and EGFR Inhibition. J. Mol. Struct..

[88] Hussein M.E., Mohamed O.G., El-Fishawy A.M., El-Askary H.I., El-Senousy A.S., El-Beih A.A., Nossier E.S., Naglah A.M., Almehizia A.A., Tripathi A. (2022). Identification of Antibacterial Metabolites from Endophytic Fungus *Aspergillus fumigatus*, Isolated from *Albizia lucidior* Leaves (Fabaceae), Utilizing Metabolomic and Molecular Docking Techniques. Molecules.

[89] Alsfouk A.A., Othman I.M., Nasr H.M., Ishak E.A., Saleh A., Nossier E.S. (2025). Synthesis, Characterization, Computational Studies and In Vitro Biological Effects of Novel Pyrazoles, Pyridazines, Thiazoles Containing a Quinazoline Scaffold. ChemistrySelect.

[90] Othman I.M., Alamshany Z.M., Tashkandi N.Y., Nossier E.S., Anwar M.M., Radwan H.A. (2022). Chemical Synthesis and Molecular Docking Study of New Thiazole, Thiophene, and Thieno[2,3-*d*]Pyrimidine Derivatives as Potential Antiproliferative and Antimicrobial Agents. J. Mol. Struct..

[91] Alsfouk A.A., Othman I.M., Anwar M.M., Saleh A., Nossier E.S. (2025). Design, Synthesis, and In Silico Studies of New Quinazolinones Tagged Thiophene, Thienopyrimidine, and Thienopyridine Scaffolds as Antiproliferative Agents with Potential p38α MAPK Kinase Inhibitory Effects. RSC Adv..

[92] Abd El-Meguid E.A., El-Deen E.M.M., Moustafa G.O., Awad H.M., Nossier E.S. (2022). Synthesis, Anticancer Evaluation and Molecular Docking of New Benzothiazole Scaffolds Targeting FGFR-1. Bioorganic Chem..

[93] Abraham M.J., Murtola T., Schulz R., Páll S., Smith J.C., Hess B., Lindahl E. (2015). GROMACS: High Performance Molecular Simulations through Multi-Level Parallelism from Laptops to Supercomputers. SoftwareX.

[94] Neese F. (2012). The ORCA Program System. Wiley Interdiscip. Rev. Comput. Mol. Sci..

[95] Becke A.D. (1993). Density-Functional Thermochemistry. III. The Role of Exact Exchange. J. Chem. Phys..

[96] Johnson E.R., Keinan S., Mori-Sánchez P., Contreras-García J., Cohen A.J., Yang W. (2010). Revealing Noncovalent Interactions. J. Am. Chem. Soc..

[97] Murray J.S., Sen K. (1996). Molecular Electrostatic Potentials: Concepts and Applications.

[98] Geerlings P., De Proft F., Langenaeker W. (2003). Conceptual Density Functional Theory. Chem. Rev..

