# Supplementary material for: Design and Synthesis of Novel Morpholine-Derived Nitrogen-Rich Scaffolds as Multifunctional Anticancer and Antibacterial Agents: Biological Evaluation and Computational Studies
Source: Pharmaceutics. 2026 Aug 9;18(8):982. doi: 10.3390/pharmaceutics18080982 (PMC13516450; doi:10.3390/pharmaceutics18080982)
Supplement: Supplementary file 1 [file pharmaceutics-18-00982-s001.zip › pharmaceutics-4483561-supplementary.pdf]

(Supplementary Information)

Design and Synthesis of Novel Morpholine-Derived Nitrogen-Rich Scaffolds as Multifunctional Anticancer and Antibacterial Agents: Biological Evaluation and Computational Studies

Hagar S. El-Hema [1,*], Esraa Adel [2], Wagdy I. El-Dougdoug [2], Ashraf A.F. Wasfy [2], Ahmed F. El-Sayed [3,4], Eman S. Nossier [5], Modather F. Hussein [6], Reem Binsuwaidan [7], Asmaa Saleh [7], and Adel A.-H. Abdel-Rahmanh [8,*]

[1] Basic Science Department (Chemistry), Thebes Higher Institute for Engineering, Thebes Academy, Maadi 11434, Egypt

[2] Chemistry Department, Faculty of Science, Benha University, Benha 13518, Egypt

[3] Microbial Genetics Department, Biotechnology Research Institute, National Research Centre, Giza, Egypt;

[4] Egypt Center for Research and Regenerative Medicine (ECRRM), Cairo, Egypt;

[5] Pharmaceutical Medicinal Chemistry and Drug Design Department, Faculty of Pharmacy (Girls), Al-Azhar University, Cairo, 11754, Egypt

[6] Chemistry Department, College of Science, Jouf University, P.O. Box 2014, Sakaka, Aljouf 72341, Saudi Arabia

[7] Department of Pharmaceutical Sciences, College of Pharmacy, Princess Nourah bint Abdulrahman University, P.O.Box 84428, Riyadh 11671, Saudi Arabia

[8] Chemistry Department, Faculty of Science, Menoufia University, Shebin El-Kom, 32511, Egypt

Content

- Experimental of chemistry, biological evaluation, ADMET prediction, molecular docking, molecular dynamics simulations, and Quantum Chemical Calculations
- **Table S1.** The percentage cytotoxicity of the active morpholine-based derivatives **1-12** on HCT116 and MCF-7, and normal WI38 cell lines at different concentrations.

- **Table S2.** Raw data from the EGFR inhibition assay of morpholine-based derivatives **3**, **10**, and **12** as obtained directly from the assay instrument.
- **Table S3.** Raw data from the PI3K inhibition assay of morpholine-based derivatives **3**, **10**, and **12** as obtained directly from the assay instrument.
- **Table S4.** Raw data from the mTOR inhibition assay of morpholine-based derivatives **3**, **10**, and **12** as obtained directly from the assay instrument.
- **Table S5.** Cell cycle distribution analysis of MCF-7 and HCT116 cells treated with morpholine-triazine derivative **10**, expressed as percentage of cells in different phases based on DNA content.
- **Table S6.** Apoptosis and necrosis analysis of MCF-7 and HCT116 cells treated with morpholine-triazine derivative **10**, showing total, early, and late apoptotic populations.
- **Table S7.** Effect of morpholine-triazine derivative **10** on mitochondrial membrane potential ($\Delta\Psi m$) in HCT-116 cells.
- **Table S8.** Quantitative fluorescence analysis of mitochondrial membrane potential ($\Delta\Psi m$) in morpholine-triazine derivative **10**-treated and untreated HCT-116 cells.
- **Table S9.** Raw data of Effect of morpholine-triazine derivative **10** on the intracellular ROS assay in HCT-116 cells, including the standard calibration curve, plate layout, and recorded fluorescence measurements used for ROS quantification.
- **Table S10.** Raw RT-PCR data showing the relative expression analysis of apoptosis-related genes (caspase-3, caspase-9, Bax, and Bcl-2) in morpholine-triazine derivative **10**-treated and untreated HCT-116 cells, including Ct values, ΔCt, $\Delta\Delta$Ct, and fold-change calculations.
- **Figure S1**–**S48**. Copies of IR, ^{1}H NMR, ^{13}C NMR, and mass spectra of Compounds.

- **Figure S49.** Concentration-dependent effects of morpholine-based derivatives **1–12** and erlotinib on the viability of HCT-116, MCF-7, and WI-38 cells. Relative cell viability (%) is plotted against compound concentration (μM).
- **Figure S50.** A, B, and C diagrams show two-dimensional views of redocking the co-crystallized ligands erlotinib, 6-(1*H*-pyrazolo[3,4-*b*]pyridin-5-yl)-4-pyridin-4-ylquinoline (LXX), and 3-(4-morpholin-4-ylpyrido[3',2':4,5]furo[3,2-*d*]pyrimidin-2-yl)phenol (X6K), within the EGFR, PI3K, and mTOR active sites (PDB IDs: 1M17, 3L54, and 4JT6, respectively).
- **Figure S51.** Two-dimensional view of redocking the co-crystallized ligand ciprofloxacin, within the *S. aureus* DNA gyrase active site (PDB code: 2XCT).

- **Figure S52.** DFT-optimized geometries (OPT) and molecular electrostatic potential (ESP) surface maps of morpholine-based derivatives **1–12**.

2. Materials and Methods

2.1. Chemistry

All reagents and solvents used in the present study were purchased from Sigma-Aldrich Chemical Co. (USA) and other reputable commercial suppliers and were used as received unless otherwise specified. Solvents were dried prior to use according to standard laboratory procedures. Melting points were determined using a Gallenkamp melting point apparatus and are reported as uncorrected values. Infrared (IR) spectra (KBr discs) were recorded on a Thermo Scientific Nicolet iS10 FTIR spectrometer at the Faculty of Science, Menoufia University, Egypt. ^{1}H and ^{13}C NMR spectra were measured in DMSO-d_6 on a Bruker spectrometer operating at 400 MHz for ^{1}H NMR and 100 MHz for ^{13}C NMR. Chemical shifts (δ) are reported in parts per million (ppm) relative to tetramethylsilane (TMS) as internal standard, while coupling constants (J) are expressed in Hertz (Hz). Mass spectrometric analyses were performed at the Faculty of Science, Al-

Azhar University, Egypt, using a Thermo Scientific GC/MS ISQ LT system equipped with a direct-probe controller inlet and single-quadrupole mass analyzer. Data acquisition and processing were carried out using Thermo Xcalibur software. Elemental analyses (C, H, and N) were conducted using a PerkinElmer 2400 elemental analyzer at the Microanalytical Unit, Faculty of Science, Cairo University, Egypt. Reaction progress and purity of the synthesized compounds were monitored by thin-layer chromatography (TLC) using Macherey–Nagel aluminum-backed silica gel 60 F_{254} plates, and spots were visualized under ultraviolet light (254 nm). All biological experiments were conducted at the Research Laboratory, Faculty of Pharmacy, Mansoura University, and VACSERA, Egypt, following standardized sterile procedures and applicable biosafety guidelines.

2.2. Biological activity

2.2.1. *In Vitro* Cytotoxicity and Selectivity Assessment

The antiproliferative activities of the synthesized compounds were evaluated against human breast adenocarcinoma (MCF-7) and colorectal carcinoma (HCT-116) cell lines. The normal human lung fibroblast cell line (WI-38) was employed to evaluate cytotoxic selectivity toward normal cells. All cell lines were obtained from the American Type Culture Collection (ATCC) through the Holding Company for Biological Products and Vaccines (VACSERA, Cairo, Egypt). Cells were cultured in RPMI-1640 medium supplemented with 10% fetal bovine serum (FBS), 100 U/mL penicillin, and 100 μg/mL streptomycin and maintained at 37 °C in a humidified atmosphere containing 5% CO_2. Cells were seeded into 96-well plates at a density of 1×10^4 cells/well and incubated for 48 h before treatment. Cell viability was assessed using the MTT assay based on the reduction of the yellow tetrazolium salt (MTT) into insoluble purple formazan crystals by mitochondrial dehydrogenases of viable cells. Following treatment with the tested compounds for 24 h, 20 μL of MTT solution (5 mg/mL) was added to each well, and the plates were incubated for an additional 4 h. Subsequently, 100 μL of DMSO was added

to dissolve the formed formazan crystals. Absorbance was measured at 570 nm using an EXL 800 microplate reader (USA). Cell viability was calculated relative to untreated control cells according to the following equation: Cell viability (%) = (A_{570} treated / A_{570} control) × 100

2.2.2. Multitarget EGFR/PI3K/mTOR Kinase Inhibition

Based on the antiproliferative screening results, compounds **3**, **10**, and **12** were selected for further evaluation against key components of the EGFR/PI3Kα/mTOR signaling pathway. EGFR inhibitory activity was determined using the EGFR Kinase Assay Kit (BPS Bioscience, San Diego, CA, USA; Catalog No. 40321) according to the manufacturer's protocol. The assay was performed in white 96-well microplates using recombinant human wild-type EGFR enzyme, ATP, and Poly(Glu 4:1) substrate. The tested compounds were evaluated at serial concentrations of 10, 1, 0.1, 0.01, and 0.001 µM. Following incubation of the reaction mixtures with EGFR enzyme at 30 °C for 40 min, Kinase-Glo® MAX reagent (Promega, Madison, WI, USA) was added, and luminescence was measured using a Tecan Spark microplate reader. Erlotinib was employed as the reference EGFR inhibitor. The inhibitory activity against PI3Kα was evaluated using the PI3Kα (p110α/p85α) Assay Kit (BPS Bioscience, San Diego, CA, USA; Catalog No. 79802). The assay utilized recombinant PI3Kα enzyme, PI3K lipid substrate, ATP, and kinase assay buffer. The tested compounds were dissolved in DMSO and serially diluted while maintaining the final DMSO concentration below 0.5%. The kinase reaction was conducted at 30 °C for 40 min, followed by sequential addition of ADP-Glo® reagent and Kinase Detection Reagent according to the manufacturer's protocol. Luminescence signals were recorded using a microplate reader, and pictilisib was used as the reference PI3K inhibitor. The inhibitory activity against mammalian target of rapamycin (mTOR, FRAP1) was assessed using the LanthaScreen® Eu Kinase Binding Assay (Invitrogen, Thermo Fisher Scientific, Carlsbad, CA, USA). This time-resolved fluorescence resonance

energy transfer (TR-FRET)-based assay measures competitive binding of test compounds to the ATP-binding site of mTOR through displacement of an Alexa Fluor® 647-labeled kinase tracer. Recombinant FRAP1 kinase, LanthaScreen® Eu-anti-GST Tag antibody, Kinase Tracer 314, and mTOR binding buffer were employed according to the supplier's instructions. Assays were performed by sequential addition of the test compound solution, kinase/antibody mixture, and tracer solution, followed by incubation at room temperature for 60 min. TR-FRET signals were measured at excitation and emission wavelengths of 340, 615, and 665 nm, respectively, and the 665/615 emission ratio was used to generate concentration–response curves. Rapamycin served as the reference mTOR inhibitor. For all assays, All measurements were performed in triplicate according to the manufacturers' protocols, and the results were expressed as mean ± SD.

2.2.3. Cell Cycle Modulation and Apoptosis Induced by Compound 10

To further investigate the mechanism underlying the antiproliferative activity of compound **10**, cell cycle distribution and apoptosis analyses were performed in MCF-7 and HCT-116 cells using flow cytometry. For cell cycle analysis, DNA content was determined using the Propidium Iodide Flow Cytometry Kit for Cell Cycle Analysis (Abcam, Cambridge, UK; Catalog No. ab139418). Briefly, treated and untreated cells were harvested, washed with phosphate-buffered saline (PBS), fixed in cold 66% ethanol, and incubated at 4 °C. Subsequently, cells were stained with propidium iodide/RNase staining solution and incubated in the dark prior to flow cytometric analysis. DNA content was measured using 488 nm laser excitation, and the percentages of cells in the G0/G1, S, and G2/M phases were determined by quantification of cellular DNA content. Apoptosis analysis was carried out using the Annexin V-FITC Apoptosis Detection Kit (BioVision Inc., Mountain View, CA, USA; Catalog No. K101-25) according to the manufacturer's instructions. Briefly, treated and untreated cells were collected, washed, and resuspended in 1× binding buffer, followed by staining with Annexin V-FITC and

propidium iodide (PI). After incubation in the dark, samples were analyzed by flow cytometry. Annexin V-FITC and PI fluorescence signals were used to quantify viable, early apoptotic, late apoptotic, and necrotic cell populations. All experiments were performed according to the manufacturers' protocols, and the results were expressed as percentages of the total analyzed cell population.

2.2.4. Effect of Compound 10 on Mitochondrial Membrane Potential (ΔΨm) in HCT-116 Cells

To investigate the effect of compound **10** on mitochondrial membrane integrity, mitochondrial membrane potential (ΔΨm) was evaluated in HCT-116 cells using the MitoProbe™ DiOC2(3) Assay Kit for Flow Cytometry (Molecular Probes™, Thermo Fisher Scientific, USA; Cat. No. M34150). HCT-116 cells were seeded under standard culture conditions and allowed to attach overnight. Cells were subsequently treated with compound **10** at its biologically active concentration for the predetermined incubation period, while untreated cells served as the negative control. Following treatment, cells were harvested, washed twice with phosphate-buffered saline (PBS), and resuspended in the staining buffer supplied with the kit. The cell suspension was incubated with the lipophilic cationic fluorescent dye DiOC2(3) according to the manufacturer's protocol. This dye accumulates within polarized mitochondria in a membrane potential-dependent manner; therefore, loss of mitochondrial membrane potential results in reduced intracellular fluorescence intensity. After staining, cells were protected from light and immediately analyzed by flow cytometry using a BD FACSCalibur flow cytometer (BD Biosciences, San Jose, CA, USA). At least 10,000 events were collected for each sample. Cellular debris and aggregates were excluded by forward-scatter (FSC) and side-scatter (SSC) gating. Fluorescence signals were recorded under identical instrument settings for all samples, and mean fluorescence intensity (MFI) values were determined using the instrument software. Mitochondrial membrane potential was expressed as a percentage

relative to untreated control cells, which were considered 100% polarized mitochondria. A reduction in fluorescence intensity was interpreted as mitochondrial membrane depolarization and disruption of mitochondrial function. All experiments were performed according to the manufacturer's instructions, and data were expressed as percentage of control cells.

2.2.5. Effect of Compound 10 on Intracellular Reactive Oxygen Species (ROS) Generation in HCT-116 Cells

Intracellular reactive oxygen species (ROS) generation was evaluated in HCT-116 cells following treatment with compound **10** using the DCFDA/H2DCFDA Cellular ROS Assay Kit (Abcam, Cambridge, UK; Catalog No. ab113851) according to the manufacturer's instructions. DCFDA (2′,7′-dichlorofluorescin diacetate) is a cell-permeable fluorogenic probe that is deacetylated intracellularly and subsequently oxidized by reactive oxygen species to yield the highly fluorescent compound dichlorofluorescein (DCF), which serves as an indicator of intracellular oxidative stress. Briefly, HCT-116 cells were cultured under standard conditions and treated with compound 10 at its corresponding IC_{50} concentration, while untreated cells served as the control group. Following treatment, cells were harvested and washed with phosphate-buffered saline (PBS) to remove residual medium. Cells were then incubated with DCFDA working solution and protected from light for 30 min at 37 °C to allow intracellular loading of the probe. After incubation, cells were washed with assay buffer to remove excess dye and resuspended as a single-cell suspension for analysis. Fluorescence intensity was measured by flow cytometry using 488 nm excitation and detection at approximately 535 nm (FITC channel). At least 10,000 events were acquired per sample, and cellular debris was excluded by forward- and side-scatter gating. The mean fluorescence intensity (MFI) was recorded and compared with untreated control cells. Intracellular ROS levels were expressed as fold change relative to the control group.

Increased fluorescence intensity was interpreted as enhanced intracellular ROS accumulation and oxidative stress. All experiments were performed according to the manufacturer's protocol, and the results were expressed as mean ± SD.

2.2.6. Modulation of Apoptosis-Related Gene Expression by Compound 10

To investigate the molecular mechanism underlying the apoptotic activity of compound **10**, the mRNA expression levels of apoptosis-related genes, namely Bax, Bcl-2, Caspase-9, and Caspase-3, were quantified in HCT-116 cells using quantitative real-time polymerase chain reaction (qRT-PCR). HCT-116 cells were seeded in 6-well plates at a density of 3 × 10^5 cells/well and incubated overnight at 37 °C in a humidified atmosphere containing 5% CO2. Subsequently, cells were treated with compound **10** at its corresponding IC_{50} concentration for 24 h, whereas untreated cells served as the control group. Each experimental group was analyzed in triplicate. In addition, no-reverse transcriptase (No-RT) and no-template controls (NTC) were included to exclude genomic DNA contamination and primer-dimer formation, respectively. Total RNA was extracted from treated and untreated cells using TRIzol reagent (Invitrogen, USA) or QIAzol lysis reagent (Qiagen, Germany) according to the manufacturers' instructions. Briefly, cells were lysed directly in the culture wells using 1 mL of lysis reagent and transferred to RNase-free microcentrifuge tubes. Chloroform (200 µL) was added to each sample followed by vigorous shaking for 15 s and incubation at room temperature for 2–3 min. Samples were centrifuged at 12,000 ×g for 15 min at 4 °C to separate the aqueous and organic phases. The upper aqueous phase was transferred to a new tube and mixed with 500 µL isopropanol for RNA precipitation. After incubation for 10 min, samples were centrifuged at 12,000 ×g for 10 min at 4 °C. The resulting RNA pellet was washed with 75% ethanol, air-dried for 5–10 min, and dissolved in 20–30 µL RNase-free water. RNA concentration and purity were determined spectrophotometrically using a NanoDrop spectrophotometer (Thermo Fisher Scientific, USA), and samples with an

A260/A280 ratio of approximately 2.0 were used for subsequent analysis. For cDNA synthesis, 1 μg of total RNA was treated with DNase I to eliminate residual genomic DNA and reverse-transcribed using a High-Capacity RNA-to-cDNA kit (Applied Biosystems, USA) according to the manufacturer's protocol. The reverse transcription program consisted of incubation at 25 °C for 5 min, followed by 46 °C for 20 min and enzyme inactivation at 95 °C for 1 min. The synthesized cDNA was diluted ten-fold with nuclease-free water prior to amplification. Quantitative PCR amplification was performed using Bio-Rad SYBR Green PCR Master Mix on a Rotor-Gene 6000 Real-Time PCR System (Corbett Life Science, Australia). Each reaction was carried out in a total volume of 20 μL containing 10 μL of 2× SYBR Green Master Mix, 0.8 μL of forward primer (10 μM), 0.8 μL of reverse primer (10 μM), 2 μL of diluted cDNA template, and 6.4 μL of nuclease-free water. The amplification program consisted of an initial denaturation step at 95 °C for 2 min, followed by 40 cycles of denaturation at 95 °C for 15 s and annealing/extension at 60 °C for 60 s. Fluorescence data were collected during each extension step. A melt curve analysis was subsequently performed from 65 °C to 95 °C with increments of 0.5 °C to confirm the specificity of the amplified products The primer sequences used for amplification are summarized below. Primer sequences used for qRT-PCR analysis of apoptosis-related genes in HCT-116 cells treated with compound **10**.

**Gene**	**Forward Primer (5′→3′)**	**Reverse Primer (5′→3′)**
**Bax**	TTCCGAGTGGCAGCTGAGATGTTT	TGCTGGCAAAGTAGAAGAGGGCAA
**Bcl-2**	ATCGCCCTGTGGATGACTGAGT	GCCAGGAGAAATCAAACAGAGGC
**Caspase-9**	GTTTGAGGACCTTCGACCAGCT	CAACGTACCAGGAGCCACTCTT

**Caspase-3**	TCATTATTCAGGCCTGCCGAGG	TTCTGACAGGCCATGTCATCCTCA
**GAPDH**	ACAGCCTCAAGATCATCAGCAA	CCATCACGCCACAGTTTCC

The expression levels of Bax, Bcl-2, Caspase-9, and Caspase-3 were normalized against the housekeeping gene GAPDH, and relative gene expression was calculated using the comparative $2^{-\Delta\Delta Ct}$ (Livak) method. Untreated control cells were assigned a value of 1, and the results were expressed as fold change relative to the control group. Melt curve analysis revealed a single sharp peak for each amplicon, confirming the specificity of the amplification reactions, while no amplification signal was detected in the NTC wells.

2.2.7. *In Vitro* Antimicrobial Activity

The antibacterial activity of the synthesized compounds was evaluated using the agar-well diffusion assay following the method reported by Sroor et al. with slight modifications. Four standard bacterial strains obtained from the Microbial Genetics Laboratory, National Research Centre (NRC, Egypt), were used, including the Gram-positive bacteria *Enterococcus faecalis* ATCC 29212 and *Staphylococcus aureus* ATCC 25923, as well as the Gram-negative bacteria *Escherichia coli* ATCC 25915 and *Pseudomonas aeruginosa* ATCC 10145. The tested compounds were prepared at a concentration of 20 μg/mL and introduced into the agar wells, while ciprofloxacin (20 μg/mL) was used as the reference antibacterial agent and DMSO served as the negative control. The inoculated plates were incubated at 37 °C for 24 h, after which the diameters of the inhibition zones were measured in millimeters. All experiments were performed in biological triplicate, and the results are expressed as mean ± SE. The assay was carried out according to the procedure described by Sroor et al. with slight modifications.

2.2.8. Determination of Minimum Inhibitory Concentrations (MICs)

The minimum inhibitory concentration (MIC) of the most active antibacterial compounds (**12**, **3**, and **10**) was determined using the broth microdilution method according to the Clinical and Laboratory Standards Institute (CLSI) guidelines, following the procedure described in Ref. [79]. Briefly, stock solutions of the tested compounds were prepared in dimethyl sulfoxide (DMSO) and subsequently diluted with sterile nutrient broth to obtain final concentrations ranging from 5 to 160 μg/mL. Each dilution was inoculated with standardized bacterial suspensions of *Enterococcus faecalis* ATCC 29212, *Staphylococcus aureus* ATCC 25923, *Escherichia coli* ATCC 25915, and *Pseudomonas aeruginosa* ATCC 10145, followed by incubation at 37 °C for 24 h. Bacterial growth was assessed visually, and aliquots from each well were cultured on nutrient agar plates when necessary to confirm growth inhibition. The MIC value was defined as the lowest concentration of the tested compound that completely inhibited visible bacterial growth compared with the untreated control. All experiments were performed in triplicate.

2.2.9. Evaluation of Antibiofilm Activity

The antibiofilm activity of the most active compounds (**12**, **3**, and **10**) was evaluated using the crystal violet microtiter plate assay according to the method described in Ref. [79]. Briefly, each compound was tested at its respective minimum inhibitory concentration (MIC). A mixture containing 170 μL of Mueller–Hinton broth, 15 μL of the bacterial suspension, and the tested compound was transferred into sterile 96-well microtiter plates and incubated at 37 °C for 24 h. Following incubation, the culture medium was carefully discarded, and the wells were gently washed three times with phosphate-buffered saline (PBS, pH 7.2) to remove non-adherent cells. The attached biofilms were stained with 0.1% (w/v) crystal violet for 1 h, followed by washing with PBS to remove excess stain. The bound dye was dissolved in 200 μL of ethanol, and the

biofilm biomass was quantified by measuring the absorbance at 590 nm using a **microplate reader. All experiments were performed in triplicate.**

2.2.10. DPPH and ABTS Radical Scavenging Activity

DPPH Radical Scavenging Assay: The DPPH free radical scavenging activity of the synthesized compounds was determined according to the method described in Ref. [80]. Briefly, 1.0 mL of each test solution was mixed with 1.0 mL of freshly prepared 0.3 mM DPPH methanolic solution. The reaction mixtures were vortexed, incubated in the dark at 30 °C for 30 min, and the absorbance was measured at 517 nm using a UV–Vis spectrophotometer. The radical scavenging activity was calculated according to the following equation: Scavenging activity (%) = $[1 - (A_{sample}/A_{control})] \times 100$

ABTS Radical Scavenging Assay: The ABTS radical scavenging activity was evaluated according to the method described in Ref. [80]. Briefly, 1.0 mL of each test solution was mixed with 1.0 mL of diluted ABTS solution and incubated at 30 °C for 30 min. The absorbance was then measured at 734 nm against the corresponding blank using a UV–Vis spectrophotometer. The percentage of ABTS radical scavenging activity was calculated using the following equation: Scavenging activity (%) = $[1 - (A_{sample}/A_{control})] \times 100$

2.3. Computational studies

2.3.1. Computational ADMET and Drug-Likeness Assessment

The absorption, distribution, metabolism, excretion, and toxicity (ADMET) properties of the most biologically active morpholine derivatives (Compounds **3**, **10**, and **12**) were predicted using the SwissADME and pkCSM online platforms. Drug-likeness was evaluated according to Lipinski's rule of five and Veber's criteria. Key physicochemical parameters, including molecular weight (MW), lipophilicity (LogP), topological polar surface area (TPSA), hydrogen bond donors (HBD), hydrogen bond

acceptors (HBA), and the number of rotatable bonds, were calculated. In addition, pharmacokinetic parameters, including gastrointestinal (GI) absorption, blood–brain barrier (BBB) permeability, P-glycoprotein (P-gp) substrate properties, cytochrome P450 enzyme inhibition (CYP3A4, CYP2C9, CYP2C19, CYP2D6, and CYP1A2), skin permeability, volume of distribution (VDss), total clearance, and renal OCT2 substrate prediction, were evaluated. Toxicological endpoints, including AMES mutagenicity, hepatotoxicity, hERG I and hERG II inhibition, maximum tolerated dose, oral rat acute toxicity (LD_{50}), chronic oral toxicity (LOAEL), skin sensitization, and minnow toxicity, were also predicted. The obtained ADMET profiles were used to evaluate the drug-likeness, pharmacokinetic behavior, and safety characteristics of the selected compounds, thereby supporting the experimental biological findings.

2.3.2. Molecular docking simulation

The 2D structures of morpholino-2-oxoethyl-based derivatives **3, 10,** and **12** were drawn through Chem. Draw. The protonated 3D was employed using standard bond lengths and angles, using Molecular Operating Environment (MOE-Dock) software version 2024.0601 (Chemical Computing Group, Montreal, Canada). Then, the geometry optimization and energy minimization were applied to get the Conf Search module in MOE, followed by saving the MOE file for the upcoming docking process. The co-crystallized structures of EGFR, PI3K, mTOR and *S. aureus* DNA gyrase (PDB IDs: 1M17, 3L54, 4JT6, and 2XCT, respectively) with their ligands erlotinib, 6-(1*H*-pyrazolo[3,4-*b*]pyridin-5-yl)-4-pyridin-4-ylquinoline (LXX), and 3-(4-morpholin-4-ylpyrido[3',2':4,5]furo[3,2-*d*]pyrimidin-2-yl)phenol (X6K) and ciprofloxacin, respectively from protein data bank. All minimizations were performed using MOE until an RMSD gradient of 0.05 $kcal \cdot mol^{-1} Å^{-1}$ with the MMFF94x force field, and the partial charges were automatically calculated. Preparation of the enzymes' structures was done for molecular docking using the Protonate 3D protocol with the default options in MOE. London dG

scoring function and Triangle Matcher placement method were used in the docking protocol. At first, validation of the docking processes was established by docking of the native ligands, followed by docking of the derivatives **3, 10,** and **12** within the ATP-binding sites after elimination of the co-crystallized ligands.

2.3.3. Molecular dynamics simulations

Molecular dynamics (MD) simulations were performed to investigate the stability and interaction dynamics of protein-ligand complexes, utilizing GROMACS 2018 with the CHARMM36 force field for protein topology and the CGenFF server (Geoff) for ligand parameters. During the equilibration phase, ligand positions were restrained in both NVT (constant particle number, volume, and temperature) and NPT (constant particle number, pressure, and temperature) ensembles, each executed for 1000 ps at 300 K and 1.0 bar to ensure system stability. Following the simulations, structural stability, residue-level flexibility, and overall compactness of the complexes were assessed using post-simulation analyses such as Root Mean Square Deviation (RMSD), Root Mean Square Fluctuation (RMSF), and radius of gyration (Rg).

2.3.4. Quantum Chemical Calculations

Density Functional Theory (DFT) calculations were carried out using the ORCA 6 quantum chemistry package at the B3LYP/6-311G++(d,p) level of theory to optimize the molecular geometries of all investigated compounds. Frequency calculations were subsequently performed to confirm that all optimized structures corresponded to true local minima by the absence of imaginary frequencies. Frontier molecular orbital (FMO) analysis was employed to estimate key global reactivity descriptors, including the HOMO–LUMO energy gap (ΔE), electronegativity (χ), chemical hardness (η), softness (σ), electrophilicity index (ω), and ionization potential. Density of states (DOS) analyses were also conducted to examine the electronic state distribution and frontier orbital contributions. Molecular electrostatic potential (ESP) maps mapped onto the electron

density surface were generated to identify electron-rich and electron-deficient regions and to rationalize charge distribution behavior. Furthermore, noncovalent interaction (NCI) analyses based on the reduced density gradient (RDG) method were performed to visualize attractive, weak van der Waals, and repulsive interaction regions.

Supporting Tables

Table S1. The percentage cytotoxicity of the active compounds **1-12** on HCT116 and MCF-7, and normal WI38 cell lines at different concentrations.

Conc.(μM)	WI-38	HCT-116	MCF7
**Erlotinib**			
100	13.1	7.4	8.1
50	17.8	12.1	15.2
25	29.0	18.8	23.0
12.5	40.3	27.9	34.8
6.25	63.7	52.7	51.6
3.125	78.6	63.5	68.9
1.56	96.5	72.4	87.5
**1**			
100	43.9	35.4	31.8
50	55.2	49.1	40.9
25	62.8	56.3	61.2
12.5	84.5	69.8	70.5
6.25	95.3	83.6	86.3
3.125	100	98.5	99.1

1.56	100	100	100
		2	
100	51.4	42.7	46.3
50	70.6	58.3	57.1
25	86.1	69.2	71.6
12.5	95.3	84.8	83.4
6.25	100	99.4	96.7
3.125	100	100	100
1.56	100	100	100
		3	
100	43.5	8.5	12.4
50	71.2	18.6	24.8
25	84.6	29.4	38.5
12.5	92.3	41.7	46.2
6.25	96.1	49.8	55.1
3.125	100	78.5	81.6
1.56	100	100	100
		4	
100	48.2	38.6	35.4
50	60.5	49.1	48.3
25	76.1	62.7	60.5
12.5	88.3	78.5	72.6
6.25	98.7	96.2	89.2
3.125	100	100	100
1.56	100	100	100

5			
100	47.1	18.7	21.9
50	55.7	26.4	28.6
25	76.5	33.9	41.7
12.5	89.2	40.6	50.3
6.25	100	68.3	72.5
3.125	100	85.5	91.2
1.56	100	100	100
6			
100	38.5	23.8	29.6
50	54.2	35.7	40.9
25	69.1	50.4	52.8
12.5	88.4	63.9	65.4
6.25	100	71.3	78.5
3.125	100	90.5	98.2
1.56	100	100	100
7			
100	31.4	51.6	44.3
50	41.8	70.4	56.1
25	52.7	86.3	70.5
12.5	73.5	95.1	83.7
6.25	88.2	100	97.4
3.125	99.3	100	100
1.56	100	100	100
8			

100	38.6	30.4	12.9
50	51.7	42.5	21.4
25	65.2	53.6	29.5
12.5	79.4	66.8	40.3
6.25	97.1	87.9	67.1
3.125	100	99.3	81.6
1.56	100	100	98.2
9			
100	52.3	49.8	42.7
50	69.6	63.1	54.4
25	78.5	75.9	66.5
12.5	96.7	91.5	78.2
6.25	100	100	93.6
3.125	100	100	100
1.56	100	100	100
10			
100	36.2	9.7	6.5
50	49.1	17.8	13.2
25	60.5	26.3	20.8
12.5	75.4	32.2	32.7
6.25	87.6	61.5	48.3
3.125	100	70.1	63.4
1.56	100	90.4	86.1
11			
100	26.5	40.7	38.2

50	35.8	58.2	50.9
25	50.6	70.4	62.3
12.5	67.1	81.9	78.6
6.25	82.3	98.6	96.4
3.125	95.2	100	100
1.56	100	100	100
	**12**		
100	41.6	9.2	10.5
50	69.4	14.4	21.4
25	82.7	21.8	30.9
12.5	91.3	38.0	33.2
6.25	96.8	48.2	49.6
3.125	92.1	77.3	80.2
1.56	100	100	100

Table S2. Raw data from the EGFR inhibition assay of morpholine-based derivatives **3**, **10**, and **12** as obtained directly from the assay instrument.

EGFR

						[redacted]					
code	IC50	conc	log	%inh	ΔT	ΔRFU	EC		RFU1	RFU2	RFU3
s3		10	1	89.47	30	6575	62414		6514	6627	6582
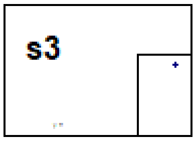		1	0	68.92	30	19400	62414		19221	19555	19423
		0.1	-1	32	30	42444	62414		42053	42784	42494
		0.01	-2	16.44	30	52154	62414		51674	52572	52216
		0.001	-3	7.423	30	57781	62414		57249	58244	57850
EC			#NUM!	0	30	62414	62414		61839	62914	62488
	[redacted]		[redacted]		[redacted]						
code	IC50	conc	log	%inh	ΔT	ΔRFU	EC				

s10		10	1	93.67	30	3940	62237	3915	3965	3940
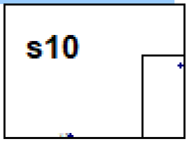		1	0	79.83	30	12556	62237	12476	12637	12556
		0.1	-1	43.62	30	35086	62237	34862	35311	35086
		0.01	-2	27.63	30	45040	62237	44752	45328	45040
		0.001	-3	12.06	30	54732	62237	54382	55082	54732
EC			#NUM!	0	30	62237	62237	61839	62635	62237
	[redacted]		[redacted]		[redacted]	[redacted]				

code	IC50	conc	log	%inh	ΔT	ΔRFU	EC			
s12		10	1	91.18	30	5492	62237	5457	5527	5492
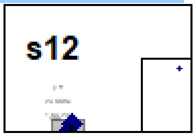		1	0	70.27	30	18505	62237	18387	18624	18505
		0.1	-1	23.13	30	47844	62237	47538	48150	47844
		0.01	-2	10.64	30	55618	62237	55262	55973	55618
		0.001	-3	3.199	30	60246	62237	59861	60631	60246
EC			#NUM!	0	30	62237	62237	61839	62635	62237
	[redacted]		[redacted]		[redacted]	[redacted]				

code	IC50	conc	log	%inh	ΔT	ΔRFU	EC			
Erlotinib		10	1	94.43	30	3464	62237	3442	3486	3464
		1	0	82.11	30	11136	62237	11065	11207	11136
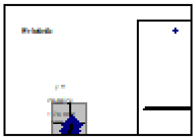		0.1	-1	51.87	30	29954	62237	29762	30145	29954
		0.01	-2	33.08	30	41648	62237	41382	41914	41648
		0.001	-3	14.69	30	53095	62237	52756	53435	53096
EC			#NUM!	0	30	62237	62237	61839	62635	62237
	[redacted]		[redacted]		[redacted]	[redacted]				

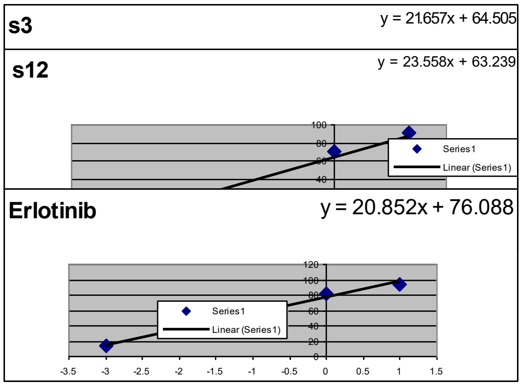

Table S3. Raw data from the PI3K inhibition assay of morpholine-based derivatives **3**, **10**, and **12** as obtained directly from the assay instrument.

PI3K*α*

code	IC50	conc	log	%inh	ΔT	ΔRFU	EC	RFU1	RFU2	RFU3
s3		10	1	87.76	30	9454	77245	9443	9470	9450
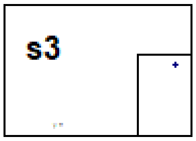		1	0	62.07	30	29300	77245	29264	29348	29287
		0.1	-1	25.65	30	57431	77245	57361	57526	57406
		0.01	-2	14.12	30	66335	77245	66254	66444	66306
		0.001	-3	6.228	30	72434	77245	72346	72554	72402
EC			#NUM!	0	30	77245	77245	77151	77373	77211

code	IC50	conc	log	%inh	ΔT	ΔRFU	EC			
s10		10	1	91.22	30	6782	77245	6774	6793	6779
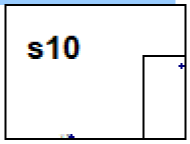		1	0	71.07	30	22346	77245	22319	22383	22336
		0.1	-1	42.07	30	44749	77245	44695	44823	44730
		0.01	-2	24.53	30	58297	77245	58226	58393	58271
		0.001	-3	18.12	30	63252	77245	63175	63357	63224
EC			#NUM!	0	30	77245	77245	77151	77373	77211

code	IC50	conc	log	%inh	ΔT	ΔRFU	EC			
s12		10	1	88.58	30	8824	77245	8813	8838	8820
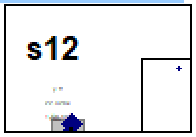		1	0	61.74	30	29551	77245	29515	29600	29538
		0.1	-1	17.37	30	63830	77245	63752	63935	63802
		0.01	-2	9.796	30	69678	77245	69593	69793	69647
		0.001	-3	2.923	30	74987	77245	74896	75111	74954
EC			#NUM!	0	30	77245	77245	77151	77373	77211

code	IC50	conc	log	%inh	ΔT	ΔRFU	EC			
pictilisib		10	1	91.22	30	6782	77245	6774	6793	6779

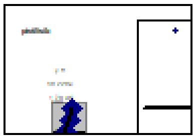

	1	0	71.42	30	22080	77245	22053	22116	22070
	0.1	-1	49.72	30	38839	77245	38792	38903	38822
	0.01	-2	30.93	30	53352	77245	53287	53440	53329
	0.001	-3	20.32	30	61549	77245	61474	61651	61522
EC		#NUM!	0	30	77245	77245	77151	77373	77211

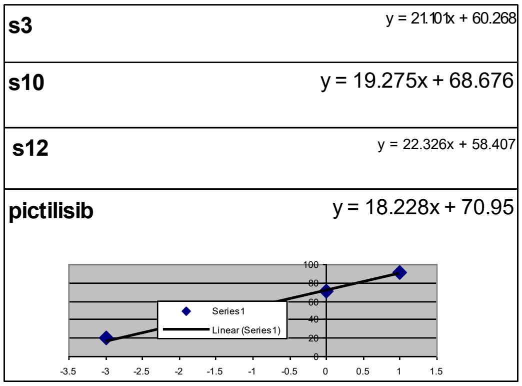

Table S4. Raw data from the mTOR inhibition assay of morpholine-based derivatives **3**, **10**, and **12** as obtained directly from the assay instrument.

mTOR

code	IC50	conc	log	%inh	ΔT	ΔRatio	EC	R1	R2	R3
s3		10	1	81.17	30	0.182	0.9651	0.181	0.183	0.181
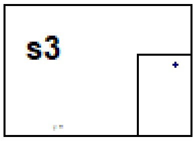		1	0	56.09	30	0.424	0.9651	0.422	0.426	0.423
		0.1	-1	20.71	30	0.765	0.9651	0.762	0.770	0.764
		0.01	-2	12.49	30	0.845	0.9651	0.841	0.850	0.843
		0.001	-3	4.058	30	0.926	0.9651	0.922	0.932	0.924
EC			#NUM!	0	30	0.965	0.9651	0.961	0.971	0.963

code	IC50	conc	log	%inh	ΔT	ΔRFU	EC			
s10		10	1	86.26	30	0.133	0.9651	0.132	0.133	0.132
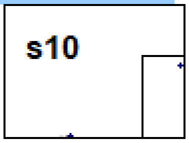		1	0	65.04	30	0.337	0.9651	0.336	0.339	0.337
		0.1	-1	38.4	30	0.595	0.9651	0.592	0.598	0.594

		0.01	-2	17.38	30	0.797	0.9651	0.794	0.802	0.796
		0.001	-3	7.076	30	0.897	0.9651	0.893	0.902	0.895
EC			#NUM!	0	30	0.965	0.9651	0.961	0.971	0.963

code	IC50	conc	log	%inh	ΔT	ΔRFU	EC			
s12		10	1	76.38	30	0.228	0.9651	0.227	0.229	0.228
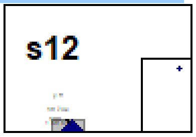		1	0	47.24	30	0.509	0.9651	0.507	0.512	0.508
		0.1	-1	14.57	30	0.825	0.9651	0.821	0.830	0.823
		0.01	-2	8.741	30	0.881	0.9651	0.877	0.886	0.879
		0.001	-3	1.977	30	0.946	0.9651	0.942	0.952	0.944
EC			#NUM!	0	30	0.965	0.9651	0.961	0.971	0.963

code	IC50	conc	log	%inh	ΔT	ΔRFU	EC			
Rapamycin		10	1	85.33	30	0.142	0.9651	0.141	0.142	0.141
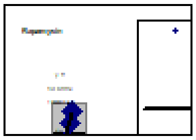		1	0	66.91	30	0.319	0.9651	0.318	0.321	0.319
		0.1	-1	36.42	30	0.614	0.9651	0.611	0.617	0.613
		0.01	-2	19.25	30	0.779	0.9651	0.776	0.784	0.778
		0.001	-3	10.72	30	0.862	0.9651	0.858	0.867	0.860
EC			#NUM!	0	30	0.965	0.9651	0.961	0.971	0.963

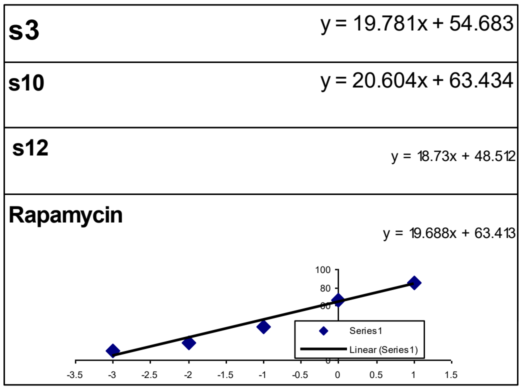

Table S5. Cell cycle distribution analysis of MCF-7 and HCT116 cells treated with morpholine-triazine derivative **10**, expressed as percentage of cells in different phases based on DNA content.

ser	Sample		DNA content				
	**code**	**IC50 uM**	**%G0-G1**	**%S**	**%G2/M**	**Comment**	
1	**S10/MCF7**	---	**39.75**	**23.46**	**36.79**	Cell cycle arrest@G2/M	
2	**C.MCF7**	---	**61.67**	**31.88**	**6.45**	---	
3	**S10/HCT116**	---	**83.22**	**12.92**	**3.86**	Cell cycle arrest@G1	
4	**C. HCT116**	---	**56.36**	**35.19**	**8.45**	---	

Table S6. Apoptosis and necrosis analysis of MCF-7 and HCT116 cells treated with morpholine-triazine derivative **10**, showing total, early, and late apoptotic populations.

s	code	conc	Apoptosis			Necrosis
			Total	Early	Late	
1	**S10/MCF7**	---	**32.66**	**19.39**	**9.85**	**3.42**
2	**C.MCF7**	---	**2.82**	**0.81**	**0.13**	**1.88**
3	**S10/HCT116**	---	**37.12**	**11.72**	**21.67**	**3.73**
4	**C. HCT116**	---	**3.67**	**0.66**	**0.27**	**2.74**

Table S7. Effect of compound **10** on mitochondrial membrane potential ($\Delta\Psi m$) in HCT-116 cells.

ser	Sample			Δψm			
	code		cells	control FL	Test FL	% of cont	
1	S10/HCT116		HCT116	39141	10846	27.71	
2	C. HCT116		---	39141	39141	100	

Table S8. Quantitative fluorescence analysis of mitochondrial membrane potential (ΔΨm) in compound **10**-treated and untreated HCT-116 cells.

Δψm sample	FL Red control	FL Green Test	% of cont
S10/HCT	39141	10846	27.71
C.HCT	39141	39141	100

Table S9. Raw data of Effect of compound **10** on the intracellular ROS assay in HCT-116 cells, including the standard calibration curve, plate layout, and recorded fluorescence measurements used for ROS quantification.

ST	conc. pg/ml
ST1	1000
ST2	500
ST3	250
ST4	125
ST5	62.5
ST6	31.2

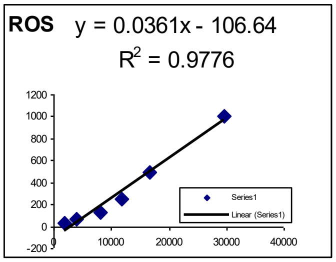

		HCT116										
	1	**2**	**3**	4	5	6	7	8	9	10	11	12
A	ST1	S10	--	--	--	--	--	--	--	--	--	--
B	ST2	S10	--	--	--	--	--	--	--	--	--	--
C	ST3	C	--	--	--	--	--	--	--	--	--	--
D	ST4	C	--	--	--	--	--	--	--	--	--	--
E	ST5	--	--	--	--	--	--	--	--	--	--	--
F	ST6	--	--	--	--	--	--	--	--	--	--	--
G	B	--	--	--	--	--	--	--	--	--	--	--
H	B	--	--	--	--	--	--	--	--	--	--	--

		HCT116										
	1	2	3	4	5	6	7	8	9	10	11	12
A	33816	8714	0	0	0	0	0	0	0	0	0	0
B	19433	8446	0	0	0	0	0	0	0	0	0	0
C	11592	4439	0	0	0	0	0	0	0	0	0	0
D	8046	4162	0	0	0	0	0	0	0	0	0	0
E	3772	0	0	0	0	0	0	0	0	0	0	0
F	2151	0	0	0	0	0	0	0	0	0	0	0
G	346	0	0	0	0	0	0	0	0	0	0	0
H	381	0	0	0	0	0	0	0	0	0	0	0

STANDARDS	OD	pg/ml	Slope	Intercept	R2
St.1	33816	1000	31.84704216	2685.454682	0.9899
St.2	19433	500			
St.3	11592	250			
St.4	8046	125			
St.5	3772	62.5			
St.6	2151	31.2			
St.7	346				
	381				

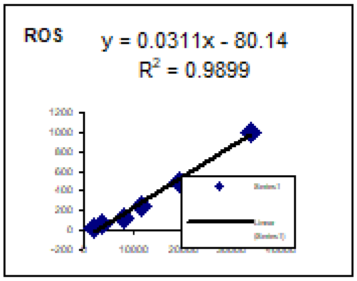

Plate map HCT116

	**1**	**2**
A	St.1	**s10**
B	St.2	**s10**
C	St.3	**C**
D	St.4	**C**
E	St.5	--
F	St.6	--
G	B	--
H	B	--

Samples FL

	**1**	**2**
A	33816	8714
B	19433	8446
C	11592	4439
D	8046	4162

E	3772	0
F	2151	0
G	346	0
H	381	0

results

	1	2
A	978	190
B	527	182
C	281	56
D	169	47
E	35	-83
F	-16	-83
G	-73	-83
H	-72	-83

Table S10. Raw RT-PCR data showing the relative expression analysis of apoptosis-related genes (caspase-3, caspase-9, Bax, and Bcl-2) in morpholine-triazine derivative **10**-treated and untreated HCT-116 cells, including Ct values, ΔCt, ΔΔCt, and fold-change calculations.

**Sample**	**Gene Expression**		
	Control cells	Test cells	FLD

Ser	code	Conc	GAPDH	Casp 3	ΔCTC	GAPDH	Casp 3	ΔCTE	ΔΔ CT	2^ ΔΔCT
			HC	TC	TC-HC	HE	TE	TE-HE	ΔCTE-ΔCTC	E=2.0
**1**	**s10/HCT116**		**19.12**	**35.77**	16.65	**19.27**	**32.64**	13.37	**-3.28**	9.7136
**2**	**C.HCT116**		**19.12**	**35.77**	16.65	**19.12**	**35.77**	16.65	**0**	1

Ser	code	Conc	GAPDH	Casp 9	ΔCTC	GAPDH	Casp 9	ΔCTE	ΔΔ CT	2^ ΔΔCT
			HC	TC	TC-HC	HE	TE	TE-HE	ΔCTE-ΔCTC	E=2.0
**1**	**s10/HCT116**		**19.12**	**36.35**	17.23	**19.27**	**34.07**	14.8	**-2.43**	5.3889
**2**	**C.HCT116**		**19.12**	**36.35**	17.23	**19.12**	**36.35**	17.23	**0**	1

Ser	code	Conc	GAPDH	Bax	ΔCTC	GAPDH	Bax	ΔCTE	ΔΔ CT	2^ ΔΔCT
			HC	TC	TC-HC	HE	TE	TE-HE	ΔCTE-ΔCTC	E=2.0
**1**	**s10/HCT116**		**19.12**	**34.88**	15.76	**19.27**	**32.26**	12.99	**-2.77**	6.8211
**2**	**C.HCT116**		**19.12**	**34.88**	15.76	**19.12**	**34.88**	15.76	**0**	1

Ser	code	Conc	GAPDH	bcl2	ΔCTC	GAPDH	bcl2	ΔCTE	ΔΔ CT	2^ ΔΔCT
			HC	TC	TC-HC	HE	TE	TE-HE	ΔCTE-ΔCTC	E=2.0

**1**	**s10/HCT116**		**19.12**	**27.51**	8.39	**19.27**	**29.82**	10.55	**2.16**	0.2238
**2**	**C.HCT116**		**19.12**	**27.51**	8.39	**19.12**	**27.51**	8.39	**0**	1

Copies of IR, ^{1}H NMR, ^{13}C NMR, and mass spectra of Compounds

Characterization of Compound A:

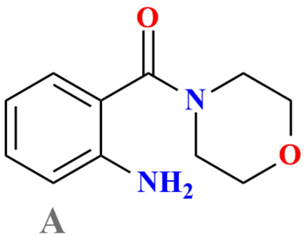

A

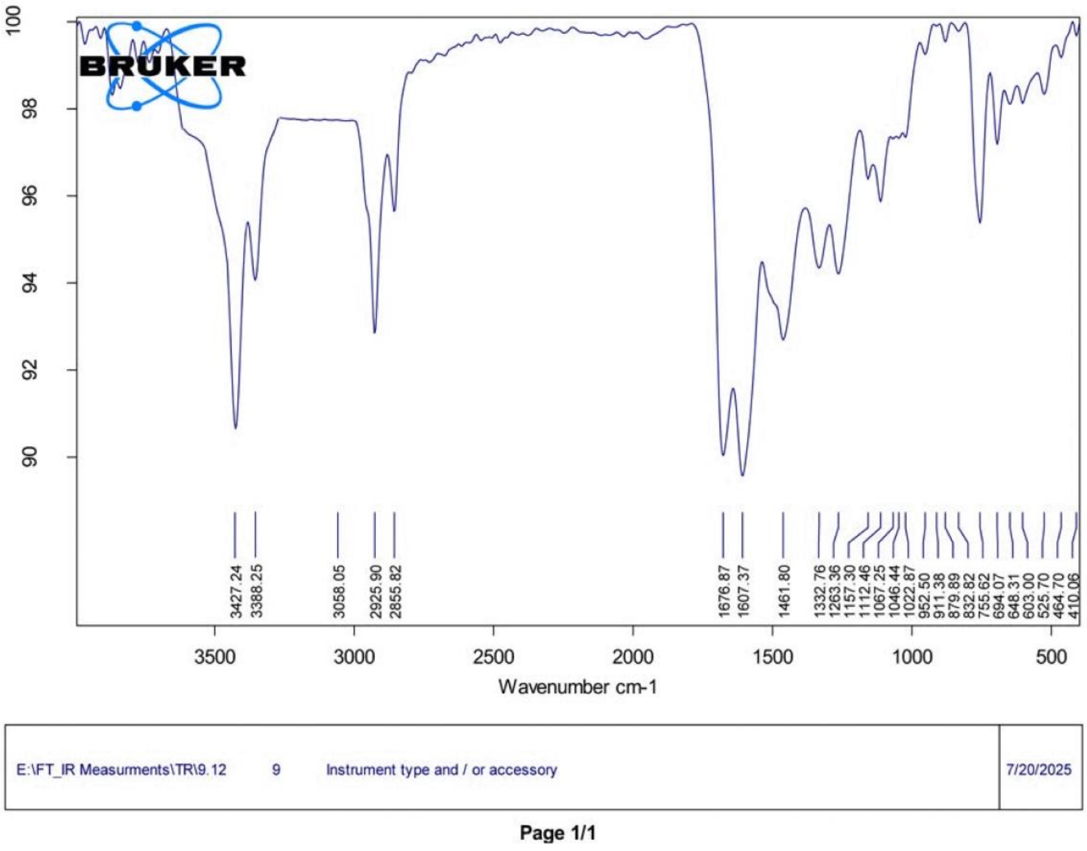

Figure S1. IR of Compound **A**

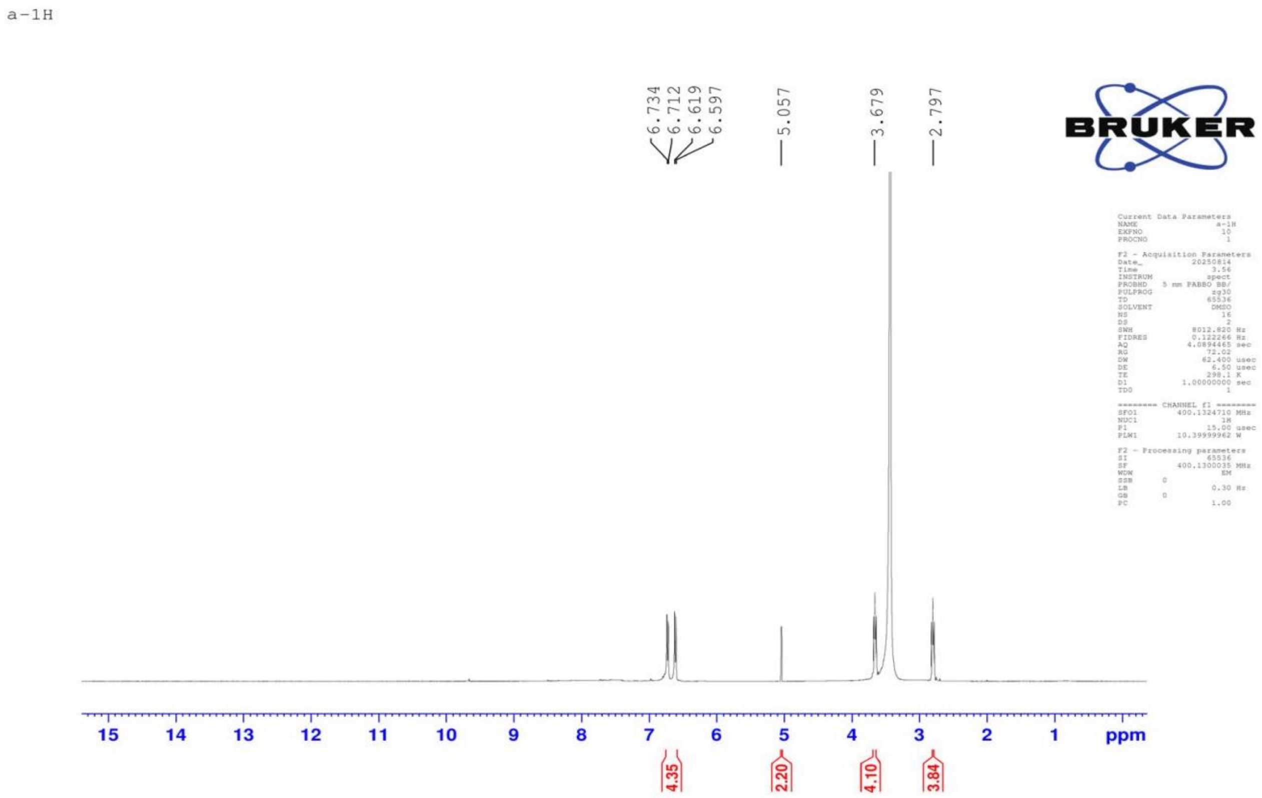

Figure S2. ^{1}H NMR spectrum (400 MHz, DMSO-*d6*) of compound **A**

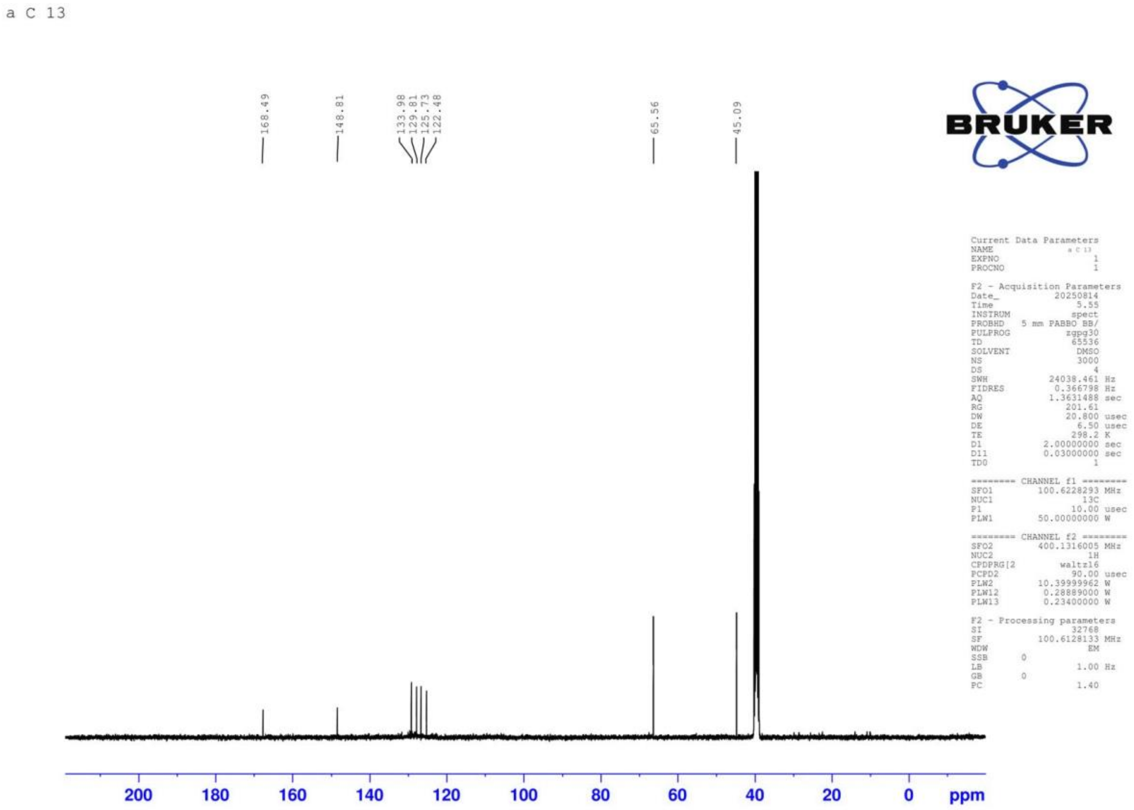

Figure S3. ^{13}C NMR spectrum (100 MHz, DMSO-*d6*) of compound **A**

Characterization of Compound B

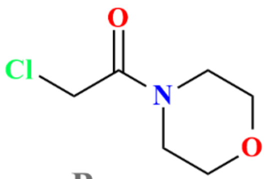

B

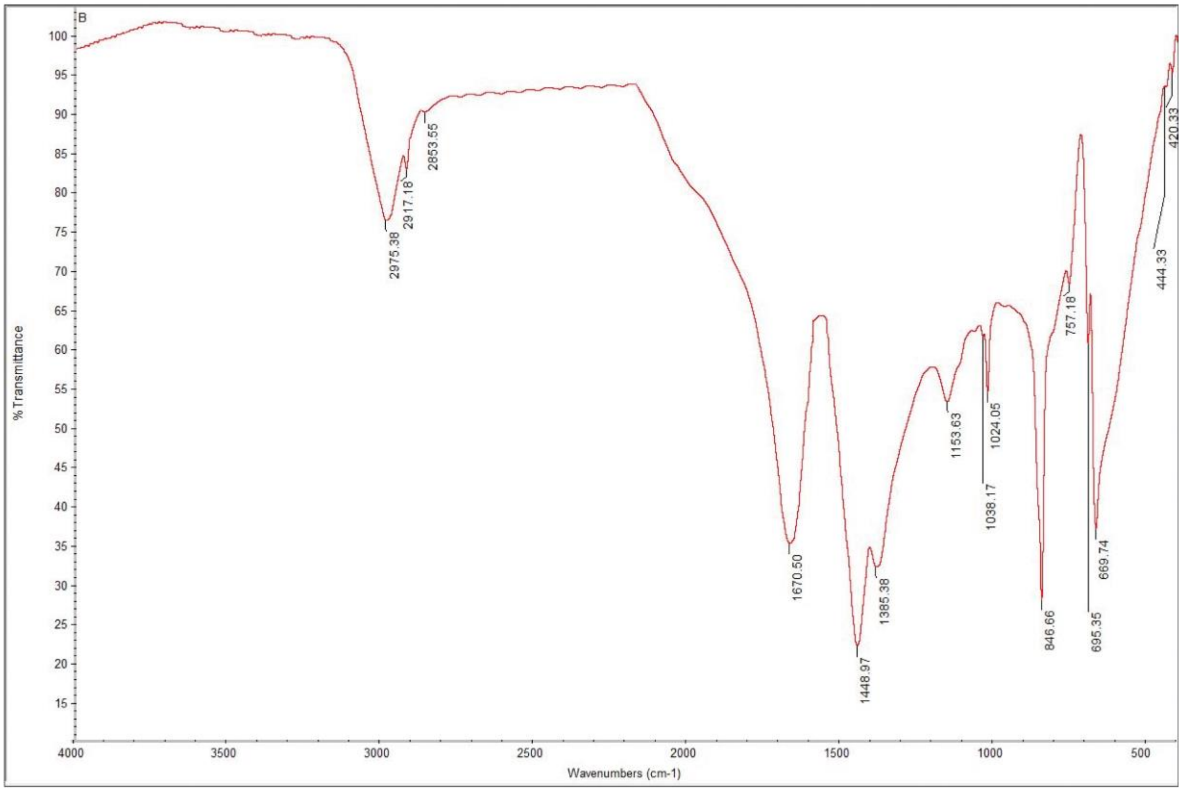

Figure S4. IR of Compound **B**

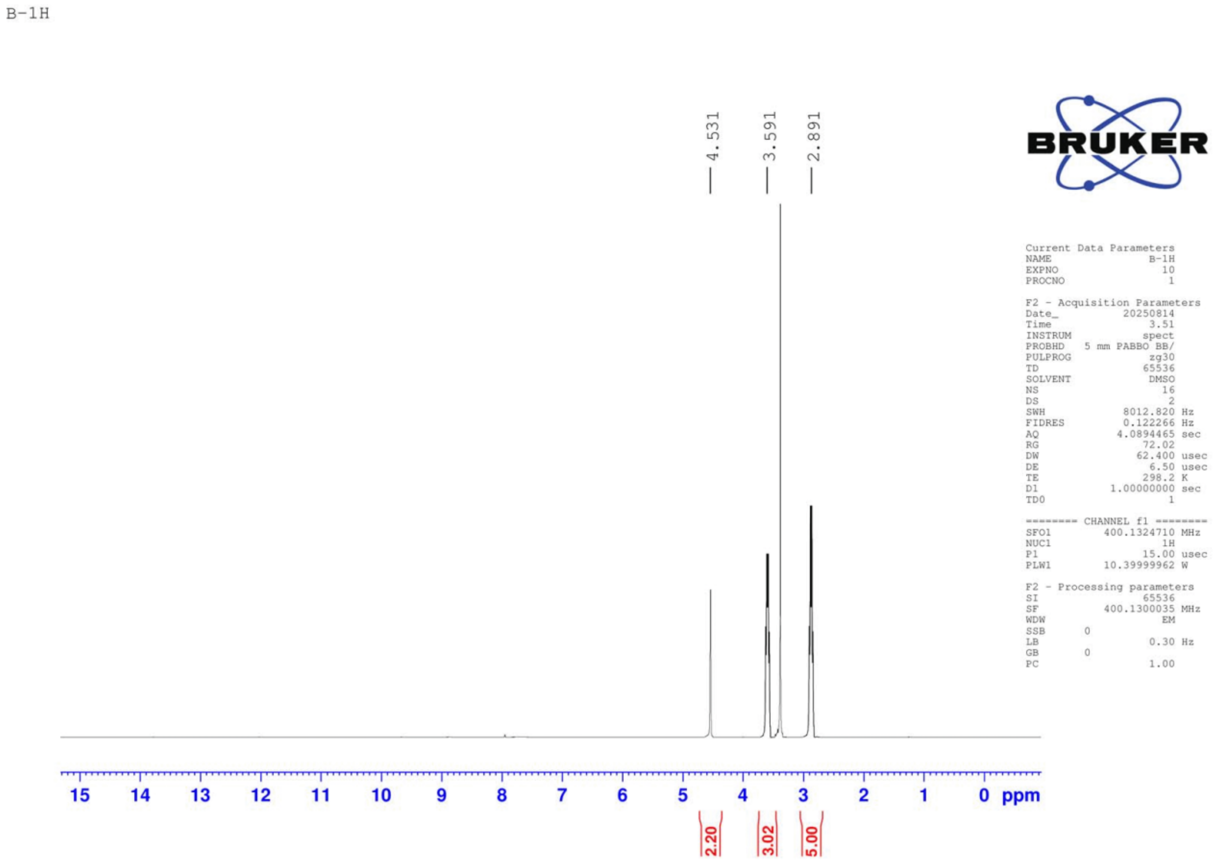

Figure S5. ^{1}H NMR spectrum (400 MHz, DMSO-*d6*) of compound **B**

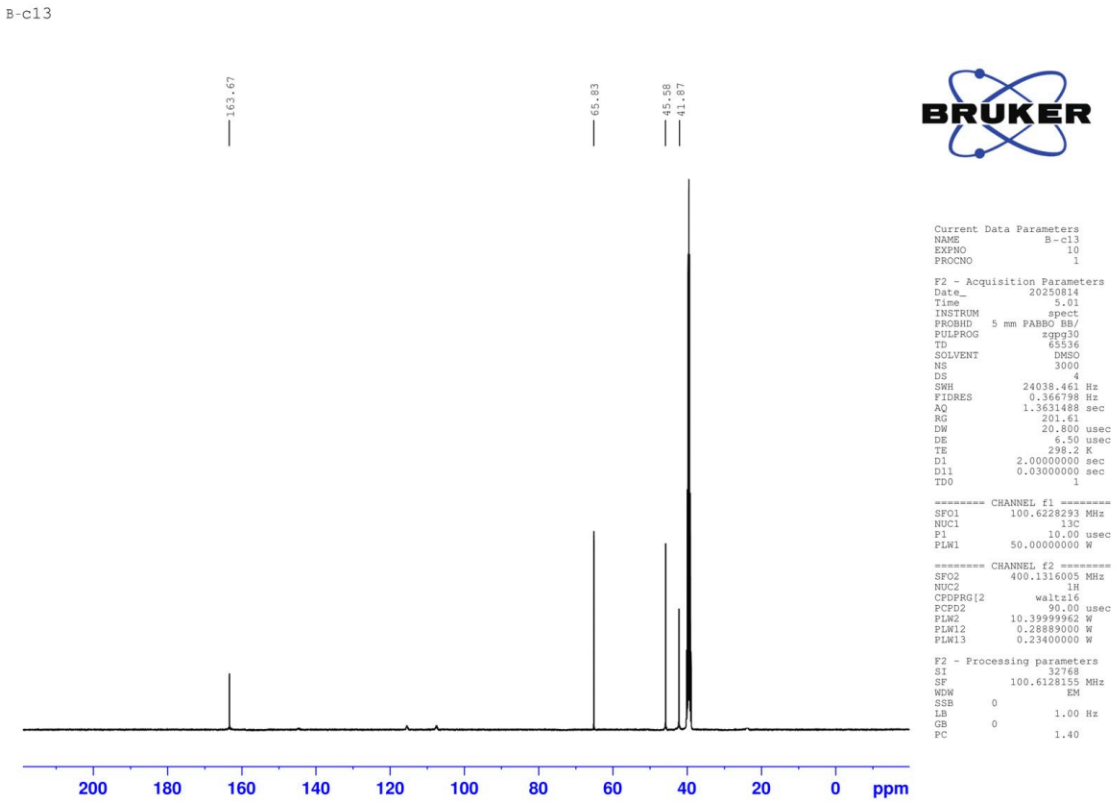

Figure S6. ^{13}C NMR spectrum (100 MHz, DMSO-*d6*) of compound **B**

Characterization of Compound 1

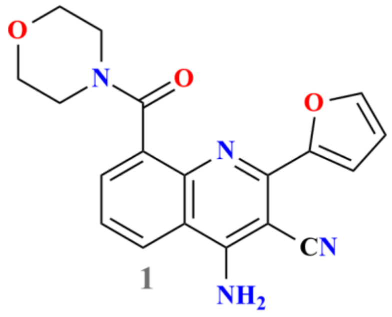

1

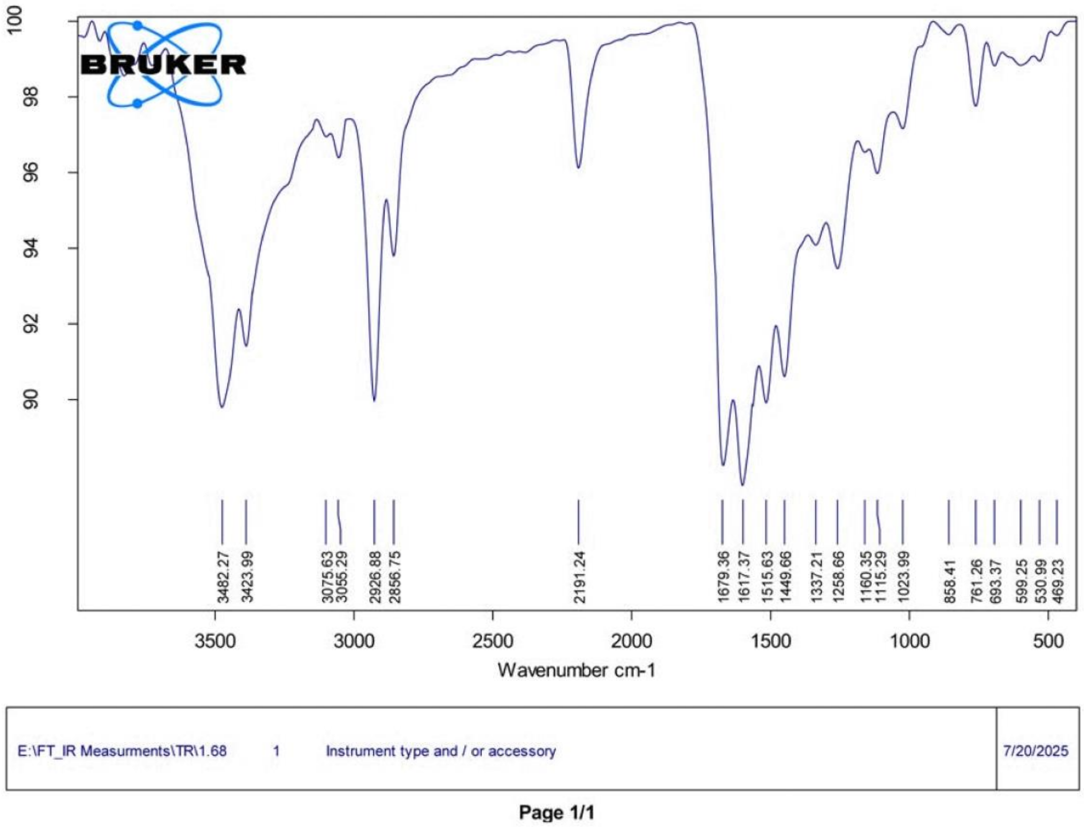

Figure S7. IR of Compound **1**

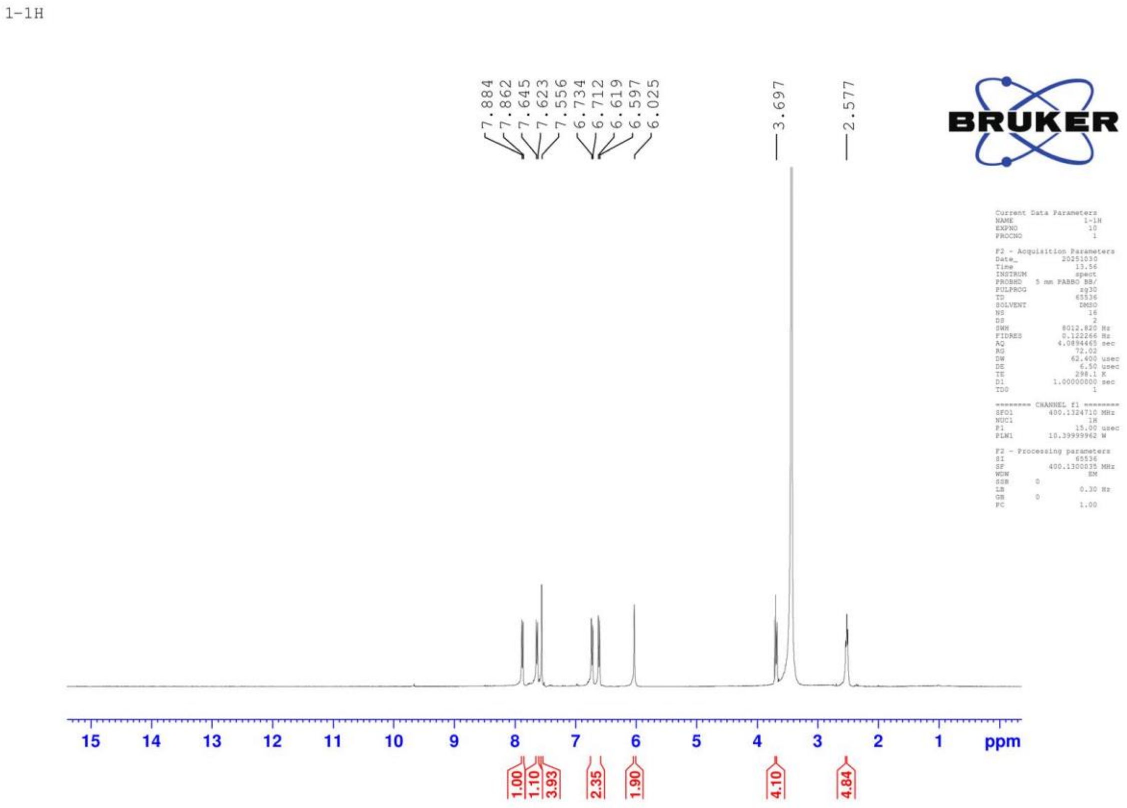

Figure S8. ^{1}H NMR spectrum (400 MHz, DMSO-*d6*) of compound **1**

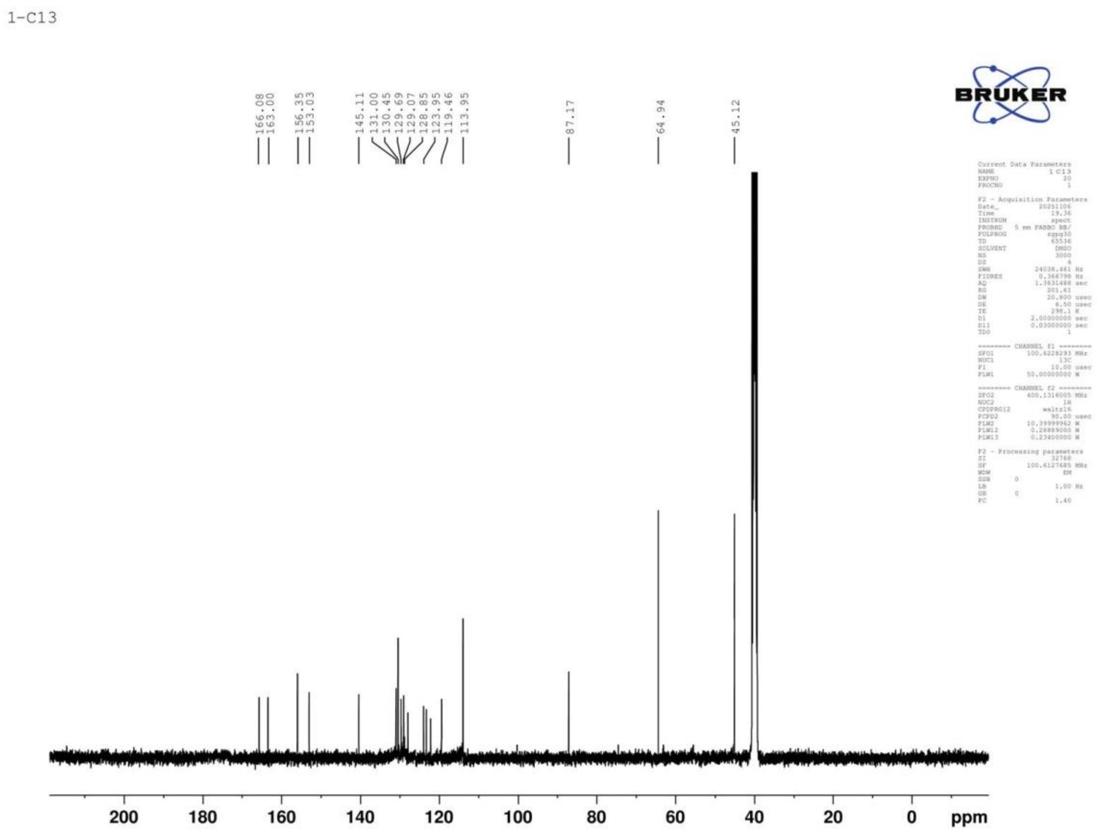

Figure S9. ^{13}C NMR spectrum (100 MHz, DMSO-*d6*) of compound **1**

Characterization of Compound 2

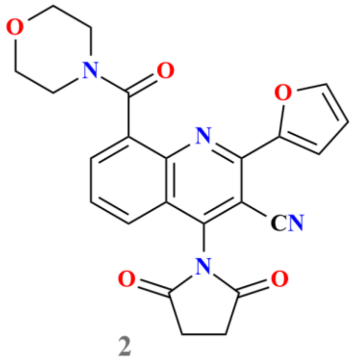

2

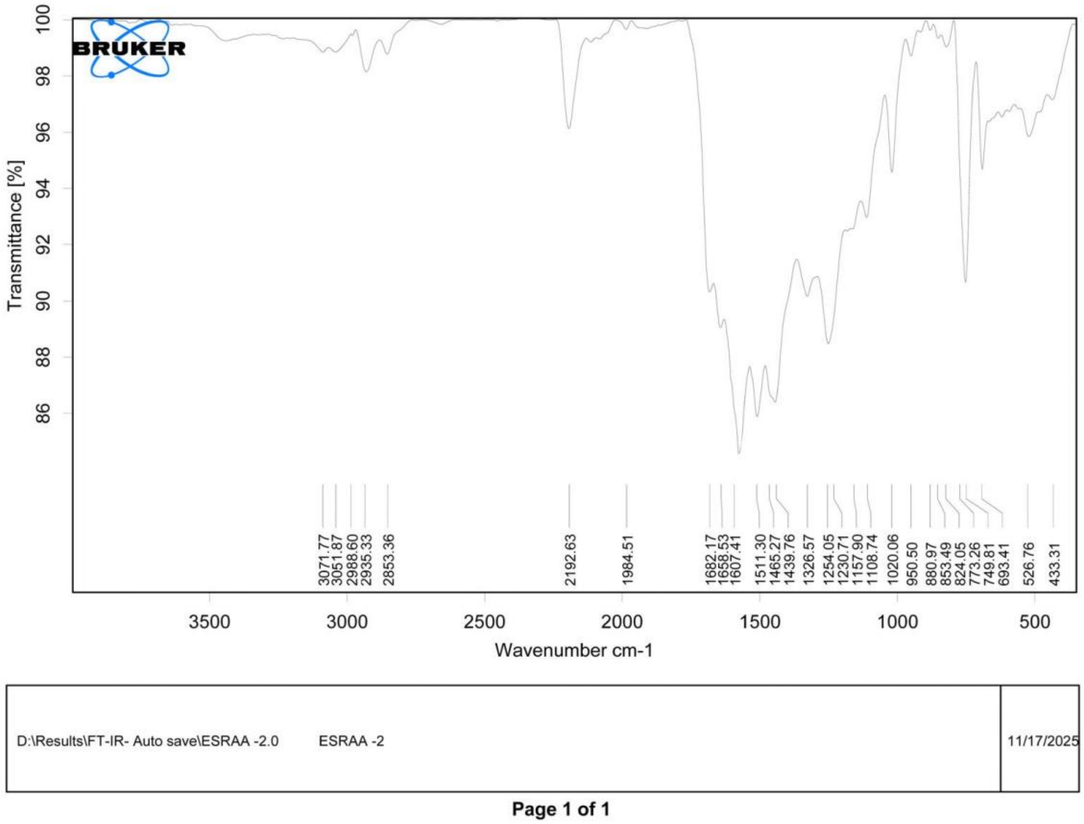

Figure S10. IR of Compound **2**

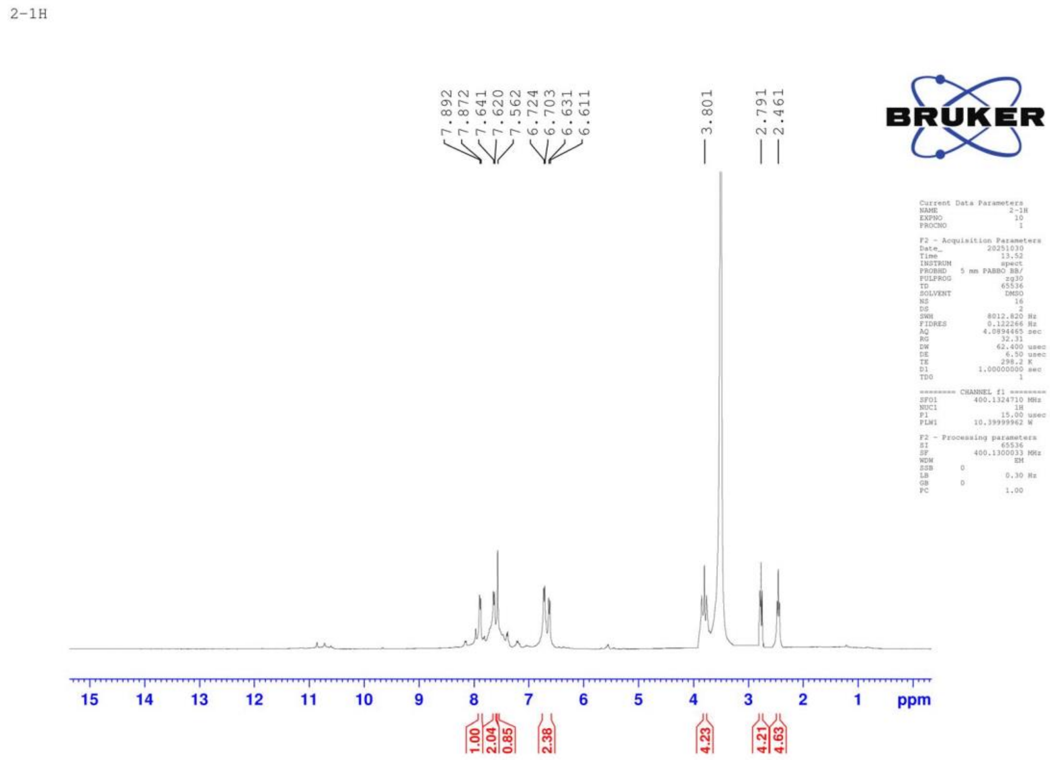

Figure S11. ^{1}H NMR spectrum (400 MHz, DMSO-*d6*) of compound **2**

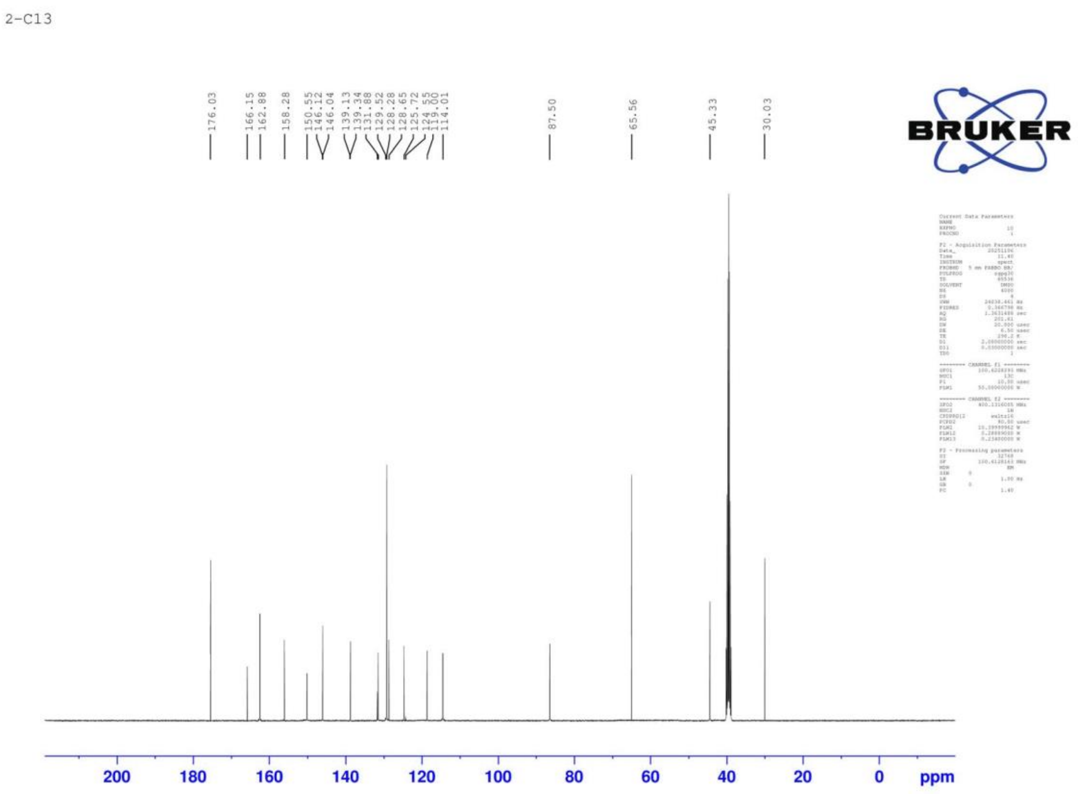

Figure S12. ^{13}C NMR spectrum (100 MHz, DMSO-*d6*) of compound **2**

Characterization of Compound 3

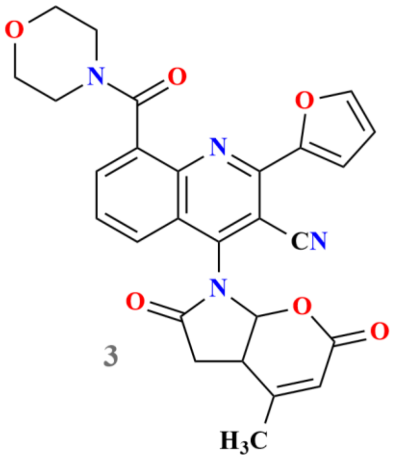

3

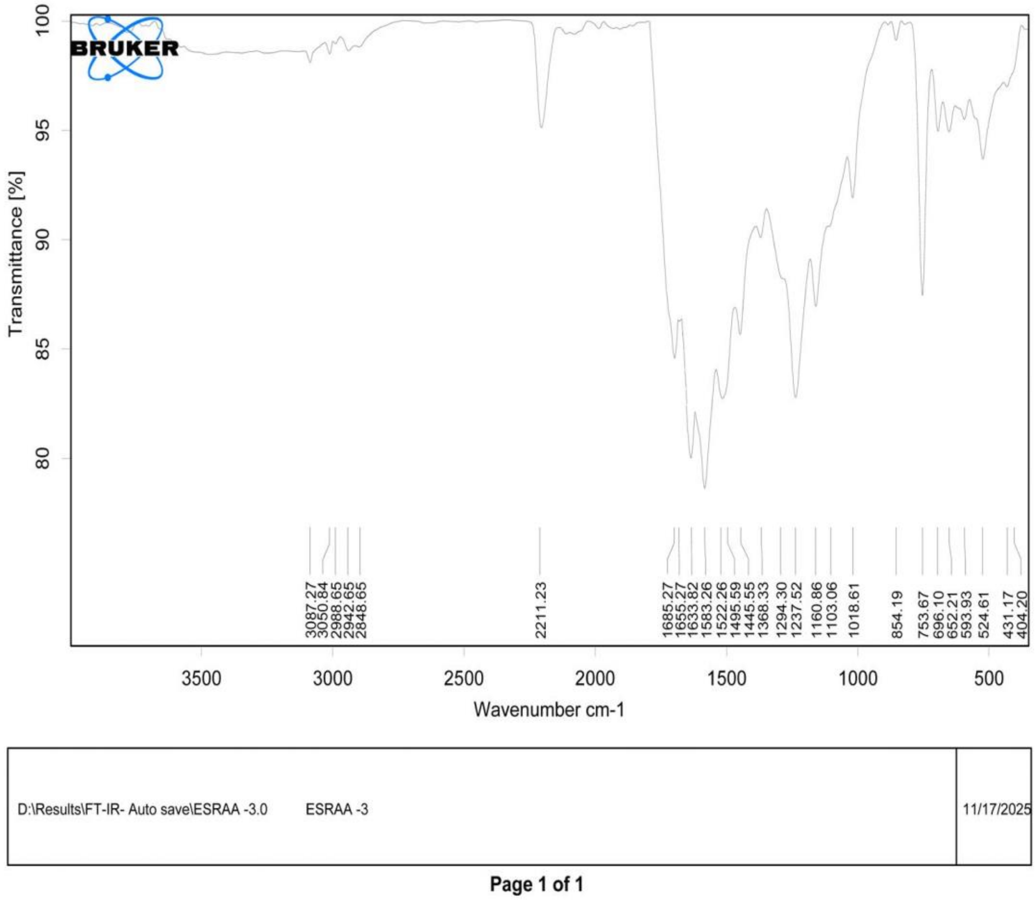

Figure S13. IR of Compound **3**

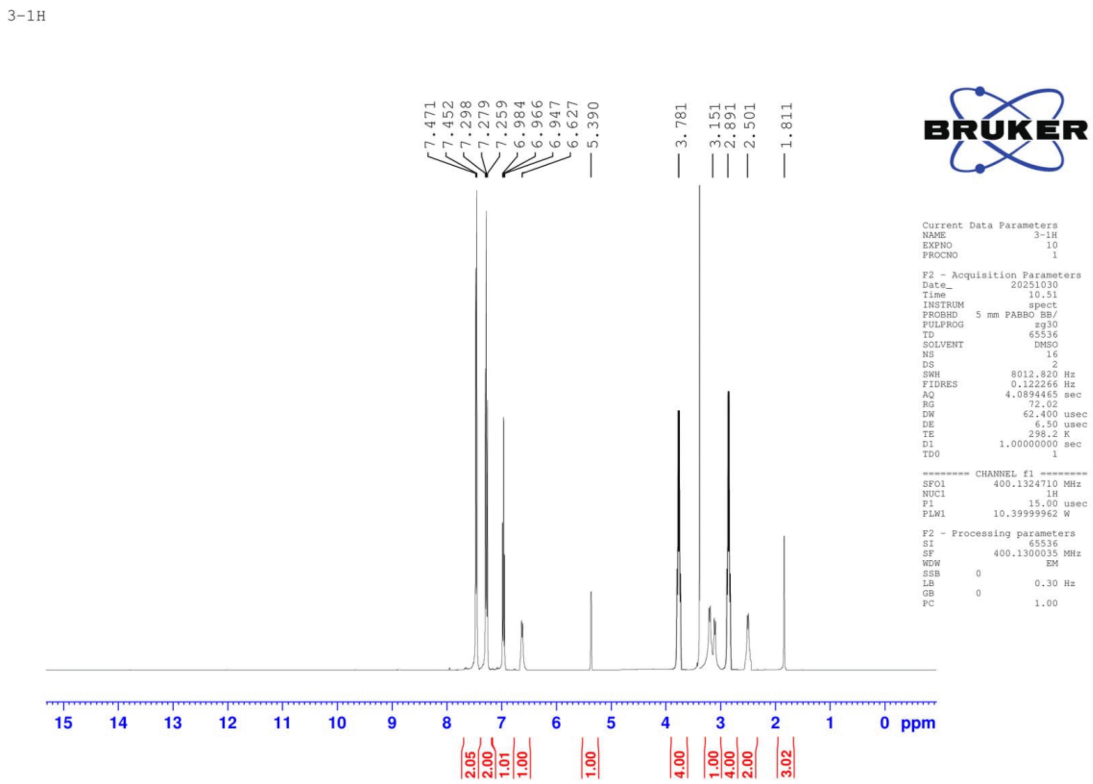

Figure S14. ^{1}H NMR spectrum (400 MHz, DMSO-*d6*) of compound **3**

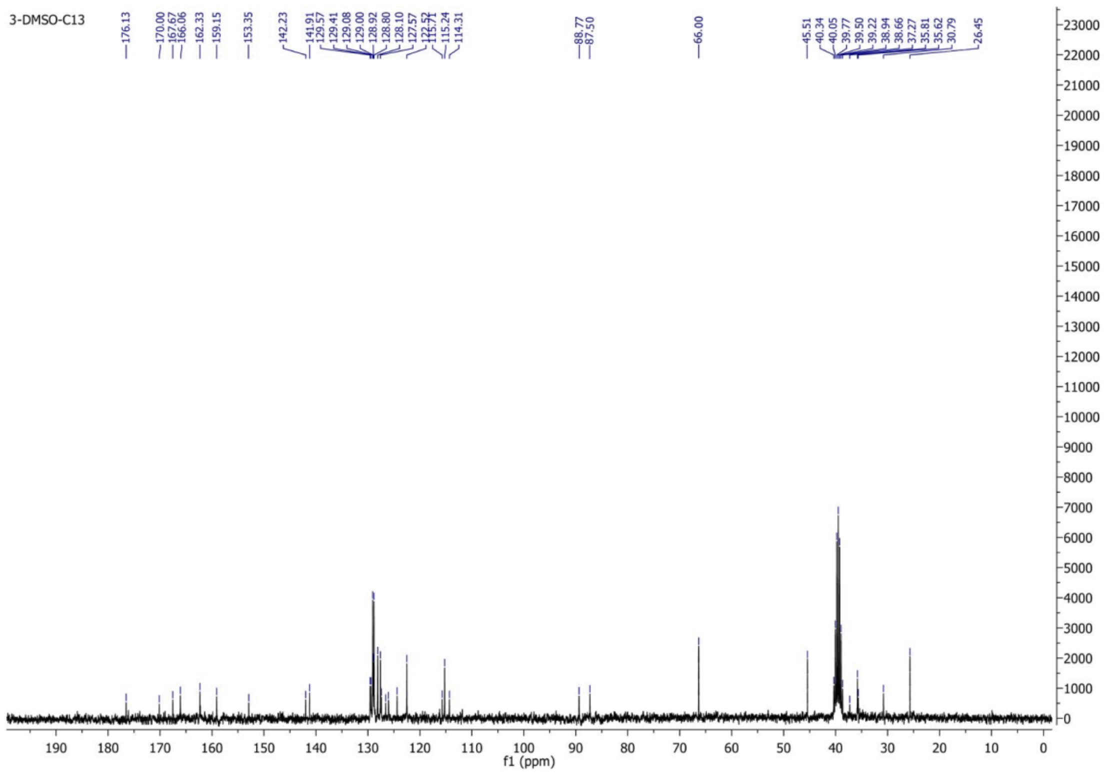

Figure S15. ^{13}C NMR spectrum (100 MHz, DMSO-*d6*) of compound **3**

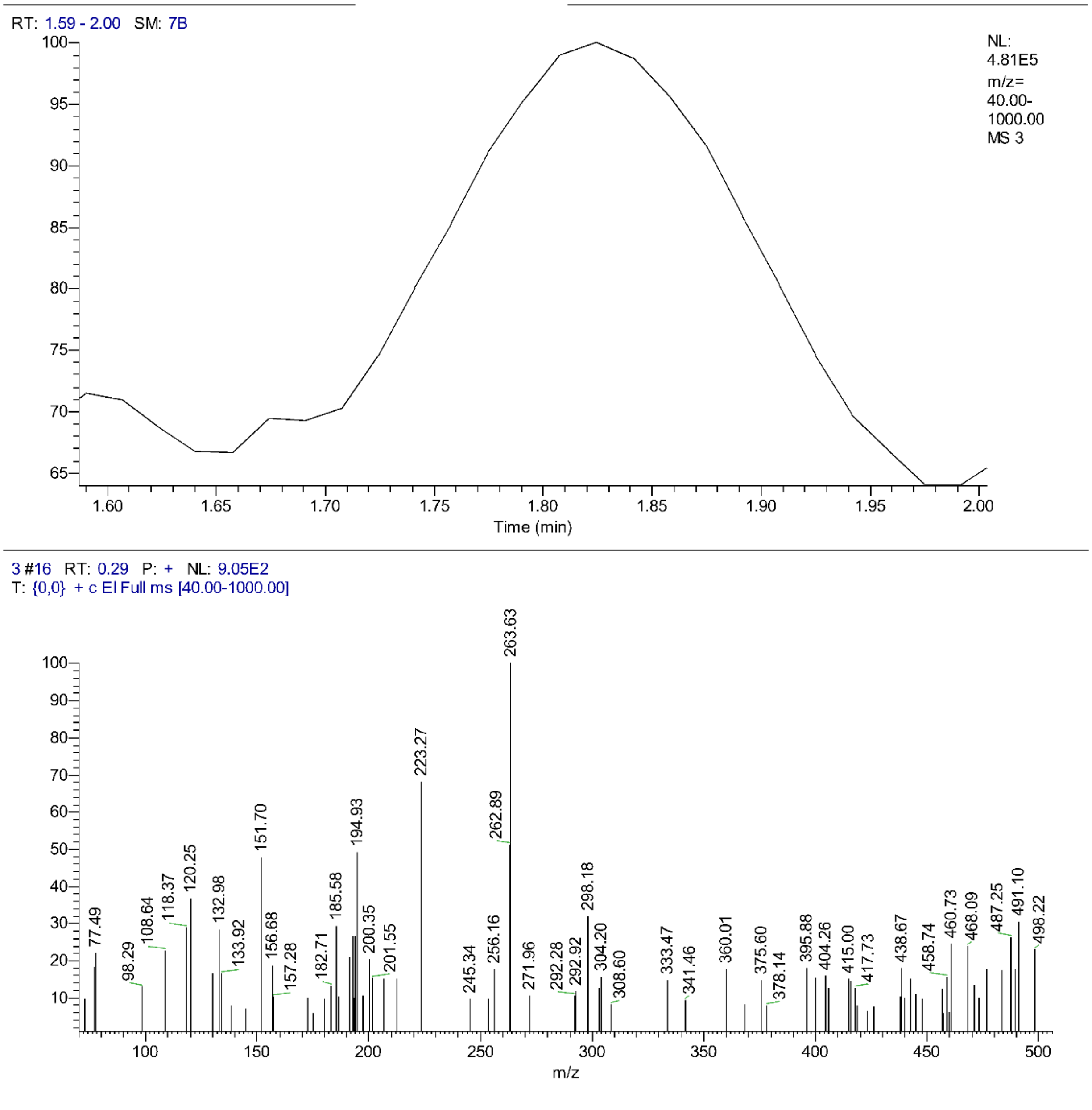

Figure S16. Mass spectrum of compound **3**

Characterization of Compound 4

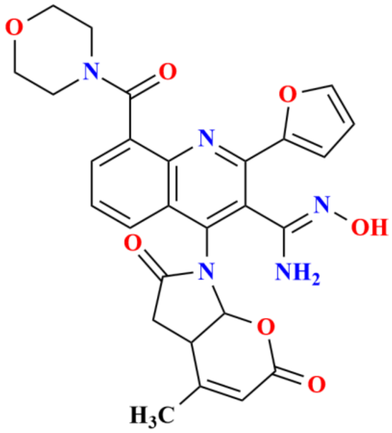

4

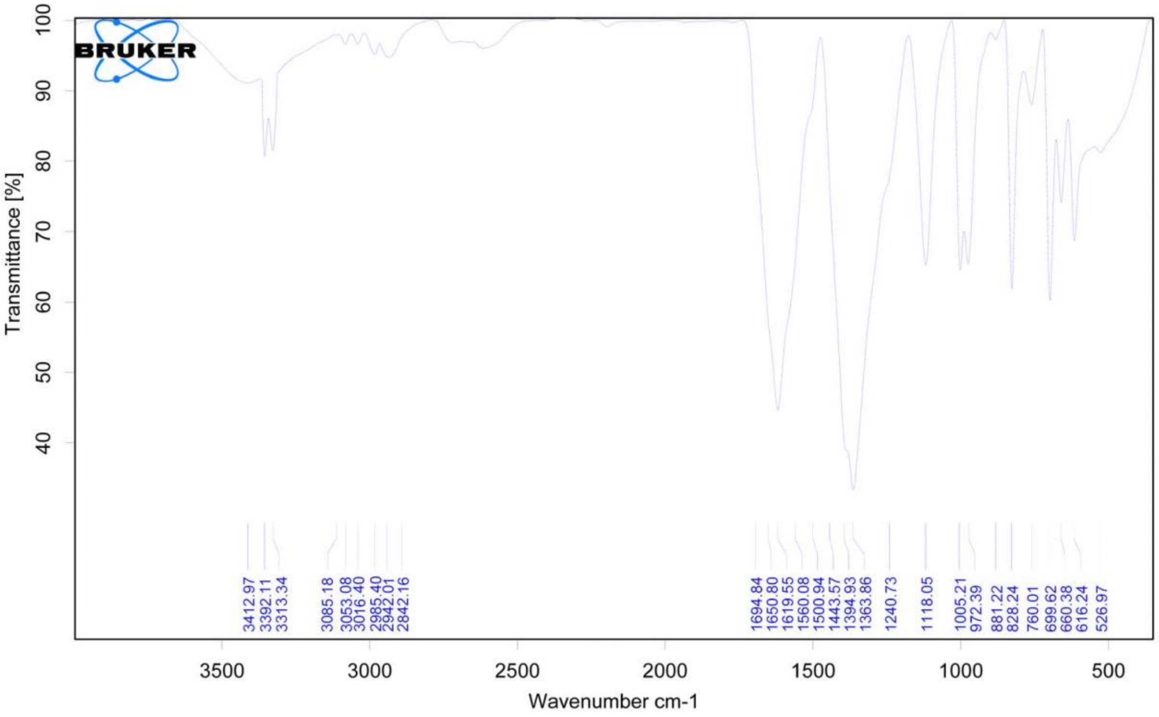

D:\Results\FT-IR- Auto save\ESRAA --4.0 ESRAA --4	11/17/2025

Page 1 of 1

Figure S17. IR of Compound **4**

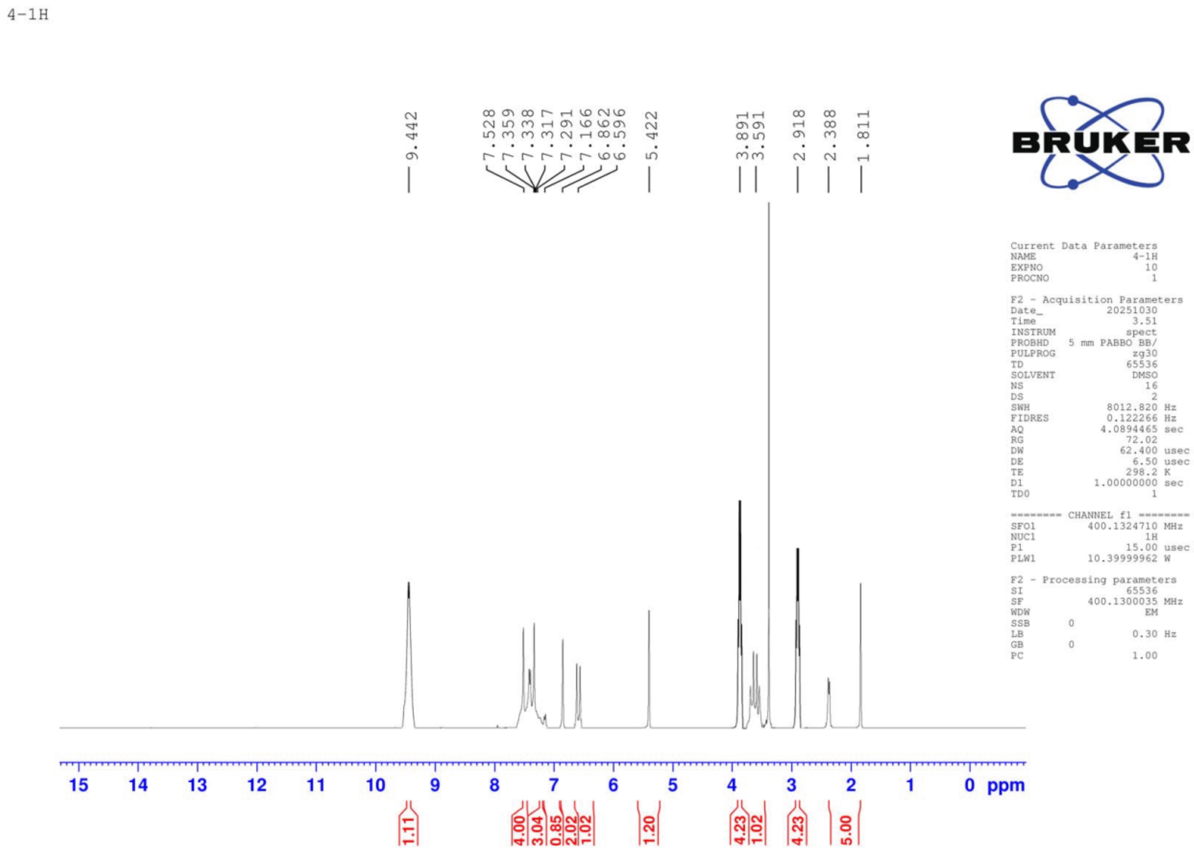

Figure S18. ^{1}H NMR spectrum (400 MHz, DMSO-*d6*) of compound **4**

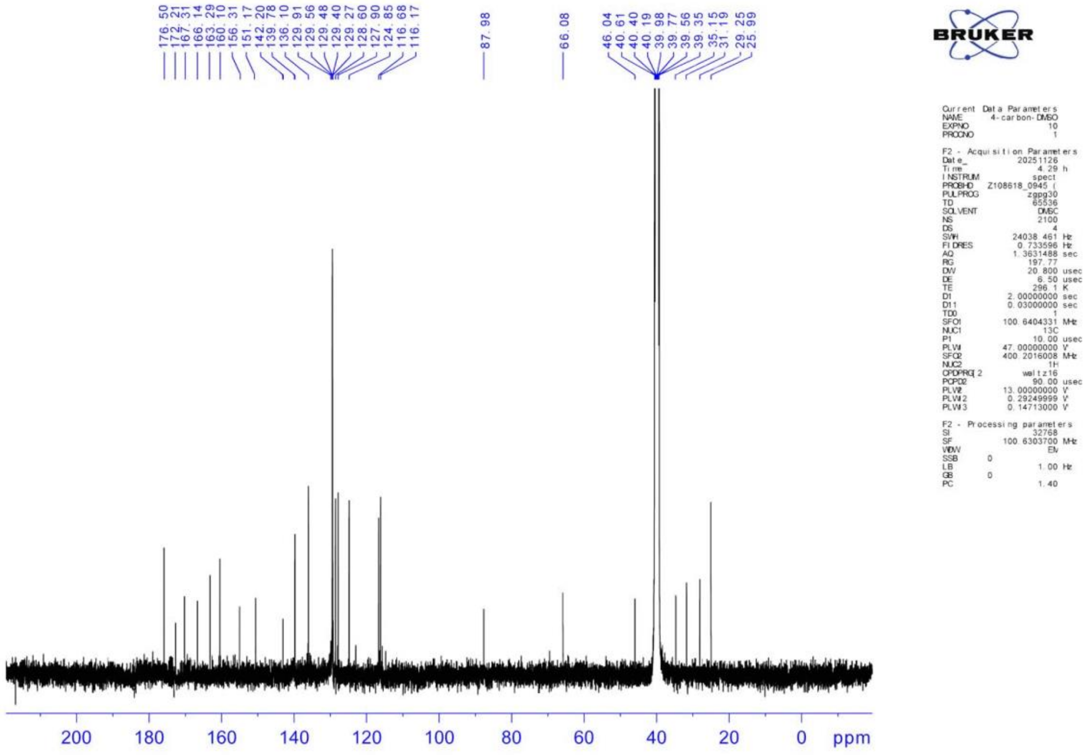

Figure S19. ^{13}C NMR spectrum (100 MHz, DMSO-*d6*) of compound **4**

Characterization of Compound 5

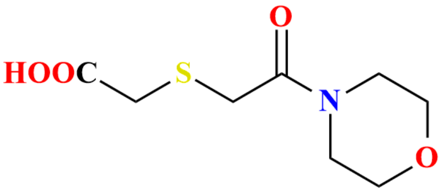

5

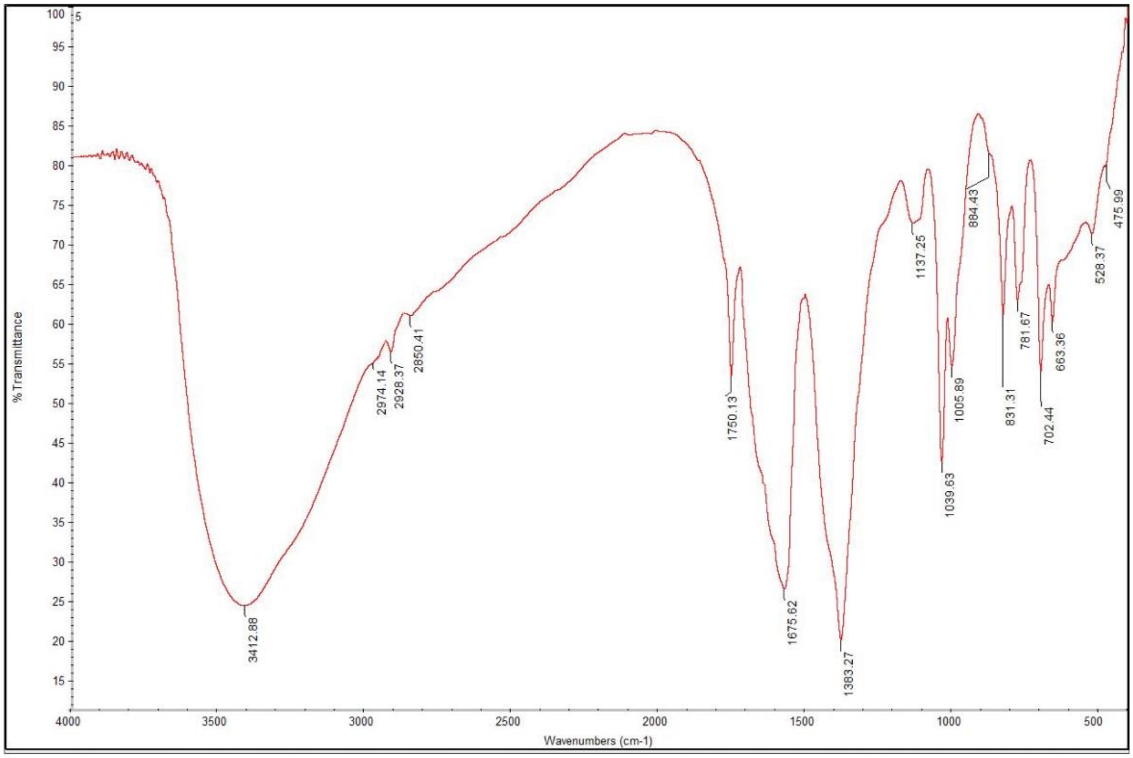

Figure S20. IR of Compound **5**

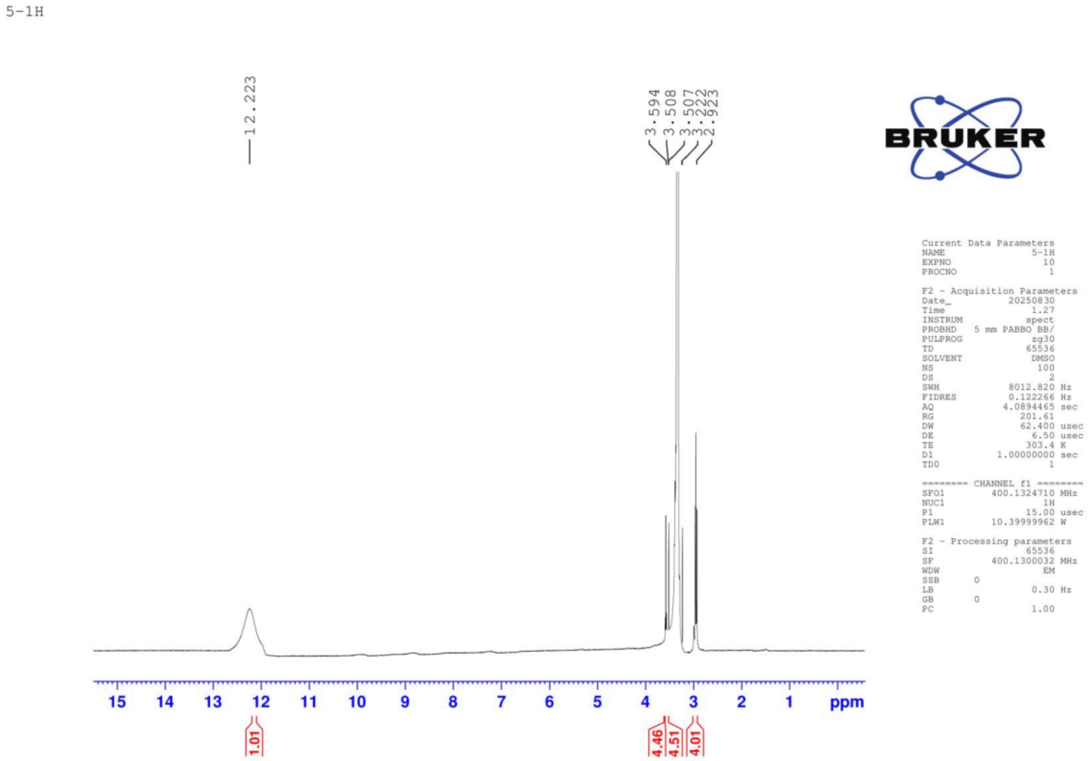

Figure S21. ^{1}H NMR spectrum (400 MHz, DMSO-*d6*) of compound **5**

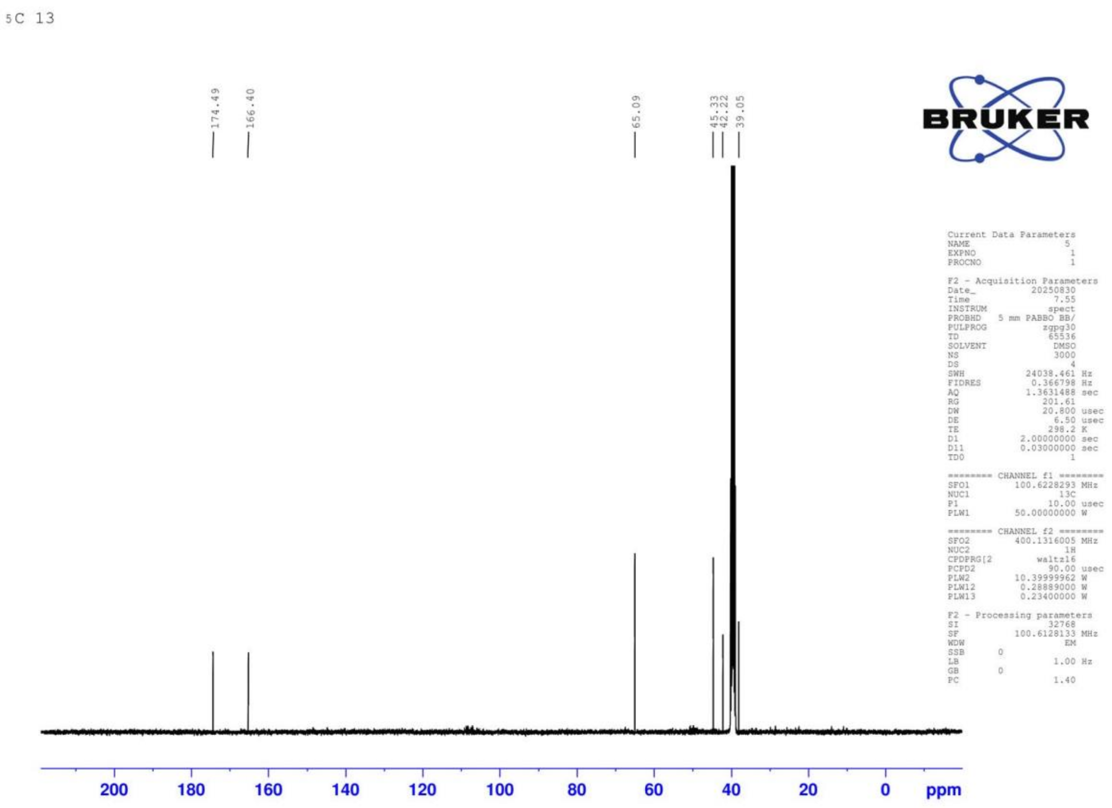

Figure S22. ^{13}C NMR spectrum (100 MHz, DMSO-*d6*) of compound **5**

Characterization of Compound 6

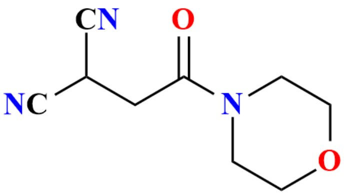

6

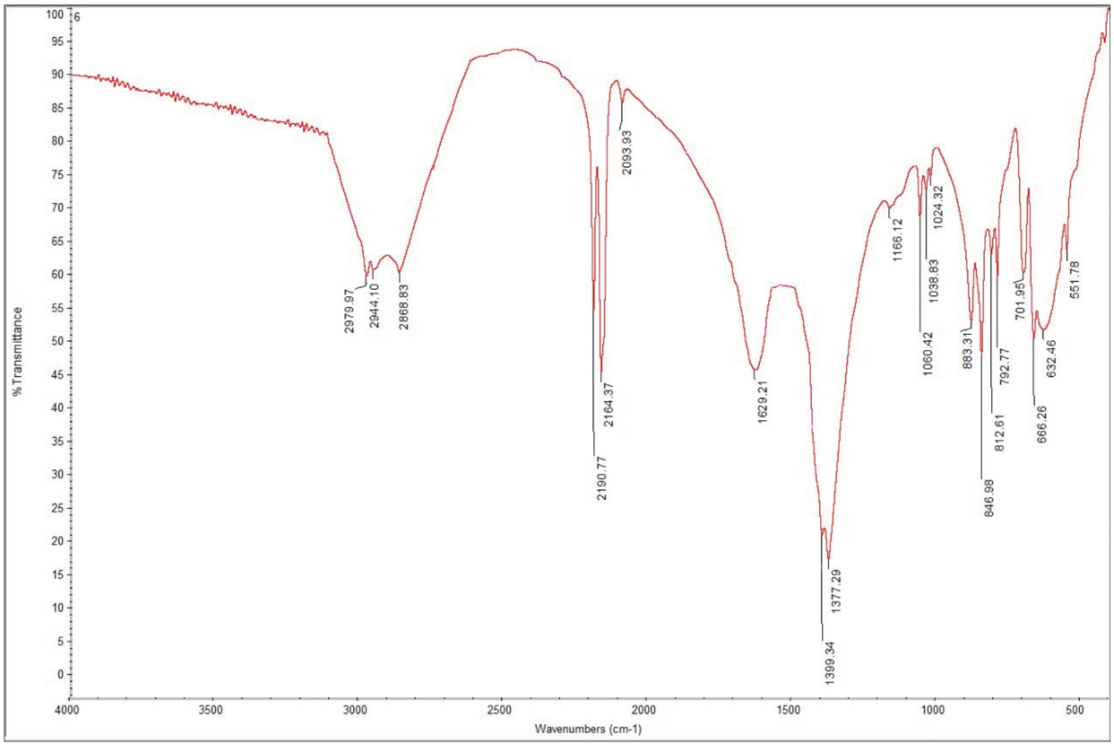

Figure S23. IR of Compound **6**

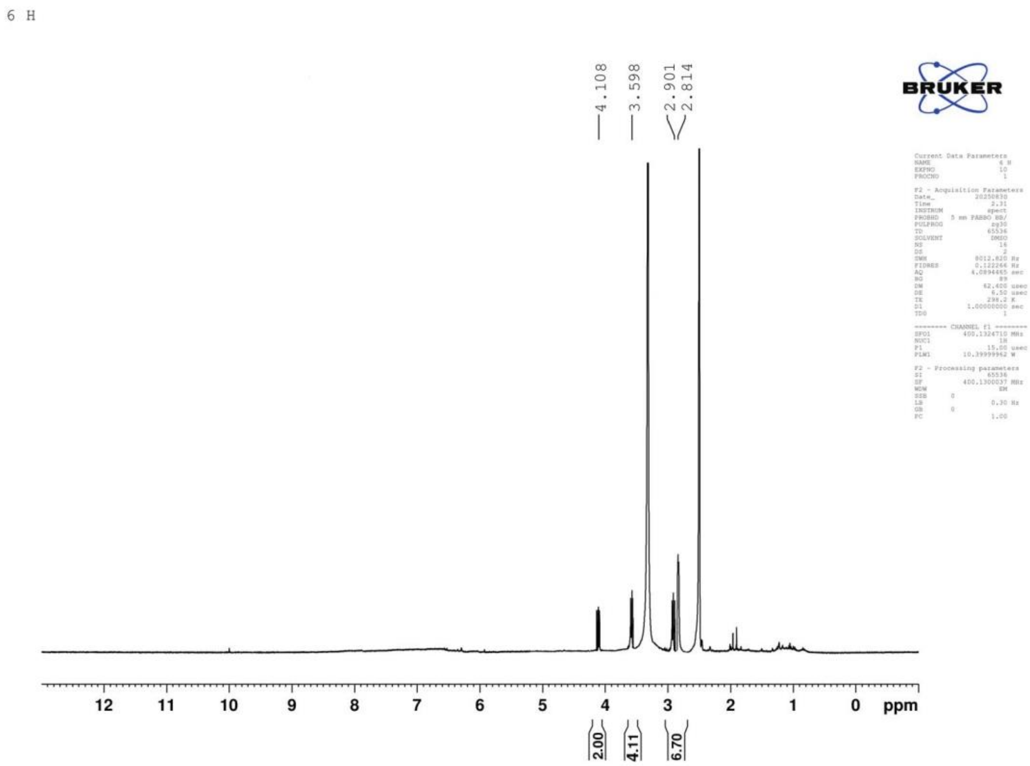

Figure S24. ^{1}H NMR spectrum (400 MHz, DMSO-*d6*) of compound **6**

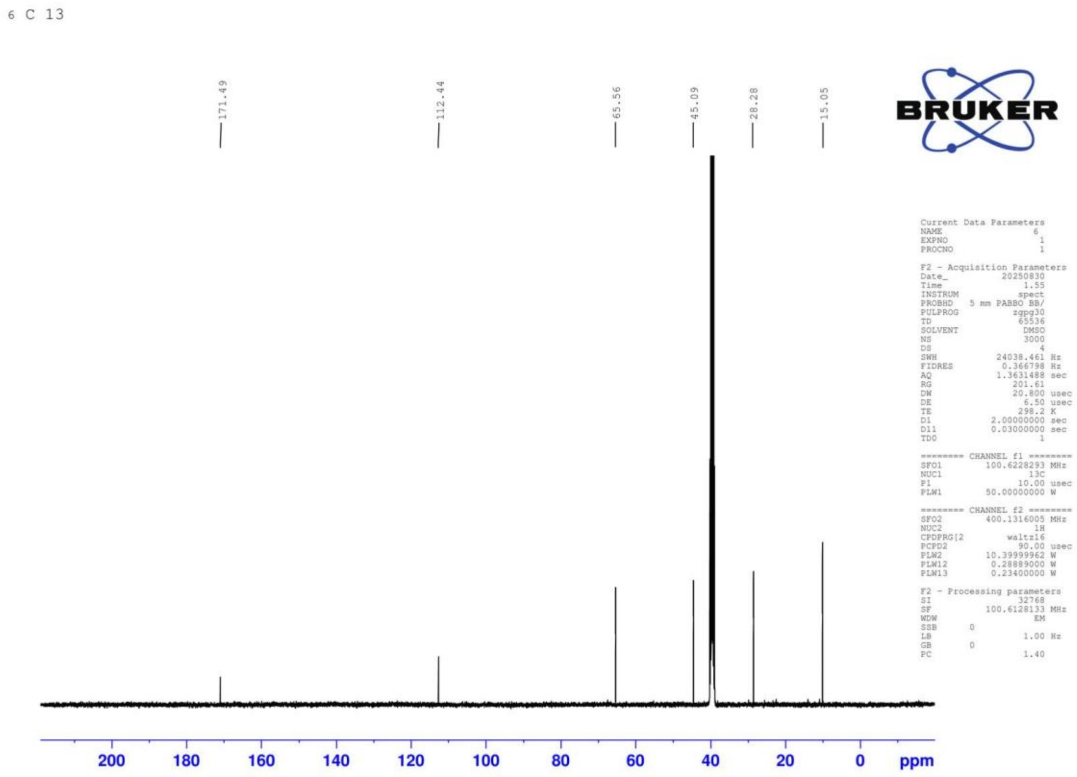

Figure S25. ^{13}C NMR spectrum (100 MHz, DMSO-*d6*) of compound **6**

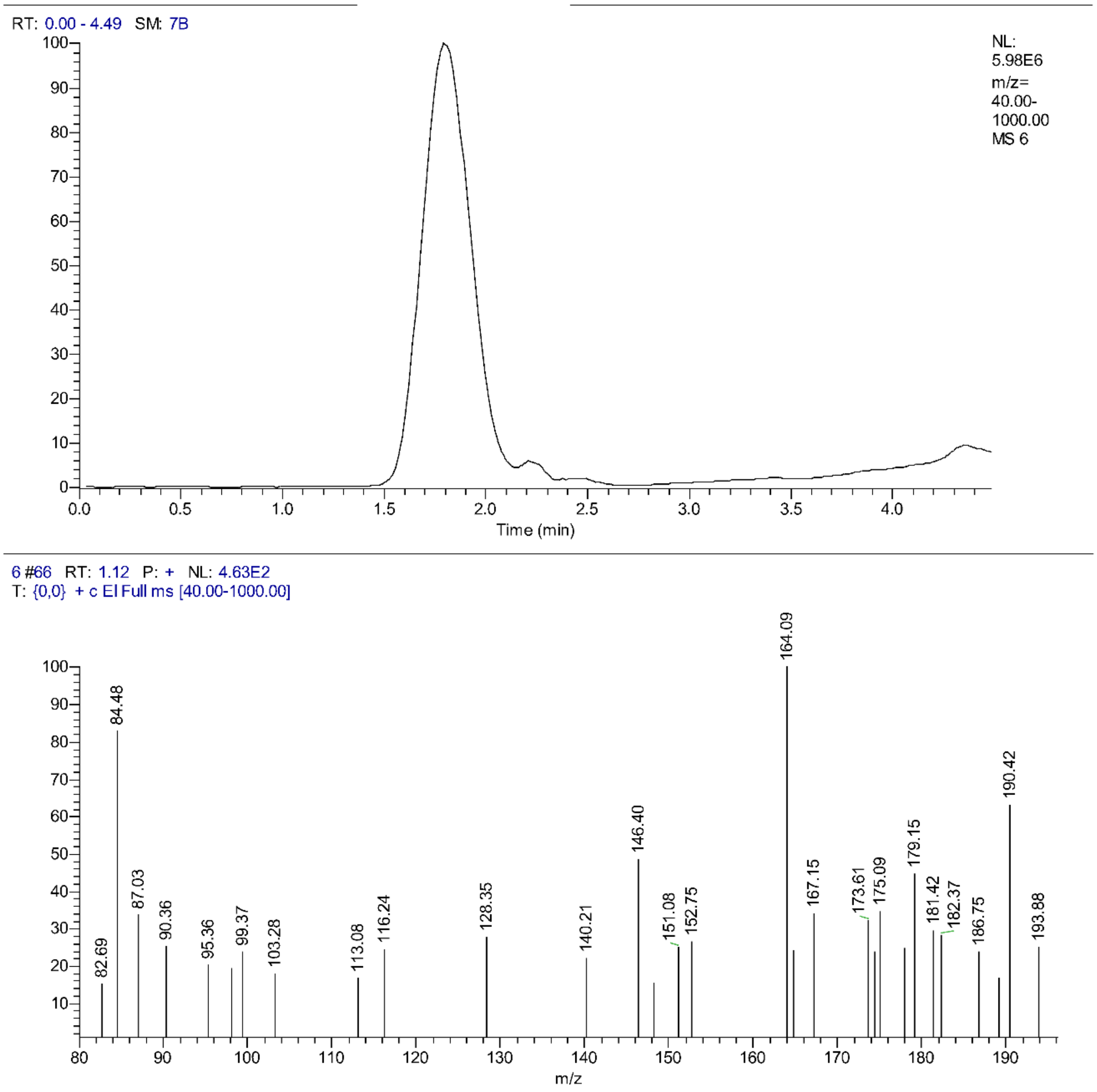

Figure S26. Mass spectrum of compound **6**

Characterization of Compound 7

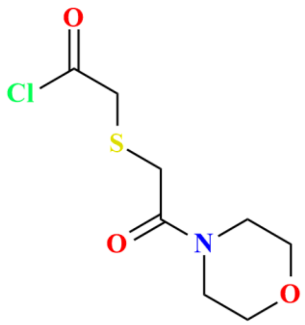

7

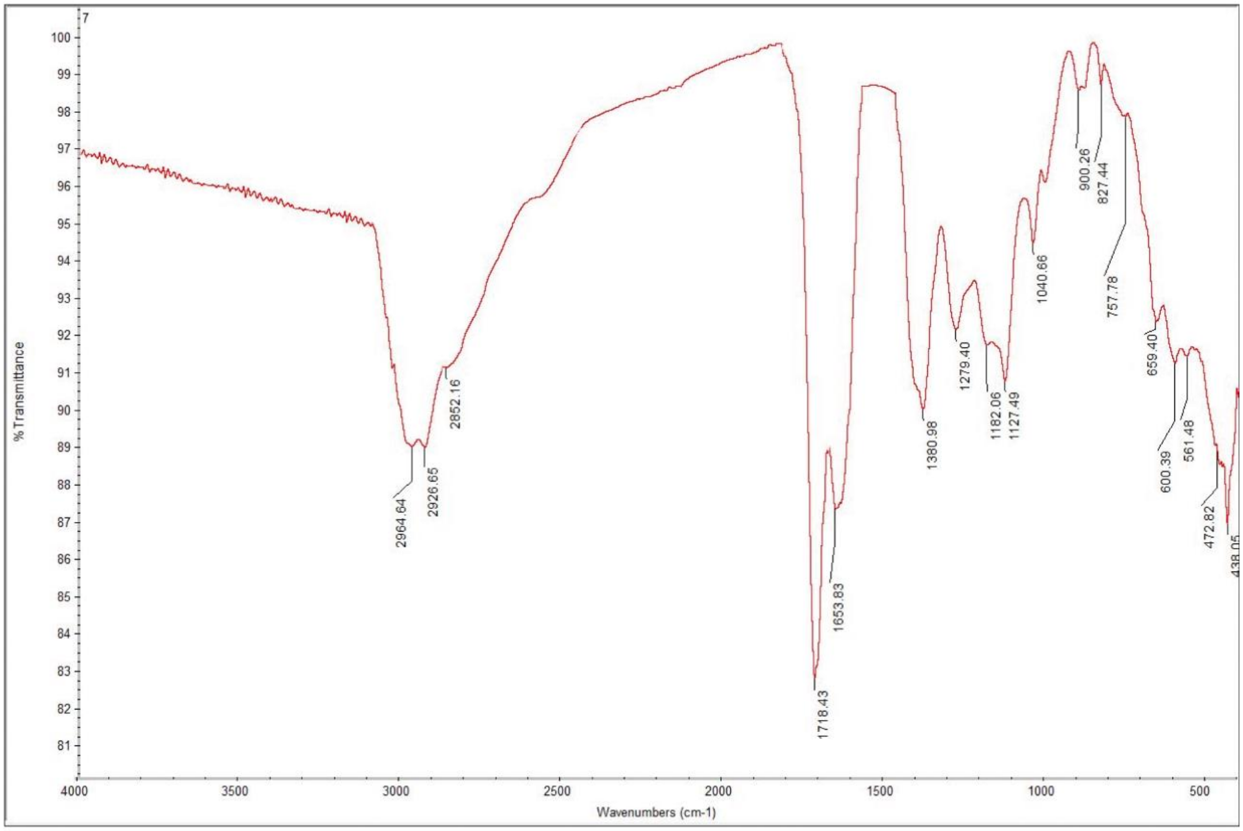

Figure S27. IR of Compound **7**

7-1H

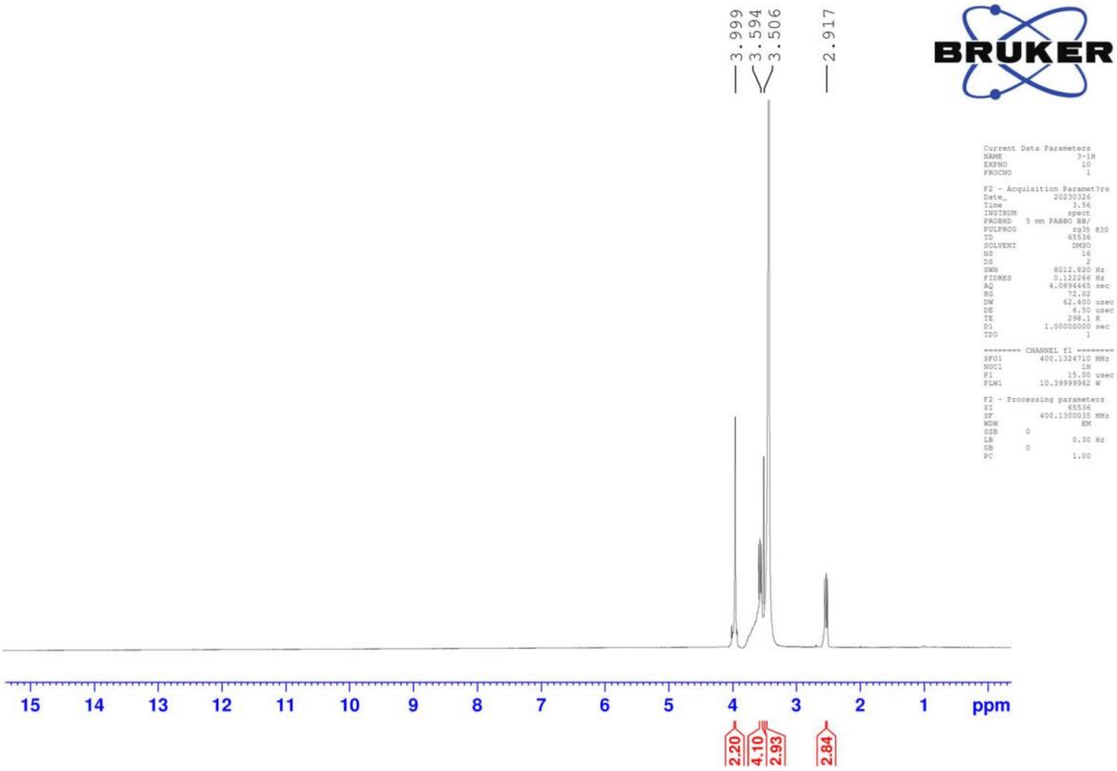

Figure S28. ^{1}H NMR spectrum (400 MHz, DMSO-*d6*) of compound **7**

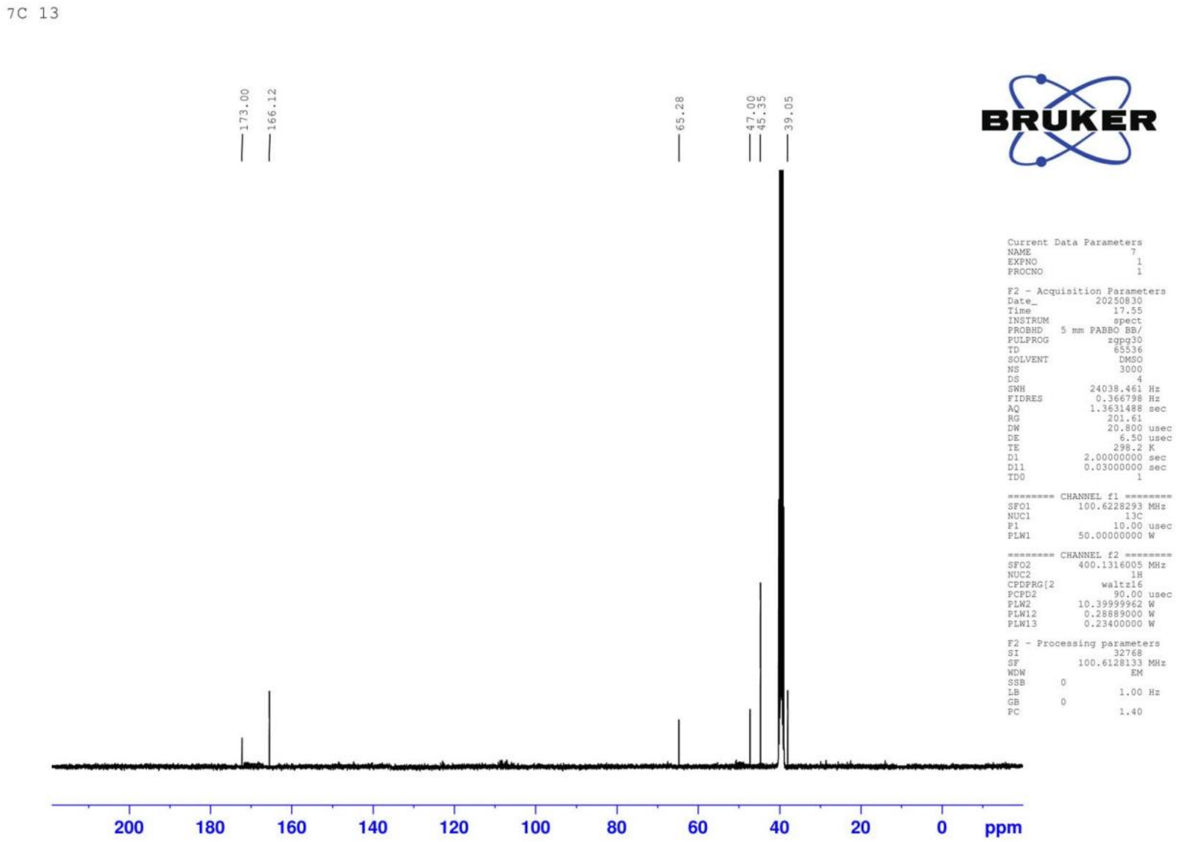

Figure S29. ^{13}C NMR spectrum (100 MHz, DMSO-*d6*) of compound **7**

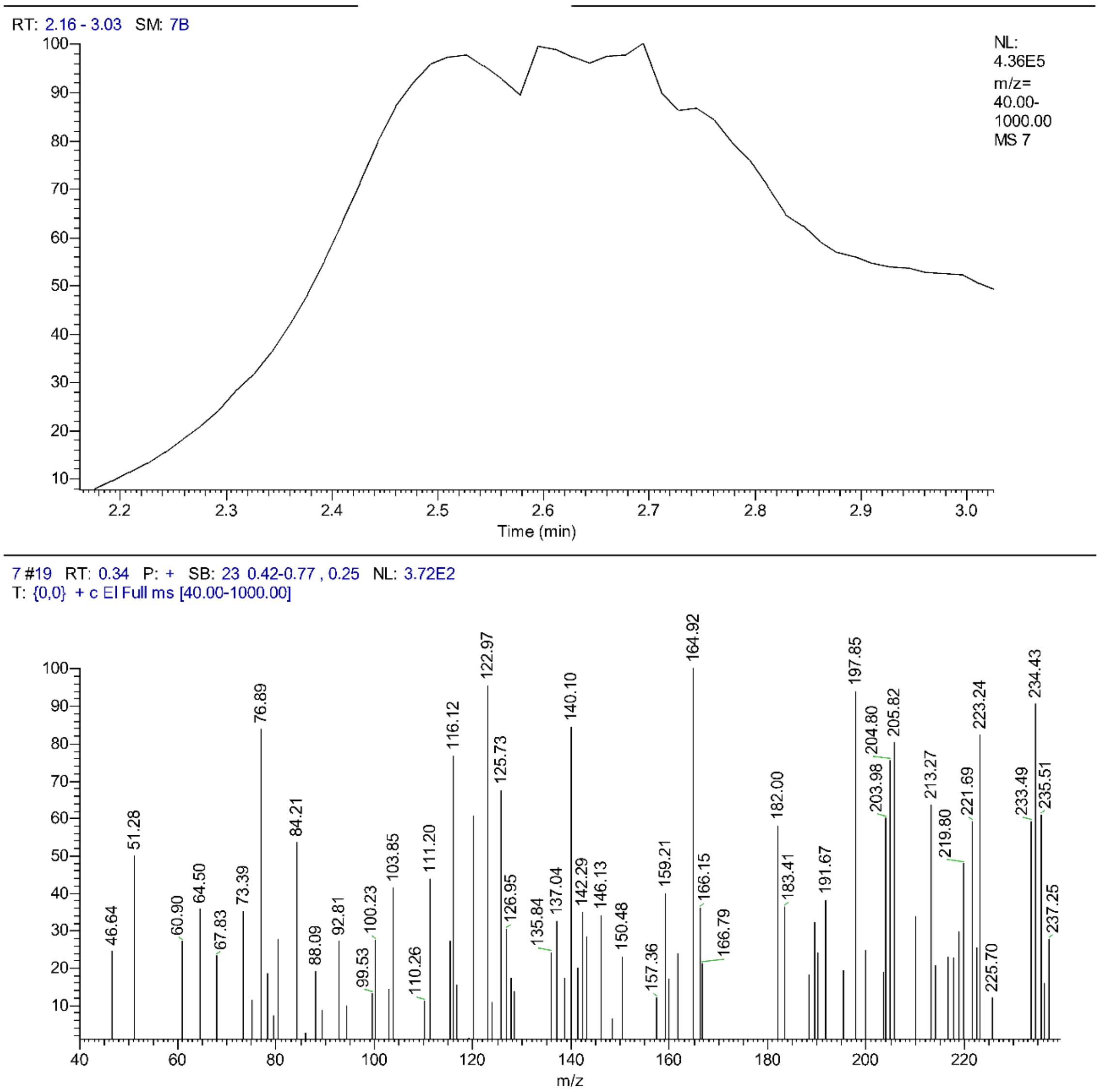

Figure S30. Mass spectrum of compound **7**

Characterization of Compound 8

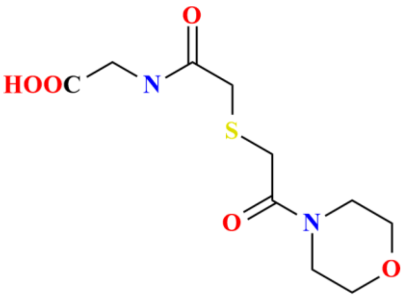

8

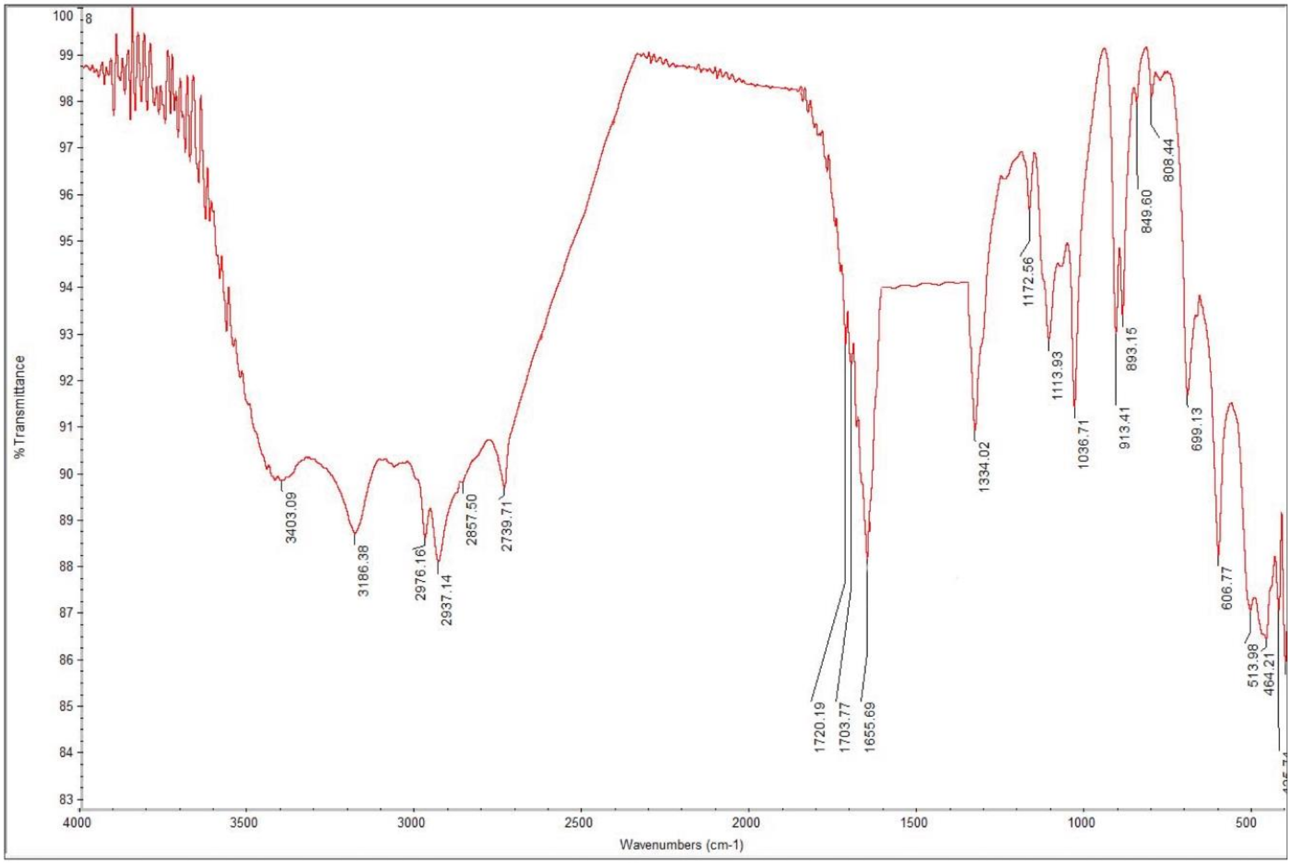

Figure S31. IR of Compound **8**

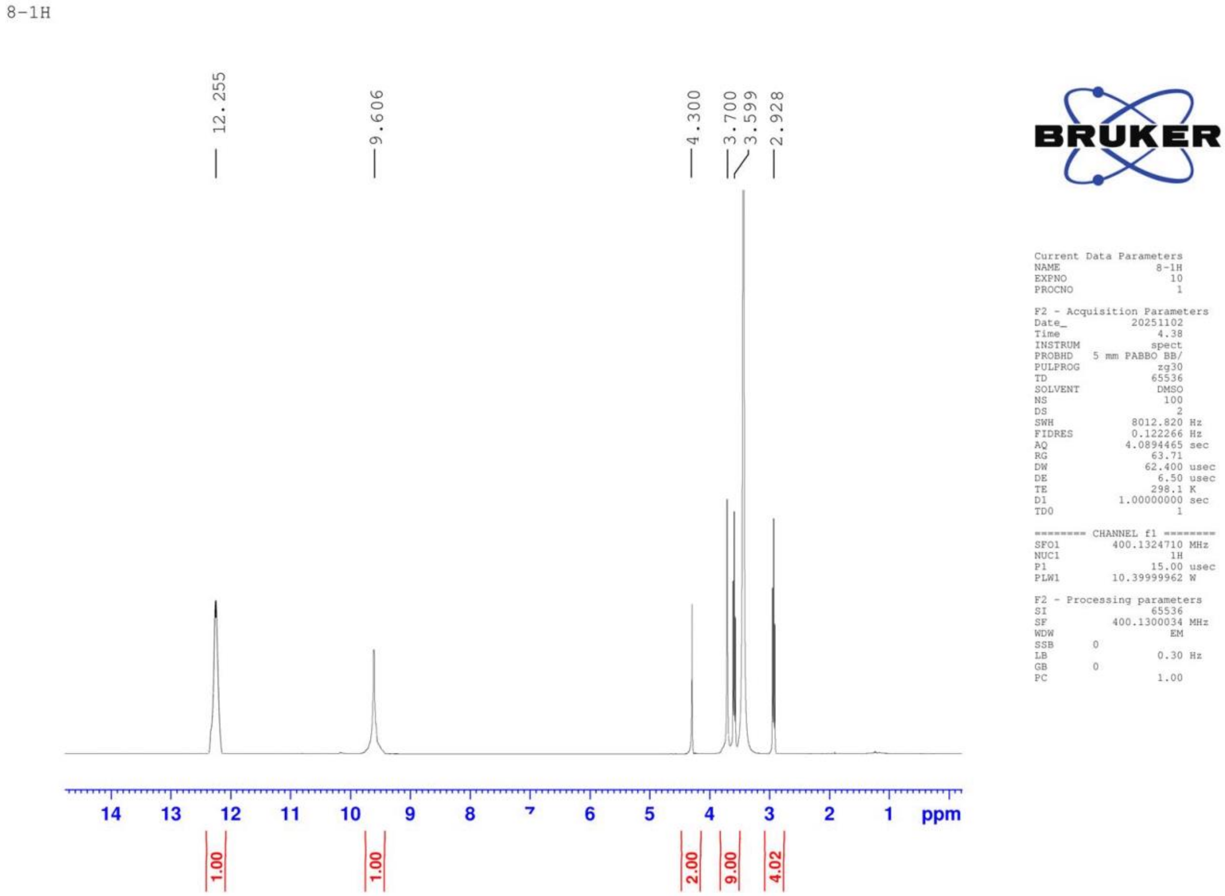

Figure S32. ^{1}H NMR spectrum (400 MHz, DMSO-*d6*) of compound **8**

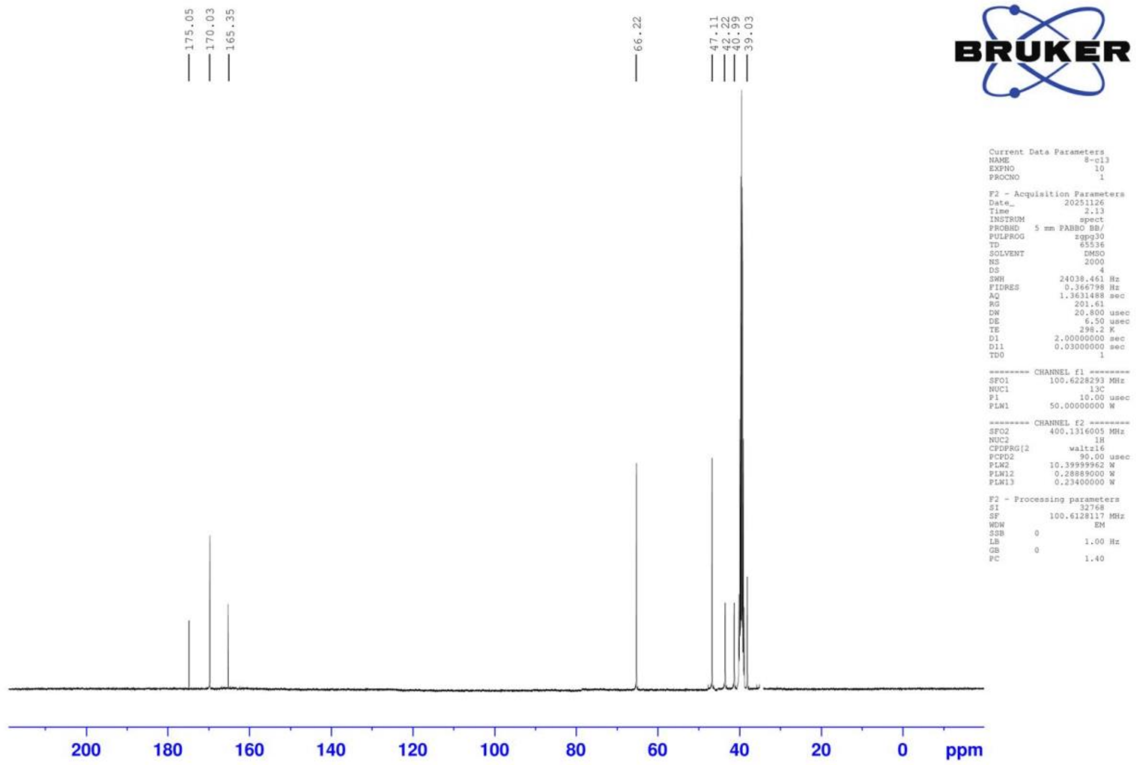

Figure S33. ^{13}C NMR spectrum (100 MHz, DMSO-*d6*) of compound **8**

Characterization of Compound 9

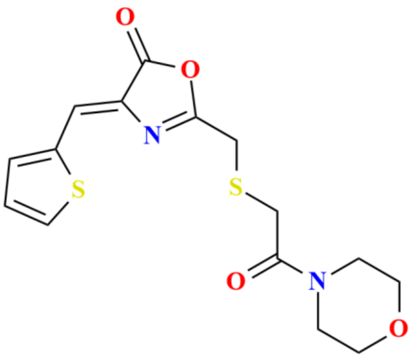

9

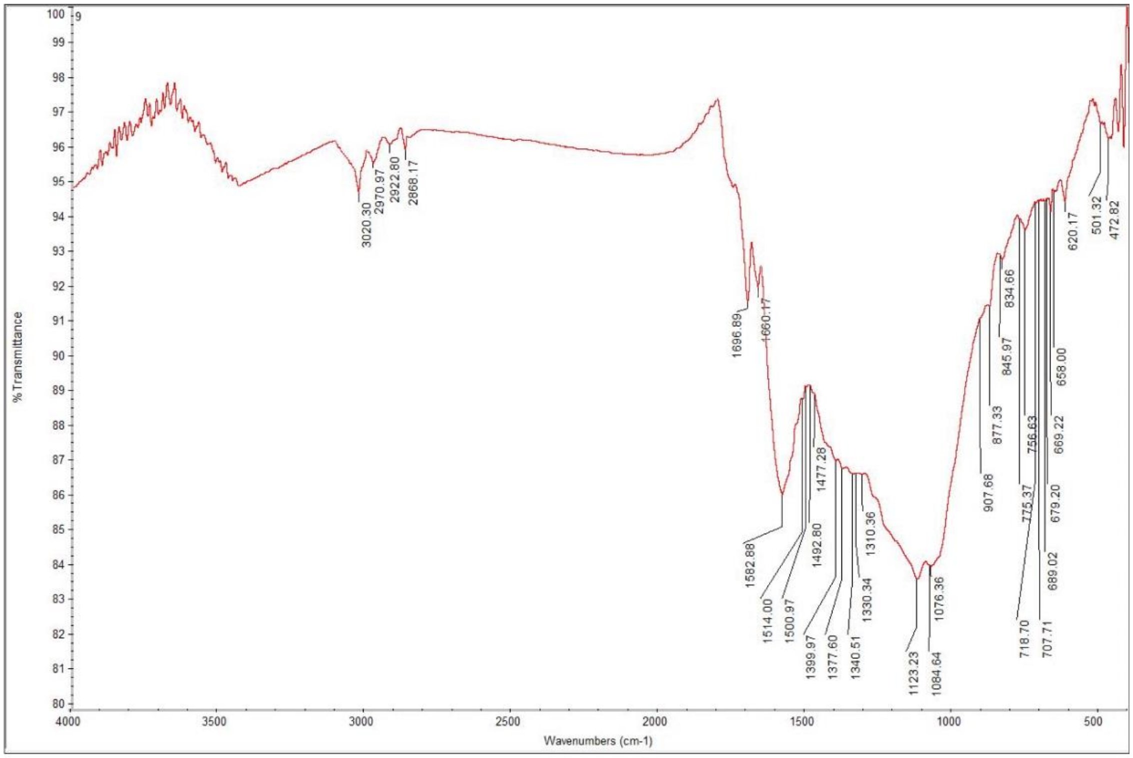

Figure S34. IR of Compound **9**

9 - 1H

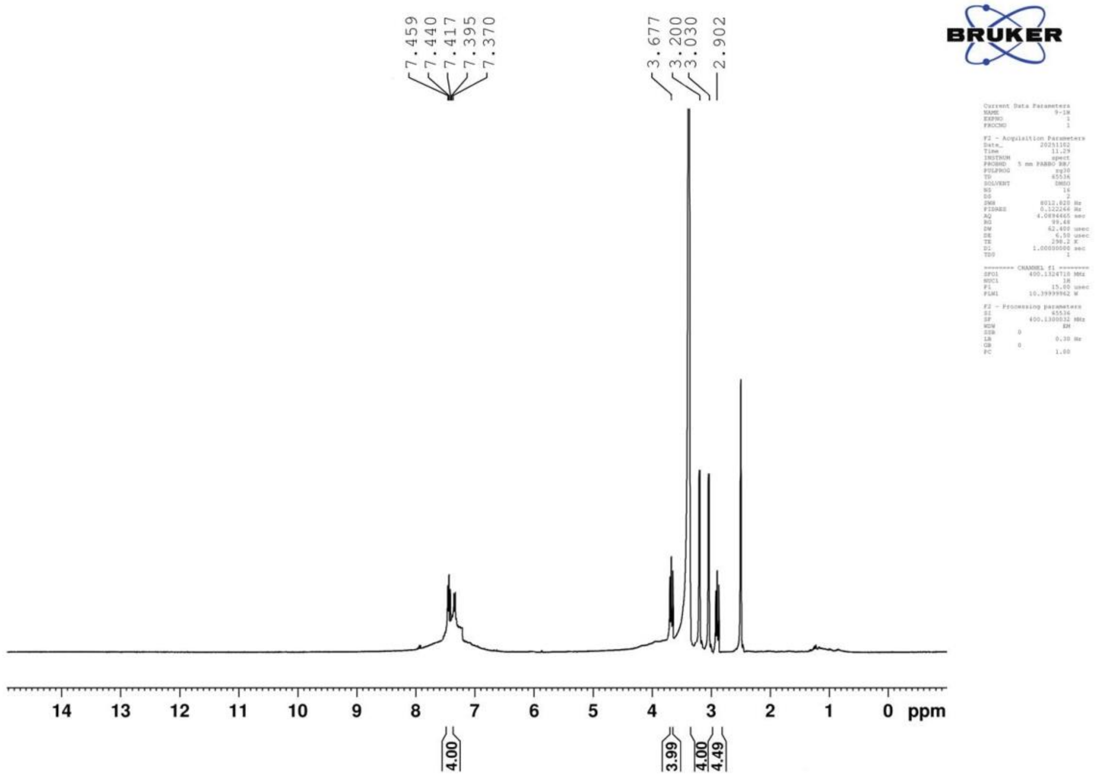

Figure S35. ^{1}H NMR spectrum (400 MHz, DMSO-*d6*) of compound **9**

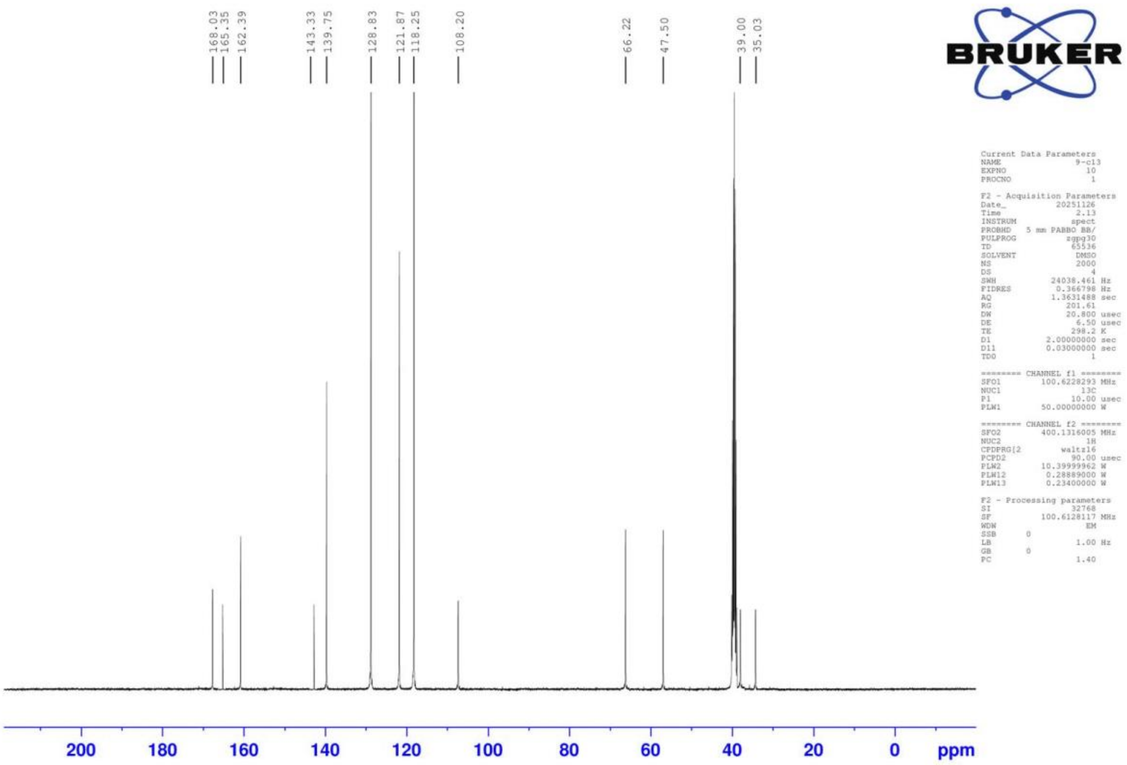

Figure S36. ^{13}C NMR spectrum (100 MHz, DMSO-*d6*) of compound **9**

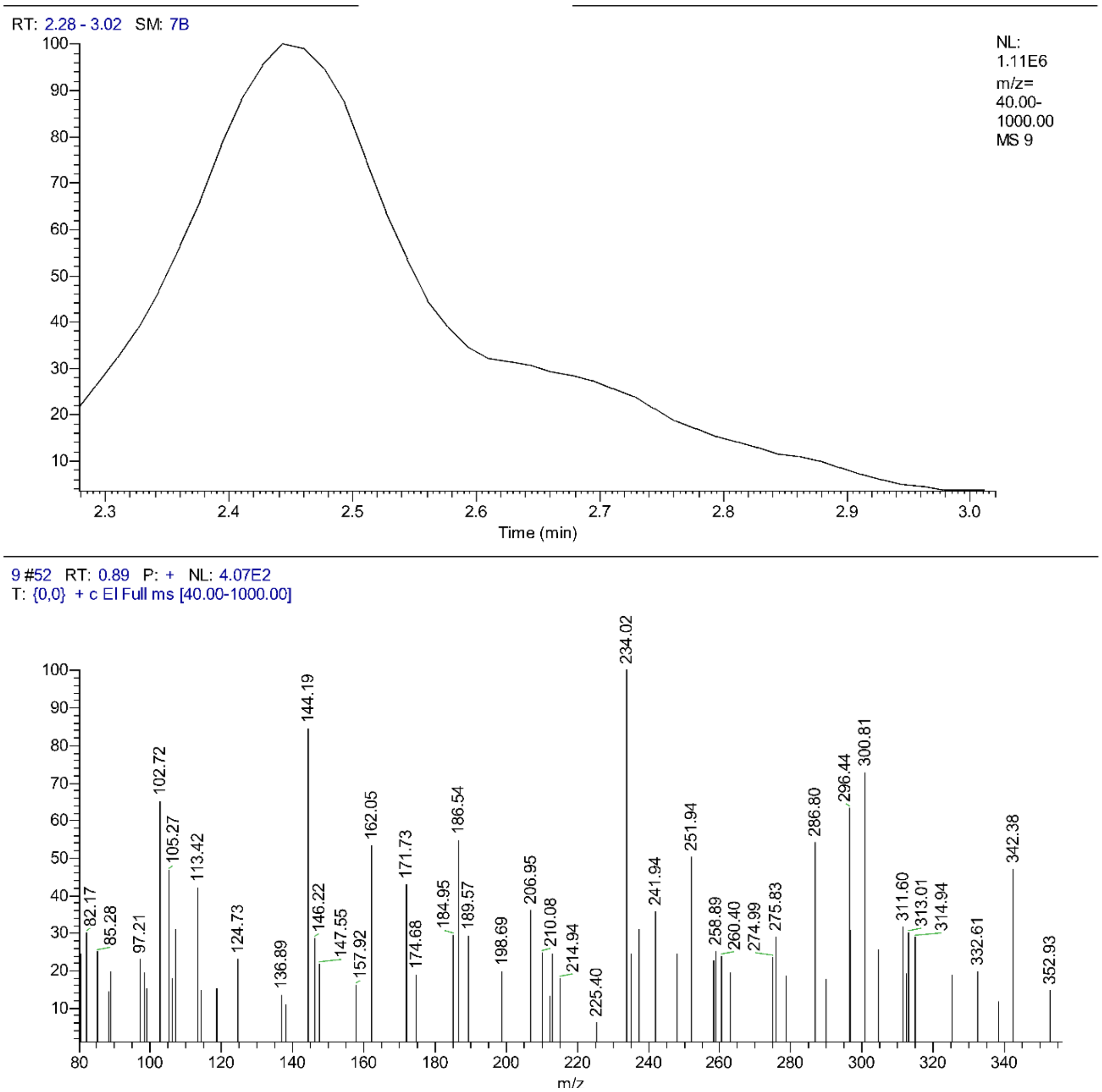

Figure S37. Mass spectrum of compound **9**

Characterization of Compound 10

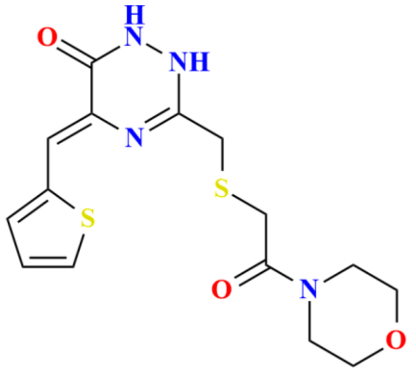

10

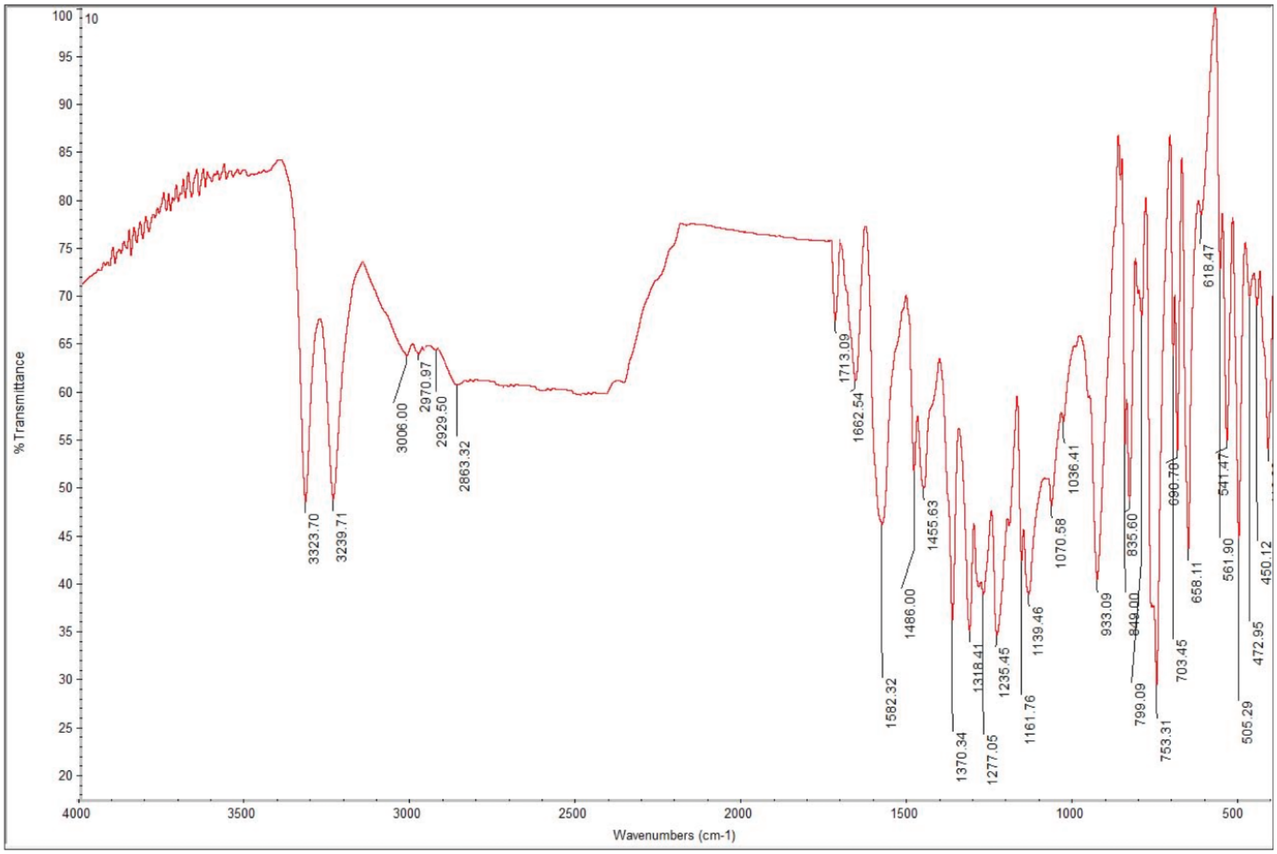

Figure S38. IR of Compound **10**

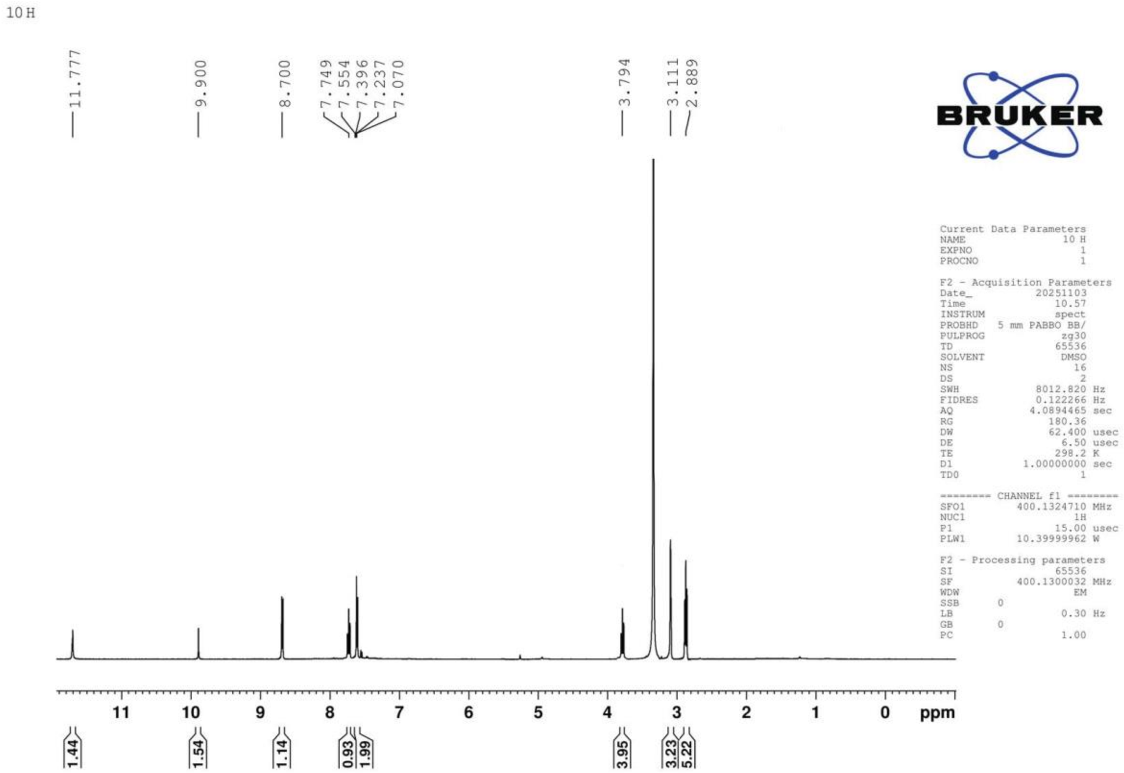

Figure S39. ^{1}H NMR spectrum (400 MHz, DMSO-*d6*) of compound **10**

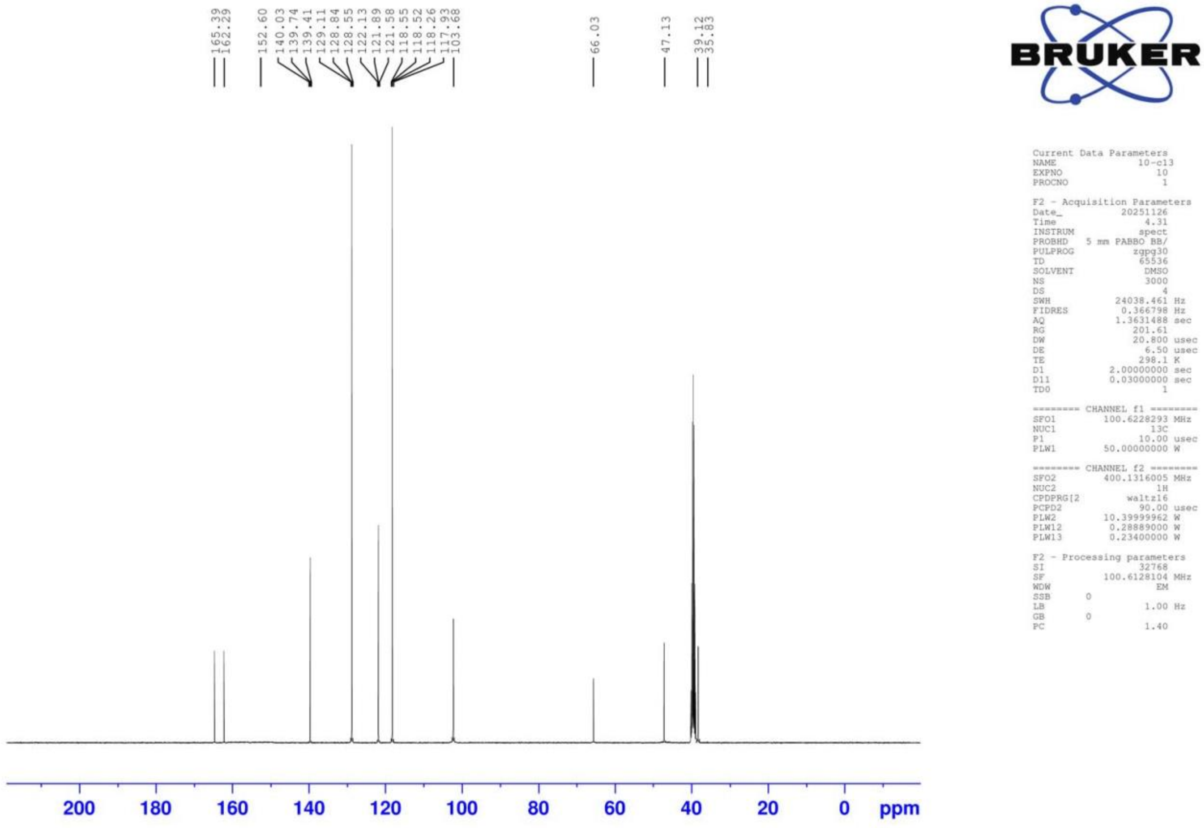

Figure S40. ^{13}C NMR spectrum (100 MHz, DMSO-*d6*) of compound **10**

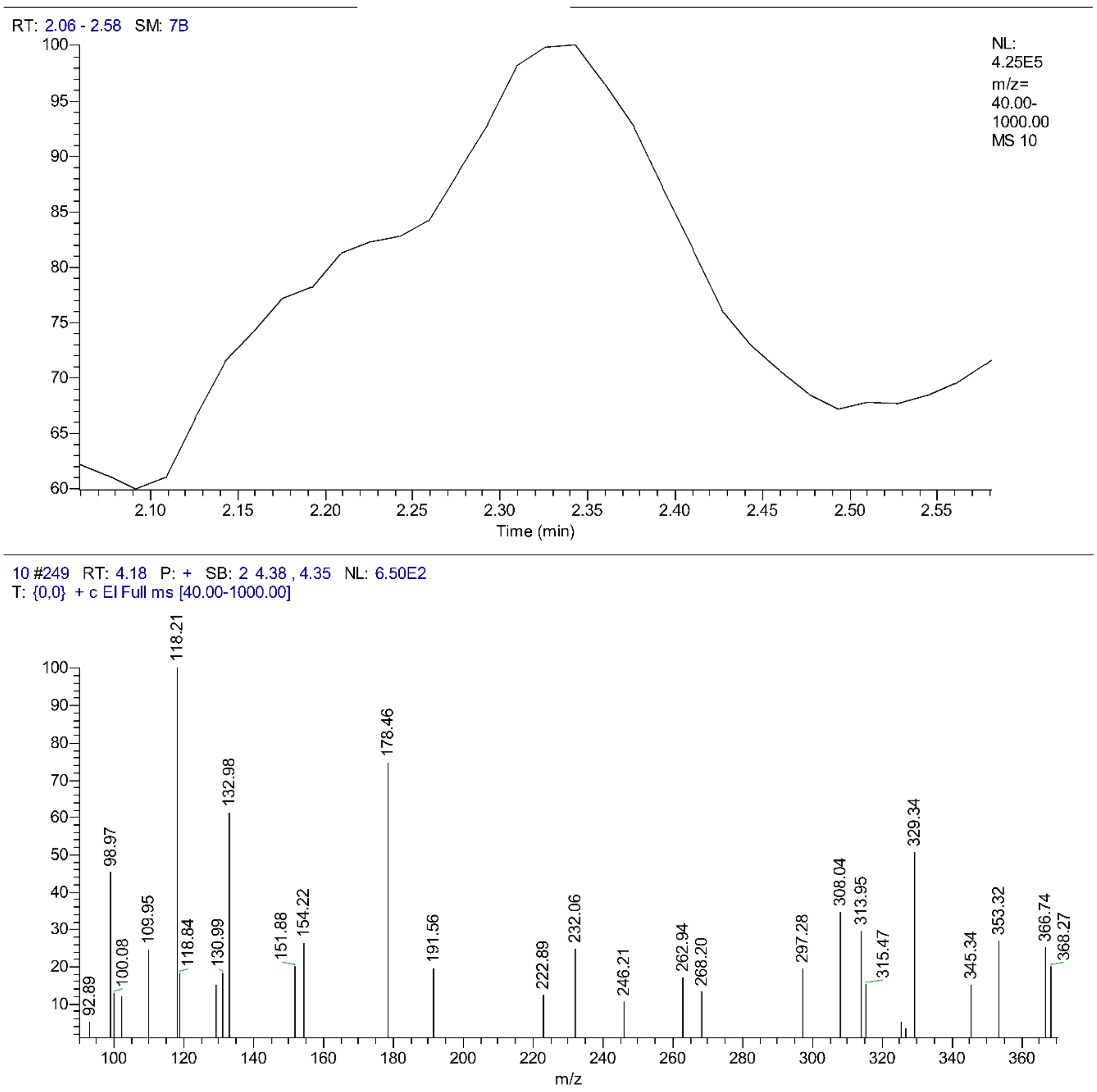

Figure S41. Mass spectrum of compound **10**

Characterization of Compound 11

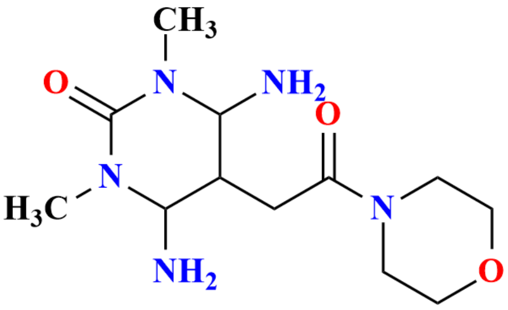

11

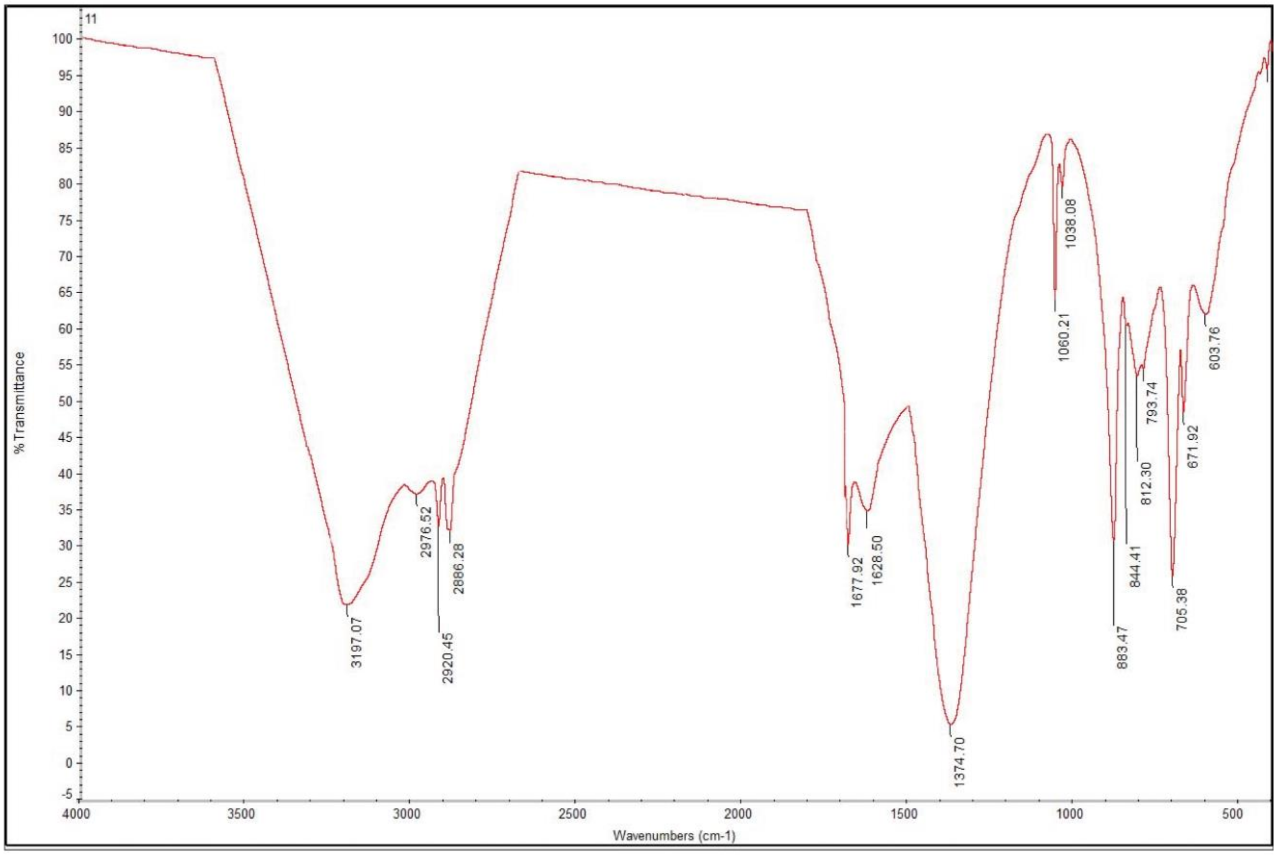

Figure S42. IR of Compound **11**

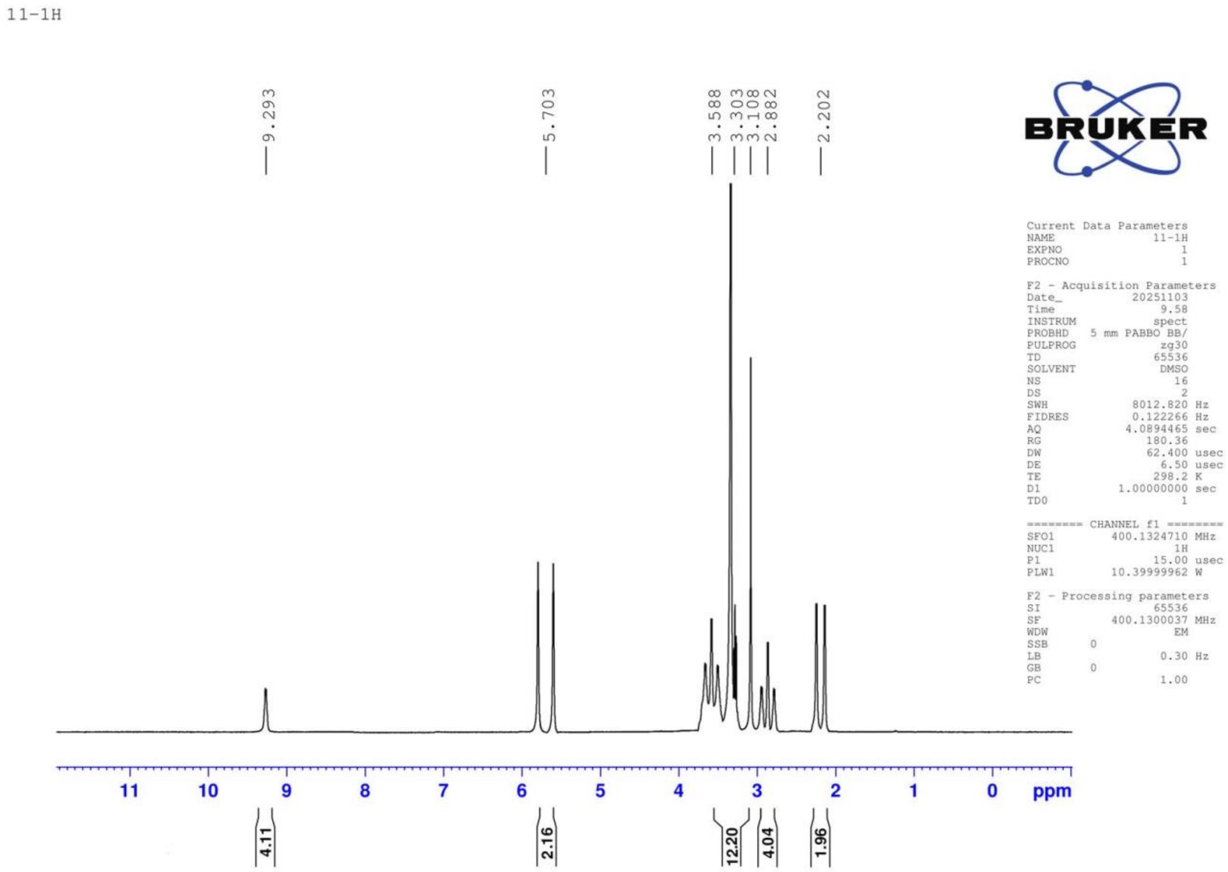

Figure S43. ^{1}H NMR spectrum (400 MHz, DMSO-*d6*) of compound **11**

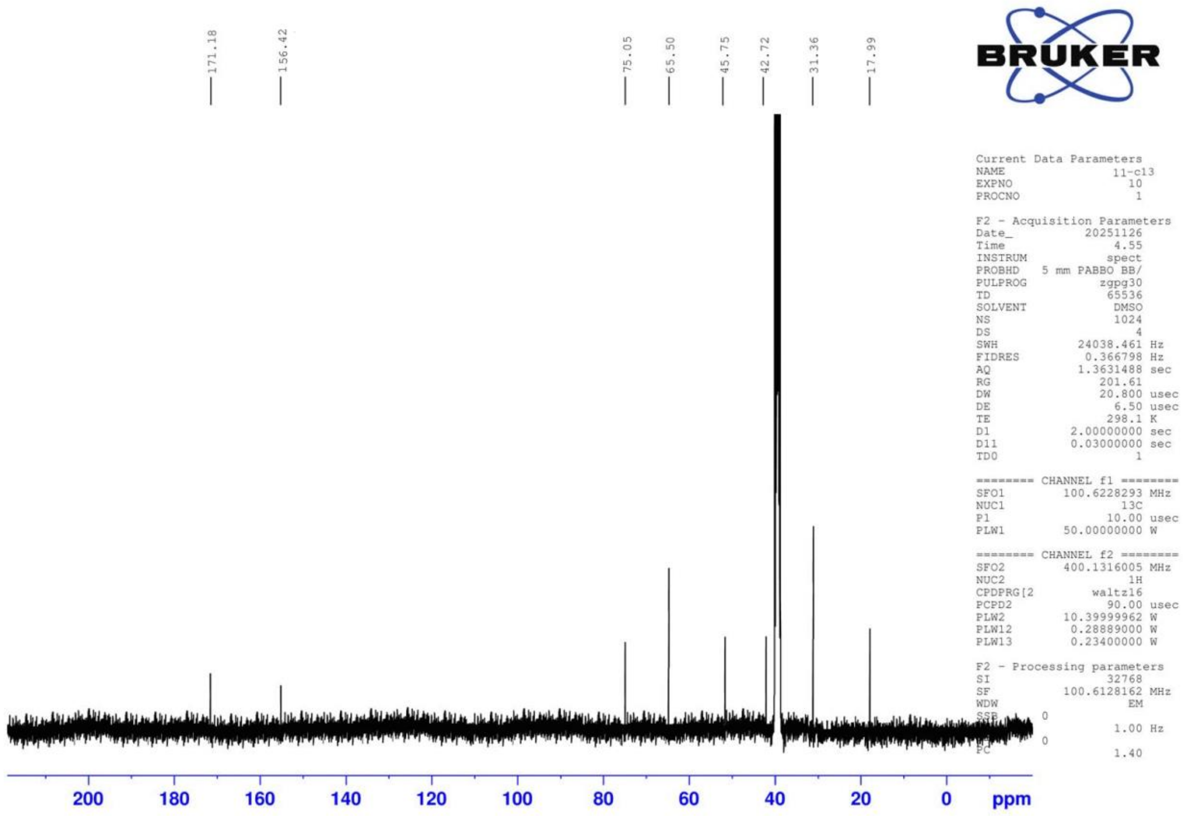

Figure S44. ^{13}C NMR spectrum (100 MHz, DMSO-*d6*) of compound **11**

Characterization of Compound 12

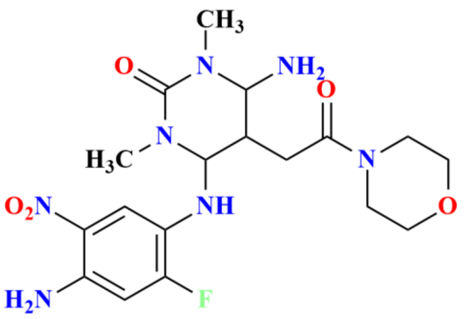

12

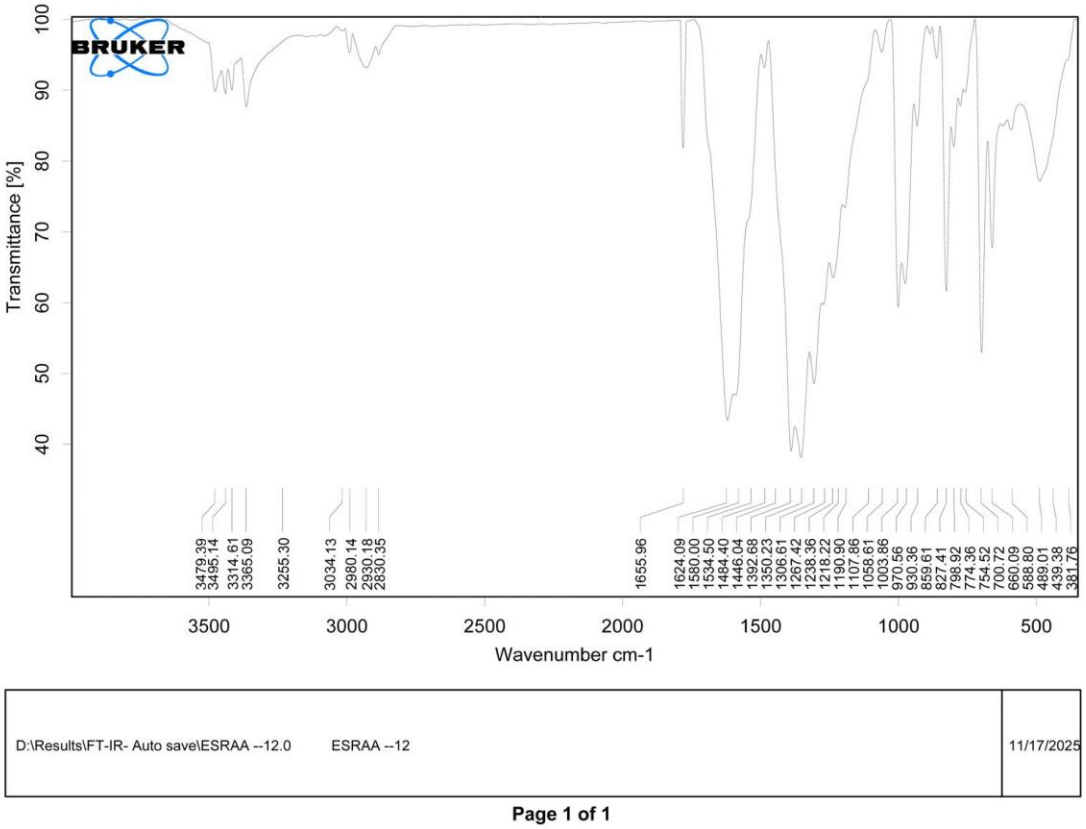

Figure S45. IR of Compound **12**

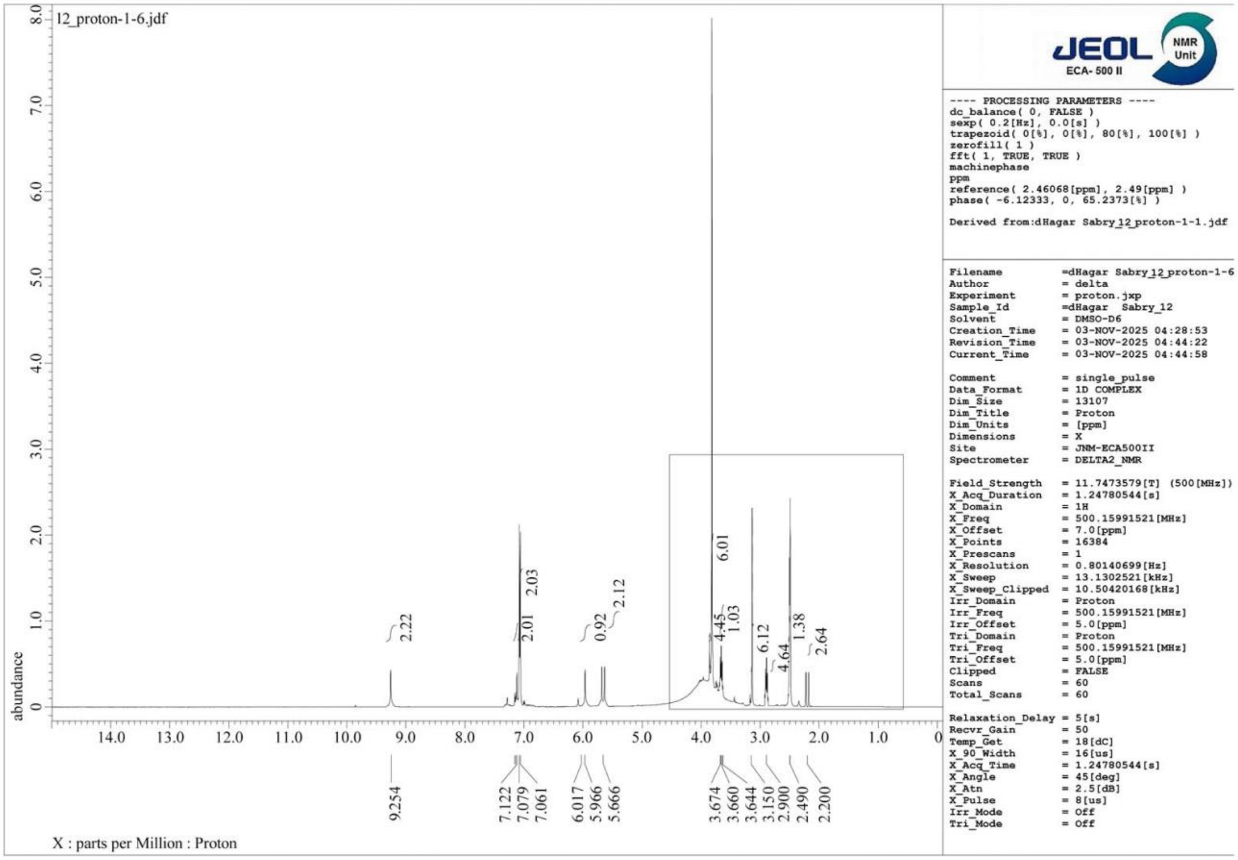

Figure S46. ^{1}H NMR spectrum (400 MHz, DMSO-*d6*) of compound **12**

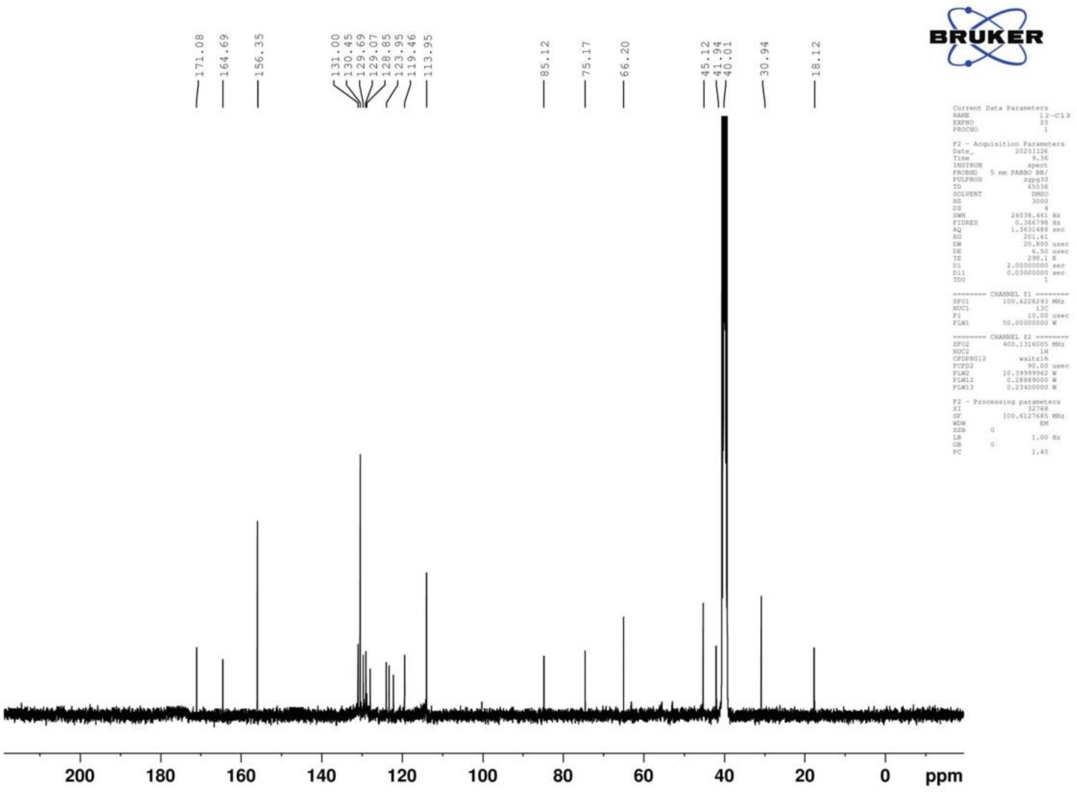

Figure S47. ^{13}C NMR spectrum (100 MHz, DMSO-*d6*) of compound **12**

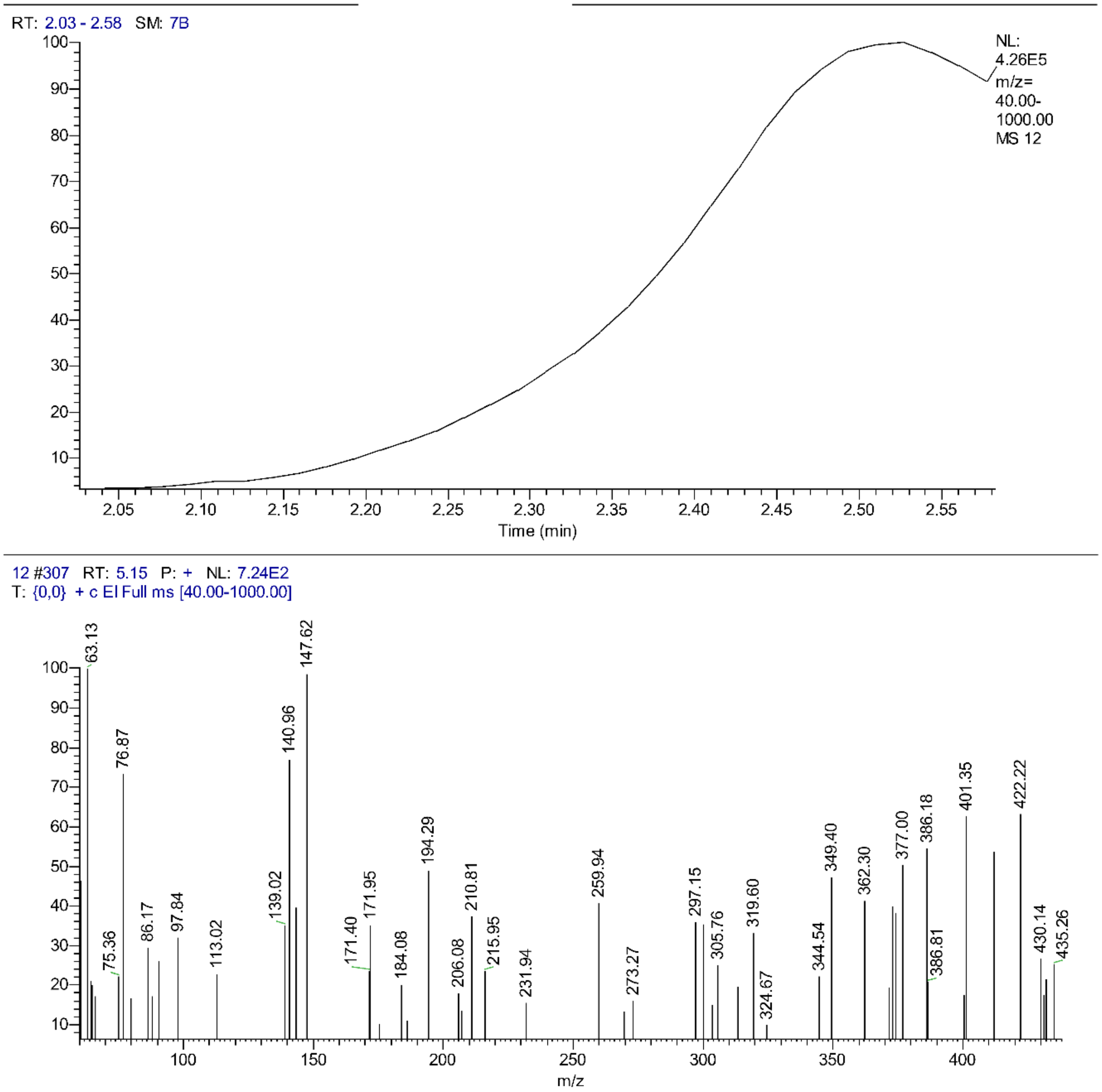

Figure S48. Mass spectrum of compound **12**

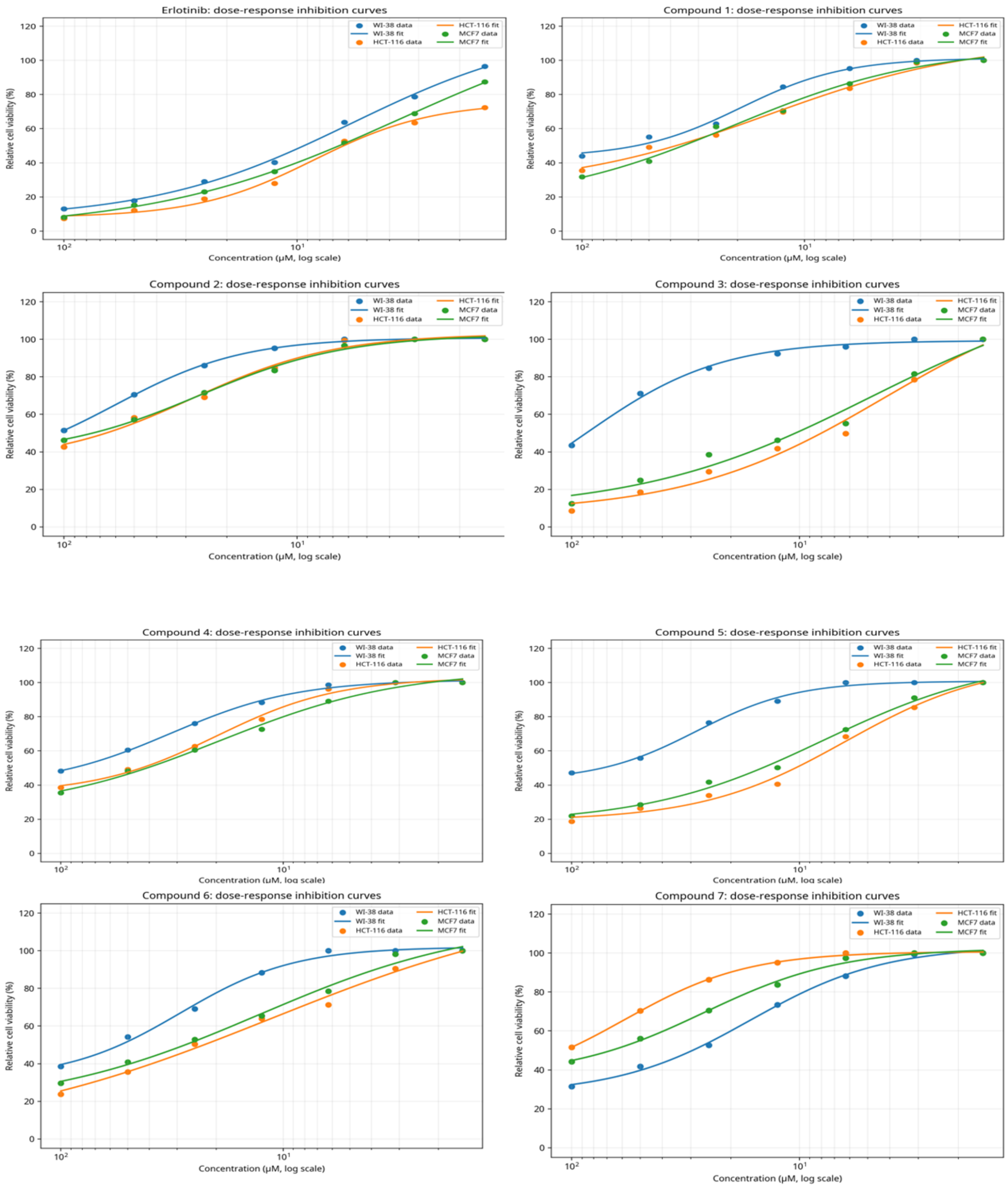
Erlotinib: dose-response inhibition curves
Compound 1: dose-response inhibition curves
Compound 2: dose-response inhibition curves
Compound 3: dose-response inhibition curves
Compound 4: dose-response inhibition curves
Compound 5: dose-response inhibition curves
Compound 6: dose-response inhibition curves
Compound 7: dose-response inhibition curves
WI-38 data
WI-38 fit
HCT-116 data
HCT-116 fit
MCF7 data
MCF7 fit
Relative cell viability (%)
Concentration (μM, log scale)

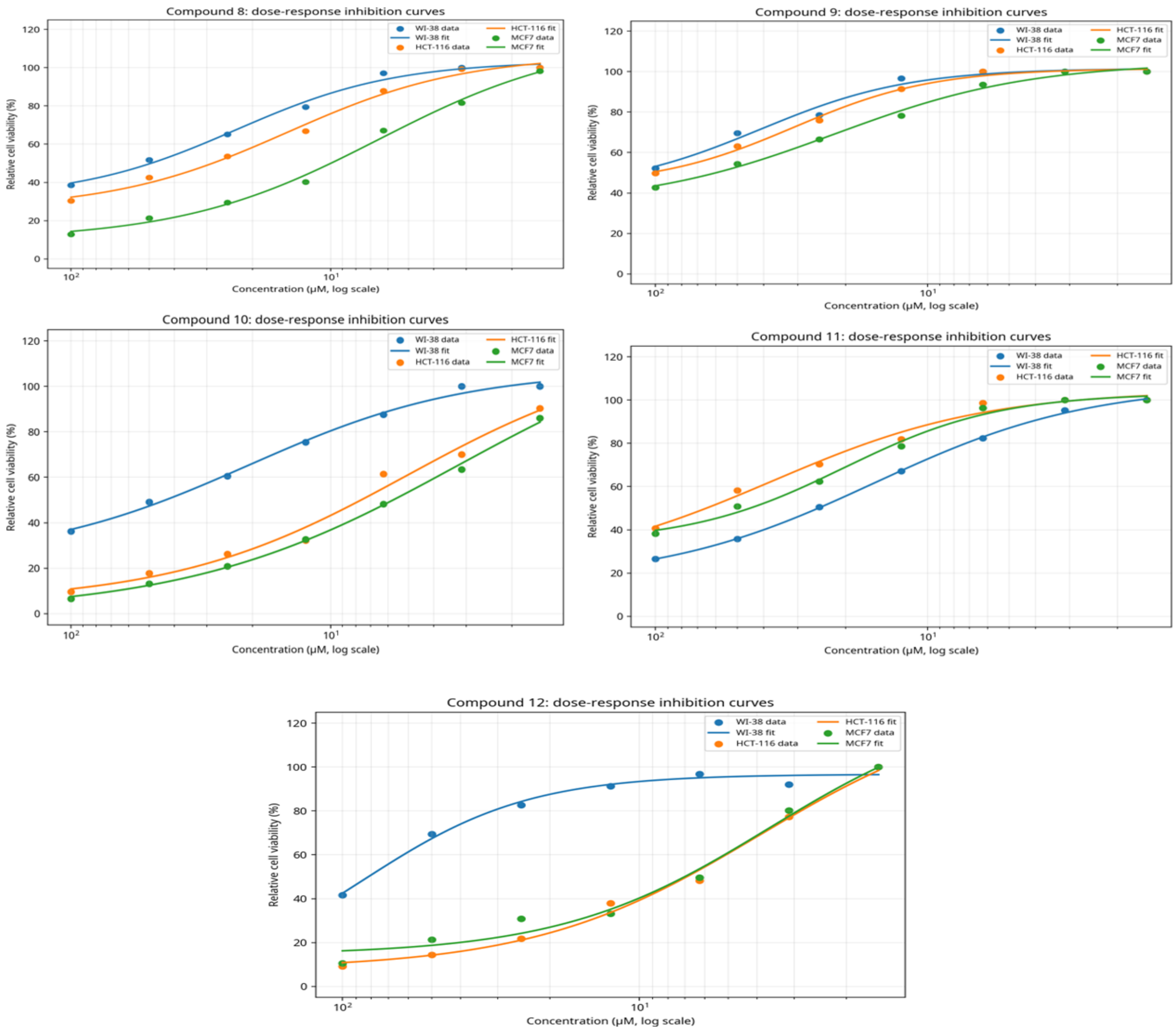

Figure S49. Concentration-dependent effects of morpholine-based derivatives **1–12** and erlotinib on the viability of HCT-116, MCF-7, and WI-38 cells. Relative cell viability (%) is plotted against compound concentration (μM).

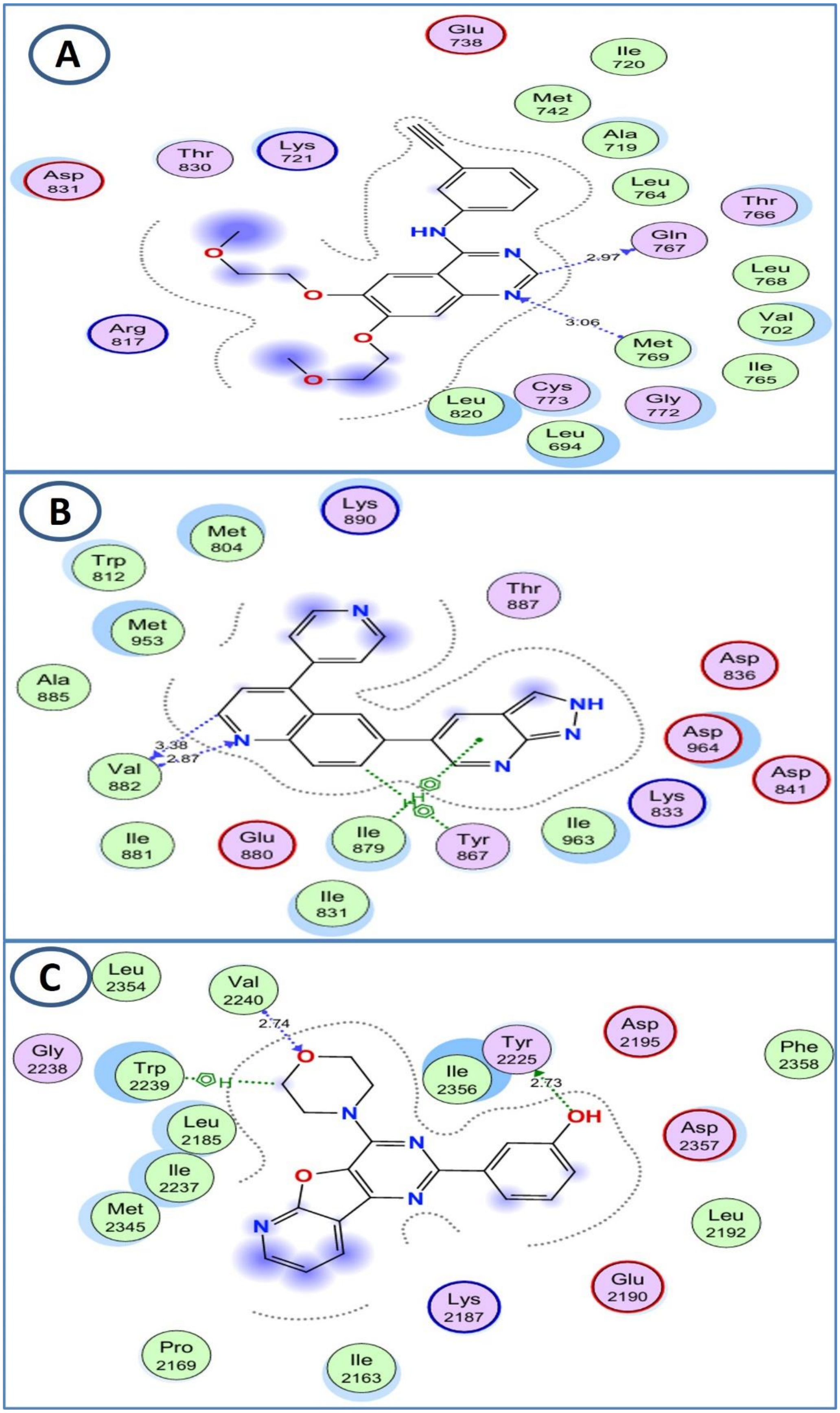
A
Glu 738
Ile 720
Met 742
Ala 719
Thr 830
Lys 721
Asp 831
Leu 764
Thr 766
HN
N
Gln 767
2.97
Leu 768
O
N
3:06
Val 702
Arg 817
O
Met 769
Ile 765
O
Leu 820
Cys 773
Gly 772
Leu 694
B
Lys 890
Met 804
Trp 812
Thr 887
Met 953
N
Asp 836
Ala 885
NH
N
Asp 964
3.38
N
2.87
N
Asp 841
Val 882
H
Lys 833
Ile 963
Ile 881
Glu 880
Ile 879
Tyr 867
Ile 831
C
Leu 2354
Val 2240
2.74
Asp 2195
Gly 2238
O
Tyr 2225
Phe 2358
Trp 2239
H
Ile 2356
2.73
OH
N
Leu 2185
N
Asp 2357
Ile 2237
O
N
Met 2345
N
Leu 2192
Glu 2190
Lys 2187
Pro 2169
Ile 2163

Figure S50. A, B, and C diagrams show two-dimensional views of redocking the co-crystallized ligands erlotinib, 6-(1*H*-pyrazolo[3,4-*b*]pyridin-5-yl)-4-pyridin-4-ylquinoline (LXX), and 3-(4-morpholin-4-ylpyrido[3',2':4,5]furo[3,2-*d*]pyrimidin-2-yl)phenol (X6K), within the EGFR, PI3K, and mTOR active sites (PDB IDs: 1M17, 3L54, and 4JT6, respectively).

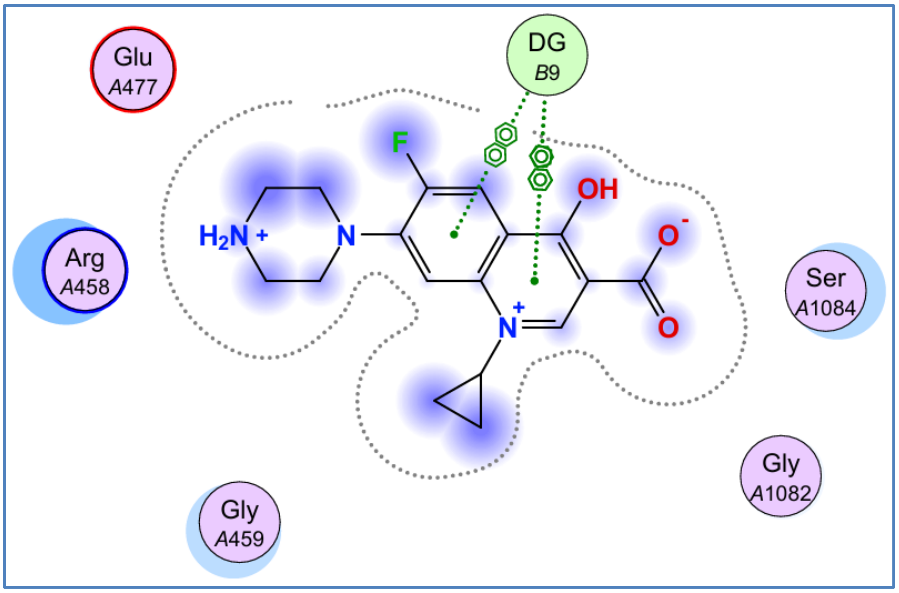

Figure S51. Two-dimensional view of redocking the co-crystallized ligand ciprofloxacin, within the *S. aureus* DNA gyrase active site (PDB code: 2XCT).

Compound	OPT	ESP

1

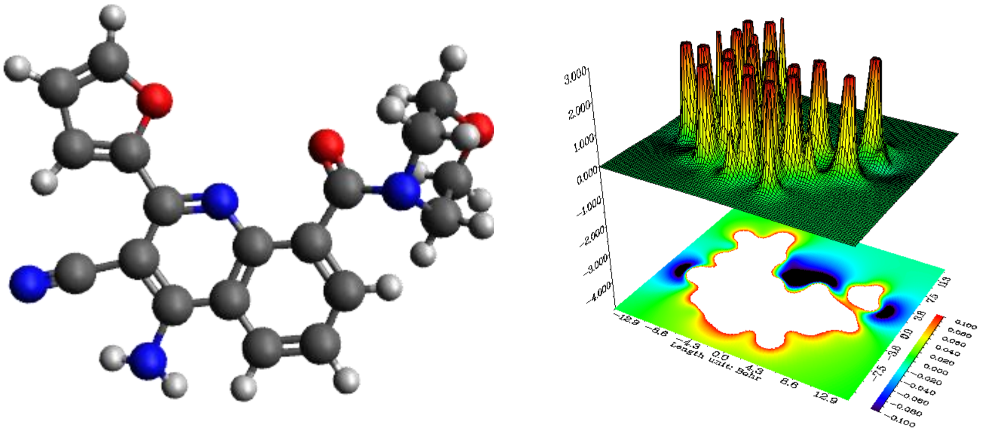

2

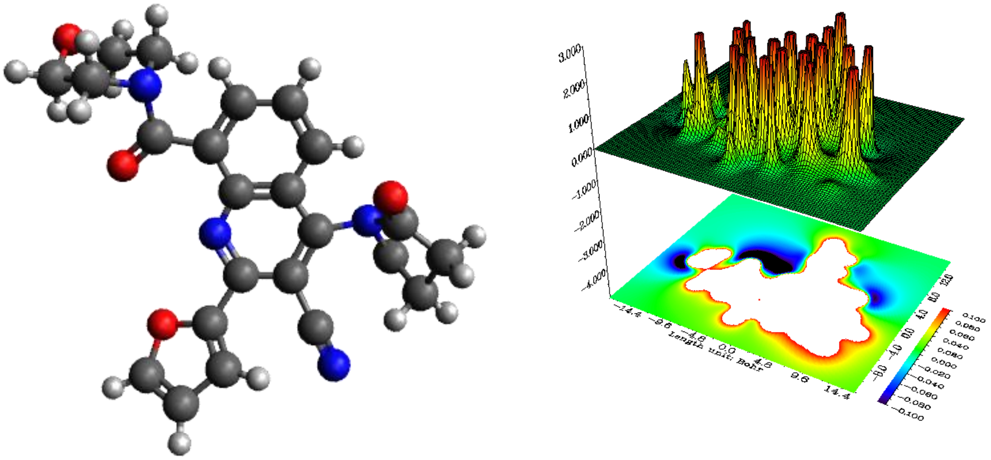

3

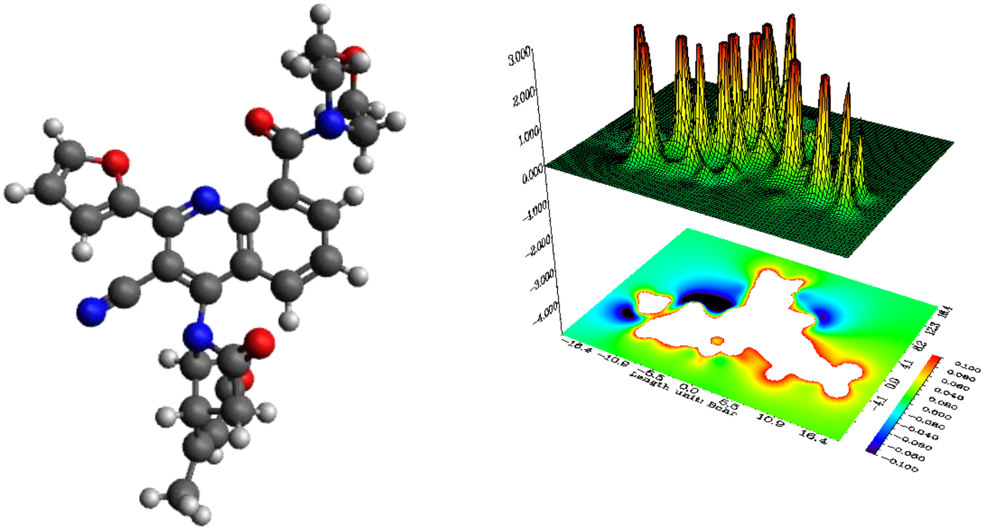

4

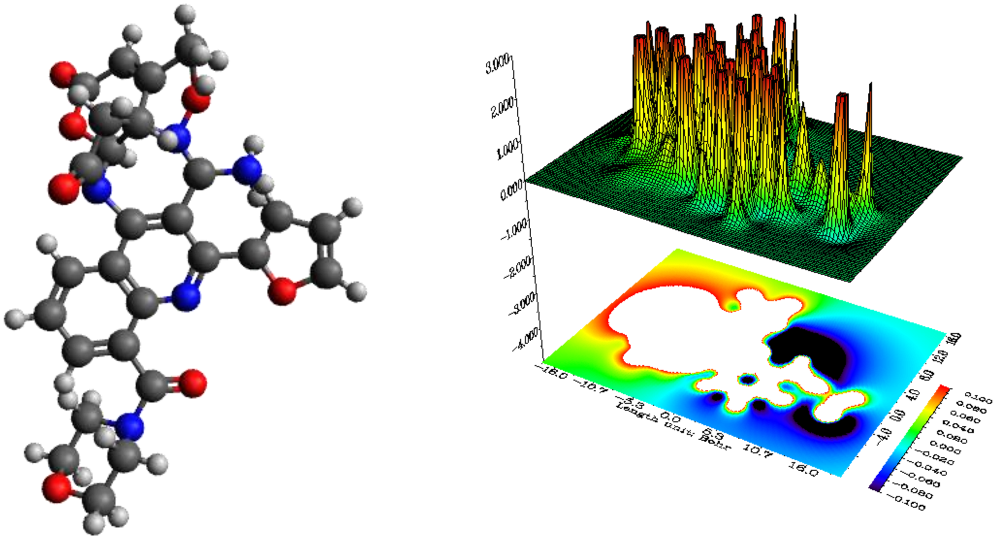

5

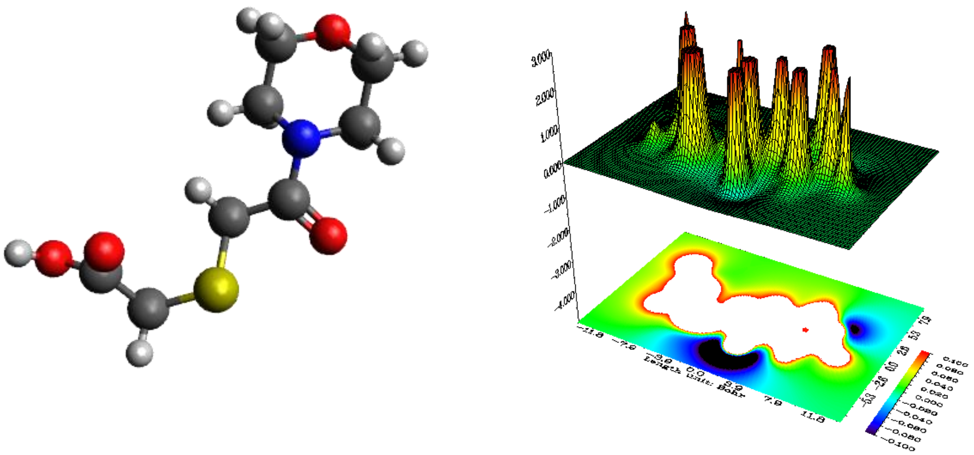

6

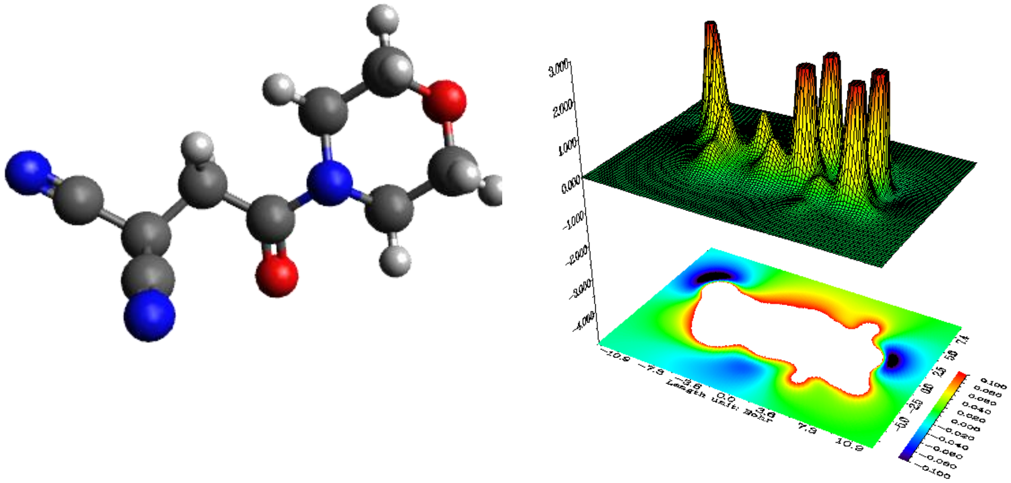

7

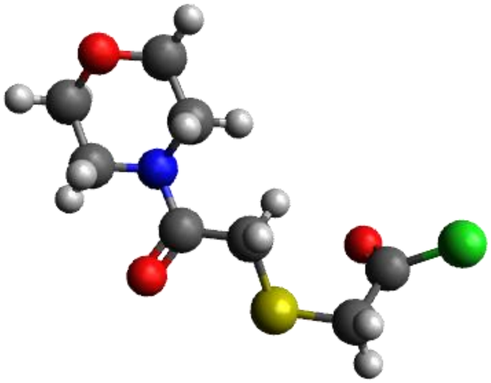

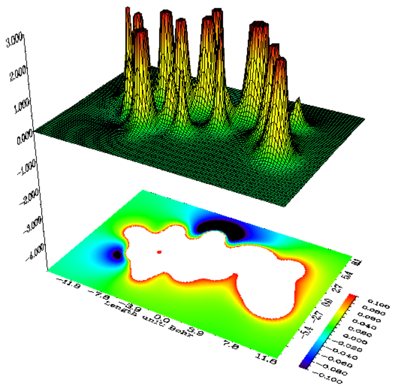

8

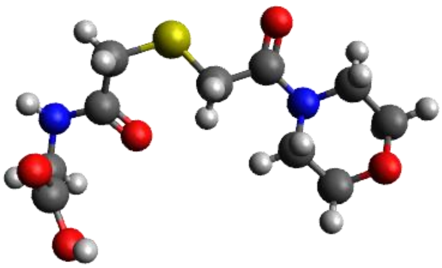

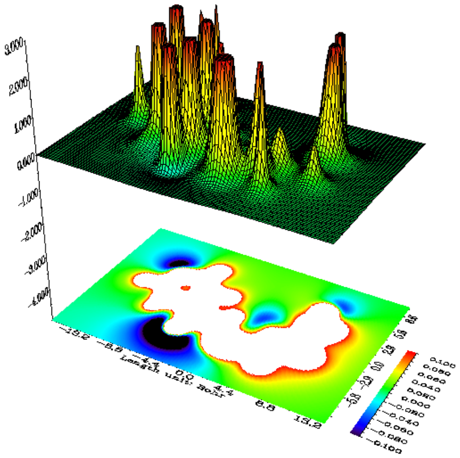

9

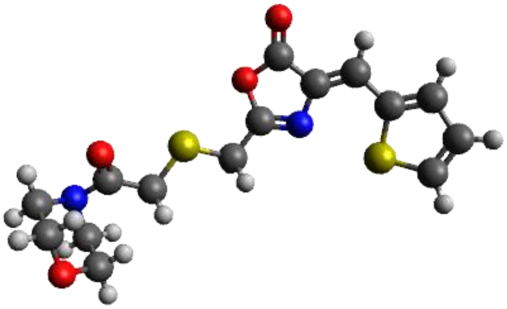

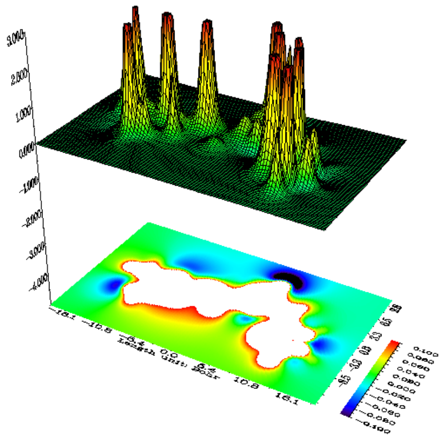

10

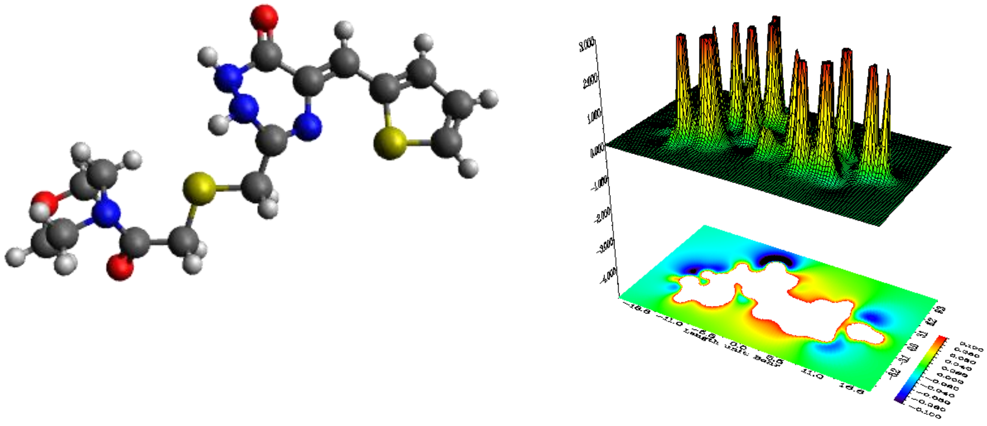

11

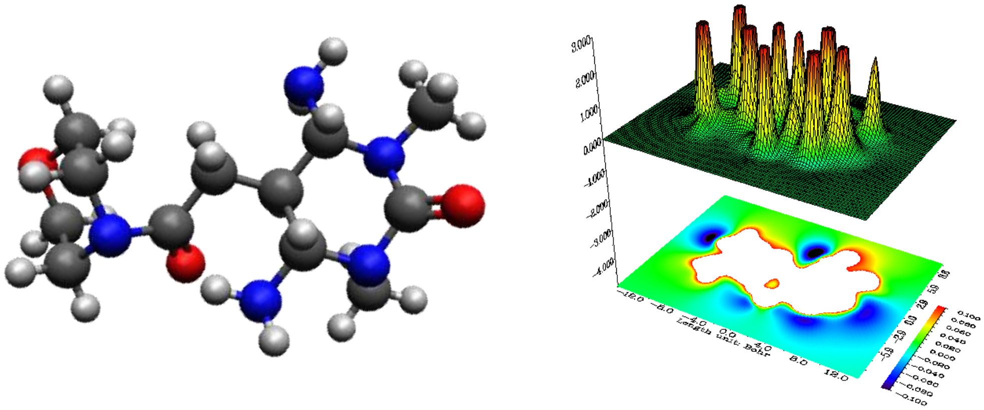

12

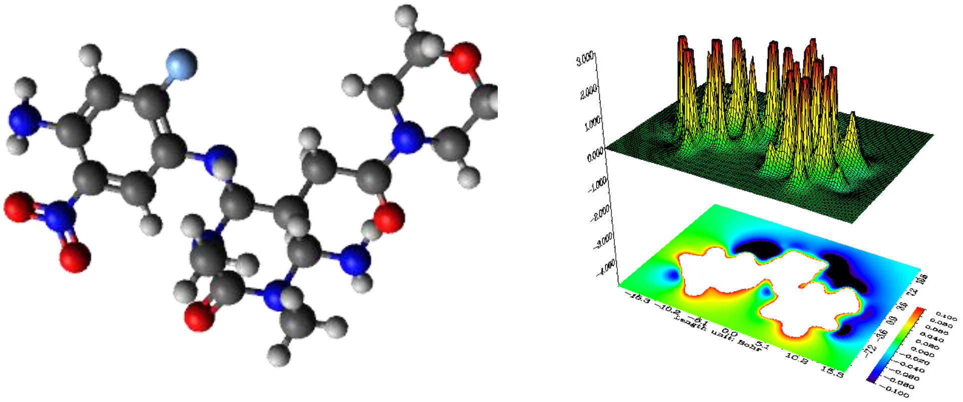

Figure S52. DFT-optimized geometries (OPT) and molecular electrostatic potential (ESP) surface maps of morpholine-based derivatives **1–12**.
